# Methylation deregulation of miRNA promoters identifies miR124-2 as a survival biomarker in Breast Cancer in very young women

**DOI:** 10.1038/s41598-018-32393-3

**Published:** 2018-09-26

**Authors:** Sara S. Oltra, Maria Peña-Chilet, Victoria Vidal-Tomas, Kirsty Flower, María Teresa Martinez, Elisa Alonso, Octavio Burgues, Ana Lluch, James M. Flanagan, Gloria Ribas

**Affiliations:** 1 INCLIVA Biomedical Research Institute, Hospital Clínico Universitario Valencia, University of Valencia, Valencia, Spain; 2 Department of Surgery and Cancer, Imperial College London, London, UK; 3 Pathology Department, Hospital Clínico Universitario Valencia, University of Valencia, Valencia, Spain; 4 Center for Biomedical Network Research on Cancer, Valencia, Spain

## Abstract

MiRNAs are part of the epigenetic machinery, and are also epigenetically modified by DNA methylation. MiRNAs regulate expression of different genes, so any alteration in their methylation status may affect their expression. We aimed to identify methylation differences in miRNA encoding genes in breast cancer affecting women under 35 years old (BCVY), in order to identify potential biomarkers in these patients. In Illumina Infinium MethylationEPIC BeadChip samples (metEPICVal), we analysed the methylation of 9,961 CpG site regulators of miRNA-encoding genes present in the array. We identified 193 differentially methylated CpG sites in BCVY (*p-value* < 0.05 and methylation differences ±0.1) that regulated 83 unique miRNA encoding genes. We validated 10 CpG sites using two independent datasets based on Infinium Human Methylation 450k array. We tested gene expression of miRNAs with differential methylation in BCVY in a meta-analysis using The Cancer Genome Atlas (TCGA), Clariom D and Affymetrix datasets. Five miRNAs (miR-9, miR-124-2, miR-184, miR-551b and miR-196a-1) were differently expressed (FDR *p-value* < 0.01). Finally, only miR-124-2 shows a significantly different gene expression by quantitative real-time PCR. MiR-124-hypomethylation presents significantly better survival rates for older patients as opposed to the worse prognosis observed in BCVY, identifying it as a potential specific survival biomarker in BCVY.

## Introduction

Breast cancer has the highest incidence rate of all cancers in women worldwide^1^. Although early breast cancer generally has an excellent prognosis, breast cancer in young women is associated with a high risk of systemic disease at long-term follow-up^2–7^. Young women tend to be diagnosed at a later stage with highly proliferative, high-grade tumours with the presence of lymphovascular invasion^3–5,8–12^.

Hypomethylation may result in aberrant or inappropriate gene expression that contributes to neoplastic transformation, tumorigenesis or cancer progression (oncogenes)^13^. In addition, genome-wide loss of methylation contributes to chromosomal instability by destabilizing pericentromeric regions of certain chromosomes^14–16^. Gene-specific hypermethylation typically reflects hypermethylation of CpG-rich regions within gene promoter sequences that lead to gene silencing events^17^.

MicroRNAs (miRNAs) are short non-coding RNAs that act as important regulators of gene expression as part of the epigenetic machinery. Epigenetic modifications were reported to play an important role in many disease onsets and progressions and can be used to explain several features of complex diseases, such as late onset and fluctuation of symptoms. In addition, miRNAs not only function as part of the epigenetic machinery but can also be epigenetically modified by DNA methylation and histone modifications themselves like any other gene. Methylation regulates the CpG islands on miRNA promoters altering their expression, the histone modifications affecting chromatin structure or changing the affinities for chromatin-associated proteins, thereby modulating gene expression, and therefore, miRNA gene expression. On the other hand, miRNAs can directly target epigenetic factors, such as DNA methyltransferases or histone deacetylases, thus regulating chromatin structure. Moreover, several studies have reported coordinated actions between miRNAs and other epigenetic mechanisms to reinforce the regulation of gene expression^18^. There is a strong connection between epigenome and miRNome, and any dysregulation of this complex system can result in various physiological and pathological conditions^19^.

The aim of the current study is to analyse the methylation alterations of CpG associated with miRNA encoding genes in breast cancer tumours in very young women (≤35 years old) and older ones (>50 years old). DNA methylation was analysed using Illumina Infinium HumanMethylation EPIC array^20^ (EPICarray); this array measures DNA methylation at approximately 850 000 CpG sites across the genome and replace the previous Infinium HumanMethylation450 BeadChip, which analyses 450,000 CpG sites. The EPICarray incorporates CpG sites located in enhancer regions identified by the ENCODE^21^ and FANTOM5^22^ projects. We hypothesized that methylation differences in miRNA-encoding genes could also represent gene expression differences. This could be a contributing factor in the poorer outcome of tumours in young women.

## Results

### Methylation differences in CpG probes regulating gene-encoding miRNAs

**Figure 1:**
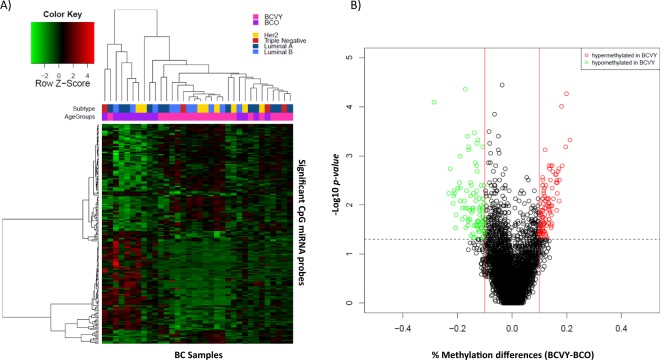
Differential miRNA methylation study in BCVY vs. BCO from metEPICVal samples. (**A**) Heatmap representing a supervised cluster centred on the median of the methylation levels at the 193 CpG sites that regulated miRNA genes distinctive in BCVY. Hypermethylated CpG probes in BCVY (red) and hypomethylated probes (green). Samples represented as BCVY (light pink) and BCO samples (purple). Molecular subtypes are indicated as: Her2 (yellow), triple negative (red), luminal A (dark blue) and luminal B (light blue). (**B**) Volcano-plot representation of methylation for significant CpG regulators of miRNA genes. Hypomethylated probes in BCVY are represented in green colour and hypermethylated probes in BCVY are represented in red. Red lines delimit ±0.1 methylation differences between BCVY vs. BCO and the dotted line represents a *p-value* threshold of 0.05.

We analysed data from 6,567 CpG sites regulators of miRNA encoding genes present in the Illumina Infinium MethylationEPIC BeadChip in BC samples from Hospital Clínico Universitario of Valencia (metEPICVal). Wilcoxon rank sum test shown 193 CpG probes that were significantly differentially methylated in BCVY (*p-value* < 0.05 and methylation differences ±0.1) and that regulated 83 unique miRNA encoding genes. Among them, 90 were hypomethylated and 103 hypermethylated in BCVY (Fig. 1A,B). Significant differentially methylated CpG sites obtained are included in Supplementary Table 1. However, hierarchical clustering showed two principal sample groups: one consisting of BCVY samples and other including BCO, four BCO samples clustered with BCVY patients. Generalized linear model (GLM) showed that methylation differences distinguishing BCVY from BCO were not related to ER status (*p-value* = 0.06) or molecular subtypes (*p-values* = 0.65) and no molecular subtype clusters were identified in heatmap representation (Fig. 1A). All data has been analysed again with the addition of five samples from healthy female donors, showing highly similar results (data not shown) proving that differences found are those related to breast cancer affecting young women and not due just produced by age differences.

### Genomic and functional context of significant CpG sites

**Figure 2:**
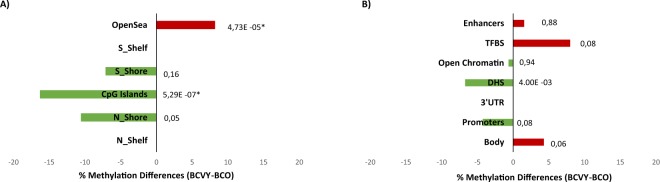
Genomic and functional context of significant CpG site regulators of genes encoding miRNAs which are differentially methylated in BCVY-metEPICVal samples. Percentage of methylation differences for statistically significant CpG sites from BCVY-BCO comparison according to location of the CpG relative to the island (**A**) and to the UCSC gene region feature category and regulatory elements (**B**). Red bars represent hypermethylation in BCVY and green bars represents hypomethylation. *Statistically significant *p-values* (*p* < 0.001). N/S: north/south; upstream or downstream to the CpG island.

In terms of CpG context, significant methylation differences for miRNA CpG probes were localized in islands (*p-value* = 5.29 × 10^−7^) and regions away from them called open sea (*p-value* = 4.73 × 10^−5^). Specifically, significant CpG probes localized in island regions were mainly hypomethylated in BCVY and those localized in open sea sites were hypermethylated in this age group (Fig. 2A). We analysed the functional localization of differentially methylated miRNA probes and the most important differences were identified in DNasa I hypersensitive sites (DHSs) (*p-value* = 4 × 10^−3^), which were generally hypermethylated in BCVY. Although not significant, transcription factor binding sites, promoters and gene body regions presented methylation differences of more than 4% between BCVY and BCO (Fig. 2B).

### Methylation Validation in two populations

Both data validation sample sets were analysed using Infinium HumanMethylation450 BeadChip (HM450K), which includes around 50% of probes included in the EPICarray. Of 193 significant differentially methylated miRNA probes identified in the metEPICVal sample set, only 85 were shared with the HM450K array and could be analysed in the validation study, whereas the 108 remaining probes were exclusively for the EPICarray and could not be included in the validation study. We verified the methylation differences for 17 CpG sites between BCVY and BCO in TCGA data (*p-value* < 0.05 and methylation differences ±0.1). Combined data showed 30 differentially methylated miRNA CpG probes among the 85 analysed in the validation study. Finally, we were able to validate a total of 10 methylation probes regulating miRNAs that were significant in both validation data sets, and all of them were hypomethylated in BCVY compared to older patients (Table 1).

**Table 1:** Table of CpG probes regulators of miRNA genes that were significantly differentially methylated in BCVY compared with BCO samples in the validation study using TCGA and the combined study data sets.

Validated CpG probes	miRNA gene	TCGA		Combined study	
		% Methylation differences (BCVY - BCO)	*p-value*	% Methylation differences (BCVY - BCO)	*p-value*
cg04735310	MIR196A1	−11,13	4,13E-50	−14,77	5,57E-04
cg07234865	MIR9-1	−12,79	1,8E-45	−14,30	6,72E-05
cg08737296	MIR124-3	−13,29	7,3E-23	−15,73	3,84E-03
cg07792478	MIR124-2	−14,31	3,92E-21	−10,07	1,03E-02
cg05474726	MIR124-2	−11,78	2,66E-20	−14,27	4,21E-03
cg22333214	MIR137	−15,42	2,74E-20	−11,66	1,41E-02
cg04947764	MIR184	−10,56	5,67E-18	−17,85	4,73E-04
cg25147193	MIR181C	−10,32	5,61E-17	−13,96	8,03E-03
cg16407471	MIR129-2	−10,07	5,6E-12	−13,04	2,84E-02
cg00210994	MIR548G	−10,13	2,11E-07	−14,67	2,29E-02

This table includes a list of 10 significant CpG probes and the corresponding miRNA that were regulated by them.

### Meta-analysis expression of miRNAs regulated by significant CpG probes

To elucidate whether differentially methylated CpG probes affected miRNA gene expression, we analysed expression of affected miRNA in a meta-analysis using three gene expression data sets (TCGA, Clariom D and Peña-Chilet *et al*.^23^). We analysed expression in miRNAs regulated by the 10 validated CpG sites. In addition, we included miRNAs regulated by those 108 CpG probes that were differently methylated in BCVY-BCO comparison and exclusive of the EPICarray, which were therefore excluded from the methylation validation study in the HM450K data sets. Finally, we had a total of 118 CpG probes that regulated 62 unique miRNAs. Gene expression for unique miRNAs was evaluated by t-test in the three data sets separately. Only 10 miRNAs were included in the three studies and could therefore be evaluated in the meta-analysis (Table 2), and 4 of them were significantly de-regulated with adjusted *p-value* < 0.05 (miR-9-1, miR-184, miR-551b and miR-196a-1).

**Table 2:** MiRNA expression results from meta-analysis.

miRNAs	*p-value*	*p-value* FDR adjusted	Gene expression differences (BCVY –BCO)
hsa-mir-196a-1	3,92E-47	3,92E-46*	−0,28
hsa-mir-184	1,31E-34	6,55E-34*	0,26
hsa-mir-9-1	6,12E-03	2,04E-02*	−0,13
hsa-mir-551b	1,45E-02	3,62E-02*	−0,38
hsa-mir-548n	1,46E-01	2,92E-01	−0,06
hsa-mir-383	3,49E-01	5,82E-01	0,28
hsa-mir-548f-1	5,74E-01	8,21E-01	−0,28
hsa-mir-490	8,05E-01	9,65E-01	0,14
hsa-mir-181c	9,46E-01	9,65E-01	0,11
hsa-mir-662	9,65E-01	9,65E-01	−0,05

Table shows miRNA expression values from the meta-analysis of the 10 miRNAs common to the three studies: TCGA, Clariom D and Peña-Chilet *et al*. study^23^.

^*^Statistically significant *p-values* adjusted by Benjamini-Hochberg False Discovery Rate procedure (FDR *p-value*s < 0.05) and gene expression differences ±0.1.

We also analysed whether we observed different expression in miRNAs regulated by differentially methylated regions. For this purpose, we selected unique miRNAs for each significant region obtained (CpG Island, open sea and DHSs) and their expression was analysed in a meta-analysis using the three previously mentioned data sets. We identified 14 differentially methylated miRNAs regulated by CpG islands regions and 3 of them were also significantly differently expressed between BCVY and BCO (miR-181c, miR-196a-1 y miR-212). Significant open sea regions regulated 55 miRNA genes and miR-184, miR-211 and miR-383 were significantly deregulated in the meta-analysis. Finally, DHSs significant in methylation analysis regulated 38 miRNAs and 5 of them presented different expression (miR-196a-1, miR-184, miR-345, miR-212 and miR-211). Results are shown in Table 3. Interestingly, we found some miRNAs that were regulated by more than one significant CpG categories and were deregulated in BCVY compared with older patients.

**Table 3:** MiRNA expression results from meta-analysis by category regions.

miRNAs	*p-value*	*p-value FDR adjusted	% gene expression differences (BCVY - BCO)	% mean methylation differences (BCVY - BCO)
**Island probes**				
hsa-mir-181c	1,29E-41	5,15E-41	−26,7	−12,0
hsa-mir-196a-1	1,39E-05	2,79E-05	−5,5	−11,3
hsa-mir-212	2,19E-02	2,92E-02	−12,8	−17,2
**OpenSea probes**				
hsa-mir-184	9,82E-41	9,82E-40	25,1	−18,5
hsa-mir-211	1,28E-03	6,41E-03	−19,9	17,6
hsa-mir-383	3,39E-03	1,13E-02	−38,0	12,1
**DNase I hypersensitive regions**				
hsa-mir-196a-1	3,79E-40	4,17E-39	−26,8	−12,0
hsa-mir-184	2,10E-36	1,16E-35	26,2	−18,5
hsa-mir-345	2,52E-21	9,25E-21	4	11,0
hsa-mir-212	5,84E-05	1,61E-04	−7,2	−11,3
hsa-mir-211	2,34E-04	5,14E-04	−20,2	17.6

Expression of miRNAs that were regulated by CpG probes localized at the most different methylated regions between BCVY and BCO were analysed separately in the meta-analysis. The table shows miRNAs that were differentially methylated and differently expressed in BCVY vs. BCO taking into account category regions analysed. *P-value* and adjusted *p-value* by Benjamini & Hochberg were calculated and differences in gene expression and methylation between BCVY and BCO are indicated in the table.

*p-values were adjusted by Benjamini-Hochberg False Discovery Rate procedure.

### Pathway enrichment analysis of significant miRNA

**Figure 3:**
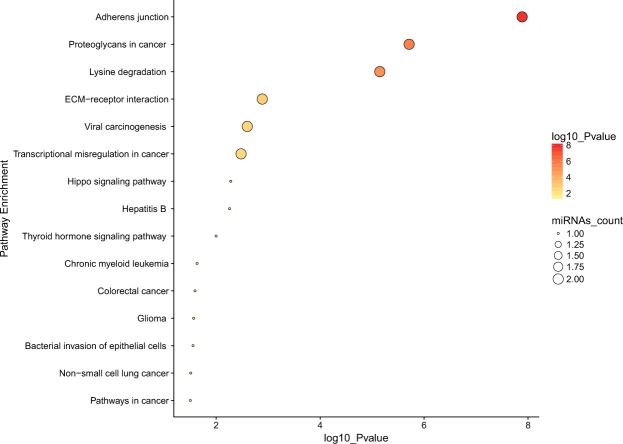
Pathway enrichment analysis results obtained by DIANA mirpath. Plot represents the main pathways in which miRNA regulated by significant CpG probes in BCVY were involved. Dot colour indicates *p-values* and miRNA count indicates the number of miRNAs involved in the represented pathways.

MiRNA genes obtained in the meta-analysis that were regulated by different methylated regions, were related to different pathways reported in Fig. 3. The miRNAs miR-9-1 and miR-196a-1 were involved in pathways related to adherent junctions, proteoglycans involved in cancer or transcriptional misregulation in cancer, among others. MiR-196a-1 took part in multiple pathways, the vast majority of them implicated in other cancer types.

MiRNAs regulated by CpG probes localized in island regions were involved in multiple pathways related to cancer (transcriptional misregulation in cancer, prostate, lung and endometrial cancer pathway, among others), adherent junctions, oestrogen signalling pathways, etc. For deregulated miRNAs in BCVY whose methylation was regulated by open sea regions, we identified pathways related to cancer as previously observed for island miRNAs and, moreover, they were implicated in tumour-necrosis signalling pathways, extracellular matrix-receptor interaction and prolactin signalling pathways. Finally, for DHSs we obtained, again, most of the pathways previously observed in island regions. Pathway enrichment results by category region are reported in Supplementary Fig. 1.

### qRT-PCR Validation of miRNA expression

**Figure 4:**
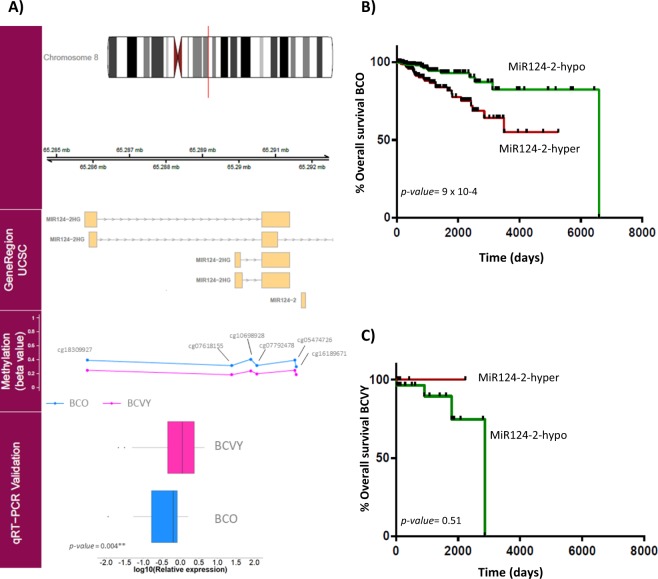
MiR124-2 expression validation by qRT-PCR and overall survival studies. First track represents the chromosome position of the different methylated region. Gene Region track represents genome position of the significant CpG regions obtained. Methylation track shows the β-values for the significant CpG probes regulating miR-124-2. Validation results for miR-124-2 expression using qRT-PCR are plotted in the qRT-PCR Validation track. Boxplots represent the relative mean expression for BCO and BCVY samples, *p-values* were obtained by Wilcoxon rank sum test (**A**); Representation of overall survival curves for miR-124-2 according to their methylation status in BCO (**B**) and BCVY (**C**). Green curves represent miRNA hypomethylation and red colour line hypermethylation. *P-values* were obtained by multivariate Cox analysis adjusting for oestrogen receptor status and molecular subtype.

Validation by quantitative real-time PCR (qRT-PCR) was performed on an independent set of samples with similar characteristics to those used in the metEPICVal study. We analysed the expression of the four significant miRNAs from the meta-analysis (miR-9-1, miR-184, miR-551b and miR-196a-1) and additionally, we analysed the expression of the precursor miR-124-2 that was hypomethylated in both methylation validation data sets but could not be evaluated in the expression study because it was not included in all data sets used in the meta-analysis expression. MiR-124-2 was significantly overexpressed in BCVY samples (*p-value* < 0.05) compared with older patients and this result correlated with the significant hypomethylation detected in BCVY (Fig. 4A). MiR-9-1 (*p-value* = 0.07), miR-196a-1 (*p-value* = 0.36) and miR-184 (*p-value* = 0.7) were overexpressed in BCVY, which are in agreement with the hypomethylation detected and miR-551b was repressed in BCVY (*p-value* = 0.07) that was consistent with the hypermethylation found. However, the differences did not reach statistical significance (Supplementary Fig. 2).

### Clinical importance of miRNA methylation as an independent prognostic factor for relapse-free survival and overall survival in BCVY

To test the hypothesis of miRNA-methylation association to relapse-free survival (RFS) and overall survival (OS), we investigated the metEPICVal samples and methylation samples from TCGA that present follow-up data. We next performed a univariate cox regression study to determine whether hypo- or hypermethylation of significant miRNAs were correlated with underlying clinical conditions in BCVY and BCO. Whereas no significance was reached in the RFS analysis, miRNA hypomethylation was related with higher relapse risk in both BC groups (BCO and BCVY) for all miRNAs analysed with the exception of miR-184, in which hypomethylation reduces the risk of relapse in BCVY, contrary to the results for BCO (Supplementary Fig. 3). For OS studies, univariate cox analysis showed that miR-124-2 hypermethylation was significantly related with reducing survival in BCO (*p-value* = 9 × 10^−4^) (Fig. 4B). In order to evaluate whether other clinical factors were impacting in the survival in BCO patients apart from miR-124-2 methylation, we performed a multivariable Cox regression analysis including data from oestrogen receptor status and BC molecular subtypes. Multivariate analysis shown a significant relation with miR-124-2-hypermethylation in BCO and poor survival (*p-value* 1.02 × 10^−3^, hazard ratio HR = 3.23). However, oestrogen receptor status and molecular subtype were not contributing to survival in BCO patients. Although no significant results were obtained in univariate cox study among BCVY survival and miR-124-2 methylation, we observed a contrary effect on them, in which poorer survival was related with miR-124-2 hypomethylation (Fig. 4C). BCVY sample size is limited compared with older patients and further studies with larger dataset should be addressed to evaluate mir-124-2-hypomethylation and prognosis in this group. Additionally, OS analysis demonstrated a good prognostic factor for miR-184 hypomethylation in both BCVY and BCO groups, and a reduced survival rate for miR-196a-1-hypomethylation in BCVY: however, these results did not reach statistical significance (Supplementary Fig. 4).

## Discussion

Several miRNAs have been involved in cancer pathogenesis^24^. Accordingly, altered miRNA expression profiles have been found in every type of human cancer including colon, brain, lung and breast cancer^25,26^ suggesting miRNAs as possible biomarkers for early cancer detection. Furthermore, miRNAs not only function as part of the epigenetic machinery, but are also epigenetically modified by DNA methylation themselves. DNA methylation plays a key role in silencing numerous cancer-related genes affecting several processes, and there is considerable evidence supporting the idea that DNA methylation is actively involved in the dysregulation of miRNAs in cancer^27^.

Breast cancer affecting young women has been previously associated with a more aggressive type of tumours than those diagnose in older women which could be responsible for the poor prognosis that characterizes these tumours. Previous group studies have shown differing methylation signatures for breast cancer in young women than in older patients^28^. Although the mechanism underlying miRNA dysregulation in cancer is not yet fully understood, recent studies have shown that epigenetic mechanisms play an important role in regulating miRNA expression^19^.

In this study, we analysed the differences in DNA methylation of miRNA encoding genes between breast cancer samples in young and old women. Results from the methylation study in metEPICVal samples performed in the Illumina Infinium MethylationEPIC BeadChip showed 193 CpG sites that were significantly differentially methylated between groups. Most highlighted methylation differences were localized in open sea and island CpG regions. Specifically, open sea sites were hypermethylated and islands and regions near them were hypomethylated in BCVY. These results are in agreement with our previous global methylation study^28^ that found hypomethylation localized in CpG island regions and hypermethylation in open sea and regions away from the islands. Relative to that, it has been seen that several gene-encoding miRNAs are more frequently hypermethylated and consequently repressed in regions away from the islands than the CpG island itself^27^, agreeing with our observations.

In terms of regulatory localization, most significantly hypomethylated probes in BCVY were located on DHSs. These regions of chromatin are sensitive to cleavage by DNase I enzyme, and chromatin has lost its condensed structure, making the DNA more accessible. This remodelled state is essential to increased transcriptional activity. DHSs therefore tended to be enriched on highly expressed genes throughout whole gene regions while not showing significant changes for low and silently expressed genes. Also, DHSs are enriched in regions away from CpG islands, suggesting preference to act within active chromatin domains that present low density CpG islands^29^. In contrast to the previously mentioned hypermethylation observed in regions away from islands, DHS regions present hypomethylation in BCVY, contributing to the more accessible DNA and consequently upregulating their expression.

In order to validate the results found in the metEPICVal sample, we used data from two previous methylation analysis (TCGA methylation and combined data) performed with HM450K array, with the limitation that we could only analyse 85 CpG probes that were shared between EPICarray and HM450K. The methylation validation study identified 10 CpG significant probes, all of them hypomethylated in young women with breast cancer. These results are in agreement with HM450K array content, given that the number of CpG islands probes is higher than open sea probes, the latter poorly presented in the HM450K array. Based on this we were able to validate some of the hypomethylation probes for BCVY localized in islands regions or near them, using the HM450K validation data sets. However, hypomethylation was concentrated mainly in open sea regions, which were underrepresented in HM450K data sets and could not be validated.

Advances in microarray and sequencing technologies have enabled comprehensive analysis of the epigenome and miRNA expression in cancer cells, which has led to the identification of miRNA which are frequent targets of aberrant DNA methylation in cancer^30^. In the present study we were able to validate significant methylation differences in a set of differentially methylated miRNAs in cancer such as: miR-181c, miR-129-2, miR-196a-1, miR-137, miR-9-1 and miR-124, of which methylation differences were observed in miR-124-2 and miR-124-3. Unfortunately, some differentially methylated miRNA could not be analysed in the expression meta-analysis because they were not included in the three data sets analysed.

We explored the expression of miRNAs regulated by differently methylated probes in BCVY taking into account their genomic/functional category. Some significant miRNAs were regulated by probes situated in more than one genomic/functional category. Differentially deregulated MiR-196a-1 and miR-212 in BCVY were in turn regulated by differently methylated regions located in islands and DHS sites. Another similar case is miR-211 and miR-184, which were regulated by probes situated in open sea and DHS sites and were deregulated in BCVY. These results suggest an important regulatory role for DNA methylation in the miRNA-encoding genes which are significantly deregulated in BCVY vs. BCO in CpG from different regions.

Next, meta-analysis of expression in miRNAs regulated by validated regulatory CpG probes and new significant probes included in the EPICarray, revealed 4 miRNAs (miR-9-1, miR-184, miR-551b and miR-196a-1) that were significantly differently expressed and methylated in BCVY. Although miR-124-2 expression could not be evaluated in the meta-analysis expression study, its hypomethylation was validated in both methylation data sets and qRT-PCR revealed significant miR-124-2 upregulation in BCVY. However, none of the four miRNAs identified (miR-9-1, miR-184, miR-551b and miR-196a-1) could be validated by qRT-PCR.

Enrichment analysis showed that the significant differentially methylated miRNAs have functions linked to cancer. Most of them were implicated in pathways related with adherent junctions, important in cancer initiation and progression. Furthermore, some miRNAs were involved in extracellular matrix organization through proteoglycans and extracellular matrix-receptor interaction.

MiRNA-methylation has been extensively investigated as a prognostic factor, and survival analysis performed with our data and TCGA revealed that hypermethylation of miR-124-2 was significantly associated with poorer OS in BCO, in contrast to BCVY, in which miR-124-2-hypomethylation presented the lowest survival rates. These results suggest that hypomethylation of miR-124-2 may be a potential prognostic risk factor specific to BCVY. Although no significant results were reached for the rest miRNAs, our study suggested that miR-184-hypomethylation could be a good prognostic factor in breast cancer patients, in which methylation loss increases survival and reduces relapse rates. Conversely, miR-9-1 and miR-196a-1 hypomethylation were associated with reduced survival and higher relapse rates.

Within the human genome, three independent loci (miR-124-1, miR-124-2 and miR-124-3) encode the identical mature miR-124, and all are associated with CpG islands, which have been described as targets of hypermethylation in colon, stomach, liver, leukaemia and cervix cancers^31^. MiR-124 exerts a tumour suppressor effect by targeting cyclin-dependent kinase 6, and epigenetic silencing of miR-124 leads to CDK6 activation and Rb phosphorylation^31,32^. Interestingly, our results show a hypomethylation of miR-124-2 gene in BCVY compared with older patients and a higher miRNA expression. Additionally, the miR-124-2 has been found to be the most abundant miRNA expressed in neuronal cells and their differentiation. Neuronal elements have been described that are related to cancer microenvironment promoting cell growth, although the mechanisms mediating neuronal influences on cancer growth and progression are likely incompletely understood^33^. Previous studies in BCVY have shown that most of the pathways deregulated in this group were related to neuronal-system processes (Oltra SS *et al*., unpublished data). However, more work is needed to gain insight into the miRNA-124-2-hypomethylation role in breast cancer affecting young women and its potential role as a biomarker of poor survival in BCVY.

Several limitations of the present study need to be acknowledged. Methylation validation analysis has been possible for only 85 of the total 193 significant CpG probes due to the lack of methylation studies performed with the EPICarray. The TCGA dataset only provides data on methylation studies performed with HM450K or previous arrays. In the case of miRNA expression meta-analysis, the three datasets employed for the study do not include all significantly differentially methylated miRNAs. Thus, the miRNAs that were significantly deregulated in some but not all of the three studies were not included in the meta-analysis and information about expression status could not be evaluated; despite obtaining the methylation status of significant CpG regions for BCVY, we could not translate them into differences in gene expression.

The present work is the first to analyse methylation of miRNA-encoding genes using the EPICarray in breast cancer samples from young women, and the association with miRNA expression. Our main finding is the relationship of miR-124-hypomethylation with significantly better survival rates for BCO as opposed to the worse prognosis observed in BCVY, identifying it as a potential specific survival biomarker in this group.

## Material and Methods

### Methylation and gene expression samples

#### Illumina Infinium MethylationEPIC BeadChip samples (metEPICVal samples)

Samples included in the Illumina Infinium MethylationEPIC BeadChip experiment were archived in formalin fixed paraffin-embedded and all were stored at the Pathology Department at the Hospital Clínico Universitario, Valencia, Spain. We used 26 samples of BCVY and 15 samples from older women with breast cancer (BCO). Additionally, we have 5 samples from healthy female donors (3 from young and 2 from old women). The clinical characteristics of the patients whose samples were included in the study are shown in Supplementary Table 2. Informed consent was obtained from all subjects and the study was approved by the Hospital Clinico Ethical Committee and all research was performed in accordance with relevant guidelines.

### DNA methylation quantification and normalization

Samples were extracted using a commercial kit (QIAamp DNA FFPE Tissue Kit, Qiagen, Hilden, Germany) following the manufacturer’s instructions. DNA samples were quantified (Quant-iT PicoGreen dsDNA Assay, Life Technologies, CA, USA), and assessed for purity by NanoDrop (Thermo Scientific, MA, USA). Samples were checked for suitability for FFPE restoration, as indicated in the Infinium HD FFPE QC Assay (Illumina Inc.); 500 ng of FFPE DNA were bisulphite converted using the EZ-96 DNA Methylation-Gold™ kit (Zymo Research Corp., CA, USA). Bisulfite-converted DNA from FFPE samples was restored following instructions from Infinium assay. Next, DNA was hybridised to the Illumina Infinium MethylationEPIC BeadChip array following the Illumina protocol. BeadChips were washed and scanned using the Illumina HiScan SQ scanner, and the intensities were extracted from GenomeStudio (v.2011.1) and Methylation module (1.9.0) software which normalises within-sample data. Raw microarray data and processed normalized data are available from Gene Expression Omnibus (GEO) (GSE100850). Background subtraction and colour correction for the dye bias seen in Infinium II probes, as well as removal of bad quality samples were performed using *minfi* package implemented in R Bioconductor^34^. The β-values (indicating methylation level at each CpG) were calculated using *minfi* package. A total of 30,271 single nucleotide polymorphism loci were excluded from subsequent analysis. Probes that were not detected in more than 20% of the samples were excluded from the analyses. We selected samples with >98% of probes detected. Additionally, we removed cross reactive probes for the methylation EPICarray described by McCartney *et al*.^35^. After pre-processing, we analysed 793,483 CpG sites in 21 samples from BCVY and 13 from BCO.

### Analysis of miRNA-associated probes

Our study focused on probes associated with gene encoding for miRNAs. According to the information available in the Illumina annotation file, EPICarray platform includes 9,961 probes that are linked to miRNAs. After the pre-processing described in previous section, we obtained a set of 6,567 CpG probes associated with 1,264 unique miRNAs which were used in the present study.

Analysis of probe distribution by gene-coding miRNAs revealed probes associated with one miRNA and probes associated with multiple miRNAs; the maximum number was for probes regulating 7 miRNAs. Information about correspondence between CpG probes and miRNAs regulated by them are included in Supplementary Table 3. Additionally, miRNAs may be regulated by unique or multiple probes (1–293). Probe count by miRNA is summarized in Supplementary Table 4.

### Statistical analysis

**Figure 5:**
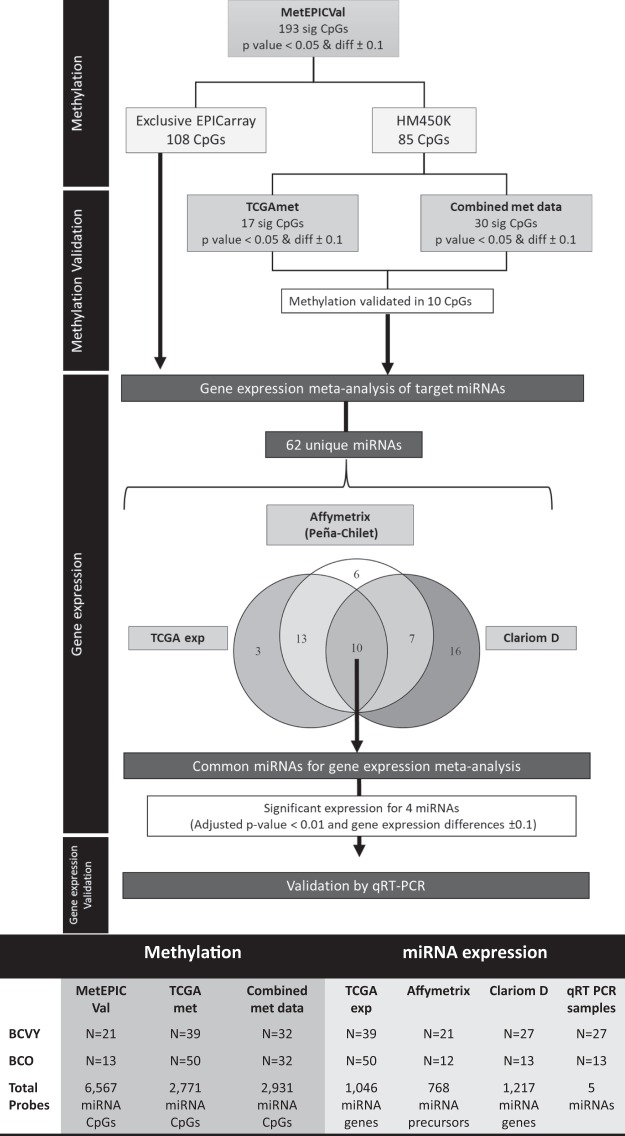
Workflow diagram of the procedure. The diagram shows the sample set analysed and the significant probes obtained in each step. The table includes the sample and probe size for each sample set. *diff: methylation differences between BCVY minus BCO; sig: significant.

We analysed methylation differences using the Wilcoxon Rank Sum test. CpG sites with both *p-values* < 0.05 and a minimum change of ±0.1 in β-values between BCVY and BCO were considered significant. Additionally, we performed a GLM analysis to assess whether specific methylation differences observed between BCVY and BCO were independent of molecular subtype and ER status. The study procedure diagram is included in Fig. 5.

MiRNA probes were classified into different categories according to their description in the Illumina manifest file, depending on function (promoter, gene body, TSS or UTRs), relation to the CpG context (N/S shore, N/S shelf and island), enhancer regions provided by ENCODE and FANTOM5 projects, and ENCODE regulatory elements (CpG sites in transcription factor binding sites [TFBS], open chromatin regions and DHSs). Methylation differences by categories in the significant probe set were assessed by Welch’s t-test and *p-values* < 0.001 were considered to be statistically significant.

Univariate Cox analysis was used to analyse the effect of clinical variables and miRNA methylation on patients’ relapse-free survival and overall survival. Multivariate Cox method was performed to adjust for clinical variables such as oestrogen receptor status and molecular subtype that could be influencing in relapse and/or survival.

### Methylation Validation in two populations

We performed a combined study using methylation data from Flower *et al*.^36^ analysed by Infinium HumanMethylation450 BeadChip and data from the metEPICVal study previously described. First, we analysed HM450K array samples according to the methods described in the data pre-processing section. We retained methylation levels for 405,068 probes present in both metEPICVal and Flower *et al*.’s^36^ HM450K study in a total of 64 samples (32 BCO samples, 32 from BCVY) that were used in the validation study.

Additionally, we used methylation data from independent breast tissue samples available from TCGA, that includes data for 485,577 probes in 720 BCO and 27 BCVY samples. Gene expression study for TCGA data was done with 50 permutations, and 50 samples were randomly selected and balanced by subtype.

Both studies were performed using the Illumina HM450K array, which includes only half of the probes present in the EPICarray. To validate our results from the BCVY-BCO study we selected differentially methylated miRNA probes that were included in HM450K and a Wilcoxon Rank Sum test was performed between BCVY and BCO tumour samples.

### miRNA expression meta-analysis

We used our own gene expression data from 42 breast cancer patients (13 from BCO and 27 from BCVY) analysed by Clariom D array (Applied Biosystems by Life Technologies, Carlsbad, California, USA)^37^. Total RNA was isolated using RecoverAll Total Nucleic Acid Isolation Kit (Applied Biosystems by Life Technologies, Carlsbad, California, USA) following the manufacturer’s protocol. RNA concentration was measured by Qubit® 3.0 (InvitrogenTM by Termo Fisher Scientific, Waltham, MA, USA) using the Qubit® RNA HS Assay Kit (Molecular Probes® by Life Technologies Carlsbad, California, USA). RNA quality was assessed by qRT-PCR; 25 ng of RNA were hybridized in the Clariom D array. Raw files (*.CEL) were normalized by Robust Multichip Average method using from the R Bioconductor *affy* package.

Expression of miRNAs regulated by differentially methylated probes were analysed using data from Clariom D samples. This study includes expression for 1,217 miRNA precursors. Additionally, in the meta-analysis we included previous miRNA expression published data from Peña-Chilet *et al*.^23^, which were analysed by GeneChip® miRNA 2.0 Array (Affymetrix, Santa Clara,CA, USA) with GEO accession number GSE48088. This data set includes 21 BCVY and 12 BCO samples. Additionally, we downloaded gene expression from TCGA and we used data from 39 BCVY and 50 BCO samples, randomly selected and balanced by subtype.

We performed a meta-analysis study to combine *p-values* from three studies by Fisher method using the *metap* R package; *p-values* were adjusted by Benjamini-Hochberg FDR procedure^38^; genes with FDR <0.01 and gene expression differences ±0.1 were considered statistically significant.

### Pathway enrichment analysis

DIANA miRPath pathway enrichment analysis was used to gain insight into global molecular networks and canonical pathways related to differentially expressed miRNAs (http://diana.imis.athena-innovation.gr/DianaTools/index.php?r=mirpath/index). The software performs an enrichment analysis of multiple miRNA target genes comparing each set of miRNA targets to all known KEGG pathways. Pathways showing a FDR *p-value* < 0.05 were considered significantly enriched between classes under comparison.

### miRNA expression validation by quantitative real-time PCR

Validation of significant miRNAs was performed in a different sample set composed of 27 samples from BCVY and 13 from BCO using qRT-PCR (clinical characteristics in Supplementary Table 2) using Advance TaqMan Gene Expression Assays and TaqMan microRNA Assays (Applied Biosystems by Life Technologies, Carlsbad, California, USA) and normalising to hsa-mir-21 or RNU43 and RNU6B expression. The data were managed using the QuantiStudio Design & Analysis software (v1.4). Relative expression was calculated by using the comparative Ct method and obtaining the fold-change value (ΔΔCt). The Wilcoxon rank sum test was used for non-parametric samples, *p-value* < 0.05 were considered statistically significant.

## Electronic supplementary material

Supplementary Figures

Supplementary Table 1 and 2

Supplementary Table 3

Supplementary Table 4

## References

[1] Ferlay J (2015). Cancer incidence and mortality worldwide: sources, methods and major patterns in GLOBOCAN 2012. International journal of cancer.

[2] Azim HA (2012). Elucidating prognosis and biology of breast cancer arising in young women using gene expression profiling. Clinical cancer research: an official journal of the American Association for Cancer Research.

[3] Fredholm H (2009). Breast cancer in young women: poor survival despite intensive treatment. PloS one.

[4] Fredholm H (2016). Long-term outcome in young women with breast cancer: a population-based study. Breast cancer research and treatment.

[5] Gnerlich JL (2009). Elevated breast cancer mortality in women younger than age 40 years compared with older women is attributed to poorer survival in early-stage disease. Journal of the American College of Surgeons.

[6] Kroman N (2000). Factors influencing the effect of age on prognosis in breast cancer: population based study. Bmj.

[7] Partridge AH (2016). Subtype-Dependent Relationship Between Young Age at Diagnosis and Breast Cancer Survival. Journal of clinical oncology: official journal of the American Society of Clinical Oncology.

[8] Anders CK (2008). Young age at diagnosis correlates with worse prognosis and defines a subset of breast cancers with shared patterns of gene expression. Journal of clinical oncology: official journal of the American Society of Clinical Oncology.

[9] Bharat A, Aft RL, Gao F, Margenthaler JA (2009). Patient and tumor characteristics associated with increased mortality in young women (<or = 40 years) with breast cancer. Journal of surgical oncology.

[10] Colleoni M (2002). Very young women (<35 years) with operable breast cancer: features of disease at presentation. Annals of oncology: official journal of the European Society for. Medical Oncology.

[11] Sabiani L (2016). Breast cancer in young women: Pathologic features and molecular phenotype. Breast.

[12] Swain SM, Nunes R, Yoshizawa C, Rothney M, Sing AP (2015). Quantitative Gene Expression by Recurrence Score in ER-Positive Breast Cancer, by Age. Advances in therapy.

[13] Feinberg AP, Vogelstein B (1983). Hypomethylation of ras oncogenes in primary human cancers. Biochemical and biophysical research communications.

[14] Eden A, Gaudet F, Waghmare A, Jaenisch R (2003). Chromosomal instability and tumors promoted by DNA hypomethylation. Science.

[15] Gaudet F (2003). Induction of tumors in mice by genomic hypomethylation. Science.

[16] Narayan A (1998). Hypomethylation of pericentromeric DNA in breast adenocarcinomas. International journal of cancer.

[17] Herman JG, Baylin SB (2003). Gene silencing in cancer in association with promoter hypermethylation. The New England journal of medicine.

[18] Bannister AJ, Kouzarides T (2011). Regulation of chromatin by histone modifications. Cell research.

[19] Piletic K, Kunej T (2016). MicroRNA epigenetic signatures in human disease. Archives of toxicology.

[20] http://support.illumina.com. *Infinium MethylationEPIC BeadChip Kit Support*.

[21] Consortium EP (2012). An integrated encyclopedia of DNA elements in the human genome. Nature.

[22] Lizio M (2015). Gateways to the FANTOM5 promoter level mammalian expression atlas. Genome biology.

[23] Pena-Chilet M (2014). MicroRNA profile in very young women with breast cancer. BMC cancer.

[24] Calin GA (2002). Frequent deletions and down-regulation of micro- RNA genes miR15 and miR16 at 13q14 in chronic lymphocytic leukemia. Proceedings of the National Academy of Sciences of the United States of America.

[25] Baffa R (2009). MicroRNA expression profiling of human metastatic cancers identifies cancer gene targets. The Journal of pathology.

[26] Di Leva G, Briskin D, Croce CM (2012). MicroRNA in cancer: new hopes for antineoplastic chemotherapy. Upsala journal of medical sciences.

[27] Suzuki H, Maruyama R, Yamamoto E, Kai M (2012). DNA methylation and microRNA dysregulation in cancer. Molecular oncology.

[28] Oltra, S. S. *et al*. Increased DNA methylation age acceleration in breast cancer tumours from very young women. *Breast cancer research and treatment In revision* (2018).

[29] Cockerill PN (2011). Structure and function of active chromatin and DNase I hypersensitive sites. The FEBS journal.

[30] Kaur S, Lotsari-Salomaa JE, Seppanen-Kaijansinkko R, Peltomaki P (2016). MicroRNA Methylation in Colorectal Cancer. Advances in experimental medicine and biology.

[31] Lujambio A (2007). Genetic unmasking of an epigenetically silenced microRNA in human cancer cells. Cancer research.

[32] Agirre X (2009). Epigenetic silencing of the tumor suppressor microRNA Hsa-miR-124a regulates CDK6 expression and confers a poor prognosis in acute lymphoblastic leukemia. Cancer research.

[33] Venkatesh Humsa, Monje Michelle (2017). Neuronal Activity in Ontogeny and Oncology. Trends in Cancer.

[34] Aryee MJ (2014). Minfi: a flexible and comprehensive Bioconductor package for the analysis of Infinium DNA methylation microarrays. Bioinformatics.

[35] McCartney DL (2016). Identification of polymorphic and off-target probe binding sites on the Illumina Infinium MethylationEPIC BeadChip. Genomics data.

[36] Flower KJ (2015). DNA methylation profiling to assess pathogenicity of BRCA1 unclassified variants in breast cancer. Epigenetics.

[37] Vidal-Tomas V (2017). Global transcriptome deregulation of Breast Cancer in Very Young Women samples. Annals of Oncology.

[38] Benjamini Y, Y. H (1995). Controlling the false discovery rate: a practical and powerful approach to multiple testing. Journal of the Royal Statistical Society.

