# Supplementary material for: Methylation deregulation of miRNA promoters identifies miR124-2 as a survival biomarker in Breast Cancer in very young women
Source: Sci Rep. 2018 Sep 26;8:14373. doi: 10.1038/s41598-018-32393-3 (PMC6158237; doi:10.1038/s41598-018-32393-3)

Methylation deregulation of miRNA promoters identifies miR124-2 as a survival biomarker in Breast Cancer in very young women

Sara S. Oltra[1], Maria Peña-Chilet[1], Victoria Vidal-Tomas [1], Kirsty Flower[2], María Teresa Martinez[1], Elisa Alonso[3], Octavio Burgues[3], Ana Lluch[1]¥, James M. Flanagan[2], and Gloria Ribas[1]¥*

Supplementary Figures

Supplementary Figure 1: Pathway enrichment analysis results for miRNAs deregulated by localization of significant categories obtained: (**A**) DNase I hypersensitive sites: (**B**) Islands; (**C**) Open sea. Dot colour indicates p-values (orange for most significant pathways and yellow for lower p-values) and miRNA count indicates the number of miRNAs involved in the represented pathways.

Supplementary Figure 2: qRT-PCR expression validation results. Boxplots represent the sample distribution with the mean for BCO and BCVY patients. Expression was quantified using the qRT-PCR technic and calculated by $\Delta\Delta$Ct method. Y axis represents logarithmic transformation of the relative expression. Differences by miRNA between BCVY and BCO were analysed by Wilcoxon rank sum test: (**A**) miR9-1; (**B**) miR196a-1; (**C**) miR184; (**D**) miR551b.

Supplementary Figure 3: Representation of relapse-free survival curves for significantly differentially methylated miRNAs according to their methylation status. The first column curves represent RFS in BCO patients and the second RFS in BCVY. X axis represents the follow-up time by days and Y axis indicates the percentage of relapse-free survival. Green curves represent miRNA hypomethylation and red line hypermethylation. P-values obtained by univariate cox analysis.

Supplementary Figure 4: Representation of overall survival curves for significantly differentially methylated miRNAs according to their methylation status. The first column curves represent OS in BCO patients and the second OS in BCVY. X axis represents the follow-up time by days and Y axis indicates the percentage of overall survival. Green curves represent miRNA hypomethylation and red colour line hypermethylation. P-values obtained by univariate cox analysis.

Supplementary Figure 1

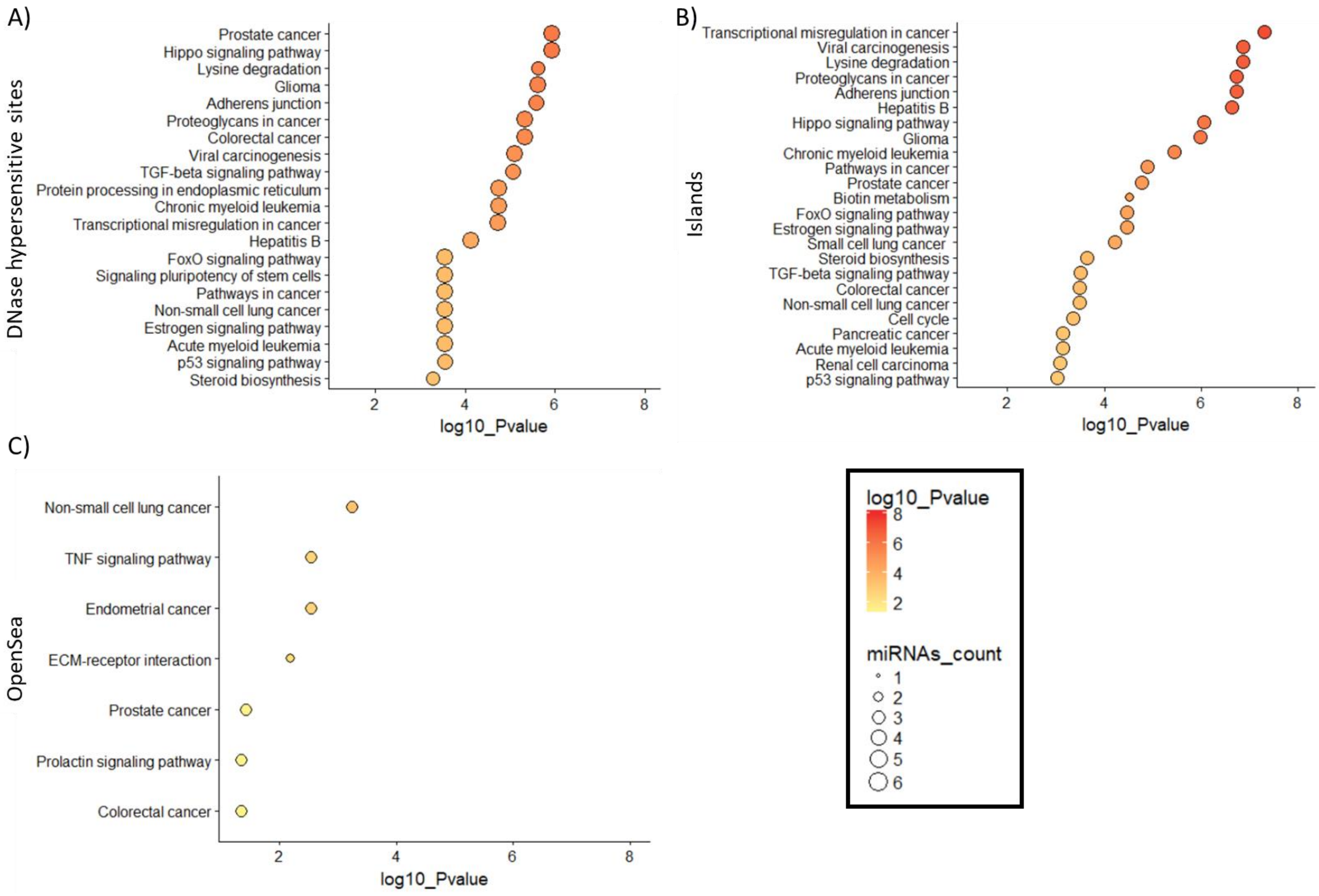

Supplementary Figure 2

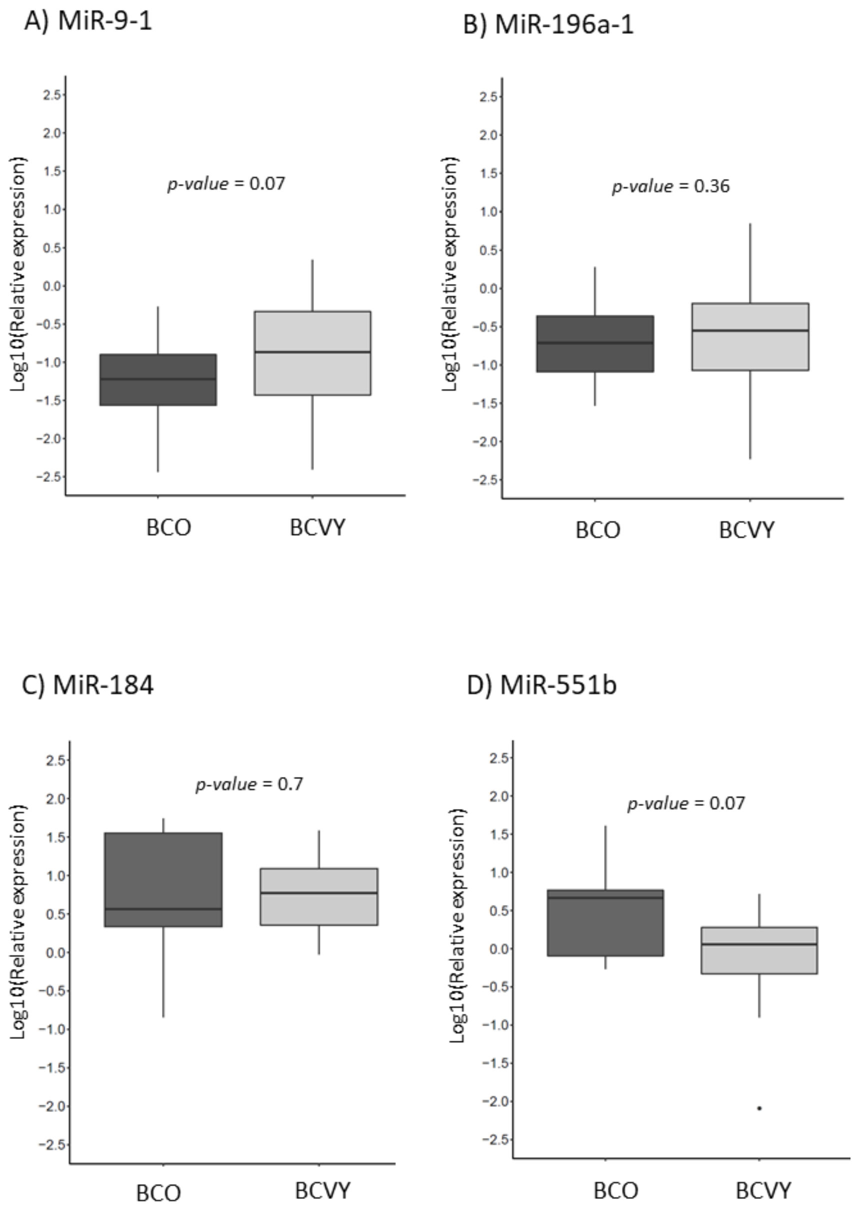

Supplementary Figure 3

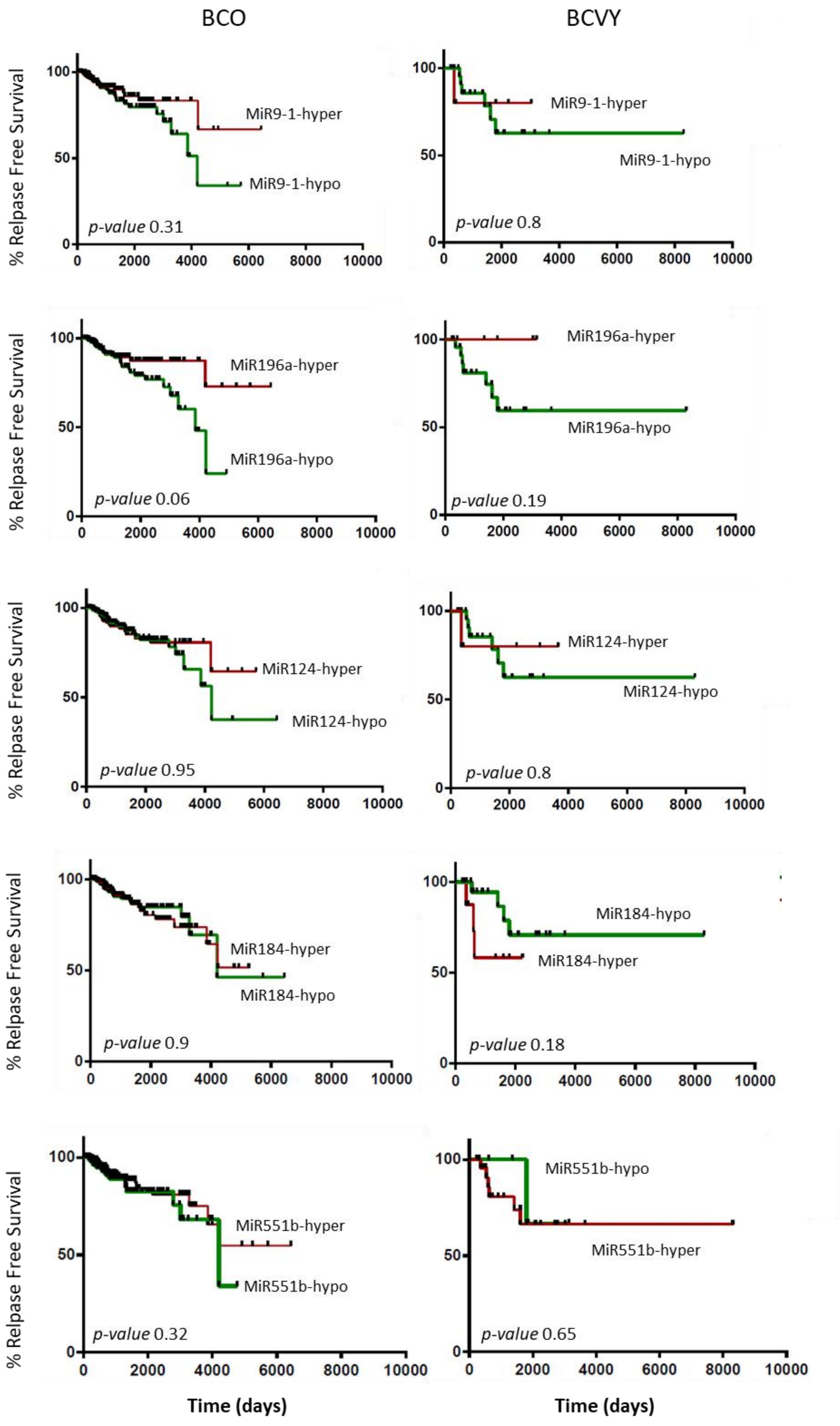

Supplementary Figure 4

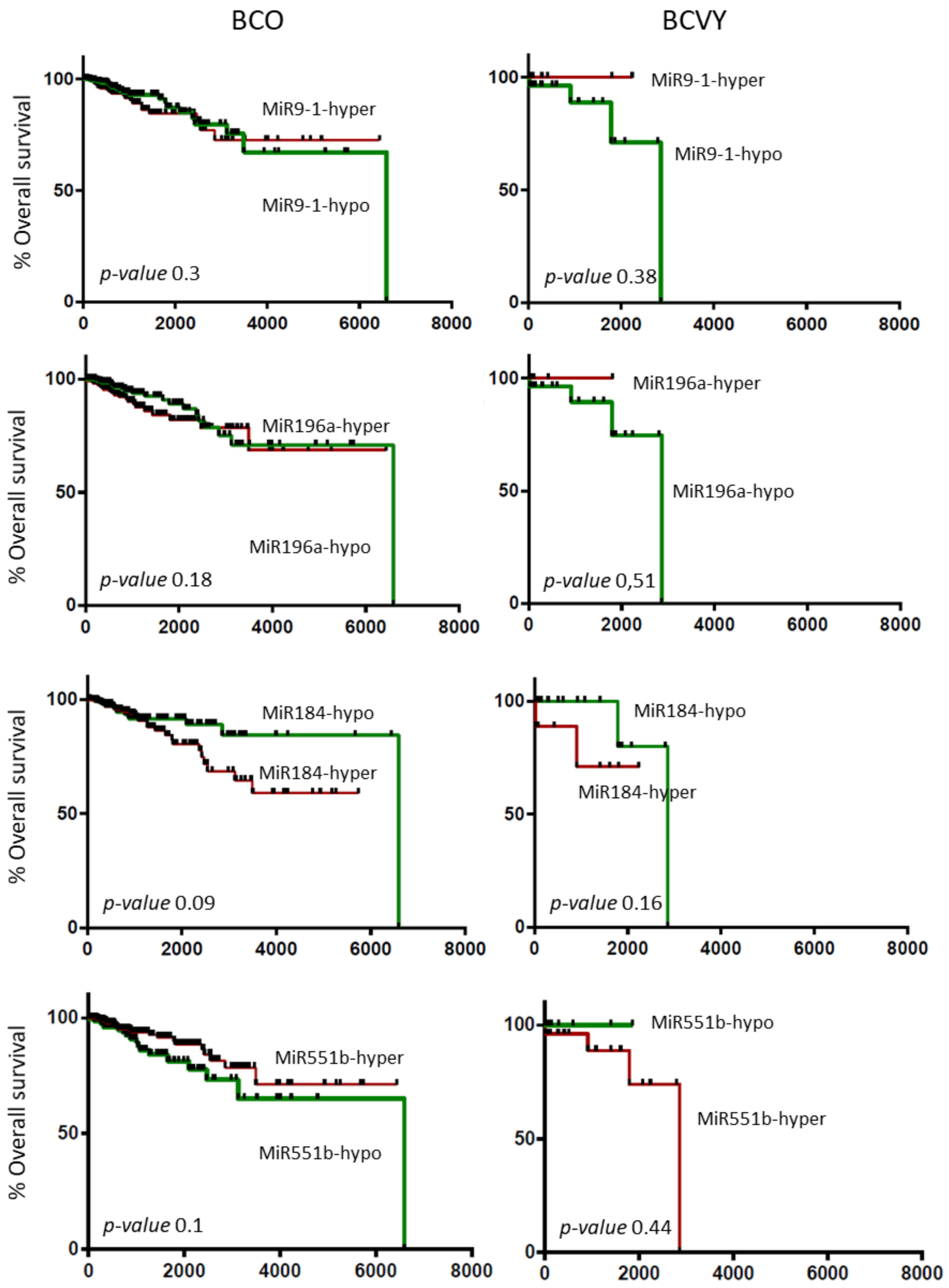

Supplement: Supplementary file 1 — Supplementary Figures [file 41598_2018_32393_MOESM1_ESM.pdf]
