# Supplementary material for: Methylation deregulation of miRNA promoters identifies miR124-2 as a survival biomarker in Breast Cancer in very young women
Source: Sci Rep. 2018 Sep 26;8:14373. doi: 10.1038/s41598-018-32393-3 (PMC6158237; doi:10.1038/s41598-018-32393-3)
Supplement: Supplementary file 2 — Supplementary Table 1 and 2 [file 41598_2018_32393_MOESM2_ESM.pdf]

Methylation deregulation of miRNA promoters identifies miR124-2 as a survival biomarker in Breast Cancer in very young women

Sara S. Oltra[1], Maria Peña-Chilet[1], Victoria Vidal-Tomas [1], Kirsty Flower[2], María Teresa Martinez[1], Elisa Alonso[3], Octavio Burgues[3], Ana Lluch[1]¥, James M. Flanagan[2], and Gloria Ribas[1]¥*

Supplementary Table 1: List of 193 CpG probes that were significantly differentially methylated in BCVY (*p-value* < 0.05 and methylation differences ± 0.1) obtained by Wilcoxon rank sum test. Table includes *p-values*, methylation differences between BCVY and BCO, miRNAs regulated by significant probe, probe position relative to gene and CpG island.

Probe	p-value	Methylation differences (BCVY - BCO)	UCSC RefGene Name	UCSC RefGene Group	Relation to Island
cg07234865	4,41E-05	-1,71E-01	MIR9-1	Body;5'UTR	N_Shore
cg25229253	5,40E-05	1,99E-01	MIR548F1	Body	OpenSea
cg08737296	8,02E-05	-2,86E-01	MIR124-3	TSS1500	Island
cg26258950	9,72E-05	1,81E-01	MIR548F1	Body	OpenSea
cg07642566	3,39E-04	-1,37E-01	MIR7-3	TSS1500;Body	OpenSea
cg15270145	4,00E-04	-1,63E-01	MIR9-1	5'UTR;TSS200	Island
cg06138966	4,71E-04	2,12E-01	MIR2113	TSS1500	OpenSea
cg06372015	4,71E-04	-1,25E-01	MIR129-2	TSS1500	N_Shore
cg23570321	5,52E-04	-1,28E-01	MIR3692	TSS1500;Body	OpenSea
cg26371731	6,47E-04	-1,58E-01	MIR9-1	TSS200;5'UTR	N_Shore
cg21099163	6,47E-04	1,96E-01	MIR548F1	ExonBnd;Body	OpenSea
cg21927946	6,47E-04	-1,07E-01	MIR7-3	TSS1500;Body	OpenSea
cg14301438	7,55E-04	1,21E-01	MIR1273H	Body	OpenSea
cg17192679	7,55E-04	-1,59E-01	MIR1256	1stExon;5'UTR;Body	Island
cg20582655	1,02E-03	-1,89E-01	MIR9-1	TSS200;5'UTR	N_Shore
cg11638181	1,37E-03	-1,34E-01	MIR129-2	TSS1500	N_Shore
cg07796261	1,58E-03	1,47E-01	MIR548F1	Body	OpenSea
cg01336796	1,58E-03	1,35E-01	MIR4439	TSS1500;Body	OpenSea
cg05775586	1,58E-03	1,83E-01	MIR873	TSS1500;5'UTR	OpenSea
cg03475101	1,58E-03	1,42E-01	MIR204	TSS1500;Body	OpenSea
cg26005082	1,58E-03	-1,30E-01	MIR7-3	TSS1500;Body	OpenSea
cg22333214	1,82E-03	-2,26E-01	MIR137	TSS200	S_Shore
cg09910993	1,82E-03	1,37E-01	MIR548F1	Body	OpenSea
cg10688790	1,82E-03	1,71E-01	MIR548F3	TSS1500;Body	OpenSea
cg15791171	2,08E-03	1,68E-01	MIR548F3	Body	OpenSea
cg07494702	2,08E-03	1,49E-01	MIR548F3	Body	OpenSea
cg06855983	2,08E-03	-1,27E-01	MIR181C;MIR181D	TSS1500	N_Shore
cg22807241	2,39E-03	1,62E-01	MIR548F3	Body	OpenSea
cg16256490	2,39E-03	1,58E-01	MIR383	TSS1500;Body	OpenSea
cg17528257	2,73E-03	1,12E-01	MIR551B	TSS200;Body	OpenSea
cg17396522	3,11E-03	1,12E-01	MIR548N	ExonBnd;Body	OpenSea
cg24720939	3,11E-03	1,65E-01	MIR548H2	Body	OpenSea
cg03976364	3,11E-03	1,73E-01	MIR5684	TSS1500	OpenSea
cg19004110	3,11E-03	1,40E-01	MIR383	TSS200;Body	OpenSea
cg17874329	3,54E-03	1,62E-01	MIR1273H	Body	OpenSea
cg03220633	3,54E-03	-1,52E-01	MIR2277	Body;TSS1500	S_Shore

cg01397141	3,54E-03	-1,92E-01	MIR184	Body	OpenSea
cg27559408	3,54E-03	1,65E-01	MIR330	TSS1500;Body	N_Shore
cg05423529	4,02E-03	-1,13E-01	MIR137	TSS200	S_Shore
cg18471021	4,02E-03	1,11E-01	MIR7853	Body	OpenSea
cg13285968	4,02E-03	-1,02E-01	MIR1258	5'UTR;TSS200	N_Shore
cg02174748	4,02E-03	1,24E-01	MIR300	TSS200	OpenSea
cg13031987	4,02E-03	1,12E-01	MIR548AE2	TSS1500;Body	N_Shore
cg13811969	4,02E-03	-1,49E-01	MIR1268A	TSS200;Body	N_Shore
cg19261968	4,56E-03	1,19E-01	MIR548F1	Body	OpenSea
cg03949274	4,56E-03	1,46E-01	MIR5684	TSS1500	OpenSea
cg04947764	4,56E-03	-1,98E-01	MIR184	TSS200	OpenSea
cg12116566	4,56E-03	-1,90E-01	MIR1256	5'UTR;Body	Island
cg02903877	5,16E-03	-1,29E-01	MIR1268A	Body	OpenSea
cg16407471	5,16E-03	-2,12E-01	MIR129-2	TSS200	Island
cg09243104	5,16E-03	1,16E-01	MIR380;MIR1197;MIR323A;MIR758	TSS1500	OpenSea
cg04927004	5,16E-03	-1,67E-01	MIR124-3	TSS1500	Island
cg26222940	5,83E-03	1,16E-01	MIR548N	5'UTR;Body	S_Shore
cg11074814	5,83E-03	-1,41E-01	MIR129-2	TSS1500	N_Shore
cg20260028	5,83E-03	1,66E-01	MIR211	TSS1500;Body	OpenSea
cg00722320	5,83E-03	-2,10E-01	MIR184	TSS200	OpenSea
cg02065637	5,83E-03	-2,31E-01	MIR124-3	TSS1500	Island
cg15854570	6,57E-03	1,19E-01	MIR548F1	TSS1500;Body	OpenSea
cg04355791	6,57E-03	-1,31E-01	MIR129-2	TSS1500	N_Shore
cg17297071	6,57E-03	-1,52E-01	MIR155HG	TSS200	Island
cg14315558	6,57E-03	-2,14E-01	MIR155HG	Body	Island
cg02176951	7,39E-03	1,34E-01	MIR548F1	Body	OpenSea
cg02758410	7,39E-03	1,30E-01	MIR548F1	Body	OpenSea
cg15164782	7,39E-03	1,13E-01	MIR548G	5'UTR;Body	OpenSea
cg06112642	7,39E-03	-1,20E-01	MIR6130	TSS1500;Body	S_Shore
cg14048837	7,39E-03	1,27E-01	MIR548Q	Body;5'UTR	OpenSea
cg01257685	7,39E-03	-1,06E-01	MIR548AZ	Body	OpenSea
cg06625777	7,39E-03	1,52E-01	MIR762HG	TSS1500;Body	S_Shore
cg05945782	7,39E-03	-1,07E-01	MIR212	TSS1500	Island
cg04936377	7,39E-03	1,12E-01	MIR1268A	Body	OpenSea
cg02405538	8,30E-03	-1,90E-01	MIR7853	TSS200;Body	N_Shore
cg00105060	8,30E-03	1,20E-01	MIR548T	Body	OpenSea
cg14416371	8,30E-03	-1,74E-01	MIR129-2	TSS200	Island
cg08096702	8,30E-03	1,72E-01	MIR211	TSS1500;Body	OpenSea
cg02921294	8,30E-03	-1,32E-01	MIR1256	Body;TSS200	N_Shore
cg06928021	9,30E-03	1,33E-01	MIR921	TSS1500;Body	OpenSea
cg00978822	9,30E-03	1,01E-01	MIR449B;MIR449A	TSS1500Body	N_Shore
cg26188365	9,30E-03	1,14E-01	MIR490	TSS1500;5'UTR;Body	OpenSea
cg16341266	9,30E-03	1,35E-01	MIR548I4	TSS1500;Body	OpenSea
cg15556502	9,30E-03	-2,15E-01	MIR129-2	TSS200	Island
cg02473781	9,30E-03	-1,01E-01	MIR130B;MIR301B	TSS1500;TSS200	S_Shore

cg19277411	1,04E-02	1,29E-01	MIR5684	Body	OpenSea
cg23545774	1,04E-02	1,46E-01	MIR3163	Body	OpenSea
cg04845520	1,04E-02	1,38E-01	MIR7641-2	TSS1500	OpenSea
cg05965535	1,04E-02	-1,14E-01	MIR1268A	ExonBnd;Body	OpenSea
cg03673191	1,04E-02	1,43E-01	MIR5009	Body	OpenSea
cg17762933	1,16E-02	-1,43E-01	MIR7853	TSS200;Body	N_Shore
cg24990327	1,16E-02	1,19E-01	MIR548F3	TSS1500;Body	OpenSea
cg03469437	1,16E-02	1,24E-01	MIR383	TSS200;Body	OpenSea
cg03678049	1,16E-02	-1,63E-01	MIR1268A	Body	OpenSea
cg14944647	1,16E-02	-1,81E-01	MIR129-2	TSS200	Island
cg02650317	1,16E-02	-1,72E-01	MIR124-3	TSS1500	Island
cg15028514	1,16E-02	-1,09E-01	MIR124-3	TSS200	Island
cg06660530	1,16E-02	-1,44E-01	MIR124-3	Body	Island
cg04282607	1,16E-02	-1,20E-01	MIR301B;MIR130B	Body;TSS1500	S_Shore
cg13578899	1,30E-02	1,12E-01	MIR548F1	Body	OpenSea
cg02559101	1,30E-02	1,05E-01	MIR1273E	Body	OpenSea
cg23936106	1,30E-02	1,12E-01	MIR217HG	Body	OpenSea
cg09836205	1,30E-02	1,63E-01	MIR2113	TSS200	OpenSea
cg02373104	1,30E-02	1,35E-01	MIR548F3	Body	OpenSea
cg08260861	1,30E-02	1,39E-01	MIR548T	Body	OpenSea
cg18309927	1,30E-02	-1,43E-01	MIR124-2HG	TSS200;Body	N_Shore
cg07792478	1,30E-02	-1,19E-01	MIR124-2	TSS1500	Island
cg16189671	1,30E-02	-1,09E-01	MIR124-2	TSS200	S_Shore
cg02540736	1,30E-02	1,12E-01	MIR345	TSS1500;TSS200	S_Shore
cg11673244	1,30E-02	-1,55E-01	MIR130B;MIR301B	TSS1500;TSS200	S_Shore
cg14282114	1,44E-02	1,03E-01	MIR6130	Body	OpenSea
cg21021332	1,44E-02	1,39E-01	MIR6130	Body	OpenSea
cg22688428	1,44E-02	-1,47E-01	MIR1247	TSS1500;TSS200	Island
cg16436686	1,60E-02	1,06E-01	MIR548F1	Body	OpenSea
cg13391521	1,60E-02	1,19E-01	MIR548F3	Body	OpenSea
cg17918158	1,60E-02	1,05E-01	MIR548G	5'UTR;Body	OpenSea
cg23917868	1,60E-02	-1,19E-01	MIR145	TSS200;Body	OpenSea
cg01939477	1,60E-02	-2,05E-01	MIR129-2	TSS200	Island
cg00608334	1,60E-02	-1,06E-01	MIR4497	TSS1500;5'UTR	N_Shore
cg02523350	1,60E-02	1,44E-01	MIR548AZ	Body	OpenSea
cg23787364	1,78E-02	1,11E-01	MIR1273E	Body	OpenSea
cg01942816	1,78E-02	1,14E-01	MIR589	Body;Body	S_Shore
cg18627360	1,78E-02	-1,05E-01	MIR124-3	TSS200	Island
cg12749863	1,78E-02	-1,72E-01	MIR155HG	Body	Island
cg03221073	1,97E-02	1,09E-01	MIR548F1	Body	OpenSea
cg02023150	1,97E-02	-1,82E-01	MIR5096	TSS1500;TSS200;Body	S_Shore
cg20505332	1,97E-02	1,24E-01	MIR548AC	Body	OpenSea
cg03436967	1,97E-02	1,36E-01	MIR548I4	Body	OpenSea
cg02582781	1,97E-02	1,35E-01	MIR548H3	Body	OpenSea
cg05376374	1,97E-02	-1,61E-01	MIR129-2	TSS200	Island

cg18301891	1,97E-02	-1,01E-01	MIR1247	TSS1500	Island
cg25147193	1,97E-02	-1,54E-01	MIR181C	TSS1500	Island
cg16759218	2,18E-02	1,13E-01	MIR548G	5'UTR;Body	OpenSea
cg25199591	2,18E-02	1,12E-01	MIR2113	TSS1500	OpenSea
cg03340334	2,18E-02	-1,24E-01	MIR1268A	Body	OpenSea
cg07035704	2,18E-02	1,13E-01	MIR2052	TSS1500	OpenSea
cg24750854	2,18E-02	-1,07E-01	MIR548H4	5'UTR;1stExon;Body	Island
cg23176340	2,18E-02	-1,27E-01	MIR7-2	TSS1500	OpenSea
cg00576773	2,18E-02	-1,05E-01	MIR9-3	TSS1500	N_Shore
cg04735310	2,18E-02	-1,22E-01	MIR196A1	TSS1500	Island
cg20277905	2,18E-02	-1,60E-01	MIR124-3	TSS200	Island
cg09005612	2,41E-02	1,15E-01	MIR548F1	Body	OpenSea
cg23460578	2,41E-02	-1,02E-01	MIR10B	TSS1500	N_Shore
cg13519595	2,41E-02	1,19E-01	MIR548A2	Body	OpenSea
cg10456132	2,41E-02	1,16E-01	MIR6130	TSS1500;Body	OpenSea
cg07101541	2,41E-02	1,34E-01	MIR548W	Body	OpenSea
cg10248218	2,41E-02	1,46E-01	MIR4526	TSS1500;Body	OpenSea
cg24669741	2,65E-02	1,02E-01	MIR548F1	1stExon;5'UTR;Body	OpenSea
cg10384855	2,65E-02	1,03E-01	MIR548F3	Body	OpenSea
cg21895505	2,65E-02	-1,61E-01	MIR5096	TSS1500;TSS200;Body	S_Shore
cg13716848	2,65E-02	1,43E-01	MIR548AJ2	Body	OpenSea
cg13332552	2,65E-02	1,14E-01	MIR2054	TSS1500	OpenSea
cg07618155	2,65E-02	-1,33E-01	MIR124-2HG	TSS200;Body	N_Shore
cg02398371	2,65E-02	1,27E-01	MIR548H3	Body	OpenSea
cg13555855	2,65E-02	-1,39E-01	MIR3663HG;MIR3663	Body;TSS1500	S_Shore
cg00362690	2,65E-02	-1,16E-01	MIR548H4	Body	OpenSea
cg18824446	2,65E-02	-1,29E-01	MIR7-2	TSS1500	OpenSea
cg09066676	2,65E-02	-1,01E-01	MIR662	TSS1500	OpenSea
cg24169735	2,65E-02	1,07E-01	MIR762HG	TSS1500;Body	S_Shore
cg11392297	2,65E-02	-1,22E-01	MIR1914	Body	S_Shore
cg14451430	2,92E-02	1,12E-01	MIR548F1	Body	OpenSea
cg21341558	2,92E-02	1,06E-01	MIR548G	Body	OpenSea
cg16062053	2,92E-02	1,44E-01	MIR551B	TSS1500;Body	OpenSea
cg10698928	2,92E-02	-1,69E-01	MIR124-2	TSS1500	Island
cg16318949	2,92E-02	-1,14E-01	MIR184	TSS200;Body	OpenSea
cg02694017	2,92E-02	-1,11E-01	MIR935	Body;TSS200	Island
cg15699267	2,92E-02	-2,07E-01	MIR124-3	TSS1500	Island
cg08378275	3,21E-02	1,11E-01	MIR548AE2	Body	S_Shelf
cg19769982	3,21E-02	-1,24E-01	MIR155HG	TSS1500	N_Shore
cg21503989	3,52E-02	-1,02E-01	MIR7853	Body	OpenSea
cg19982471	3,52E-02	-1,01E-01	MIR1258	Body;5'UTR	N_Shore
cg00972731	3,52E-02	1,02E-01	MIR548H2	Body	OpenSea
cg04185799	3,52E-02	1,07E-01	MIR548AJ2	Body	OpenSea
cg23841711	3,52E-02	1,16E-01	MIR548H3	Body	OpenSea
cg23976185	3,52E-02	1,03E-01	MIR31HG	Body	OpenSea

cg11210410	3,52E-02	1,19E-01	MIR1268A	Body	OpenSea
cg21884062	3,52E-02	-1,19E-01	MIR548F5	Body;TSS200	N_Shore
cg15558727	3,52E-02	1,00E-01	MIR4500HG	Body	OpenSea
cg24229568	3,52E-02	1,12E-01	MIR365A	TSS1500	OpenSea
cg13933773	3,52E-02	-1,21E-01	MIR1256	Body;TSS200	N_Shore
cg24987741	3,86E-02	1,02E-01	MIR3714	TSS1500;Body	OpenSea
cg23480273	3,86E-02	1,03E-01	MIR548AC	Body	OpenSea
cg00351980	3,86E-02	1,23E-01	MIR548G	5'UTR;Body	OpenSea
cg15164276	3,86E-02	1,02E-01	MIR551B	TSS200;Body	OpenSea
cg10123619	3,86E-02	1,12E-01	MIR6082	TSS1500	OpenSea
cg07426000	3,86E-02	1,01E-01	MIR3163	Body	OpenSea
cg05474726	3,86E-02	-1,43E-01	MIR124-2	TSS200	S_Shore
cg25720803	3,86E-02	-1,01E-01	MIR619	Body;TSS1500	OpenSea
cg08162457	4,22E-02	1,23E-01	MIR1273H	Body	OpenSea
cg06036509	4,22E-02	1,06E-01	MIR3163	Body	OpenSea
cg07007506	4,22E-02	-1,50E-01	MIR155HG	TSS1500	N_Shore
cg01334432	4,61E-02	1,06E-01	MIR205	Body;TSS1500	OpenSea
cg26374481	4,61E-02	1,28E-01	MIR548G	Body	OpenSea
cg00210994	4,61E-02	-1,33E-01	MIR548G	5'UTR;Body	N_Shore
cg27083040	4,61E-02	-1,08E-01	MIR145	TSS200;Body	OpenSea
cg03387135	4,61E-02	-1,40E-01	MIR124-3	TSS200	Island
cg23628411	4,61E-02	-1,09E-01	MIR1256	5'UTR;Body	S_Shore

Supplementary Table 2. Clinical tumour characteristics of samples from the EPIC array and the validation sample set.

	Discovery set n = 34		Validation set n = 40	
	BCVY (n = 21)	BCO (n = 13)	BCVY (n = 27)	BCO (n = 13)
**Age mean (SD)**	32.5 (2.7)	65.5 (8.5)	31.8 (3.2)	68.2 (7.3)
**Histological subtype (%)**				
Luminal A	2 (9.5)	2 (15.4)	3 (11.1)	4 (30.8)
Luminal B	8 (38.1)	4 (30.8)	11 (40.7)	5 (38.5)
TN	6 (28.8)	4 (30.8)	6 (22.2)	1 (7.7)
HER2	5 (23.8)	3 (23.1)	7 (25.9)	3 (23.1)
**ER status (%)**				
ER positive	11 (52.4)	9 (69.2)	15 (55.5)	10 (76.9)
ER negative	10 (47.62)	4 (30.8)	10 (37.0)	3 (23.1)
**Relapse (%)**	4 (19.05)	1 (7.69)	7 (25.9)	0

SD: standard deviation; TN: triple negative subtype; ER: oestrogen receptor; BCVY: breast cancer in very young women; BCO: breast cancer in older women.
