# Supplementary material for: Methylation deregulation of miRNA promoters identifies miR124-2 as a survival biomarker in Breast Cancer in very young women
Source: Sci Rep. 2018 Sep 26;8:14373. doi: 10.1038/s41598-018-32393-3 (PMC6158237; doi:10.1038/s41598-018-32393-3)
Supplement: Supplementary file 3 — Supplementary Table 3 [file 41598_2018_32393_MOESM3_ESM.pdf]

Methylation deregulation of miRNA promoters identifies miR124-2 as a survival biomarker in Breast Cancer
women. Sara S. Oltra1, Maria Peña-Chilet1, Victoria Vidal-Tomas 1, Kirsty Flower2, María Teresa Martinez1,
Octavio Burgues3, Ana Lluch1¥, James M. Flanagan2, and Gloria Ribas1¥*

Supplementary Table 3: Table includes information about correspondence between CpG probes and miRN
by them. Additionally, miRNAs may be regulated by unique or multiple probes (1-293). Probe count by miR
summarized in Supplementary Table 4.

CpG Probe	Regulated miRNAs					
cg01537494	MIR200B	MIR200A				
cg02825344	MIR200B	MIR200A				
cg02331673	MIR200B	MIR200A				
cg11203990	MIR200B	MIR200A				
cg13362546	MIR200A	MIR429				
cg21810793	MIR200A	MIR429				
cg25101291	MIR200A	MIR429				
cg16561256	MIR200A	MIR429				
cg16712243	MIR6727					
cg06883956	MIR551A					
cg15903571	MIR551A					
cg07720540	MIR551A					
cg07830126	MIR551A					
cg01432902	MIR551A					
cg12427039	MIR551A					
cg10506390	MIR4689					
cg10804699	MIR4689					
cg17776832	MIR6729					
cg24540396	MIR6729					
cg22487972	MIR7846					
cg18833720	MIR7846					
cg12002159	MIR7846					
cg05427056	MIR5096					
cg00061368	MIR5096					
cg03785447	MIR5096					
cg08540942	MIR5096					
cg13560937	MIR5096					
cg20524150	MIR5096					
cg20207416	MIR5096					
cg17344234	MIR5096					
cg10053134	MIR5096					
cg12846127	MIR5096					
cg12462099	MIR5096					
cg16129065	MIR5096					
cg05617775	MIR5096					
cg02650522	MIR3972					
cg25174210	MIR1290					
cg26824639	MIR1290					
cg21986272	MIR1290					
cg24343445	MIR1290					
cg21784980	MIR1290					

cg07298692	MIR1256					
cg06786434	MIR1256					
cg06271655	MIR3115					
cg00209355	MIR3115					
cg25059646	MIR3917					
cg17342214	MIR3917					
cg16183402	MIR3917					
cg01168343	MIR3917					
cg15707579	MIR3917					
cg24440131	MIR1976					
cg00359365	MIR1976					
cg24468934	MIR1976					
cg16395432	MIR1976					
cg09641213	MIR1976					
cg23955417	MIR1976					
cg11377047	MIR1976					
cg24993174	MIR4254					
cg12515083	MIR4254					
cg12999797	MIR4254					
cg13110636	MIR6732					
cg03909081	MIR30E					
cg26820000	MIR30E					
cg07387734	MIR30E					
cg14092259	MIR30E					
cg02482035	MIR30E					
cg06379876	MIR30E					
cg15984861	MIR30C1					
cg01959873	MIR30C1					
cg13716165	MIR30C1					
cg11135080	MIR30C1					
cg22674756	MIR30C1					
cg24700993	MIR30C1					
cg19872746	MIR6733					
cg07494239	MIR6733					
cg21063313	MIR6733					
cg09620514	MIR6733					
cg20407154	MIR6733					
cg08669738	MIR6733					
cg15280864	MIR6735					
cg10637930	MIR5584					
cg19716542	MIR761					
cg05876692	MIR761					
cg21108691	MIR761					
cg05909164	MIR1273F					
cg02813845	MIR1273F					
cg06360841	MIR1273G					
cg19784244	MIR4781					
cg13749777	MIR4781					
cg16698293	MIR4781					
cg13669156	MIR4781					

cg02058215	MIR548D2					
cg22525715	MIR548D2					
cg00763223	MIR548D2					
cg13691257	MIR548D2					
cg14777634	MIR548D2					
cg19238448	MIR548D2					
cg01067603	MIR548D2					
cg18642361	MIR548D2					
cg00944580	MIR548D2					
cg06339716	MIR4794					
cg25665165	MIR3671	MIR101-1				
cg22731975	MIR3671	MIR101-1				
cg02629942	MIR1262					
cg27455537	MIR1262					
cg25945989	MIR1262					
cg10164186	MIR186					
cg21603975	MIR186					
cg01118295	MIR186					
cg25434116	MIR186					
cg05624234	MIR7156					
cg11722227	MIR7156					
cg13575606	MIR548AP					
cg11929960	MIR548AP					
cg10806543	MIR548AP					
cg12338747	MIR548AP					
cg10774435	MIR548AP					
cg02216570	MIR548AP					
cg23820245	MIR548AP					
cg07034096	MIR548AP					
cg12746041	MIR548AP					
cg05851510	MIR548AP					
cg09254646	MIR548AP					
cg08696889	MIR548AP					
cg09556871	MIR4423					
cg09160344	MIR760					
cg13417096	MIR2682	MIR137	MIR137HG			
cg16789076	MIR2682	MIR137	MIR137HG			
cg23207534	MIR137	MIR137HG				
cg14007733	MIR548D1	MIR548AA1				
cg17308926	MIR548D1	MIR548AA1				
cg19709754	MIR548D1	MIR548AA1				
cg20263826	MIR548D1					
cg00700455	MIR548D1					
cg18375960	MIR553					
cg19154516	MIR553					
cg02490260	MIR553					
cg10824792	MIR7852					
cg09099500	MIR942					
cg19811108	MIR942					
cg25940485	MIR942					

cg19582647	MIR942					
cg01918824	MIR942					
cg00324023	MIR6736					
cg24771345	MIR5087					
cg00385751	MIR6878					
cg09680704	MIR6878					
cg05986111	MIR4257					
cg18616702	MIR4257					
cg16414821	MIR554					
cg18135087	MIR554					
cg02890365	MIR554					
cg10688896	MIR6737					
cg27157872	MIR6737					
cg10687301	MIR6737					
cg10239863	MIR6737					
cg02223139	MIR6737					
cg08446735	MIR6737					
cg11095157	MIR6737					
cg25824899	MIR6737					
cg14805136	MIR6737					
cg20448212	MIR4258					
cg17257175	MIR92B					
cg06420088	MIR92B					
cg03055449	MIR92B					
cg09292077	MIR555					
cg24836396	MIR555					
cg12446939	MIR555					
cg16998122	MIR555					
cg07234865	MIR9-1					
cg20582655	MIR9-1					
cg26371731	MIR9-1					
cg15270145	MIR9-1					
cg13149127	MIR9-1					
cg10778599	MIR9-1					
cg06609427	MIR9-1					
cg09793584	MIR9-1					
cg22630361	MIR9-1					
cg22629266	MIR765					
cg06266663	MIR765					
cg12604331	MIR765					
cg08480098	MIR765					
cg05345888	MIR765					
cg26543539	MIR765					
cg25935178	MIR4259					
cg21348515	MIR5187					
cg10855283	MIR5187					
cg00683996	MIR5187					
cg25682974	MIR5187					
cg22967866	MIR5187					
cg06783901	MIR556					

cg22341854	MIR556					
cg26766880	MIR556					
cg16746864	MIR921					
cg17940268	MIR921					
cg24428232	MIR921					
cg06928021	MIR921					
cg24554839	MIR921					
cg17323982	MIR921					
cg10332369	MIR921					
cg23910043	MIR1255B2					
cg12530592	MIR1255B2					
cg12176191	MIR1295					
cg16716044	MIR1295					
cg20972167	MIR214					
cg20269458	MIR214					
cg25214966	MIR199A2					
cg01006503	MIR199A2					
cg19219378	MIR199A2					
cg24002149	MIR199A2					
cg21450888	MIR199A2					
cg09281118	MIR199A2					
cg17178220	MIR199A2					
cg03322057	MIR199A2					
cg09043115	MIR199A2					
cg16084482	MIR488					
cg12033297	MIR488					
cg09024791	MIR488					
cg20323571	MIR488					
cg09005612	MIR548F1					
cg10987850	MIR548F1					
cg07796261	MIR548F1					
cg11333566	MIR548F1					
cg01188867	MIR548F1					
cg21099163	MIR548F1					
cg02176951	MIR548F1					
cg11344217	MIR548F1					
cg22439209	MIR548F1					
cg04384018	MIR548F1					
cg13393373	MIR548F1					
cg26258950	MIR548F1					
cg13578899	MIR548F1					
cg04616652	MIR548F1					
cg11029198	MIR548F1					
cg16201684	MIR548F1					
cg03221073	MIR548F1					
cg16436686	MIR548F1					
cg09411231	MIR548F1					
cg09910993	MIR548F1					
cg13459734	MIR548F1					
cg24264066	MIR548F1					

cg14451430	MIR548F1					
cg15854570	MIR548F1					
cg13274183	MIR548F1					
cg10801670	MIR548F1					
cg24505619	MIR548F1					
cg00066270	MIR548F1					
cg04788442	MIR548F1					
cg24669741	MIR548F1					
cg12626411	MIR548F1					
cg02758410	MIR548F1					
cg11245017	MIR548F1					
cg13801259	MIR548F1					
cg11227300	MIR548F1					
cg11460208	MIR548F1					
cg14156134	MIR548F1					
cg27097224	MIR548F1					
cg02989808	MIR548F1					
cg02544160	MIR548F1					
ch.1.3642016F	MIR548F1					
cg04312413	MIR548F1					
cg10387807	MIR548F1					
cg20041381	MIR548F1					
cg24577137	MIR548F1					
cg26803837	MIR548F1					
cg13365658	MIR548F1					
cg27342087	MIR548F1					
cg24356321	MIR548F1					
cg27294431	MIR548F1					
cg03992926	MIR548F1					
cg19510057	MIR548F1					
cg21751733	MIR548F1					
cg10524070	MIR548F1					
cg13065938	MIR548F1					
cg08910524	MIR548F1					
cg07667243	MIR548F1					
cg19452298	MIR548F1					
cg25811451	MIR548F1					
cg14676242	MIR548F1					
cg24379599	MIR548F1					
cg09390927	MIR548F1					
cg07387629	MIR548F1					
cg08970825	MIR548F1					
cg13899576	MIR548F1					
cg07897389	MIR548F1					
cg05193015	MIR548F1					
cg03434095	MIR548F1					
cg08582384	MIR548F1					
cg07840186	MIR548F1					
cg12917056	MIR548F1					
cg05535321	MIR548F1					

cg15809837	MIR548F1					
cg18670723	MIR548F1					
cg14745151	MIR548F1					
cg13393986	MIR548F1					
cg20786733	MIR548F1					
cg25214531	MIR548F1					
cg05709437	MIR548F1					
cg12723191	MIR548F1					
cg11873772	MIR548F1					
cg07472880	MIR548F1					
cg22351390	MIR548F1					
cg04875097	MIR1278					
cg03237401	MIR1278					
cg26481500	MIR1278					
cg08969297	MIR1278					
cg07435647	MIR1278					
cg26160460	MIR181B1	MIR181A1				
cg16010809	MIR181B1	MIR181A1				
cg00111333	MIR181B1	MIR181A1				
cg06105747	MIR181B1	MIR181A1	MIR181A1HG			
cg03426600	MIR181A1	MIR181A1HG				
cg17969792	MIR181A1	MIR181A1HG				
cg17360849	MIR5191					
cg11661940	MIR1231					
cg23236366	MIR1231					
cg10539122	MIR1231					
cg10987004	MIR1231					
cg12636482	MIR1231					
cg19093225	MIR6740					
cg26045471	MIR6740					
cg26455554	MIR6740					
cg20254084	MIR135B					
cg19841284	MIR135B					
cg18987126	MIR6769B					
cg07664909	MIR29C	MIR29B2				
cg00823526	MIR29B2	MIR29C				
cg27261597	MIR205					
cg05922993	MIR205					
cg01334432	MIR205					
cg17860090	MIR205					
cg17848546	MIR205					
cg03794445	MIR205					
cg20918933	MIR4260					
cg11377865	MIR3122					
cg03332116	MIR548F3					
cg16400754	MIR215	MIR194-1				
cg26289892	MIR215	MIR194-1				
cg26645003	MIR215	MIR194-1				
cg23617067	MIR194-1	MIR215				
cg24658737	MIR194-1	MIR215				

cg19530142	MIR320B2					
cg12740087	MIR320B2					
cg14502628	MIR6741					
cg17166334	MIR6742					
cg27592871	MIR6742					
cg22318562	MIR6742					
cg25850845	MIR1182					
cg21440584	MIR1182					
cg26032238	MIR1182					
cg03318314	MIR1182					
cg15987431	MIR1182					
cg01534125	MIR1182					
cg01569295	MIR1182					
cg20821842	MIR1182					
cg17441181	MIR1182					
cg08473764	MIR1182					
cg26614229	MIR1182					
cg02464665	MIR1182					
cg05445932	MIR5096					
cg26799245	MIR5096					
cg00242242	MIR5096					
cg02491119	MIR5096					
cg20461262	MIR5096					
cg18564052	MIR5096					
cg13712950	MIR5096					
cg02776913	MIR5096					
cg20650194	MIR5096					
cg06673490	MIR5096					
cg19152529	MIR5096					
cg20386024	MIR5096					
cg27665913	MIR5096					
cg14788420	MIR5096					
cg27628857	MIR5096					
cg09184742	MIR5096					
cg21580460	MIR5096					
cg09856671	MIR5096					
cg27656579	MIR5096					
cg17086320	MIR5096					
cg07608537	MIR5096					
cg20585792	MIR5096					
cg26189762	MIR5096					
cg07240301	MIR5096					
cg00617212	MIR5096					
cg13999701	MIR5096					
cg15404715	MIR5096					
cg15931313	MIR5096					
cg23632539	MIR5096					
cg13504602	MIR5096					
cg18341081	MIR5096					
cg21769534	MIR5096					

cg02023150	MIR5096					
cg21895505	MIR5096					
cg10866988	MIR5096					
cg16393730	MIR5096					
cg27304428	MIR5096					
cg03760658	MIR5096					
cg01011934	MIR5096					
cg16962115	MIR1537					
cg03678274	MIR1537					
cg00045689	MIR1537					
cg04144714	MIR1537					
cg07821529	MIR1273E					
cg25223332	MIR1273E					
cg16129451	MIR1273E					
cg22602349	MIR1273E					
cg00354771	MIR1273E					
cg17112200	MIR1273E					
cg15236354	MIR1273E					
cg13598366	MIR1273E					
cg00209105	MIR1273E					
cg12670110	MIR1273E					
cg04732087	MIR1273E					
cg07241406	MIR1273E					
cg01545863	MIR1273E					
cg14294034	MIR1273E					
cg04737542	MIR1273E					
cg22006089	MIR1273E					
cg14038077	MIR4677					
cg13494769	MIR4677					
cg12966865	MIR3124					
cg16864003	MIR3124					
cg03828080	MIR7515HG	MIR7515				
cg18755413	MIR7515HG	MIR7515				
cg15972734	MIR7515HG	MIR7515				
cg13670878	MIR4429					
cg00178749	MIR4429					
cg15960578	MIR3125					
cg05652528	MIR1301					
cg16892661	MIR1301					
cg05516842	MIR1301					
cg26803803	MIR1301					
cg10525105	MIR1301					
cg02746110	MIR1301					
cg02118630	MIR1301					
cg02606469	MIR558					
cg16769912	MIR558					
cg09247556	MIR558					
cg21058639	MIR558					
cg09874326	MIR558					
cg00956987	MIR558					

cg24443673	MIR548AD					
cg13823166	MIR559					
cg15792957	MIR559					
cg10285618	MIR559					
cg01866861	MIR8485					
cg09373730	MIR8485					
cg20668834	MIR8485					
cg12668144	MIR216B	MIR217HG				
cg27438307	MIR4434					
cg19298835	MIR4434					
cg00931692	MIR4434					
cg11941066	MIR6071					
cg20428383	MIR5696					
cg11503425	MIR5696					
cg10410507	MIR4265					
cg03065507	MIR4435-2HG					
cg03726156	MIR4435-2HG					
cg17190157	MIR4435-2HG					
cg02874133	MIR4435-2HG					
cg22686872	MIR4435-2HG					
cg24783876	MIR4435-2HG					
cg10621390	MIR4435-2HG					
cg25486386	MIR663B					
cg14691596	MIR663B					
cg13379731	MIR663B					
cg23682214	MIR7853					
cg01035219	MIR7853					
cg26266985	MIR7853					
cg18480318	MIR7853					
cg25317338	MIR7853					
cg26833654	MIR7853					
cg14506197	MIR7853					
cg13363808	MIR7853					
cg12232996	MIR7853					
cg26576274	MIR7853					
cg23289226	MIR7853					
cg16557125	MIR7853					
cg11390344	MIR7853					
cg16450290	MIR7853					
cg20769477	MIR7853					
cg03085443	MIR7853					
cg07330251	MIR7853					
cg14511781	MIR7853					
cg22687497	MIR7853					
cg21503989	MIR7853					
cg17592802	MIR7853					
cg17762933	MIR7853					
cg02405538	MIR7853					
cg02851043	MIR7853					
cg07469205	MIR7853					

cg23062120	MIR7853					
cg15427166	MIR7853					
cg18471021	MIR7853					
cg22247160	MIR7853					
cg04221848	MIR7853					
cg26471191	MIR7853					
cg14592981	MIR7853					
cg16498879	MIR7853					
cg15928974	MIR7853					
cg26837747	MIR7853					
cg16208848	MIR7853					
cg07510373	MIR7853					
cg04840148	MIR7853					
cg14471282	MIR7853					
cg25930602	MIR7853					
cg02440797	MIR7853					
cg21472830	MIR7853					
cg11479653	MIR7853					
cg18584042	MIR7853					
cg22097777	MIR7853					
cg08471449	MIR7853					
cg25409638	MIR7853					
cg01243312	MIR128-1					
cg27339255	MIR1978					
cg00213281	MIR1978					
cg05826944	MIR1978					
cg07459181	MIR1978					
cg18900544	MIR4785					
cg22084004	MIR4785					
cg17307471	MIR4774					
cg08631141	MIR4774					
cg06064461	MIR933					
cg19447671	MIR933					
cg02622866	MIR933					
cg26965059	MIR933					
cg00829688	MIR933					
cg01943478	MIR933					
cg19758054	MIR933					
cg08643007	MIR933					
cg14931631	MIR933					
cg17504336	MIR933					
cg18637574	MIR933					
cg17668949	MIR933					
cg12480658	MIR933					
cg15573536	MIR933					
cg01737698	MIR933					
cg19045894	MIR933					
cg07011913	MIR10B					
cg12127282	MIR10B					
cg08717880	MIR10B					

cg27160395	MIR10B					
cg17104824	MIR10B					
cg00014998	MIR10B					
cg25942990	MIR10B					
cg01152019	MIR10B					
cg14399060	MIR10B					
cg00767581	MIR10B					
cg04248916	MIR7704					
cg01923073	MIR7704					
cg23157415	MIR7704					
cg24386890	MIR7704					
cg09633152	MIR4444-1	MIR4444-2				
cg16427534	MIR6512					
cg00789792	MIR548N					
cg04486436	MIR548N					
cg24192368	MIR548N					
cg21182526	MIR548N					
cg18251859	MIR548N					
cg13348640	MIR548N					
cg14253764	MIR548N					
cg13667965	MIR548N					
cg17563622	MIR548N					
cg00580354	MIR548N					
cg13151361	MIR548N					
cg04697953	MIR548N					
cg27480241	MIR548N					
cg00460704	MIR548N					
cg15580874	MIR548N					
cg14386946	MIR548N					
cg19060227	MIR548N					
cg04432606	MIR548N					
cg11770407	MIR548N					
cg03012876	MIR548N					
cg00534253	MIR548N					
cg06226973	MIR548N					
cg20310435	MIR548N					
cg17833257	MIR548N					
cg11625255	MIR548N					
cg13411604	MIR548N					
cg14807549	MIR548N					
cg23927983	MIR548N					
cg03724445	MIR548N					
cg25102782	MIR548N					
cg06333167	MIR548N					
cg09890200	MIR548N					
cg24808667	MIR548N					
cg00352349	MIR548N					
cg21446511	MIR548N					
cg20370152	MIR548N					
cg05106421	MIR548N					

cg18351741	MIR548N					
cg26222940	MIR548N					
cg21868410	MIR548N					
cg25572910	MIR548N					
cg03956640	MIR548N					
cg15658577	MIR548N					
cg21749200	MIR548N					
cg16419334	MIR548N					
cg14284588	MIR548N					
cg22050893	MIR548N					
cg03941238	MIR548N					
cg16298238	MIR548N					
cg08735147	MIR548N					
cg24230840	MIR548N					
cg06125055	MIR548N					
cg11969913	MIR548N					
cg10949583	MIR548N					
cg03283842	MIR548N					
cg11548914	MIR548N					
cg24560920	MIR548N					
cg05098551	MIR548N					
cg11251378	MIR548N					
cg06341370	MIR548N					
cg04053862	MIR548N					
cg23412136	MIR548N					
cg17458653	MIR548N					
cg06287708	MIR548N					
cg26149223	MIR548N					
cg12089249	MIR548N					
cg03991736	MIR548N					
cg18193109	MIR548N					
cg11539052	MIR548N					
cg01659489	MIR548N					
cg13962724	MIR548N					
cg02342910	MIR548N					
cg04888852	MIR548N					
cg06807315	MIR548N					
cg04321497	MIR548N					
cg15609237	MIR548N					
cg19906284	MIR548N					
cg08691235	MIR548N					
cg08736813	MIR548N					
cg18977423	MIR548N					
cg16214269	MIR548N					
cg02659030	MIR548N					
cg10038720	MIR548N					
cg13313833	MIR548N					
cg10087519	MIR548N					
cg09915519	MIR548N					
cg17933893	MIR548N					

cg16910097	MIR548N					
cg06867904	MIR548N					
cg27495572	MIR548N					
cg10939966	MIR548N					
cg03814399	MIR548N					
cg14792798	MIR548N					
cg09777256	MIR548N					
cg14869618	MIR548N					
cg01690287	MIR548N					
cg17396522	MIR548N					
cg18183294	MIR548N					
cg16234718	MIR548N					
cg22950670	MIR548N					
cg24600426	MIR548N					
cg27352348	MIR548N					
cg05273635	MIR548N					
cg15569739	MIR548N					
cg16826256	MIR548N					
cg01103718	MIR548N					
cg06316114	MIR548N					
cg14195166	MIR548N					
cg16516429	MIR548N					
cg19982471	MIR1258					
cg16705245	MIR1258					
cg13285968	MIR1258					
cg01452873	MIR1258					
cg10866755	MIR1258					
cg22657780	MIR1258					
cg24575676	MIR1258					
cg11113760	MIR1258					
cg09279240	MIR1258					
cg06695611	MIR1258					
cg16400999	MIR1258					
cg14670435	MIR1258					
cg00651020	MIR1258					
cg05687686	MIR1258					
cg11854219	MIR4437					
cg26753290	MIR561					
cg11354643	MIR561					
cg04103432	MIR1245A					
cg08237722	MIR1245A					
cg15476410	MIR1245A					
cg21558423	MIR1245B					
cg25080630	MIR3129					
cg22124629	MIR3130-1					
cg26006165	MIR2355					
cg17827949	MIR2355					
cg18929085	MIR2355					
cg06161762	MIR7845					
cg15993931	MIR7845					

cg07566172	MIR7845					
cg05337739	MIR7845					
cg07865161	MIR548F2					
cg20953052	MIR548F2					
cg00332776	MIR6809					
cg14777783	MIR6513					
cg27291385	MIR26B					
cg15616915	MIR26B					
cg19223411	MIR26B					
cg08460635	MIR26B					
cg14045814	MIR26B					
cg23131909	MIR26B					
cg05787409	MIR375					
cg13723257	MIR375					
cg01822124	MIR375					
cg18854412	MIR375					
cg26394220	MIR375					
cg12981188	MIR3131					
cg23869335	MIR153-1					
cg24411913	MIR153-1					
cg04671367	MIR153-1					
cg10225197	MIR153-1					
cg02571840	MIR3132					
cg01336796	MIR4439					
cg12337227	MIR4439					
cg06324993	MIR5703					
cg05729783	MIR5703					
cg27002958	MIR5703					
cg18906489	MIR5703					
cg06832651	MIR4777					
cg14084144	MIR5001					
cg23743058	MIR5001					
cg04813781	MIR5001					
cg05037455	MIR6811					
cg12285570	MIR4441					
cg24119006	MIR4269					
cg13414834	MIR149					
cg01712737	MIR149					
cg11574092	MIR149					
cg13232118	MIR149					
cg17367832	MIR149					
cg06591185	MIR149					
cg24133207	MIR149					
cg08495468	MIR378B					
cg10093972	MIR885					
cg20745248	MIR885					
cg24605341	MIR885					
cg23230910	MIR885					
cg05365685	MIR885					
cg15254736	MIR885					

cg07314821	MIR885					
cg12115190	MIR885					
cg06444180	MIR885					
cg24987741	MIR3714					
cg01047111	MIR548AC					
cg20505332	MIR548AC					
cg20015729	MIR548AC					
cg12416878	MIR548AC					
cg16119643	MIR548AC					
cg11782594	MIR548AC					
cg15928247	MIR548AC					
cg01966636	MIR548AC					
cg20806436	MIR548AC					
cg15688583	MIR548AC					
cg08004406	MIR548AC					
cg26022159	MIR548AC					
cg04591012	MIR548AC					
cg23480273	MIR548AC					
cg00841784	MIR548AC					
cg24956366	MIR548AC					
cg25459931	MIR548AC					
cg12309867	MIR548AC					
cg14485651	MIR548AC					
cg22978384	MIR128-2					
cg02179478	MIR128-2					
cg09736928	MIR128-2					
cg08490094	MIR128-2					
cg24102420	MIR128-2					
cg21384588	MIR128-2					
cg13383435	MIR128-2					
cg23220346	MIR128-2					
cg26160492	MIR26A1					
cg04787317	MIR26A1					
cg08171483	MIR26A1					
cg26054057	MIR26A1					
cg07816047	MIR26A1					
cg10866847	MIR26A1					
cg15555217	MIR26A1					
cg08922540	MIR6822					
cg26949465	MIR6822					
cg16615211	MIR564					
cg08430604	MIR564					
cg24674304	MIR564					
cg25189074	MIR564					
cg13740840	MIR564					
cg23569156	MIR564					
cg10911865	MIR564					
cg07542475	MIR564					
cg02160333	MIR564					
cg17410922	MIR564					

cg00979307	MIR1226					
cg22067839	MIR1226					
cg14777519	MIR1226					
cg18775606	MIR1226					
cg15085006	MIR1226					
cg11521721	MIR1226					
cg05411186	MIR1226					
cg01090930	MIR1226					
cg21933155	MIR6823					
cg20436810	MIR711					
cg19219068	MIR711					
cg09150212	MIR711					
cg11565377	MIR711					
cg01112720	MIR6824					
cg02629170	MIR6824					
cg04257209	MIR4793					
cg07109801	MIR425					
cg27634187	MIR425					
cg04878973	MIR425					
cg06365567	MIR425					
cg25691553	MIR425					
cg01142001	MIR425					
cg17217654	MIR425					
cg15463769	MIR191	MIR425				
cg17092349	MIR425	MIR191				
cg19526908	MIR425	MIR191				
cg19767734	MIR191	MIR425				
cg03861029	MIR191	MIR425				
cg19682856	MIR425	MIR191				
cg14764512	MIR191	MIR425				
cg04792715	MIR191	MIR425				
cg00490249	MIR191	MIR425				
cg25312696	MIR425	MIR191				
cg14069251	MIR425	MIR191				
cg26874558	MIR425	MIR191				
cg20610269	MIR425	MIR191				
cg21038223	MIR425	MIR191				
cg14148944	MIR191	MIR425				
cg21019820	MIR191					
cg23479905	MIR191					
cg07725313	MIR6890					
cg24584723	MIR6890					
cg17988347	MIR4271					
cg12741606	MIR566					
cg06362328	MIR566					
cg07969202	MIR566					
cg22991739	MIR5787					
cg14035970	MIR5787					
cg03345933	MIR5787					
cg17259862	MIR6872					

cg07610339	MIR6872					
cg23581009	MIR6872					
cg17790804	MIR6872					
cg08673882	MIR4787					
cg09066298	MIRLET7G					
cg19019198	MIRLET7G					
cg25583774	MIRLET7G					
cg20891622	MIRLET7G					
cg00906833	MIR135A1					
cg02017047	MIR135A1					
cg01600732	MIR3938					
cg03690528	MIR548A2					
cg02953897	MIR548A2					
cg19376734	MIR548A2					
cg00832222	MIR548A2					
cg03981818	MIR548A2					
cg10926971	MIR548A2					
cg06615524	MIR548A2					
cg27308177	MIR548A2					
cg26526531	MIR548A2					
cg01970886	MIR548A2					
cg23015354	MIR548A2					
cg20382675	MIR1284					
cg18558767	MIR1284					
cg15226786	MIR1284					
cg21585477	MIR1284					
cg18951035	MIR4795					
cg27434411	MIR548G					
cg08078966	MIR548G					
cg03277051	MIR548G					
cg20948024	MIR548G					
cg18056266	MIR548G					
cg09782454	MIR548G					
cg19968403	MIR548G					
cg02441647	MIR548G					
cg05283542	MIR548G					
cg24198004	MIR548G					
cg26105803	MIR548G					
cg17207485	MIR548G					
cg16759218	MIR548G					
cg21175685	MIR548G					
cg21010715	MIR548G					
cg01628181	MIR548G					
cg15165735	MIR548G					
cg08243711	MIR548G					
cg18344769	MIR548G					
cg09765831	MIR548G					
cg09648366	MIR548G					
cg21900069	MIR548G					
cg18114034	MIR548G					

cg25091526	MIR548G					
cg22952011	MIR548G					
cg19229209	MIR548G					
cg20530613	MIR548G					
cg13409084	MIR548G					
cg11019088	MIR548G					
cg17918158	MIR548G					
cg25199192	MIR548G					
cg01443549	MIR548G					
cg22366214	MIR548G					
cg14976283	MIR548G					
cg00351980	MIR548G					
cg14460963	MIR548G					
cg15164782	MIR548G					
cg25162651	MIR548G					
cg24123724	MIR548G					
cg07805332	MIR548G					
cg17388683	MIR548G					
cg08539350	MIR548G					
cg13477253	MIR548G					
cg03275067	MIR548G					
cg15077343	MIR548G					
cg12196473	MIR548G					
cg11168235	MIR548G					
cg05642802	MIR548G					
cg03059470	MIR548G					
cg00104570	MIR548G					
cg01854420	MIR548G					
cg06062590	MIR548G					
cg08331946	MIR548G					
cg08905723	MIR548G					
cg25826640	MIR548G					
cg03617850	MIR548G					
cg01227064	MIR548G					
cg00648285	MIR548G					
cg03021388	MIR548G					
cg21589858	MIR548G					
cg09271158	MIR548G					
cg17047890	MIR548G					
cg20009349	MIR548G					
cg00695271	MIR548G					
cg06888463	MIR548G					
cg05906075	MIR548G					
cg15055095	MIR548G					
cg22481643	MIR548G					
cg26234786	MIR548G					
cg06758801	MIR548G					
cg03656137	MIR548G					
cg07600990	MIR548G					
cg09573197	MIR548G					

cg23221889	MIR548G					
cg00015159	MIR548G					
cg13612275	MIR548G					
cg08350549	MIR548G					
cg00210994	MIR548G					
cg15963552	MIR548G					
cg20276377	MIR548G					
cg13132965	MIR548G					
cg04271054	MIR548G					
cg22092811	MIR548G					
cg18337222	MIR548G					
cg03671265	MIR548G					
cg13790797	MIR548G					
cg14394740	MIR548G					
cg10561392	MIR548G					
cg21419939	MIR548G					
cg14196304	MIR548G					
cg16808912	MIR548G					
cg04411201	MIR548G					
cg26273312	MIR548G					
cg05878887	MIR548G					
cg06077670	MIR548G					
cg06533586	MIR548G					
cg00991994	MIR548G					
cg15518046	MIR548G					
cg05396688	MIR548G					
cg24828422	MIR548G					
cg04371579	MIR548G					
cg17983017	MIR548G					
cg11268077	MIR548G					
cg05759046	MIR548G					
cg04606556	MIR548G					
cg07552538	MIR548G					
cg17241937	MIR548G					
cg04114269	MIR548G					
cg13219301	MIR548G					
cg04569855	MIR548G					
cg04521697	MIR3921	MIR548G				
cg00573148	MIR548G					
cg09957895	MIR548G					
cg04016922	MIR548G					
cg00814786	MIR548G					
cg16318181	MIR548G					
cg18804562	MIR548G					
cg10620048	MIR567					
cg05966699	MIR567					
cg13717684	MIR567					
cg01397968	MIR567					
cg18627042	MIR568					
cg08840687	MIR198					

cg22894604	MIR198					
cg00445142	MIR198					
cg23405198	MIR198					
cg17473262	MIR7110					
cg01184905	MIR6825					
cg01598284	MIR6825					
cg12809539	MIR6825					
cg04323831	MIR6825					
cg10782455	MIR6825					
cg18321315	MIR6825					
cg27292264	MIR1280					
cg05801066	MIR1280					
cg19505196	MIR1280					
cg16821175	MIR1280					
cg14208839	MIR1280					
cg17068417	MIR1280					
cg22484330	MIR548H2					
cg04394119	MIR548H2					
cg13090484	MIR548H2					
cg16794867	MIR548H2					
cg01965463	MIR548H2					
cg26827139	MIR548H2					
cg01030411	MIR548H2					
cg27641106	MIR548H2					
cg24720939	MIR548H2					
cg00972731	MIR548H2					
cg16245944	MIR548H2					
cg04596122	MIR548H2					
cg12509383	MIR548H2					
cg16226436	MIR548H2					
cg15334941	MIR548H2					
cg00060683	MIR548H2					
cg06386451	MIR548H2					
cg15847886	MIR548H2					
cg26663696	MIR15B	MIR16-2				
cg13783238	MIR15B	MIR16-2				
cg12785694	MIR15B	MIR16-2				
cg17554896	MIR16-2	MIR15B				
cg04001997	MIR16-2	MIR15B				
cg09100593	MIR16-2					
cg21490751	MIR551B					
cg07747690	MIR551B					
cg16062053	MIR551B					
cg15164276	MIR551B					
cg17528257	MIR551B					
cg21658839	MIR551B					
cg09652501	MIR569					
cg01543197	MIR569					
cg18042072	MIR569					
cg16179239	MIR548AY					

cg01895189	MIR548AY					
cg22008020	MIR548AY					
cg09704444	MIR548AY					
cg05171064	MIR548AY					
cg14553506	MIR1224					
cg23059797	MIR1224					
cg10187475	MIR1224					
cg00299558	MIR1224					
cg17009978	MIR1224					
cg12821315	MIR1224					
cg03981074	MIR1224					
cg09618015	MIR1224					
cg01026256	MIR1224					
cg21545762	MIR1224					
cg09616647	MIR1224					
cg11660531	MIR5588					
cg09438228	MIR548AQ					
cg05789925	MIR1248					
cg20674424	MIR1248					
cg24082174	MIR1248					
cg05199755	MIR1248					
cg27374881	MIR1248					
cg00670721	MIR1248					
cg01712079	MIR28					
cg23010205	MIR944					
cg19400113	MIR944					
cg09085792	MIR944					
cg15245749	MIR944					
cg27227930	MIR570					
cg04605696	MIR4797					
cg15435900	MIR922					
cg10579986	MIR922					
cg06336230	MIR922					
cg17304531	MIR922					
cg19463199	MIR922					
cg12023911	MIR922					
cg27013765	MIR922					
cg18834352	MIR922					
cg09559242	MIR922					
cg19975560	MIR922					
cg04548705	MIR571					
cg10303145	MIR571					
cg18304251	MIR571					
cg25291692	MIR943					
cg23952550	MIR943					
cg20680819	MIR943					
cg03189678	MIR943					
cg21238254	MIR943					
cg04288299	MIR943					
cg24951514	MIR943					

cg07550016	MIR943					
cg04889413	MIR4274					
cg23398781	MIR95					
cg25926549	MIR95					
cg13370209	MIR95					
cg27033302	MIR95					
cg00352325	MIR5091					
cg19628270	MIR5091					
cg16683394	MIR5091					
cg21129041	MIR218-1					
cg06316739	MIR218-1					
cg00749969	MIR218-1					
cg10603296	MIR218-1					
cg07711036	MIR218-1					
cg22508905	MIR218-1					
cg21766808	MIR574					
cg17223176	MIR574					
cg27389764	MIR574					
cg04252242	MIR574					
cg16919074	MIR574					
cg24518794	MIR574					
cg27212978	MIR574					
cg03499052	MIR574					
cg02628902	MIR574					
cg11532267	MIR574					
cg05616278	MIR574					
cg08055924	MIR574					
cg00818473	MIR574					
cg10387769	MIR574					
cg25943096	MIR574					
cg03084103	MIR574					
cg02699698	MIR1273H					
cg16246356	MIR1273H					
cg05674309	MIR1273H					
cg22567852	MIR1273H					
cg15029882	MIR1273H					
cg03955542	MIR1273H					
cg15534778	MIR1273H					
cg11732729	MIR1273H					
cg08162457	MIR1273H					
cg10503919	MIR1273H					
cg06833610	MIR1273H					
cg07848274	MIR1273H					
cg13018338	MIR1273H					
cg10380484	MIR1273H					
cg13280484	MIR1273H					
cg10605649	MIR1273H					
cg00058903	MIR1273H					
cg12004639	MIR1273H					
cg24962826	MIR1273H					

cg06678432	MIR1273H					
cg07017842	MIR1273H					
cg02499945	MIR1273H					
cg05731995	MIR1273H					
cg11589462	MIR1273H					
cg01622001	MIR1273H					
cg13144371	MIR1273H					
cg01366462	MIR1273H					
cg10713842	MIR1273H					
cg04933317	MIR1273H					
cg12289524	MIR1273H					
cg21387653	MIR1273H					
cg17874329	MIR1273H					
cg22311686	MIR1273H					
cg12344891	MIR1273H					
cg09538777	MIR1273H					
cg05670935	MIR1273H					
cg15071233	MIR1273H					
cg09503809	MIR1273H					
cg06321636	MIR1273H					
cg02446672	MIR1273H					
cg14278514	MIR1273H					
cg20818807	MIR1273H					
cg26953669	MIR5591	MIR1273H				
cg02877191	MIR1273H					
cg20009044	MIR1273H					
cg17446457	MIR1273H					
cg16167943	MIR1273H					
cg17844867	MIR1273H					
cg18839354	MIR1273H					
cg24735829	MIR1273H					
cg08729793	MIR1273H					
cg18124756	MIR1273H					
cg27025274	MIR1273H					
cg20185964	MIR1273H					
cg15335915	MIR1273H					
cg17431830	MIR1273H					
cg08589987	MIR1273H					
cg15466196	MIR1273H					
cg16437766	MIR1273H					
cg13671094	MIR1273H					
cg21321611	MIR1273H					
cg23551256	MIR1273H					
cg26777800	MIR1273H					
cg12532854	MIR1273H					
cg04787349	MIR1273H					
cg13083186	MIR1273H					
cg06265999	MIR1273H					
cg15272005	MIR1273H					
cg12874961	MIR1273H					

cg14301438	MIR1273H					
cg12533689	MIR4802					
cg05672616	MIR575					
cg04237666	MIR575					
cg18948646	MIR575					
cg09031823	MIR575					
cg08030987	MIR575					
cg09363604	MIR575					
cg03650550	MIR575					
cg19057326	MIR575					
cg10066125	MIR1255A					
cg02147683	MIR1255A					
cg10021238	MIR367					
cg02875879	MIR302A	MIR302D	MIR367			
cg06540697	MIR367	MIR302D	MIR302C	MIR302A		
cg14719129	MIR302D	MIR302A	MIR302B	MIR302C	MIR367	
cg04086871	MIR367	MIR302D	MIR302A	MIR302C	MIR302B	
cg05868449	MIR367	MIR302D	MIR302A	MIR302C	MIR302B	
cg12093220	MIR302D	MIR302B	MIR302A	MIR302C		
cg15939539	MIR1243					
cg11175091	MIR1243					
cg03204787	MIR577					
cg14640220	MIR577					
cg19426761	MIR577					
cg01003872	MIR4799					
cg11214083	MIR4453					
cg14738611	MIR4453					
cg03562412	MIR4453					
cg06772049	MIR578					
cg06169041	MIR578					
cg10857203	MIR578					
cg09538819	MIR1979					
cg26518884	MIR1979					
cg06549863	MIR1979					
cg15700520	MIR4635					
cg14209764	MIR6075					
cg04959716	MIR4458HG					
cg26423185	MIR4458HG					
cg04828267	MIR4458HG					
cg18269595	MIR4458HG					
cg08322697	MIR4458HG	MIR4458				
cg07072293	MIR4636					
cg11941971	MIR4636					
cg19089725	MIR4637					
cg07364760	MIR887					
cg06017405	MIR887					
cg21477068	MIR887					
cg10252212	MIR887					
cg09149005	MIR887					
cg19713787	MIR887					

cg26566472	MIR4279					
cg23863230	MIR579					
cg10269619	MIR579					
cg18190776	MIR579					
cg14042821	MIR7641-2					
cg08478427	MIR7641-2					
cg21470876	MIR7641-2					
cg05239577	MIR580					
cg25013978	MIR580					
cg26171815	MIR580					
cg11367853	MIR3650					
cg03135982	MIR3650					
cg23019039	MIR581					
cg26019654	MIR581					
cg01578939	MIR581					
cg13650580	MIR449A					
cg26894722	MIR449A	MIR449B				
cg19296299	MIR449A	MIR449B				
cg00978822	MIR449B	MIR449A				
cg01988325	MIR449B	MIR449A				
cg25728115	MIR449A	MIR449B				
cg03902122	MIR449C					
cg07042371	MIR449C					
cg05288356	MIR449C					
cg02711460	MIR449C					
cg15433309	MIR449C					
cg14753740	MIR449C					
cg21604660	MIR449C					
cg25626508	MIR449C					
cg26487966	MIR449C					
cg05005023	MIR449C					
cg07451859	MIR449C					
cg18654971	MIR449C					
cg15201847	MIR449C					
cg14079131	MIR449C					
cg07707227	MIR449C					
cg10928257	MIR449C					
cg21905516	MIR3607					
cg21240555	MIR3607					
cg03638907	MIR3607					
cg09942712	MIR3607					
cg23035142	MIR9-2					
cg20857794	MIR9-2					
cg10576384	MIR9-2					
cg13237382	MIR9-2					
cg15411403	MIR548AO					
cg17588371	MIR548AO					
cg06616408	MIR548AO					
cg05246994	MIR548AO					
cg04877466	MIR548AO					

cg23551527	MIR548AO					
cg17170590	MIR548AO					
cg15774976	MIR548AO					
cg05001715	MIR2277					
cg25771615	MIR2277					
cg21830711	MIR2277					
cg09848695	MIR2277					
cg19875969	MIR2277					
cg22703659	MIR2277					
cg17533458	MIR2277					
cg07803236	MIR2277					
cg05880353	MIR2277					
cg06401019	MIR2277					
cg03220633	MIR2277					
cg16695999	MIR1974					
cg00964137	MIR1974					
cg03964851	MIR1974					
cg22733357	MIR1974					
cg23504723	MIR548P					
cg10511110	MIR548P					
cg07988843	MIR548P					
cg19152802	MIR548F3					
cg02562921	MIR548F3					
cg03214468	MIR4633					
cg15254366	MIR4633					
cg20943266	MIR6830					
cg25966737	MIR6830					
cg01023783	MIR3936					
cg01516119	MIR1289-2					
cg10205925	MIR1289-2					
cg08360457	MIR1289-2					
cg03307581	MIR1289-2					
cg14205800	MIR3661					
cg19032799	MIR874					
cg04184179	MIR874					
cg03894789	MIR874					
cg22030962	MIR874					
cg18251187	MIR874					
cg04986004	MIR874					
cg23186640	MIR6831					
cg25520280	MIR6831					
cg24920847	MIR6831					
cg17915286	MIR6831					
cg14759064	MIR3655					
cg02726137	MIR3655					
cg02370376	MIR3655					
cg13688408	MIR143HG					
cg01726320	MIR143HG					
cg26585864	MIR143HG					
cg00113247	MIR143HG					

cg16109075	MIR143HG					
cg20090508	MIR143HG					
cg27167181	MIR143HG					
cg09655487	MIR143HG					
cg17416790	MIR143HG					
cg03543120	MIR143					
cg07125256	MIR143					
cg04317047	MIR143					
cg16684117	MIR143					
cg21242144	MIR143					
cg12675571	MIR145					
cg24549289	MIR145					
cg17856005	MIR145					
cg27083040	MIR145					
cg23917868	MIR145					
cg11671363	MIR145					
cg22941668	MIR145					
cg08537847	MIR145					
cg25768776	MIR378A					
cg27200158	MIR378A					
cg09970855	MIR378A					
cg09865068	MIR378A					
cg10230957	MIR378A					
cg19318253	MIR6499					
cg11216595	MIR378H					
cg03407996	MIR103-1	MIR103-1AS				
cg27137124	MIR103A1					
cg25938287	MIR103-1					
cg12812838	MIR103-1					
cg05664895	MIR218-2					
cg27271216	MIR218-2					
cg26502194	MIR218-2					
cg09887953	MIR218-2					
cg24141911	MIR218-2					
cg00534479	MIR218-2					
cg17324804	MIR585					
cg24185852	MIR585					
cg25592746	MIR585					
cg07569575	MIR585					
cg11855741	MIR585					
cg23573591	MIR3912					
cg04354589	MIR3912					
cg24404973	MIR3912					
cg05063169	MIR3912					
cg17872779	MIR3912					
cg08981957	MIR1271					
cg13786191	MIR4281					
cg18319687	MIR1229					
cg19726536	MIR1229					
cg16578938	MIR1229					

cg05851148	MIR1229					
cg16370061	MIR1229					
cg03834394	MIR1229					
cg03341985	MIR1229					
cg06189266	MIR1229					
cg09133836	MIR340					
cg03369432	MIR340					
cg23035172	MIR340					
cg12697637	MIR340					
cg04851992	MIR340					
cg00004915	MIR8089					
cg08486060	MIR8089					
cg24291455	MIR4638					
cg06831953	MIR6720					
cg09433733	MIR3143					
cg05142576	MIR3143					
cg01704860	MIR877					
cg12065914	MIR877					
cg01627201	MIR877					
cg10030624	MIR877					
cg23104816	MIR877					
cg20635067	MIR877					
cg17327665	MIR877					
cg04292889	MIR877					
cg17711252	MIR4640					
cg24636809	MIR4640					
cg25613385	MIR4640					
cg23222808	MIR4640					
cg18577693	MIR4640					
cg12316667	MIR4640					
cg25771580	MIR1236					
cg06165213	MIR1236					
cg18051316	MIR1236					
cg09583599	MIR1236					
cg08810053	MIR1236					
cg01869058	MIR1236					
cg10223234	MIR1236					
cg25511278	MIR219-1					
cg19356389	MIR219-1					
cg26106778	MIR219-1					
cg11011640	MIR219-1					
cg18486707	MIR219-1					
cg03341723	MIR219-1					
cg25026287	MIR219-1					
cg27331144	MIR219-1					
cg02214637	MIR6834					
cg20255906	MIR1234					
cg00468163	MIR5004					
cg05525200	MIR5004					
cg21789597	MIR5690					

cg22216174	MIR6780B					
cg11304866	MIR4647					
cg13061103	MIR586					
cg26014966	MIR5685					
cg01211496	MIR4282					
cg25275283	MIR548H3					
cg05893251	MIR548H3					
cg09705232	MIR548H3					
cg13957432	MIR548H3					
cg15778867	MIR548H3					
cg23300129	MIR548H3					
cg15968925	MIR548H3					
cg19535507	MIR548H3					
cg23689404	MIR548H3					
cg22395021	MIR548H3					
cg12172080	MIR548H3					
cg26759943	MIR548H3					
cg16511841	MIR548H3					
cg17774634	MIR548H3					
cg23858074	MIR548H3					
cg00472393	MIR548H3					
cg11182931	MIR548H3					
cg22682162	MIR548H3					
cg22566643	MIR548H3					
cg10518340	MIR548H3					
cg21877580	MIR548H3					
cg07439409	MIR548H3					
cg03108701	MIR548H3					
cg18863314	MIR548H3					
cg13873343	MIR548H3					
cg19562453	MIR548H3					
cg15134168	MIR548H3					
cg14756724	MIR548H3					
cg23766333	MIR548H3					
cg23841711	MIR548H3					
cg19366816	MIR587					
cg03697143	MIR587					
cg27364880	MIR587					
cg12396622	MIR548B					
cg08687105	MIR548B					
cg06445043	MIR548B					
cg05835950	MIR3145					
cg19698987	MIR3163					
cg00637980	MIR3163					
cg19016265	MIR3163					
cg12965535	MIR3163					
cg13237947	MIR3163					
cg02471248	MIR3163					
cg17198132	MIR3163					
cg12482648	MIR3163					

cg07426000	MIR3163					
cg22574343	MIR3163					
cg20889285	MIR3163					
cg18051234	MIR3163					
cg20999110	MIR3163					
cg18605905	MIR3163					
cg09571887	MIR3163					
cg04706583	MIR3163					
cg06036509	MIR3163					
cg23545774	MIR3163					
cg00752601	MIR3163					
cg08759764	MIR4466					
cg23570321	MIR3692					
cg13057938	MIR7641-2					
cg23676215	MIR7641-2					
cg00884195	MIR7641-2					
cg16970310	MIR7641-2					
cg14649665	MIR7641-2					
cg18762422	MIR1913					
cg02588532	MIR1913					
cg23804785	MIR1913					
cg09089341	MIR1913					
cg09406630	MIR1913					
cg17871838	MIR1913					
cg05005235	MIR1913					
cg21020852	MIR3939					
cg13528713	MIR3939					
cg12934576	MIR3939					
cg07883606	MIR3939					
cg27205928	MIR339					
cg16492851	MIR339					
cg04682977	MIR339					
cg10089657	MIR339					
cg13727277	MIR339					
cg17581870	MIR339					
cg09174653	MIR339					
cg02022181	MIR339					
cg06242730	MIR339					
cg21308528	MIR4655					
cg12338935	MIR4655					
cg23184739	MIR4655					
cg13063348	MIR6836					
cg11807318	MIR4648					
cg09196059	MIR4648					
cg16011164	MIR4656					
cg01942816	MIR589					
cg22108567	MIR589					
cg25343388	MIR589					
cg09286367	MIR589					
cg17419731	MIR589					

cg01024247	MIR589					
cg00966405	MIR589					
cg04155485	MIR589					
cg03040622	MIR589					
cg02355304	MIR589					
cg20511815	MIR589					
cg17149568	MIR1302-6					
cg15802249	MIR1302-6					
cg26792730	MIR1183					
cg00240971	MIR1183					
cg17777998	MIR1183					
cg15293367	MIR196B					
cg18134292	MIR196B					
cg11642336	MIR550B1					
cg12570246	MIR1200					
cg22492271	MIR1200					
cg11845241	MIR1200					
cg00629788	MIR6837					
cg15444722	MIR6838					
cg21840031	MIR4649					
cg24709463	MIR4649					
cg12487742	MIR4649					
cg20912014	MIR4649					
cg21414933	MIR4649					
cg16954452	MIR4649					
cg16315945	MIR4657					
cg04102916	MIR590					
cg05354946	MIR590					
cg26840824	MIR590					
cg07733057	MIR590					
cg01459030	MIR590					
cg18773807	MIR4651					
cg12348198	MIR4651					
cg16120102	MIR653					
cg26749833	MIR489	MIR653				
cg03816029	MIR489	MIR653				
cg21317815	MIR489					
cg04650977	MIR489					
cg22669561	MIR489					
cg04138378	MIR591					
cg09021393	MIR591					
cg18964251	MIR591					
cg19624555	MIR591					
cg26977749	MIR3609					
cg09184764	MIR3609					
cg09555403	MIR3609					
cg01290725	MIR3609					
cg24854681	MIR25					
cg22420044	MIR25					
cg09522706	MIR25	MIR93				

cg22638766	MIR25	MIR93				
cg00170236	MIR25	MIR93				
cg08971171	MIR25	MIR93				
cg06218186	MIR25	MIR106B	MIR93			
cg17022727	MIR25	MIR106B	MIR93			
cg03265037	MIR25	MIR106B	MIR93			
cg02924836	MIR25	MIR106B	MIR93			
cg09238235	MIR25	MIR106B	MIR93			
cg00287016	MIR25	MIR106B	MIR93			
cg09538129	MIR25	MIR106B	MIR93			
cg09186183	MIR25	MIR93	MIR106B			
cg00094751	MIR106B	MIR93				
cg10094907	MIR106B					
cg18745690	MIR4658					
cg14701566	MIR4658					
cg19680554	MIR4658					
cg20422157	MIR4658					
cg22266405	MIR4658					
cg14224139	MIR4658					
cg22018180	MIR4658					
cg23391045	MIR4658					
cg08314089	MIR4658					
cg15355620	MIR6840					
cg24142470	MIR6840					
cg01674169	MIR6840					
cg07133355	MIR6840					
cg27008901	MIR6875					
cg24190430	MIR6875					
cg18547866	MIR6875					
cg09946681	MIR548O					
cg14344583	MIR548O					
cg13673375	MIR548O					
cg13075119	MIR548O					
cg08111064	MIR5090					
cg18053783	MIR5090					
cg10506249	MIR5090					
cg11857310	MIR3666					
cg12402823	MIR6132					
cg20332907	MIR6132					
cg14158769	MIR592					
cg02090654	MIR592					
cg17540499	MIR592					
cg15908975	MIR592					
cg11694513	MIR593					
cg03326399	MIR593					
cg00002531	MIR593					
cg26768703	MIR593					
cg00872676	MIR593					
cg01877565	MIR183	MIR96				
cg06242719	MIR183	MIR96				

cg03104765	MIR183	MIR96				
cg00314904	MIR183	MIR96				
cg18161285	MIR183	MIR96				
cg16321266	MIR183	MIR96				
cg14794735	MIR183	MIR96				
cg22047901	MIR183	MIR96				
cg04819959	MIR335					
cg24037795	MIR335					
cg08761490	MIR335					
cg21434355	MIR29B1	MIR29A				
cg03856510	MIR29B1	MIR29A				
cg03776809	MIR29B1					
cg13396334	MIR3654					
cg26312916	MIR6509					
cg10242760	MIR6509					
cg10979181	MIR490					
cg26188365	MIR490					
cg10493317	MIR490					
cg21200229	MIR490					
cg16486721	MIR6892					
cg00710715	MIR6892					
cg20052308	MIR6892					
cg13192883	MIR6892					
cg11769486	MIR6892					
cg19139509	MIR548I4					
cg14355023	MIR548F4	MIR548I4				
cg25429902	MIR548F4	MIR548I4				
cg09335647	MIR548I4					
cg01615815	MIR548I4					
cg26636917	MIR548I4					
cg06880494	MIR548I4					
cg14627974	MIR548I4					
cg03436967	MIR548I4					
cg06927522	MIR548I4					
cg02515354	MIR548I4					
cg16341266	MIR548I4					
cg26548251	MIR548F3					
cg01123783	MIR548F3					
cg24990327	MIR548F3					
cg10688790	MIR548F3					
cg02373104	MIR548F3					
cg16595458	MIR548F3					
cg09362918	MIR548T					
cg22807241	MIR548F3					
cg00105060	MIR548T					
cg15578140	MIR548F3					
cg04486528	MIR548F3					
cg14739284	MIR548T					
cg09597759	MIR548T					
cg18970103	MIR548T					

cg00826997	MIR548T					
cg01067813	MIR548T					
cg23548616	MIR548T					
cg17096328	MIR548T					
cg08260861	MIR548T					
cg17198937	MIR548T					
cg22507969	MIR548T					
cg16975584	MIR548T					
cg03184350	MIR548T					
cg05013730	MIR548T					
cg08374341	MIR548T					
cg19796617	MIR548T					
cg01722003	MIR548T					
cg09981964	MIR548T					
cg23998942	MIR548T					
cg22556505	MIR548T					
cg19686543	MIR548T					
cg22735402	MIR548T					
cg06755671	MIR548T					
cg04720886	MIR548T					
cg17868330	MIR548T					
cg05640346	MIR548T					
cg02756921	MIR671					
cg00860590	MIR671					
cg12616484	MIR671					
cg02572956	MIR671					
cg15529111	MIR671					
cg06083642	MIR671					
cg04382077	MIR671					
cg18479961	MIR671					
cg10832012	MIR671					
cg18476993	MIR671					
cg17548326	MIR3907					
cg16477744	MIR153-2					
cg10310310	MIR153-2					
cg03361379	MIR153-2					
cg12779445	MIR153-2					
cg17527969	MIR153-2					
cg08791395	MIR153-2					
cg01088352	MIR153-2					
cg10893656	MIR153-2					
cg12877853	MIR595					
cg15187151	MIR595					
cg26308704	MIR595					
cg06747087	MIR595					
cg06179179	MIR595					
cg08865574	MIR7160					
cg22246148	MIR4659B					
cg06460618	MIR4659B					
cg00678005	MIR597					

cg25708403	MIR597					
cg13048261	MIR597					
cg06142662	MIR597					
cg25304543	MIR597					
cg06032483	MIR597					
cg00571033	MIR124-1					
cg01275681	MIR124-1					
cg14278808	MIR124-1					
cg15248835	MIR124-1					
cg12616174	MIR124-1					
cg06292304	MIR124-1					
cg24185864	MIR124-1					
cg18246262	MIR124-1					
cg02690648	MIR1322					
cg17371911	MIR1322					
cg02167149	MIR1322					
cg20332679	MIR1322					
cg13457217	MIR1322					
cg04194933	MIR1322					
cg02310165	MIR598					
cg18339098	MIR598					
cg01706263	MIR598					
cg14201324	MIR598					
cg04963089	MIR598					
cg25010142	MIR3926-2					
cg19004110	MIR383					
cg03469437	MIR383					
cg03748458	MIR383					
cg16256490	MIR383					
cg22778206	MIR383					
cg03368399	MIR320A					
cg21925296	MIR320A					
cg06112727	MIR320A					
cg08316073	MIR320A					
cg06333435	MIR320A					
cg11699593	MIR320A					
cg10206887	MIR320A					
cg25284208	MIR320A					
cg08264885	MIR320A					
cg14294245	MIR6876					
cg22217144	MIR6843					
cg21111824	MIR6843					
cg21867571	MIR6843					
cg10734892	MIR6843					
cg12489601	MIR6843					
cg00471826	MIR4287					
cg06820725	MIR7641-2					
cg05527782	MIR7641-2					
cg20196141	MIR7641-2					
cg26454401	MIR7641-2					

cg21565218	MIR548O2					
cg09761153	MIR548O2					
cg01491603	MIR548O2					
cg08386913	MIR548O2					
cg07262266	MIR548O2					
cg18132919	MIR548O2					
cg02235235	MIR548O2					
cg17404487	MIR548O2					
cg17836499	MIR548O2					
cg08382292	MIR548O2					
cg09801901	MIR548O2					
cg17367733	MIR548O2					
cg18672880	MIR548O2					
cg27369048	MIR548O2					
cg10471143	MIR548O2					
cg14140920	MIR548O2					
cg11611793	MIR548O2					
cg01128614	MIR548O2					
cg15295468	MIR548O2					
cg11625387	MIR548O2					
cg23100309	MIR548O2					
cg13413777	MIR548O2					
cg01344051	MIR548O2					
cg21968223	MIR548O2					
cg06264430	MIR548O2					
cg03248910	MIR548O2					
cg13558115	MIR486-2					
cg03311185	MIR486-2					
cg05836659	MIR486-2					
cg08504601	MIR486-2					
cg13603914	MIR486					
cg10794439	MIR486					
cg17886457	MIR486					
cg10715527	MIR486					
cg10945180	MIR486-1					
cg22180675	MIR486-1					
cg04319844	MIR486					
cg00328284	MIR486					
cg05066959	MIR486					
cg19214487	MIR4469					
cg05535168	MIR4470					
cg07817409	MIR4470					
cg05722052	MIR4470					
cg05250467	MIR124-2HG					
cg04249036	MIR124-2HG					
cg18309927	MIR124-2HG					
cg06080856	MIR124-2HG					
cg25499471	MIR124-2HG					
cg07618155	MIR124-2HG					
cg25678108	MIR124-2HG					

cg19119970	MIR378D2					
cg22413205	MIR378D2					
cg12957092	MIR378D2					
cg10801015	MIR378D2					
cg06204973	MIR378D2					
cg27238381	MIR378D2					
cg03463578	MIR378D2					
cg19712386	MIR599					
cg10500570	MIR599	MIR875				
cg24919348	MIR599	MIR875				
cg10659042	MIR599	MIR875				
cg19681251	MIR1273A					
cg24315859	MIR1273A					
cg20544279	MIR1273A					
cg02742934	MIR5680					
cg14732116	MIR5680					
cg00088264	MIR5680					
cg25847416	MIR5680					
cg00293796	MIR3151					
cg03317309	MIR2053					
cg15943335	MIR2053					
cg22679949	MIR2053					
cg13589481	MIR2053					
cg27593320	MIR3610					
cg18588204	MIR3610					
cg23283234	MIR1204					
cg03457528	MIR1204					
cg08334153	MIR1204					
cg13784855	MIR1204					
cg25247520	MIR1204					
cg11201447	MIR1204					
cg27190014	MIR1205					
cg03611307	MIR1205					
cg22353369	MIR1205					
cg05480350	MIR1205					
cg14105551	MIR1205					
cg08633665	MIR1205					
cg23063070	MIR1206					
cg01084257	MIR1206					
cg01621145	MIR1206					
cg04945860	MIR1207					
cg16934485	MIR1207					
cg05076730	MIR1207					
cg26180383	MIR1207					
cg02243522	MIR1207					
cg01940297	MIR1207					
cg07084941	MIR1207					
cg18290852	MIR3686					
cg13719966	MIR7848					
cg08573869	MIR937					

cg18004197	MIR937					
cg16627915	MIR937					
cg08936078	MIR937					
cg17082719	MIR937					
cg02850815	MIR937					
cg13509702	MIR937					
cg15650298	MIR937					
cg19670588	MIR6845					
cg00128812	MIR661					
cg10451724	MIR661					
cg24120597	MIR661					
cg16434940	MIR661					
cg26373812	MIR661					
cg00611614	MIR661					
cg09235308	MIR661					
cg04018533	MIR661					
cg02140559	MIR661					
cg24137216	MIR661					
cg19119464	MIR661					
cg09553160	MIR6846					
cg19997557	MIR6847					
cg00694874	MIR6847					
cg22358108	MIR6847					
cg17395859	MIR6848					
cg09045465	MIR6848					
cg15604056	MIR6848					
cg13131168	MIR6848					
cg13330760	MIR6848					
cg08453238	MIR6848					
cg02151939	MIR6848					
cg21254731	MIR939					
cg05012516	MIR939					
cg08172304	MIR939					
cg05972303	MIR939					
cg22638461	MIR939					
cg07471156	MIR1234					
cg27617225	MIR1234					
cg03515927	MIR1234					
cg00503998	MIR1234					
cg02369245	MIR6849					
cg11861389	MIR1234					
cg20555305	MIR1234					
cg03636657	MIR1234					
cg17741937	MIR6849					
cg22384877	MIR1234					
cg09056223	MIR1234					
cg02672776	MIR6893					
cg02224202	MIR6850					
cg14719465	MIR6850					
cg07625237	MIR6850					

cg19069367	MIR6850					
cg12903452	MIR6850					
cg25526243	MIR6850					
cg12389845	MIR6850					
cg12094723	MIR6850					
cg07103258	MIR101-2					
cg02133084	MIR101-2					
cg12538810	MIR4665					
cg12495376	MIR31HG					
cg05687603	MIR31	MIR31HG				
cg04901018	MIR31	MIR31HG				
cg07241951	MIR876					
cg05775586	MIR873					
cg21576021	MIR6851					
cg07725673	MIR6851					
cg06921261	MIR4667					
cg08207531	MIR4667					
cg17340314	MIR4667					
cg06257733	MIR6852					
cg08471950	MIR6852					
cg11347475	MIR6853					
cg00122951	MIR6853					
cg08882264	MIR6853					
cg23532270	MIR6853					
cg06227369	MIR6853					
cg04522339	MIR4540					
cg26054672	MIR204					
cg03475101	MIR204					
cg10456132	MIR6130					
cg02254327	MIR6130					
cg20292224	MIR6130					
cg12452440	MIR6130					
cg02294239	MIR6130					
cg06398756	MIR6130					
cg06112642	MIR6130					
cg02121060	MIR6130					
cg11384475	MIR6130					
cg03308555	MIR6130					
cg05206385	MIR6130					
cg08942734	MIR7-1					
cg04299170	MIR7-1					
cg11728484	MIR3153					
cg19177732	MIR3153					
cg04819135	MIR3153					
cg04946456	MIR4290					
cg10829374	MIR4290					
cg04158241	MIR3651					
cg14820613	MIR3651					
cg01024878	MIR548AU					
cg15651103	MIR548AU					

cg20815911	MIRLET7A1	MIRLET7F1				
cg10386111	MIRLET7F1	MIRLET7A1				
cg21799523	MIRLET7D	MIRLET7DHG				
cg05555502	MIRLET7DHG					
cg02277272	MIRLET7DHG					
cg04582108	MIR2278					
cg06265451	MIR2278					
cg04169578	MIR2278					
cg20555778	MIR2278					
cg08849095	MIR23B					
cg00351472	MIR23B	MIR27B				
cg24104437	MIR23B	MIR27B				
cg07975356	MIR27B	MIR24-1	MIR23B			
cg26417468	MIR27B	MIR24-1	MIR23B			
cg09229423	MIR24-1					
cg02268620	MIR24-1					
cg20355301	MIR24-1					
cg00532885	MIR24-1					
cg02324722	MIR24-1					
cg09951047	MIR24-1					
cg12017968	MIR3074					
cg05022212	MIR1302-8					
cg06812263	MIR1302-8					
cg06889514	MIR6854					
cg04451615	MIR6854					
cg19444866	MIR6854					
cg25355826	MIR6854					
cg21281732	MIR6854					
cg17201343	MIR548Q					
cg14048837	MIR548Q					
cg10068516	MIR548Q					
cg16246882	MIR548Q					
cg14057303	MIR548Q					
cg08838825	MIR548Q					
cg10096536	MIR548Q					
cg14130004	MIR548Q					
cg01951972	MIR548Q					
cg20134331	MIR548Q					
cg13496359	MIR548Q					
cg03602781	MIR548Q					
cg02408243	MIR548Q					
cg01418386	MIR548Q					
cg18508449	MIR548Q					
cg22785801	MIR548Q					
cg13973641	MIR548Q					
cg23330818	MIR548Q					
cg25844969	MIR548Q					
cg03706840	MIR548Q					
cg08446512	MIR548Q					
cg13595195	MIR548Q					

cg04885140	MIR548Q					
cg10016185	MIR548Q					
cg05821976	MIR548Q					
cg22500349	MIR548Q					
cg13234893	MIR548Q					
cg13576904	MIR548Q					
cg00020229	MIR548Q					
cg12479035	MIR548Q					
cg01590557	MIR548Q					
cg10545141	MIR548Q					
cg09779010	MIR548Q					
cg15139258	MIR32					
cg01920129	MIR32					
cg08527343	MIR32					
cg07704578	MIR3134					
cg15133882	MIR3134					
cg00476022	MIR3134					
cg03835940	MIR3134					
cg23563692	MIR3134					
cg25774694	MIR3134					
cg08000025	MIR3134					
cg09767648	MIR3134					
cg01630798	MIR3134					
cg06845425	MIR3134					
cg10183426	MIR3134					
cg22459858	MIR3134					
cg15841006	MIR3134					
cg12326635	MIR3134					
cg22166550	MIR3134					
cg07494164	MIR3134					
cg15067802	MIR3134					
cg10974064	MIR3134					
cg11929218	MIR3134					
cg08115465	MIR3134					
cg26683906	MIR3134					
cg06513099	MIR3134					
cg05878876	MIR3134					
cg22415746	MIR3134					
cg06033953	MIR3134					
cg07313836	MIR3134					
cg05046382	MIR3134					
cg05392988	MIR3134					
cg11199714	MIR3134					
cg09890892	MIR3134					
cg11740035	MIR3134					
cg00410048	MIR3134					
cg03415664	MIR3134					
cg15325530	MIR3134					
cg27072201	MIR3134					
cg14728609	MIR3134					

cg14862827	MIR3134					
cg21046659	MIR3134					
cg24239321	MIR3134					
cg22887281	MIR3134					
cg11297378	MIR3134					
cg15806381	MIR3134					
cg12303909	MIR3134					
cg22189216	MIR3134					
cg09449167	MIR3134					
cg11499685	MIR3134					
cg16423770	MIR3134					
cg05273161	MIR3134					
cg11000817	MIR3134					
cg00291004	MIR3134					
cg03014004	MIR3134					
cg05410331	MIR3134					
cg15167811	MIR3134					
cg11512305	MIR3134					
cg07089575	MIR3134					
cg23404491	MIR3134					
cg26087577	MIR3134					
cg25884222	MIR3134					
cg07426997	MIR3134					
cg05226457	MIR3134					
cg19950186	MIR3134					
cg11925789	MIR3134					
cg04054359	MIR3134					
cg27428462	MIR3134					
cg08474649	MIR3134					
cg14040871	MIR455					
cg14004457	MIR455					
cg23936341	MIR455					
cg18717689	MIR455					
cg10504741	MIR455					
cg14766769	MIR600					
cg13930811	MIR600					
cg14431734	MIR600					
cg13563462	MIR600					
cg00424588	MIR601					
cg14166365	MIR601					
cg01567328	MIR601					
cg00978117	MIR601					
cg12954102	MIR181A2HG					
cg06321925	MIR181A2HG					
cg12286351	MIR181A2HG					
cg05106358	MIR181A2HG					
cg04042162	MIR181A2HG					
cg09595054	MIR181A2HG					
cg17713013	MIR181A2HG					
cg27342371	MIR181A2HG					

cg19122747	MIR181A2	MIR181A2HG				
cg01246632	MIR181A2	MIR181B2				
cg07909384	MIR181B2	MIR181A2HG				
cg16849649	MIR181A2HG					
cg17639867	MIR3911					
cg09105685	MIR3911					
cg17615262	MIR3960	MIR2861				
cg08999352	MIR3960	MIR2861				
cg27070458	MIR3960	MIR2861				
cg22783100	MIR2861	MIR3960				
cg17017819	MIR2861					
cg07292215	MIR2861					
cg27343747	MIR2861					
cg14723090	MIR3154	MIR199B				
cg03959541	MIR199B	MIR3154				
cg06953499	MIR199B	MIR3154				
cg13718827	MIR199B					
cg17153540	MIR1268A					
cg16481138	MIR1268A					
cg08445157	MIR1268A					
cg24225917	MIR1268A					
cg01916700	MIR1268A					
cg11418490	MIR1268A					
cg21824858	MIR1268A					
cg11100685	MIR1268A					
cg15010497	MIR1268A					
cg15068681	MIR1268A					
cg26802390	MIR1268A					
cg04710273	MIR1268A					
cg26179116	MIR1268A					
cg22873268	MIR1268A					
cg15167810	MIR1268A					
cg10028175	MIR1268A					
cg04353365	MIR1268A					
cg18745642	MIR219B	MIR1268A				
cg20451272	MIR219A2	MIR1268A				
cg04235540	MIR219A2	MIR1268A				
cg10494844	MIR1268A					
cg17016635	MIR1268A					
cg02748477	MIR1268A					
cg11441832	MIR1268A					
cg11338041	MIR1268A					
cg06710212	MIR1268A					
cg20487632	MIR1268A					
cg12765908	MIR1268A					
cg18870599	MIR1268A					
cg07363202	MIR1268A					
cg10232005	MIR1268A					
cg10461164	MIR1268A					
cg13157805	MIR1268A					

cg23593672	MIR1268A					
cg12168488	MIR1268A					
cg09986564	MIR1268A					
cg11465913	MIR1268A					
cg15509000	MIR1268A					
cg23806774	MIR1268A					
cg04580583	MIR1268A					
cg23262272	MIR1268A					
cg13311392	MIR1268A					
cg16702144	MIR1268A					
cg01447389	MIR1268A					
cg10481470	MIR1268A					
cg11050908	MIR1268A					
cg21792562	MIR1268A					
cg24529616	MIR1268A					
cg20562143	MIR1268A					
cg02027349	MIR1268A					
cg02067741	MIR1268A					
cg14953687	MIR1268A					
cg07381231	MIR1268A					
cg08193376	MIR1268A					
cg26753781	MIR1268A					
cg26789412	MIR1268A					
cg23741702	MIR1268A					
cg09661924	MIR1268A					
cg05965535	MIR1268A					
cg24942039	MIR1268A					
cg05594766	MIR1268A					
cg05733895	MIR1268A					
cg19775372	MIR1268A					
cg23815565	MIR1268A					
cg23642110	MIR1268A					
cg19061781	MIR1268A					
cg06518008	MIR1268A					
cg05483851	MIR1268A					
cg26580968	MIR1268A					
cg22545525	MIR1268A					
cg24346519	MIR1268A					
cg25333889	MIR1268A					
cg01850295	MIR1268A					
cg03678049	MIR1268A					
cg02903877	MIR1268A					
cg17879823	MIR1268A					
cg21829017	MIR1268A					
cg04370758	MIR1268A					
cg16013896	MIR1268A					
cg01242189	MIR1268A					
cg01662894	MIR1268A					
cg22912535	MIR1268A					
cg07140093	MIR1268A					

cg01784331	MIR1268A					
cg11065747	MIR1268A					
cg12970946	MIR1268A					
cg11851976	MIR1268A					
cg05581661	MIR1268A					
cg05072637	MIR1268A					
cg01309597	MIR1268A					
cg23590542	MIR1268A					
cg17388916	MIR1268A					
cg16756504	MIR1268A					
cg18360270	MIR1268A					
cg01206174	MIR1268A					
cg24188030	MIR1268A					
cg07396237	MIR1268A					
cg15821414	MIR1268A					
cg04772569	MIR1268A					
cg02408313	MIR1268A					
cg23847029	MIR1268A					
cg03635774	MIR1268A					
cg02566189	MIR1268A					
cg11210410	MIR1268A					
cg12306793	MIR1268A					
cg01194567	MIR1268A					
cg02273999	MIR1268A					
cg03196845	MIR1268A					
cg03269638	MIR6855					
cg03131516	MIR6856					
cg11295002	MIR548AW					
cg12207024	MIR548AW					
cg00425865	MIR548AW					
cg12146158	MIR548AW					
cg05233902	MIR548AW					
cg27495603	MIR548AW					
cg20528695	MIR6877					
cg06701473	MIR6877					
cg02740487	MIR4669					
cg09526255	MIR4669					
cg09227887	MIR4669					
cg01894436	MIR4669					
cg21507303	MIR3689B	MIR3689D2	MIR3689E	MIR3689A	MIR3689F	
cg13304430	MIR4673					
cg11626619	MIR4674					
cg04271687	MIR4674					
cg12328140	MIR4674					
cg00501622	MIR126					
cg09260946	MIR126					
cg23302214	MIR126					
cg16903347	MIR126					
cg14435720	MIR126					
cg17485016	MIR4292					

cg22624321	MIR4479					
cg17292885	MIR4479					
cg07459895	MIR3621					
cg18576861	MIR3621					
cg13224090	MIR3621					
cg06292259	MIR3621					
cg10281361	MIR3621					
cg12326440	MIR7641-2					
cg21105876	MIR7641-2					
cg08653328	MIR7641-2					
cg19760211	MIR7641-2					
cg15905865	MIR7641-2					
cg02458318	MIR5699					
cg04390575	MIR3155A					
cg27116149	MIR3155A					
cg10537327	MIR3155A					
cg11629527	MIR3155B					
cg21348752	MIR1915					
cg27244585	MIR1915					
cg03611732	MIR1915					
cg25726414	MIR1915					
cg05088677	MIR1915					
cg06760536	MIR1915					
cg12362662	MIR1915					
cg05387457	MIR1915					
cg04686790	MIR1915					
cg11165626	MIR1915					
cg26945813	MIR1915					
cg26213561	MIR1915					
cg04153571	MIR1915					
cg00282704	MIR1915					
cg24823751	MIR1915					
cg05341330	MIR1915					
cg01815730	MIR603					
cg07158816	MIR603					
cg01283398	MIR603					
cg19478638	MIR604					
cg05559808	MIR604					
cg12329853	MIR604					
cg09916797	MIR604					
cg19767857	MIR938					
cg00064589	MIR938					
cg00622375	MIR938					
cg15856028	MIR938					
cg24439831	MIR3611					
cg25060796	MIR4683					
cg11478495	MIR605					
cg02887712	MIR605					
cg15437223	MIR1296					
cg16324767	MIR1296					

cg00022938	MIR1296					
cg21524784	MIR1296					
cg02790122	MIR1296					
cg16276613	MIR7152					
cg02194384	MIR7152					
cg07538027	MIR7152					
cg13992202	MIR7152					
cg15279236	MIR1256					
cg06348245	MIR1256					
cg02501544	MIR1256					
cg27242911	MIR1256					
cg16617349	MIR1256					
cg02632490	MIR346					
cg25972714	MIR346					
cg16692757	MIR346					
cg16570507	MIR346					
cg23322812	MIR346					
cg12757181	MIR346					
cg01856509	MIR346					
cg26271970	MIR346					
cg17210014	MIR346					
cg24497344	MIR4678					
cg23477281	MIR4678					
cg21562001	MIR4678					
cg17343879	MIR107					
cg03948048	MIR107					
cg24445034	MIR107					
cg17856907	MIR107					
cg10581650	MIR1287					
cg01375259	MIR1287					
cg23751416	MIR1287					
cg26882429	MIR1287					
cg12469964	MIR1287					
cg09443153	MIR1287					
cg26241514	MIR608					
cg02393496	MIR608					
cg25979474	MIR608					
cg10949319	MIR1307					
cg12798274	MIR1307					
cg06102342	MIR1307					
cg20502234	MIR1307					
cg25574153	MIR936					
cg25717590	MIR936					
cg21688999	MIR936					
cg17384806	MIR936					
cg04205369	MIR936					
cg23130731	MIR609					
cg03775802	MIR609					
cg25414746	MIR609					
cg08540829	MIR609					

cg06773798	MIR609					
cg25588914	MIR4482					
cg16889524	MIR6715A					
cg06031160	MIR6715B					
cg24201716	MIR2110					
cg15372479	MIR2110					
cg09632185	MIR2110					
cg23206103	MIR2110					
cg18864164	MIR2110					
cg00568128	MIR2110					
cg25451306	MIR2110					
cg17741865	MIR2110					
cg01390168	MIR2110					
cg24012440	MIR2110					
cg26301661	MIR2110					
cg25135143	MIR2110					
cg13565543	MIR2110					
cg23426816	MIR2110					
cg03719236	MIR2110					
cg03115097	MIR2110					
cg13555855	MIR3663HG	MIR3663				
cg27102904	MIR3663	MIR3663HG				
cg26986226	MIR3663	MIR3663HG				
cg06722483	MIR3663	MIR3663HG				
cg26042267	MIR3663HG					
cg20893537	MIR4681					
cg17636008	MIR4681					
cg14141340	MIR5694					
cg05678252	MIR5694					
cg04681122	MIR5694					
cg10467906	MIR5694					
cg14907703	MIR5694					
cg13666122	MIR5694					
cg24579698	MIR5694					
cg15809032	MIR5694					
cg01502979	MIR5694					
cg17625407	MIR5694					
cg07991335	MIR5694					
cg25194512	MIR5694					
cg14634569	MIR5694					
cg18607338	MIR5694					
cg13596132	MIR5694					
cg13207036	MIR5694					
cg12382424	MIR5694					
cg21623395	MIR5694					
cg13151449	MIR5694					
cg03062642	MIR5694					
cg20246901	MIR5694					
cg13182610	MIR202	MIR202HG				
cg15686115	MIR202	MIR202HG				

cg16892611	MIR202	MIR202HG				
cg01825707	MIR6743					
cg14858991	MIR210HG	MIR210				
cg19916545	MIR210	MIR210HG				
cg14063402	MIR7847					
cg26663525	MIR7847					
cg15269875	MIR675					
cg14937069	MIR675					
cg19943238	MIR675					
cg03175030	MIR675					
cg18511798	MIR675					
cg21167159	MIR675					
cg16153294	MIR675					
cg15317267	MIR675					
cg22424892	MIR675					
cg05448231	MIR675					
cg07342901	MIR675					
cg11753499	MIR675					
cg13210239	MIR675					
cg17985533	MIR675					
cg15922305	MIR675					
cg09701145	MIR675					
cg25437674	MIR675					
cg06029905	MIR483					
cg02808220	MIR483					
cg24183187	MIR483					
cg22932993	MIR483					
cg21728792	MIR483					
cg14890224	MIR483					
cg01668279	MIR483					
cg12158055	MIR4686					
cg26253759	MIR4686					
cg15489575	MIR4687					
cg22824552	MIR4687					
cg06149971	MIR6124					
cg21362233	MIR6124					
cg16732457	MIR610					
cg12517288	MIR1343					
cg19536085	MIR1343					
cg09196809	MIR1343					
cg13559712	MIR1343					
cg17877110	MIR3973					
cg13202936	MIR4688					
cg03832078	MIR4688					
cg13098091	MIR4688					
cg12580820	MIR3160-2					
cg17680641	MIR4488					
cg20176573	MIR4488					
cg05046026	MIR611					
cg25628257	MIR611					

cg26061001	MIR611					
cg16150798	MIR611					
cg02005336	MIR611					
cg22704775	MIR611					
cg10248302	MIR611					
cg18009798	MIR611					
cg11730100	MIR611					
cg03114244	MIR611					
cg07583651	MIR611					
cg16916522	MIR611					
cg08873353	MIR611					
cg22662482	MIR611					
cg18799266	MIR611					
cg03805684	MIR611					
cg25401284	MIR1908					
cg14725641	MIR1908					
cg03735013	MIR1908					
cg25326896	MIR1908					
cg12517394	MIR1908					
cg09677638	MIR1908					
cg15598662	MIR1908					
cg00786201	MIR1908					
cg24870774	MIR1908					
cg07152460	MIR1908					
cg13475388	MIR1908					
cg13100764	MIR1908					
cg24636220	MIR1908					
cg05623655	MIR1908					
cg11229063	MIR1908					
cg03921599	MIR1908					
cg09782344	MIR1908					
cg20295071	MIR1908					
cg23992449	MIR1908					
cg27173322	MIR1908					
cg11985562	MIR1908					
cg13282180	MIR6746					
cg08460710	MIR6746					
cg05322090	MIR6748					
cg08224477	MIR6748					
cg20798394	MIR7155					
cg00861316	MIR1237					
cg02705752	MIR1237					
cg12778580	MIR1237					
cg02675260	MIR1237					
cg13037081	MIR1237					
cg27163061	MIR1237					
cg20699960	MIR1237					
cg04024822	MIR1237					
cg22788485	MIR1237					
cg25770702	MIR1237					

cg22831978	MIR1237					
cg24803202	MIR194-2	MIR192				
cg08432452	MIR192	MIR194-2				
cg00589493	MIR192	MIR194-2				
cg13092487	MIR192	MIR194-2				
cg24154336	MIR192	MIR194-2				
cg00400165	MIR192	MIR194-2				
cg00376448	MIR192	MIR194-2				
cg02494703	MIR194-2	MIR192				
cg06104877	MIR194-2	MIR192				
cg18715874	MIR6749					
cg21463067	MIR6879					
cg07438546	MIR6751					
cg06103064	MIR6751					
cg08793239	MIR612					
cg15595954	MIR612					
cg10090749	MIR612					
cg22040449	MIR1234					
cg14906130	MIR3163					
cg12556644	MIR3163					
cg24823998	MIR6860					
cg24286190	MIR6752					
cg22862808	MIR6752					
cg07099752	MIR6752					
cg26703857	MIR7113					
cg12293583	MIR4691					
cg16249681	MIR4691					
cg24213572	MIR4691					
cg06075793	MIR4691					
cg26859772	MIR6753					
cg23904570	MIR6753					
cg13965908	MIR548K					
cg16020638	MIR548K					
cg10548355	MIR548K					
cg02797411	MIR3664					
cg10051714	MIR139					
cg00804634	MIR139					
cg22123156	MIR139					
cg19245310	MIR139					
cg06466538	MIR4692					
cg07588932	MIR4692					
cg08377894	MIR4692					
cg08536044	MIR4696					
cg25899829	MIR326					
cg18821418	MIR326					
cg22969079	MIR326					
cg17074821	MIR326					
cg03932760	MIR326					
cg16137891	MIR326					
cg08960830	MIR326					

cg05473648	MIR708					
cg25837979	MIR708					
cg17233829	MIR708					
cg04726985	MIR708					
cg02397217	MIR6755					
cg07107321	MIR3166					
cg08337765	MIR3166					
cg16111645	MIR3166					
cg11050977	MIR1261					
cg17650615	MIR1261					
cg00530918	MIR1304					
cg01580228	MIR1304					
cg21011320	MIR1304					
cg27164575	MIR548L					
cg21173689	MIR548L					
cg05401712	MIR548L					
cg11617306	MIR548L					
cg03242880	MIR34C	MIR34B				
cg22806002	MIR34C	MIR34B				
cg18515591	MIR34C	MIR34B				
cg22879515	MIR34B	MIR34C				
cg21881253	MIR34B	MIR34C				
cg13767940	MIR34B	MIR34C				
cg26561785	MIR34B	MIR34C				
cg01192900	MIR34B	MIR34C				
cg24041078	MIR34C					
cg08827001	MIR34C					
cg05281603	MIR34C					
cg09148270	MIR34C					
cg12108074	MIR6716					
cg09505361	MIR6716					
cg18217031	MIR4492					
cg20342998	MIR4492					
cg02208900	MIR3656					
cg27124567	MIR3656					
cg12547459	MIR3656					
cg01768115	MIR3656					
cg27523854	MIR3656					
cg07762927	MIR3656					
cg11282724	MIR3656					
cg03558921	MIR6756					
cg16865908	MIR125B1					
cg26916936	MIR125B1					
cg02101355	MIR125B1					
cg03891346	MIR125B1					
cg24213115	MIR125B1					
cg07281370	MIR125B1					
cg24150623	MIR125B1					
cg06749053	MIR125B1					
cg20475322	MIR125B1					

cg24603444	MIR125B1					
cg01837269	MIR125B1	MIR100HG				
cg07685357	MIR125B1					
cg11795976	MIR100HG					
cg19888881	MIR100HG					
cg03649143	MIRLET7A2					
cg06811361	MIRLET7A2					
cg18590130	MIRLET7A2					
cg19116912	MIR100					
cg17726105	MIR100					
cg24655701	MIR100					
cg11613644	MIR7641-2					
cg10789610	MIR7641-2					
cg17425265	MIR7641-2					
cg20296844	MIR7641-2					
cg23827862	MIR3167					
cg17793749	MIR4697HG	MIR4697				
cg24702147	MIR141	MIR200C				
cg20979075	MIR200C	MIR141				
cg00366413	MIR141	MIR200C				
cg27534624	MIR141	MIR200C				
cg18959988	MIR141	MIR200C				
cg02753020	MIR1244-4	MIR1244-2	MIR1244-3	MIR1244-1		
cg13786932	MIR1244-4	MIR1244-2	MIR1244-3	MIR1244-1		
cg08411094	MIR613					
cg11297610	MIR613					
cg08698035	MIR613					
cg05737842	MIR613					
cg10564060	MIR7641-2					
cg06771291	MIR920					
cg13277047	MIR920					
cg21663427	MIR7851					
cg06677980	MIR1975					
cg13488877	MIR1975					
cg23018181	MIR1975					
cg02864240	MIR1975					
cg09300189	MIR1291					
cg20984590	MIR1291					
cg18838148	MIR1293					
cg02911529	MIR6757					
cg10561239	MIR615					
cg09137135	MIR148B					
cg13827287	MIR148B					
cg00240007	MIR148B					
cg16784745	MIR148B					
cg24219985	MIR1228					
cg10989175	MIR1228					
cg16521032	MIR1228					
cg05776138	MIR1228					
cg21913159	MIR1228					

cg03668470	MIR1228					
cg13038544	MIR1228					
cg07124859	MIR1228					
cg21702971	MIR1228					
cg11122256	MIR1228					
cg11313099	MIR6758					
cg07617764	MIR26A2					
cg01190168	MIR26A2					
cg15851964	MIR26A2					
cg17169243	MIR26A2					
cg27606822	MIR26A2					
cg09263904	MIR26A2					
cg02115865	MIR6125					
cg23911696	MIR6125					
cg01149144	MIR6125					
cg24789224	MIR6125					
cg19835331	MIR6125					
cg09612552	MIR6125					
cg06162516	MIRLET7I					
cg23174344	MIRLET7I					
cg12023318	MIRLET7I					
cg21544377	MIRLET7I					
cg00874357	MIRLET7I					
cg05102308	MIRLET7I					
cg14108394	MIRLET7I					
cg25084332	MIRLET7I					
cg17099076	MIRLET7I					
cg21883042	MIRLET7I					
cg19484886	MIRLET7I					
cg23118562	MIRLET7I					
cg16098340	MIR548C					
cg11782684	MIR548C					
cg20351875	MIR548C					
cg26305717	MIR548C					
cg12332613	MIR548C					
cg19629843	MIR1279					
cg09177306	MIR3913-2					
cg19808663	MIR3913-1					
cg14166992	MIR3913-1					
cg18262805	MIR3913-1					
cg02778009	MIR3913-1					
cg16544548	MIR3913-1					
cg22268341	MIR1252					
cg08668140	MIR1252					
cg24233224	MIR1252					
cg13320585	MIR1252					
cg24437010	MIR617					
cg03742132	MIR617					
cg08595690	MIR617					
cg02387491	MIR617					

cg02554051	MIR618					
cg22986737	MIR618					
cg08784247	MIR618					
cg00323458	MIR618					
cg02585417	MIR618					
cg05348982	MIR618					
cg09876651	MIR618					
cg00256294	MIR548AL					
cg03444423	MIR7844					
cg17967602	MIR1251					
cg27533635	MIR1251					
cg00030523	MIR1251					
cg19702383	MIR1251					
cg25492056	MIR1251					
cg04663692	MIR1251					
cg10243781	MIR3652					
cg26395896	MIR3652					
cg01019512	MIR3652					
cg17203920	MIR3652					
cg11025641	MIR3652					
cg02083433	MIR7641-2					
cg22963317	MIR7641-2					
cg09791785	MIR7641-2					
cg10721066	MIR4496					
cg13449394	MIR619					
cg07225475	MIR619					
cg24871226	MIR619					
cg25720803	MIR619					
cg00608334	MIR4497					
cg19061142	MIR4497					
cg18176057	MIR7106					
cg12573843	MIR7106					
cg18087803	MIR620					
cg09230679	MIR620					
cg13017634	MIR620					
cg19685229	MIR620					
cg12704462	MIR1178					
cg18805612	MIR1178					
cg21913981	MIR1178					
cg00009001	MIR1178					
cg19844829	MIR1178					
cg18144866	MIR4304					
cg17120358	MIR6880					
cg24826413	MIR6880					
cg20337110	MIR6880					
cg14026942	MIR6880					
cg23491508	MIR5188					
cg26749351	MIR5188					
cg00379002	MIR5188					
cg08837158	MIR5188					

cg18479875	MIR5188					
cg10298988	MIR5188					
cg04781016	MIR5188					
cg03159456	MIR5188					
cg17963042	MIR4499					
cg05047276	MIR4499					
cg07160044	MIR2276					
cg22982536	MIR2276					
cg00967013	MIR2276					
cg09180566	MIR2276					
cg00861695	MIR2276					
cg15042891	MIR2276					
cg07152812	MIR2276					
cg22412649	MIR2276					
cg10587183	MIR548F5					
cg21392700	MIR548F5					
cg03356172	MIR548F5					
cg13238479	MIR548F5					
cg00505001	MIR548F5					
cg16877681	MIR548F5					
cg12178237	MIR548F5					
cg15259593	MIR548F5					
cg15561647	MIR548F5					
cg05093686	MIR548F5					
cg21884062	MIR548F5					
cg01952313	MIR548F5					
cg12573705	MIR548F5					
cg03014934	MIR548F5					
cg22855860	MIR548F5					
cg12029639	MIR548F5					
cg13446906	MIR548F5					
cg25618572	MIR548F5					
cg24769969	MIR548F5					
cg07554357	MIR548F5					
cg14408605	MIR548F5					
cg02558362	MIR548F5					
cg20704442	MIR548F5					
cg04916802	MIR548F5					
cg02229135	MIR548F5					
cg05446322	MIR548F5					
cg22037492	MIR548F5					
cg21364568	MIR548F5					
cg16510860	MIR548F5					
cg22182936	MIR548F5					
cg13003178	MIR548F5					
cg19947060	MIR548F5					
cg25192925	MIR548F5					
cg08520986	MIR548F5					
cg18830373	MIR548F5					
cg26700590	MIR548F5					

cg03640487	MIR548F5					
cg05623392	MIR548F5					
cg12062668	MIR548F5					
cg03471320	MIR548F5					
cg13652338	MIR548F5					
cg11657317	MIR548F5					
cg04476865	MIR548F5					
cg13027104	MIR548F5					
cg15421870	MIR548F5					
cg06641651	MIR548F5					
cg07617482	MIR548F5					
cg10564626	MIR548F5					
cg21100351	MIR548F5					
cg08981212	MIR548F5					
cg13953455	MIR548F5					
cg15535534	MIR548F5					
cg01397463	MIR548F5					
cg12329484	MIR548F5					
cg02201491	MIR548F5					
cg06118206	MIR548F5					
cg12140530	MIR548F5					
cg26995690	MIR548F5					
cg17205233	MIR548F5					
cg04541021	MIR548F5					
cg01640572	MIR548F5					
cg08031206	MIR548F5					
cg19552794	MIR548F5					
cg21880213	MIR548F5					
cg09355500	MIR548F5					
cg05107190	MIR548F5					
cg07249860	MIR548F5					
cg02298831	MIR548F5					
cg15207708	MIR548F5					
cg21629505	MIR548F5					
cg19350059	MIR548F5					
cg23814365	MIR548F5					
cg12942606	MIR548F5					
cg21092373	MIR548F5					
cg12206359	MIR548F5					
cg08766508	MIR548F5					
cg10623685	MIR548F5					
cg13669476	MIR548F5					
cg19409254	MIR548F5					
cg04415610	MIR548F5					
cg17674402	MIR548F5					
cg16725262	MIR548F5					
cg19353642	MIR548F5					
cg24558576	MIR548F5					
cg12646800	MIR548F5					
cg01719718	MIR548F5					

cg03565833	MIR548F5					
cg01929699	MIR548F5					
cg16621560	MIR548F5					
cg25874148	MIR548F5					
cg00269670	MIR548F5					
cg01409434	MIR548F5					
cg14247325	MIR548F5					
cg21504345	MIR548F5					
cg05757376	MIR548F5					
cg09532726	MIR548F5					
cg05361272	MIR621					
cg00363066	MIR621					
cg24331563	MIR5006					
cg14791048	MIR3613					
cg20988440	MIR16-1	MIR15A				
cg18526167	MIR16-1	MIR15A				
cg21683783	MIR15A					
cg20116420	MIR3665					
cg07380755	MIR3665					
cg06616674	MIR3665					
cg22476237	MIR3665					
cg08728669	MIR4500	MIR4500HG				
cg25139619	MIR17HG					
cg09732987	MIR17HG					
cg25308542	MIR17HG					
cg01068014	MIR17HG					
cg17926458	MIR17HG					
cg08604905	MIR17HG	MIR17				
cg19629686	MIR17HG	MIR17	MIR18A			
cg07235355	MIR19A	MIR17HG	MIR17	MIR18A		
cg17799287	MIR19A	MIR17HG	MIR17	MIR18A		
cg07641807	MIR19A	MIR17HG	MIR20A	MIR17	MIR19B1	MIR18A
cg02297838	MIR19A	MIR17HG	MIR92A1	MIR20A	MIR17	MIR19B1
cg19723657	MIR17HG	MIR20A	MIR19B1	MIR92A1	MIR18A	MIR19A
cg04908961	MIR17HG	MIR92A1	MIR20A	MIR19B1		
cg10740663	MIR17HG	MIR19B1	MIR92A1			
cg15179595	MIR92A1	MIR17HG	MIR19B1			
cg07418289	MIR17HG					
cg22409939	MIR548AN					
cg16346169	MIR548AN					
cg24521382	MIR548AN					
cg02140096	MIR548AN					
cg16154454	MIR548AN					
cg21043695	MIR548AN					
cg08172426	MIR548AN					
cg06164122	MIR548AN					
cg09379151	MIR548AN					
cg04430472	MIR548AN					
cg19567023	MIR548AN					
cg06215240	MIR548AN					

cg11857748	MIR548AN					
cg15562377	MIR548AN					
cg12538845	MIR548AN					
cg11292807	MIR548AN					
cg27111395	MIR548AN					
cg25536196	MIR548AN					
cg01855585	MIR548AN					
cg12507840	MIR548AN					
cg09114848	MIR548AN					
cg16178786	MIR548AN					
cg04627415	MIR548AN					
cg00033818	MIR548AN					
cg01772760	MIR548AN					
cg12585342	MIR548AN					
cg08218990	MIR548AN					
cg21049840	MIR548AN					
cg11982292	MIR548AN					
cg23333994	MIR548AN					
cg20405620	MIR548AN					
cg12382480	MIR548AN					
cg23363242	MIR548AN					
cg23821020	MIR548AN					
cg04257196	MIR548AN					
cg18480071	MIR548AN					
cg16864411	MIR548AN					
cg05394484	MIR548AN					
cg17574593	MIR548AN					
cg18714170	MIR548AN					
cg24856024	MIR548AN					
cg17518643	MIR548AN					
cg04036282	MIR548AN					
cg06338239	MIR548AN					
cg21317441	MIR548AN					
cg10145411	MIR548AN					
cg06199909	MIR548AN					
cg15353530	MIR548AN					
cg13885053	MIR548AN					
cg02013168	MIR548AN					
cg17148213	MIR548AN					
cg09561830	MIR623					
cg19635644	MIR623					
cg02702515	MIR623					
cg18560638	MIR623					
cg16193278	MIR623					
cg24330404	MIR548AN					
cg07424038	MIR548AN					
cg03156020	MIR548AN					
cg12413643	MIR548AN					
cg16261025	MIR548AN					
cg19890584	MIR548AN					

cg23872155	MIR548AN					
cg14015828	MIR548AN					
cg11477273	MIR548AN					
cg26532527	MIR548AN					
cg22719998	MIR548AN					
cg23109066	MIR548AN					
cg17549097	MIR548AN					
cg04721126	MIR548AN					
cg14765087	MIR2681					
cg25549968	MIR1267					
cg07532986	MIR1267					
cg03542468	MIR1267					
cg13130979	MIR1267					
cg19233792	MIR548AR					
cg18729186	MIR1201					
cg10336220	MIR6717					
cg07589927	MIR6717					
cg19576080	MIR6717					
cg09335599	MIR6717					
cg18020268	MIR6717					
cg15094224	MIR6717					
cg15703728	MIR6717					
cg21994794	MIR6717					
cg04594986	MIR6717					
cg10325896	MIR6717					
cg02839951	MIR6717					
cg17050183	MIR4707					
cg27126814	MIR4707					
cg04573702	MIR208A					
cg21828716	MIR208A					
cg05241732	MIR208A					
cg25428233	MIR208B					
cg13785779	MIR208B					
cg21242212	MIR208B					
cg03918703	MIR208B					
cg14030388	MIR208B					
cg02658046	MIR7703					
cg20871143	MIR7703					
cg08588953	MIR3171					
cg24939330	MIR3171					
cg17651653	MIR548AI					
cg25408758	MIR548AI					
cg21449177	MIR548AI					
cg22451117	MIR548AI					
cg27503740	MIR548AI					
cg11597887	MIR548AI					
cg05709325	MIR548AI					
cg12070337	MIR548AI					
cg10103520	MIR548AI					
cg08134221	MIR548AI					

cg13676849	MIR548AI					
cg08274139	MIR624					
cg10195619	MIR624					
cg19500057	MIR624					
cg06490299	MIR4503					
cg20126937	MIR548AZ					
cg00561206	MIR548AZ					
cg17424516	MIR548AZ					
cg19081329	MIR548AZ					
cg21790677	MIR548AZ					
cg01230180	MIR548AZ					
cg02114711	MIR548AZ					
cg21677210	MIR548AZ					
cg04631545	MIR548AZ					
cg23254057	MIR548AZ					
cg24118155	MIR548AZ					
cg00356134	MIR548AZ					
cg27116069	MIR548AZ					
cg11701550	MIR548AZ					
cg05365942	MIR548AZ					
cg10921624	MIR548AZ					
cg10460086	MIR548AZ					
cg14133042	MIR548AZ					
cg02523350	MIR548AZ					
cg05580240	MIR548H1	MIR548AZ				
cg12367585	MIR548H1	MIR548AZ				
cg08718220	MIR548AZ					
cg16348475	MIR548AZ					
cg04893124	MIR548AZ					
cg11730290	MIR548AZ					
cg04590632	MIR548AZ					
cg22475368	MIR548AZ					
cg16083780	MIR548AZ					
cg22708188	MIR548AZ					
cg17649996	MIR548AZ					
cg06593686	MIR548AZ					
cg15422361	MIR548AZ					
cg27170797	MIR548AZ					
cg09523808	MIR548AZ					
cg09948795	MIR548AZ					
cg04933432	MIR548AZ					
cg04615725	MIR548AZ					
cg09663027	MIR548AZ					
cg16057182	MIR548AZ					
cg15773296	MIR548AZ					
cg01257685	MIR548AZ					
cg25763735	MIR548AZ					
cg06284898	MIR548AZ					
cg20894023	MIR548AZ					
cg02322229	MIR548AZ					

cg10181798	MIR548AZ					
cg00884188	MIR548AZ					
cg25144602	MIR548AZ					
cg10363208	MIR548AZ					
cg07923716	MIR548AZ					
cg14230816	MIR548AZ					
cg15745972	MIR548AZ					
cg21615907	MIR548AZ					
cg06653974	MIR548AZ					
cg00579734	MIR548AZ					
cg21647636	MIR548AZ					
cg04823958	MIR548AZ					
cg12411817	MIR548AZ					
cg24025859	MIR548AZ					
cg16185223	MIR548AZ					
cg05185154	MIR548AZ					
cg07591882	MIR548AZ					
cg14417226	MIR548AZ					
cg16964791	MIR548AZ					
cg19324608	MIR548AZ					
cg26779688	MIR548AZ					
cg02027685	MIR548AZ					
cg09764150	MIR548AZ					
cg20488765	MIR548AZ					
cg12798649	MIR548AZ					
cg18730515	MIR548AZ					
cg06154883	MIR548AZ					
cg07355632	MIR548AZ					
cg21434113	MIR548AZ					
cg20793562	MIR548AZ					
cg00453774	MIR548AZ					
cg14980467	MIR548AZ					
cg10799802	MIR548AZ					
cg03916245	MIR548AZ					
cg15543621	MIR548AZ					
cg03859739	MIR548AZ					
cg15746029	MIR548AZ					
cg08332815	MIR548AZ					
cg26535023	MIR548AZ					
cg17224504	MIR548AZ					
cg17112491	MIR548AZ					
cg25966852	MIR548AZ					
cg06239513	MIR548AZ					
cg14016128	MIR548AZ					
cg17775912	MIR548AZ					
cg26748435	MIR548AZ					
cg14111726	MIR548AZ					
cg27267322	MIR548AZ					
cg02284419	MIR548AZ					
cg09297676	MIR548AZ					

cg08635913	MIR548AZ					
cg24438311	MIR548AZ					
cg09241708	MIR548AZ					
cg09099968	MIR548AZ					
cg23375912	MIR548AZ					
cg07968094	MIR548AZ					
cg11763745	MIR548AZ					
cg12414009	MIR548AZ					
cg01846028	MIR548AZ					
cg00407815	MIR548AZ					
cg08253253	MIR548AZ					
cg19225438	MIR548AZ					
cg27386631	MIR548AZ					
cg11658051	MIR548AZ					
cg09077612	MIR548AZ					
cg27513510	MIR548AZ					
cg03357215	MIR548AZ					
cg14325848	MIR548AZ					
cg05623247	MIR548AZ					
cg09666525	MIR548AZ					
cg16192458	MIR4706					
cg16424038	MIR625					
cg09934721	MIR625					
cg25845814	MIR4505					
cg14257776	MIR7641-2					
cg23674379	MIR7641-2					
cg18421195	MIR7641-2					
cg10993988	MIR7641-2					
cg24006305	MIR1260					
cg06883544	MIR1260					
cg25952096	MIR1260					
cg10134156	MIR1260					
cg21095982	MIR1260					
cg07843309	MIR3173					
cg19929625	MIR342					
cg12264462	MIR342					
cg13622660	MIR151B	MIR342				
cg17191445	MIR342	MIR151B				
cg26671937	MIR342	MIR151B				
cg15358487	MIR151B					
cg09552714	MIR6764					
cg17002091	MIR345					
cg18986330	MIR345					
cg11840833	MIR345					
cg08694786	MIR345					
cg00527484	MIR345					
cg27569588	MIR345					
cg06910115	MIR345					
cg00912722	MIR345					
cg10527285	MIR345					

cg26157803	MIR345					
cg02540736	MIR345					
cg10597322	MIR345					
cg06846458	MIR345					
cg18186343	MIR770					
cg03039990	MIR770					
cg01022345	MIR770					
cg19509303	MIR770					
cg24044238	MIR665	MIR337				
cg09565745	MIR337	MIR665				
cg18485627	MIR665	MIR337				
cg00594101	MIR665	MIR337				
cg20526085	MIR431	MIR433				
cg02022380	MIR431	MIR433				
cg06165706	MIR431	MIR433				
cg18340059	MIR431	MIR433				
cg23546343	MIR431	MIR433				
cg15516314	MIR431	MIR433				
cg07087646	MIR431	MIR433				
cg08244382	MIR127	MIR433				
cg13662173	MIR127	MIR433				
cg08175935	MIR127	MIR433				
cg02135795	MIR433	MIR127				
cg24721750	MIR127					
cg13555689	MIR127					
cg26976046	MIR127					
cg05899507	MIR127					
cg21103400	MIR127					
cg21217911	MIR127					
cg10726559	MIR127					
cg25063505	MIR127					
cg18862502	MIR127					
cg11205335	MIR432	MIR127				
cg14937446	MIR432					
cg06792448	MIR432	MIR136				
cg02675985	MIR432	MIR136				
cg19603885	MIR432	MIR136				
cg27267198	MIR432	MIR136				
cg05371620	MIR432	MIR136				
cg18141891	MIR136	MIR432				
cg23377000	MIR136	MIR432				
cg23682215	MIR136	MIR432				
cg26254637	MIR136	MIR432				
cg18947995	MIR136	MIR432				
cg02344891	MIR136	MIR432				
cg02524808	MIR136					
cg04699566	MIR136					
cg14410227	MIR411	MIR379				
cg18440069	MIR411	MIR379				
cg13278105	MIR411	MIR379				

cg20067612	MIR299	MIR411				
cg00989505	MIR299	MIR411				
cg18384960	MIR299	MIR411				
cg21876806	MIR299	MIR411				
cg01132653	MIR299	MIR411				
cg02135464	MIR299	MIR411				
cg19603100	MIR299	MIR380				
cg25069907	MIR299	MIR380				
cg23492249	MIR299	MIR380				
cg10969550	MIR299	MIR380				
cg10036091	MIR299	MIR380				
cg18484488	MIR299	MIR380				
cg03556771	MIR299	MIR380				
cg10082525	MIR380	MIR299				
cg00175487	MIR380	MIR299				
cg25562958	MIR1197	MIR380	MIR323			
cg09243104	MIR380	MIR1197	MIR323A	MIR758		
cg23310549	MIR758	MIR1197	MIR323	MIR380		
cg12820006	MIR758	MIR1197	MIR323			
cg26015115	MIR758	MIR1197	MIR329-1	MIR323		
cg06549228	MIR758	MIR1197	MIR329-1	MIR323		
cg21501241	MIR758	MIR1197	MIR329-1	MIR323		
cg16575409	MIR758	MIR1197	MIR329-1	MIR323		
cg20796695	MIR758	MIR329-1	MIR323A	MIR1197		
cg11035122	MIR758	MIR329-2	MIR329-1	MIR323		
cg09250367	MIR758	MIR323	MIR329-2	MIR329-1		
cg13637893	MIR758	MIR329-2	MIR329-1			
cg23935255	MIR758	MIR329-2	MIR329-1			
cg21456450	MIR329-1	MIR329-2	MIR758			
cg08969114	MIR329-1	MIR329-2	MIR758			
cg01587049	MIR329-2	MIR329-1	MIR758			
cg00752143	MIR329-2	MIR329-1				
cg13435266	MIR329-2	MIR329-1				
cg12044862	MIR329-2	MIR329-1				
cg02047319	MIR654	MIR376C	MIR376A2			
cg01200264	MIR654	MIR376B	MIR376C	MIR376A2		
cg10268144	MIR654	MIR376B	MIR376C	MIR376A2		
cg20084852	MIR654	MIR376B	MIR376A1	MIR376A2	MIR376C	
cg08095475	MIR654	MIR376B	MIR376A1	MIR376A2	MIR376C	
cg14037354	MIR654	MIR376B	MIR376A1	MIR376A2	MIR376C	
cg14371292	MIR654	MIR376B	MIR376A1	MIR376A2	MIR376C	
cg13995230	MIR654	MIR376B	MIR376A1	MIR376A2	MIR376C	
cg19757573	MIR654	MIR376B	MIR376A2	MIR300	MIR376A1	
cg12881150	MIR654	MIR376B	MIR376A2	MIR300	MIR376A1	
cg14144314	MIR376B	MIR376A2	MIR654	MIR300	MIR376A1	
cg17869516	MIR376B	MIR376A1	MIR300	MIR654	MIR376A2	
cg04585937	MIR376B	MIR654	MIR300	MIR376A1		
cg21882238	MIR654	MIR300	MIR376A1	MIR376B		
cg05348084	MIR376B	MIR300	MIR376A1			
cg13098800	MIR1185-2	MIR1185-1				

cg01709493	MIR1185-1	MIR1185-2				
cg00844429	MIR487B	MIR381				
cg26233951	MIR381	MIR487B	MIR381HG			
cg10177827	MIR487B	MIR381				
cg25296532	MIR487B	MIR381	MIR539	MIR381HG		
cg17613498	MIR487B	MIR381	MIR539	MIR381HG		
cg01078903	MIR487B	MIR539				
cg02882979	MIR487B	MIR539				
cg19560831	MIR487B	MIR539				
cg18863119	MIR487B	MIR539				
cg14667980	MIR539	MIR487B	MIR381HG			
cg14361526	MIR487B	MIR539				
cg02044725	MIR889	MIR487B	MIR539			
cg18604419	MIR889	MIR539				
cg08051194	MIR539	MIR889	MIR381HG			
cg18096939	MIR889	MIR539				
cg08509270	MIR889	MIR539				
cg09337069	MIR889	MIR539				
cg21124040	MIR544A	MIR381HG	MIR655			
cg21346855	MIR544A	MIR381HG	MIR655			
cg00707427	MIR544A	MIR381HG	MIR655			
cg10734581	MIR134	MIR382				
cg14942536	MIR134	MIR668	MIR382			
cg01533177	MIR134	MIR668	MIR382	MIR485		
cg12573499	MIR134	MIR668	MIR382	MIR485		
cg01648556	MIR134	MIR382	MIR668	MIR485		
cg22423977	MIR134	MIR668	MIR485	MIR382		
cg26238975	MIR134	MIR668	MIR382	MIR485		
cg11439869	MIR668	MIR485	MIR134			
cg13753460	MIR134	MIR668	MIR485			
cg04761768	MIR453	MIR485	MIR668			
cg21103170	MIR453	MIR485	MIR668			
cg06934265	MIR453	MIR485	MIR668			
cg23488816	MIR453	MIR485	MIR668			
cg14431528	MIR453	MIR485	MIR668			
cg01119169	MIR485	MIR453	MIR668			
cg18444589	MIR485	MIR453	MIR668			
cg21071540	MIR485	MIR453				
cg23940023	MIR485	MIR453				
cg09291131	MIR453	MIR485				
cg11069071	MIR496	MIR154				
cg06265809	MIR496	MIR154				
cg21492137	MIR496	MIR154				
cg05249271	MIR496	MIR154				
cg06753050	MIR496	MIR154				
cg15508280	MIR496	MIR154				
cg09213165	MIR154	MIR496				
cg23468699	MIR496	MIR377				
cg07126399	MIR412	MIR541	MIR409	MIR369		
cg03050096	MIR412	MIR541	MIR409	MIR369		

cg10948070	MIR412	MIR409	MIR369	MIR541		
cg21230021	MIR412	MIR410	MIR409	MIR369	MIR541	
cg04672182	MIR409	MIR412	MIR541	MIR369	MIR410	
cg02180737	MIR412	MIR410	MIR409	MIR369	MIR541	
cg07918453	MIR412	MIR410	MIR409	MIR369		
cg20547131	MIR412	MIR409	MIR410	MIR369		
cg23270924	MIR409	MIR410	MIR412	MIR369	MIR656	
cg14285142	MIR409	MIR410	MIR412	MIR369	MIR656	
cg18264298	MIR410	MIR409	MIR412	MIR369	MIR656	
cg02887623	MIR369	MIR410	MIR412	MIR656		
cg12782201	MIR369	MIR410	MIR412	MIR656		
cg05537796	MIR369	MIR410	MIR412	MIR656		
cg14007694	MIR369	MIR410	MIR412	MIR656		
cg22977139	MIR369	MIR410	MIR412	MIR656		
cg25122402	MIR369	MIR410	MIR656			
cg19944840	MIR369	MIR410	MIR656			
cg21878393	MIR410	MIR369	MIR656			
cg18269894	MIR410	MIR656				
cg02582514	MIR410	MIR656				
cg05297328	MIR410	MIR656				
cg21513316	MIR410	MIR656				
cg03721993	MIR1247					
cg00328051	MIR1247					
cg22325330	MIR1247					
cg10590512	MIR1247					
cg01693026	MIR1247					
cg18301891	MIR1247					
cg15238382	MIR1247					
cg02287710	MIR1247					
cg22688428	MIR1247					
cg01664864	MIR1247					
cg02840823	MIR1247					
cg15341124	MIR1247					
cg18804615	MIR1247					
cg00741900	MIR1247					
cg17188759	MIR203A	MIR203B				
cg14521367	MIR6765					
cg24705841	MIR4715					
cg09976282	MIR211					
cg01684389	MIR211					
cg06314969	MIR211					
cg13905606	MIR211					
cg20260028	MIR211					
cg08096702	MIR211					
cg16930925	MIR626					
cg22894805	MIR626					
cg22233082	MIR627					
cg14472937	MIR1282					
cg15563355	MIR1282					
cg14864357	MIR1282					

cg21190038	MIR1282					
cg04521583	MIR1282					
cg10579797	MIR1282					
cg14661236	MIR147B					
cg20636382	MIR147B					
cg23505252	MIR147B					
cg15372290	MIR147B					
cg08436772	MIR147B					
cg08824909	MIR147B					
cg02655980	MIR4716					
cg00360534	MIR4716					
cg08860879	MIR4716					
cg08390622	MIR4716					
cg25998678	MIR4716					
cg01267648	MIR4716					
cg03144814	MIR7973-2					
cg06706204	MIR1266					
cg20615141	MIR1266					
cg13174229	MIR1266					
cg07567679	MIR1266					
cg12392104	MIR628					
cg09672452	MIR628					
cg20033079	MIR628					
cg20070464	MIR628					
cg18806815	MIR628					
cg10193091	MIR2116					
cg01362581	MIR2116					
cg03297845	MIR2116					
cg11440679	MIR2116					
cg17365618	MIR2116					
cg09392381	MIR190					
cg10545768	MIR190					
cg11560666	MIR190					
cg02219476	MIR190					
cg03944501	MIR190					
cg04182114	MIR190					
cg23486561	MIR190					
cg09610163	MIR190					
cg18065177	MIR190					
cg18901478	MIR1272					
cg12002820	MIR1272					
cg10537328	MIR1272					
cg11935618	MIR1272					
cg00089864	MIR1272					
cg15533448	MIR4311					
cg08397218	MIR4311					
cg12945769	MIR548H4					
cg03066823	MIR548H4					
cg27357049	MIR548H4					
cg17286244	MIR548H4					

cg23698666	MIR548H4					
cg24750854	MIR548H4					
cg27236331	MIR548H4					
cg14554415	MIR548H4					
cg09886641	MIR548H4					
cg07775813	MIR548H4					
cg13757194	MIR548H4					
cg26820811	MIR548H4					
cg09476148	MIR548H4					
cg25991730	MIR548H4					
cg15985873	MIR548H4					
cg03162973	MIR548H4					
cg08500698	MIR548H4					
cg04880611	MIR548H4					
cg14251844	MIR548H4					
cg22169791	MIR548H4					
cg15477165	MIR548H4					
cg05387996	MIR548H4					
cg09253111	MIR548H4					
cg20823973	MIR548H4					
cg12734206	MIR548H4					
cg13848961	MIR548H4					
cg07446205	MIR548H4					
cg18819275	MIR548H4					
cg15197583	MIR548H4					
cg25345561	MIR548H4					
cg15040157	MIR548H4					
cg08148660	MIR548H4					
cg20532999	MIR548H4					
cg17073392	MIR548H4					
cg09453870	MIR548H4					
cg27118929	MIR548H4					
cg02670903	MIR548H4					
cg19461383	MIR548H4					
cg22952917	MIR548H4					
cg06680906	MIR548H4					
cg12704993	MIR548H4					
cg11400650	MIR548H4					
cg25173269	MIR548H4					
cg04525074	MIR548H4					
cg20577165	MIR548H4					
cg23818401	MIR548H4					
cg07344019	MIR548H4					
cg23966476	MIR548H4					
cg18149794	MIR548H4					
cg20189274	MIR548H4					
cg17635774	MIR548H4					
cg02002788	MIR548H4					
cg15098410	MIR548H4					
cg24399529	MIR548H4					

cg00687674	MIR548H4					
cg18263455	MIR548H4					
cg23036340	MIR548H4					
cg18445764	MIR548H4					
cg14851122	MIR548H4					
cg08041140	MIR548H4					
cg26208764	MIR548H4					
cg09662531	MIR548H4					
cg06222917	MIR548H4					
cg15241106	MIR548H4					
cg23365173	MIR548H4					
cg00309133	MIR548H4					
cg19993316	MIR548H4					
cg22381808	MIR548H4					
cg20363989	MIR548H4					
cg07057074	MIR548H4					
cg13086606	MIR548H4					
cg17641440	MIR548H4					
cg26929513	MIR548H4					
cg21605389	MIR548H4					
cg13343238	MIR548H4					
cg10098175	MIR548H4					
cg25584422	MIR548H4					
cg07148591	MIR548H4					
cg22192614	MIR548H4					
cg13460319	MIR548H4					
cg20948366	MIR629					
cg05185738	MIR629					
cg17972789	MIR629					
cg13912196	MIR629					
cg02852421	MIR629					
cg16168683	MIR629					
cg06586700	MIR4513					
cg22363909	MIR4513					
cg18503694	MIR6882					
cg18146964	MIR631					
cg19563248	MIR631					
cg00260818	MIR631					
cg02012771	MIR631					
cg03387238	MIR631					
cg02426940	MIR631					
cg16318949	MIR184					
cg13096580	MIR5572					
cg14239640	MIR5572					
cg06612459	MIR5572					
cg08976738	MIR5572					
cg16530970	MIR549					
cg22929280	MIR549					
cg03589751	MIR549					
cg05208249	MIR549					

cg05721790	MIR549					
cg15421821	MIR4515					
cg06773519	MIR4515					
cg18488733	MIR1276					
cg01766718	MIR1276					
cg17457701	MIR1276					
cg11479035	MIR1276					
cg22243298	MIR1276					
cg13892257	MIR1276					
cg08616234	MIR1276					
cg26291655	MIR1276					
cg12058064	MIR1276					
cg06527213	MIR1276					
cg03682656	MIR6766					
cg01407044	MIR6766					
cg03922814	MIR5094					
cg14266604	MIR5094					
cg07276140	MIR3174					
cg19652186	MIR3175					
cg20705518	MIR3175					
cg00267482	MIR3175					
cg15832108	MIR3175					
cg26584653	MIR1469					
cg21318213	MIR1469					
cg04330371	MIR1469					
cg23042706	MIR1469					
cg02278499	MIR1469					
cg18614734	MIR1469					
cg01501208	MIR1469					
cg23763043	MIR1469					
cg09500815	MIR1469					
cg05127369	MIR1469					
cg15070718	MIR1469					
cg15533524	MIR3176					
cg06502071	MIR662					
cg16108835	MIR662					
cg26945867	MIR662					
cg26775289	MIR662					
cg16159090	MIR3177					
cg08983215	MIR1225					
cg10268345	MIR1225					
cg05598886	MIR1225					
cg07036112	MIR1225					
cg18447131	MIR1225					
cg01965047	MIR1225					
cg13949829	MIR1225					
cg02879554	MIR1225					
cg19949931	MIR1225					
cg16187303	MIR4516					
cg08683389	MIR6768					

cg07224637	MIR6769A					
cg11631342	MIR4718					
cg27288741	MIR6511B2	MIR6511B1				
cg01109243	MIR484					
cg00485047	MIR484					
cg22178085	MIR484					
cg07370023	MIR484					
cg09730820	MIR484					
cg20342118	MIR484					
cg09662369	MIR484					
cg17737088	MIR484					
cg12706810	MIR484					
cg16786260	MIR3680-2	MIR3680-1				
cg04271870	MIR548D2	MIR548AA2				
cg13273037	MIR548D2	MIR548AA2				
cg15307230	MIR548D2	MIR548AA2				
cg22916042	MIR548D2	MIR548AA2				
cg20671702	MIR4721					
cg06682219	MIR4721					
cg11725326	MIR4517					
cg16804902	MIR4517					
cg01866220	MIR4517					
cg19641184	MIR4517					
cg16797148	MIR4517					
cg26837844	MIR4517					
cg23054308	MIR3680-2	MIR3680-1				
cg16935914	MIR762HG					
cg18129863	MIR762HG					
cg10854110	MIR762HG					
cg27048624	MIR762HG	MIR4519				
cg04571421	MIR4519	MIR762HG				
cg22264517	MIR4519	MIR762HG				
cg02396086	MIR4519	MIR762HG				
cg06036686	MIR762HG					
cg03372467	MIR762					
cg02558026	MIR762					
cg09041483	MIR762					
cg00960050	MIR762					
cg07378245	MIR762					
cg08297053	MIR762					
cg06665774	MIR762HG					
cg10515900	MIR762HG					
cg21469373	MIR762HG					
cg24169735	MIR762HG					
cg13914857	MIR762HG					
cg06625777	MIR762HG					
cg01064941	MIR762HG					
cg13031987	MIR548AE2					
cg24246947	MIR548AE2					
cg04779243	MIR548AE2					

cg24975947	MIR548AE2					
cg00843452	MIR548AE2					
cg08378275	MIR548AE2					
cg11564551	MIR548AE2					
cg26351511	MIR548AE2					
cg16956574	MIR548AE2					
cg15859072	MIR548AE2					
cg09945985	MIR548AE2					
cg15832560	MIR548AE2	MIR5095				
cg04127262	MIR548AE2	MIR5095				
cg21489445	MIR548AE2	MIR5095				
cg27415713	MIR548AE2	MIR5095				
cg01314715	MIR548AE2	MIR5095				
cg10667857	MIR548AE2	MIR5095				
cg21046624	MIR548AE2	MIR5095				
cg07994786	MIR548AE2	MIR5095				
cg05924741	MIR548AE2	MIR5095				
cg12966005	MIR548AE2	MIR5095				
cg08558652	MIR548AE2	MIR5095				
cg23923179	MIR548AE2	MIR5095				
cg01782749	MIR548AE2	MIR5095				
cg17673059	MIR548AE2	MIR5095				
cg25222357	MIR548AE2	MIR5095				
cg22224257	MIR548AE2	MIR5095				
cg00816290	MIR548AE2	MIR5095				
cg07828800	MIR548AE2	MIR5095				
cg05147158	MIR548AE2	MIR5095				
cg16862518	MIR548AE2	MIR5095				
cg00071872	MIR548AE2	MIR5095				
cg18763079	MIR548AE2	MIR5095				
cg17685131	MIR5095					
cg04433936	MIR5095					
cg13615360	MIR5095					
cg04868709	MIR5095					
cg14929554	MIR5095					
cg10849147	MIR5095					
cg11063021	MIR5095					
cg17567941	MIR5095					
cg00766382	MIR5095					
cg09730359	MIR5095					
cg01723420	MIR5095					
cg00204984	MIR5095					
cg13440132	MIR5095					
cg04862658	MIR5095					
cg23820714	MIR5095					
cg27309907	MIR5095					
cg25283230	MIR5095					
cg00327399	MIR5095					
cg23810424	MIR5095					
cg03013259	MIR5095					

cg17345633	MIR5095					
cg02839651	MIR3181					
cg19767580	MIR3181					
cg06149446	MIR3935					
cg22328598	MIR6863					
cg16902958	MIR6772					
cg16421621	MIR328					
cg03125605	MIR328					
cg04650403	MIR328					
cg07173484	MIR328					
cg00054301	MIR328					
cg19966955	MIR6773					
cg06912425	MIR6773					
cg06386401	MIR6773					
cg25534638	MIR7641-2					
cg21898052	MIR1538					
cg01301935	MIR1538					
cg26670552	MIR1538					
cg26967433	MIR1538					
cg08665271	MIR1538					
cg16996262	MIR1538					
cg17985281	MIR1538					
cg07307803	MIR1538					
cg01814186	MIR1538					
cg08804013	MIR1538					
cg03417340	MIR140					
cg00699693	MIR140					
cg07281938	MIR140					
cg03848856	MIR140					
cg01735503	MIR140					
cg03549146	MIR140					
cg00158530	MIR140					
cg08209934	MIR140					
cg04703221	MIR140					
cg03885048	MIR1972-1	MIR1972-2				
cg03009864	MIR1972-1	MIR1972-2				
cg09213330	MIR1972-1	MIR1972-2				
cg24834981	MIR7854					
cg12481789	MIR6504					
cg14571071	MIR6504					
cg16854097	MIR6504					
cg05712513	MIR6504					
cg07829693	MIR1910					
cg25575961	MIR1910					
cg26317934	MIR1910					
cg01664843	MIR1910					
cg01770799	MIR6774					
cg27467238	MIR6775					
cg00052925	MIR6775					
cg08941871	MIR4722					

cg02972607	MIR3183					
cg12765886	MIR22HG					
cg06053959	MIR22					
cg26697882	MIR22					
cg06927305	MIR22					
cg22516975	MIR22					
cg19015264	MIR22					
cg17801058	MIR22HG	MIR22				
cg02529627	MIR22					
cg03775941	MIR22HG					
cg19896046	MIR22HG					
cg12696026	MIR22HG					
cg03244874	MIR22HG					
cg13191319	MIR22HG					
cg14610962	MIR132	MIR212				
cg16048942	MIR132	MIR212				
cg21810173	MIR212	MIR132				
cg00940313	MIR212	MIR132				
cg02342533	MIR212	MIR132				
cg18758230	MIR212	MIR132				
cg04631281	MIR212	MIR132				
cg03978498	MIR212	MIR132				
cg22208012	MIR212	MIR132				
cg03592438	MIR6776					
cg12537546	MIR497HG					
cg06679494	MIR497	MIR195				
cg02329670	MIR497	MIR195				
cg21040575	MIR497	MIR195				
cg24257550	MIR497HG	MIR195	MIR497			
cg11263011	MIR195	MIR497				
cg04430582	MIR195	MIR497				
cg14634563	MIR324					
cg12579196	MIR324					
cg06674503	MIR4314					
cg27617207	MIR744					
cg01234911	MIR744					
cg01678692	MIR744					
cg26397352	MIR744					
cg18156845	MIR744					
cg26779305	MIR744					
cg06775068	MIR1269B					
cg26804183	MIR1288					
cg16848280	MIR1288					
cg25591377	MIR1288					
cg00442174	MIR1288					
cg20544516	MIR33B					
cg24161106	MIR33B					
cg11393407	MIR33B					
cg09494646	MIR33B					
cg13245539	MIR33B					

cg07415388	MIR33B					
cg20443227	MIR6777	MIR33B				
cg03164243	MIR33B					
cg04805065	MIR33B					
cg09186408	MIR33B					
cg19619576	MIR33B					
cg00017887	MIR6778					
cg24357003	MIR6778					
cg05896714	MIR1180					
cg19157647	MIR1180					
cg04864152	MIR1180					
cg20272287	MIR1180					
cg07733567	MIR1180					
cg24553547	MIR1180					
cg24547137	MIR1180					
cg02796621	MIR1180					
cg02206323	MIR1180					
cg26619894	MIR1180					
cg19196414	MIR451	MIR144				
cg16670446	MIR451	MIR144				
cg12682573	MIR451	MIR144				
cg09030392	MIR451	MIR144				
cg17723549	MIR423					
cg05054006	MIR423					
cg01863613	MIR423					
cg26813908	MIR423					
cg27066254	MIR423					
cg21930443	MIR423					
cg03837627	MIR423					
cg13770529	MIR423					
cg25346576	MIR423					
cg07855056	MIR423					
cg14888846	MIR423					
cg24102938	MIR423					
cg24675879	MIR423					
cg17369088	MIR423					
cg05141870	MIR423					
cg11257193	MIR423					
cg18949641	MIR4733					
cg01373258	MIR4724					
cg15005345	MIR632					
cg09903166	MIR632					
cg03529568	MIR632					
cg19617599	MIR632					
cg25191231	MIR632					
cg04865412	MIR632					
cg21184495	MIR632					
cg16007628	MIR632					
cg08209724	MIR632					
cg20017464	MIR632					

cg21680502	MIR4727					
cg19862334	MIR6779					
cg26403285	MIR6866					
cg02249795	MIR6866					
cg14511007	MIR6866					
cg03576592	MIR6510					
cg08943214	MIR548AT					
cg03810621	MIR6781					
cg26883949	MIR6781					
cg06264199	MIR6784					
cg21384971	MIR152					
cg24389730	MIR152					
cg10382221	MIR152					
cg05096161	MIR152					
cg06598332	MIR152					
cg09111258	MIR152					
cg02742085	MIR152					
cg14949813	MIR152					
cg04458627	MIR152					
cg18075185	MIR152					
cg01521220	MIR1203					
cg11210138	MIR1203					
cg17019616	MIR1203					
cg17826531	MIR1203					
cg12964647	MIR1203					
cg21164660	MIR6129					
cg01638497	MIR6165					
cg16280986	MIR6165					
cg13849515	MIR3614					
cg06625332	MIR3614					
cg09342858	MIR3614					
cg14890660	MIR454					
cg26111107	MIR454					
cg19343352	MIR454					
cg01964121	MIR454					
cg10325038	MIR454					
cg10822495	MIR301A					
cg02656609	MIR301A					
cg02611741	MIR301A					
cg07505964	MIR301A					
cg13132363	MIR301A					
cg19273756	MIR301A					
cg14032089	MIR21					
cg08342762	MIR548W					
cg01033787	MIR548W					
cg04660733	MIR548W					
cg16241842	MIR548W					
cg14733743	MIR548W					
cg04115908	MIR548W					
cg16094326	MIR548W					

cg05099035	MIR548W					
cg01593570	MIR548W					
cg20194910	MIR548W					
cg13095998	MIR548W					
cg04959250	MIR548W					
cg13547672	MIR548W					
cg06634637	MIR548W					
cg24816622	MIR548W					
cg05976412	MIR548W					
cg23371940	MIR548W					
cg14487926	MIR548W					
cg23726145	MIR548W					
cg23013151	MIR548W					
cg07101541	MIR548W					
cg27047829	MIR548W					
cg07871369	MIR548W					
cg20836224	MIR548W					
cg27097489	MIR548W					
cg15511049	MIR548W					
cg02219451	MIR548W					
cg21012050	MIR548W					
cg18528621	MIR548W					
cg02611257	MIR548W					
cg22414362	MIR548W					
cg04855975	MIR548W					
cg23417140	MIR548W					
cg13960857	MIR633	MIR548W				
cg21781319	MIR633	MIR548W				
cg26640037	MIR633	MIR548W				
cg01334081	MIR548W					
cg10046367	MIR548W					
cg06360318	MIR548W					
cg18122056	MIR548W					
cg04093078	MIR548W					
cg18982976	MIR548W					
cg15369381	MIR548W					
cg15163417	MIR548W					
cg21952149	MIR548W					
cg15252599	MIR548W					
cg11242972	MIR548W					
cg15095482	MIR548W					
cg07374160	MIR548W					
cg17864737	MIR548W					
cg07398555	MIR548W					
cg24791843	MIR548W					
cg20848842	MIR548W					
cg05351998	MIR548W					
cg00169586	MIR548W					
cg18876162	MIR548W					
cg06921368	MIR6080					

cg02007225	MIR4315-2	MIR4315-1				
cg15724116	MIR634					
cg25134567	MIR634					
cg04153722	MIR634					
cg20152382	MIR634					
cg09259308	MIR635					
cg07043025	MIR635					
cg08963258	MIR635					
cg04798303	MIR635					
cg12896445	MIR635					
cg11375638	MIR4524B	MIR4524A				
cg19942007	MIR4524A					
cg03633686	MIR4524A					
cg09408064	MIR3615					
cg01814935	MIR3615					
cg16361383	MIR3678					
cg22731393	MIR4738					
cg04782064	MIR4738					
cg26199443	MIR4738					
cg09964116	MIR4538					
cg09025253	MIR636					
cg12404798	MIR636					
cg23473419	MIR636					
cg26815021	MIR636					
cg17205803	MIR636					
cg16046954	MIR636					
cg23263051	MIR636					
cg25333216	MIR636					
cg00819114	MIR636					
cg03570858	MIR636					
cg01055610	MIR636					
cg19125323	MIR636					
cg22450146	MIR636					
cg12503190	MIR636					
cg13324603	MIR636					
cg21028463	MIR636					
cg01574134	MIR636					
cg05482832	MIR636					
cg25538649	MIR636					
cg22059098	MIR636					
cg05394852	MIR6516					
cg23471991	MIR6516					
cg21586296	MIR6516					
cg26980256	MIR6516					
cg15818827	MIR6516					
cg14158468	MIR6516					
cg25171729	MIR6516					
cg15398430	MIR6516					
cg06414998	MIR4730					
cg20292484	MIR3065					

cg18609037	MIR657					
cg19835688	MIR3065	MIR657				
cg02317955	MIR657					
cg26766064	MIR657	MIR338				
cg18637486	MIR657	MIR338				
cg06807993	MIR657	MIR338				
cg06869212	MIR657	MIR338				
cg23295826	MIR657	MIR338				
cg11600078	MIR657	MIR338				
cg23176214	MIR657	MIR338				
cg26068527	MIR657	MIR338				
cg24085713	MIR338					
cg13466409	MIR1250					
cg11138142	MIR1250					
cg02004044	MIR1250					
cg24688837	MIR1250					
cg23535449	MIR1250					
cg11236755	MIR1250					
cg20273576	MIR1250					
cg26998842	MIR1250					
cg17517390	MIR1250					
cg08106445	MIR4740					
cg10914865	MIR4740					
cg14140599	MIR3186					
cg15394672	MIR6786					
cg08782169	MIR6786					
cg12714522	MIR6787					
cg07238273	MIR6787					
cg11321186	MIR6787					
cg21974464	MIR6787					
cg23216916	MIR6787					
cg08693080	MIR4525					
cg06099315	MIR8078					
cg11124635	MIR6718					
cg02942546	MIR4317					
cg05747251	MIR4317					
cg10248218	MIR4526					
cg09895876	MIR4526					
cg12273502	MIR4526					
cg02292342	MIR4526					
cg05766064	MIR320C1					
cg25890168	MIR320C1					
cg02645550	MIR320C1					
cg15671915	MIR133A1					
cg21024311	MIR133A1					
cg16915294	MIR133A1HG	MIR133A1				
cg07202461	MIR133A1					
cg18554492	MIR133A1HG	MIR133A1				
cg06865629	MIR1-2					
cg11475886	MIR133A1HG	MIR1-2				

cg17106157	MIR1-2					
cg15236080	MIR133A1HG					
cg22204047	MIR4741					
cg22806068	MIR4741					
cg18253971	MIR320C2					
cg24399872	MIR320C2					
cg18288303	MIR320C2					
cg01962062	MIR320C2					
cg27422060	MIR5583-1					
cg22576612	MIR4319					
cg11070777	MIR4319					
cg16126877	MIR4743					
cg01878705	MIR1539					
cg18394275	MIR1539					
cg21736038	MIR1539					
cg13361168	MIR1539					
cg06655225	MIR1539					
cg17675546	MIR1539					
cg09885380	MIR1539					
cg08879181	MIR4529					
cg03217254	MIR1302-2	MIR1302-10	MIR1302-9	MIR1302-11		
cg20442503	MIR1909					
cg09703947	MIR1909					
cg03413810	MIR1909					
cg00969570	MIR1909					
cg24774002	MIR1909					
cg27091642	MIR1909					
cg02467518	MIR1909					
cg01386868	MIR1909					
cg02368428	MIR1909					
cg04207179	MIR1909					
cg14763933	MIR1909					
cg23837289	MIR1909					
cg26480358	MIR1909					
cg18654464	MIR1227					
cg14066773	MIR1227					
cg24866700	MIR1227					
cg01627405	MIR1227					
cg03694555	MIR1227					
cg20930863	MIR6789					
cg12804227	MIR6789					
cg16907400	MIR4321					
cg16964413	MIR7108					
cg14933412	MIR7850					
cg25109528	MIR1268A					
cg26764880	MIR1268A					
cg08893293	MIR1268A					
cg15190406	MIR1268A					
cg10094037	MIR1268A					
cg00968931	MIR1268A					

cg02124727	MIR1268A					
cg05079117	MIR1268A					
cg22091742	MIR1268A					
cg14885826	MIR1268A					
cg12688336	MIR1268A					
cg24695115	MIR1268A					
cg05421282	MIR1268A					
cg02710210	MIR1268A					
cg09896650	MIR1268A					
cg02591892	MIR1268A					
cg09739816	MIR1268A					
cg05562282	MIR1268A					
cg16673791	MIR1268A					
cg00433107	MIR1268A					
cg23908593	MIR1268A					
cg02721852	MIR1268A					
cg19554207	MIR1268A					
cg25916625	MIR1268A					
cg10915225	MIR1268A					
cg15830378	MIR1268A					
cg13020663	MIR1268A					
cg01316116	MIR1268A					
cg05295233	MIR1268A					
cg09319210	MIR1268A					
cg04936377	MIR1268A					
cg10502071	MIR1268A					
cg16164739	MIR1268A					
cg16494576	MIR1268A					
cg21376204	MIR1268A					
cg16543247	MIR1268A					
cg08000406	MIR1268A					
cg10825521	MIR1268A					
cg09556749	MIR1268A					
cg04119107	MIR1268A					
cg05034718	MIR1268A					
cg26837739	MIR1268A					
cg09505423	MIR1268A					
cg27053789	MIR1268A					
cg05916938	MIR1268A					
cg13795156	MIR1268A					
cg05048676	MIR1268A					
cg03656862	MIR1268A					
cg27328231	MIR1268A					
cg09002055	MIR1268A					
cg25472617	MIR1268A					
cg00611436	MIR1268A					
cg22302608	MIR1268A					
cg20693146	MIR1268A					
cg19072837	MIR1268A					
cg13811969	MIR1268A					

cg22782812	MIR1268A					
cg21690947	MIR1268A					
cg14128911	MIR1268A					
cg01074840	MIR1268A					
cg19433767	MIR1268A					
cg26090256	MIR1268A					
cg24283288	MIR1268A					
cg27028514	MIR637					
cg06494105	MIR637					
cg23953773	MIR637					
cg09402652	MIR637					
cg22771960	MIR637					
cg18355533	MIR637					
cg09590897	MIR637					
cg18331165	MIR637					
cg21887303	MIR637					
cg24943002	MIR637					
cg14520588	MIR1268A					
cg06455385	MIR1268A					
cg05444640	MIR1268A					
cg25219651	MIR1268A					
cg05716290	MIR1268A					
cg24763265	MIR1268A					
cg21300318	MIR7-3					
cg20893022	MIR7-3					
cg19646028	MIR7-3					
cg01400401	MIR7-3					
cg04640886	MIR7-3					
cg02479575	MIR7-3					
cg26005082	MIR7-3					
cg07642566	MIR7-3					
cg21927946	MIR7-3					
cg27547281	MIR7-3					
cg02351925	MIR7-3					
cg20375099	MIR6790					
cg16804044	MIR6790					
cg25458523	MIR6790					
cg14684642	MIR6790					
cg04942111	MIR6790					
cg00083848	MIR6790					
cg06299872	MIR6790					
cg04866810	MIR220B					
cg12088501	MIR6791					
cg05215474	MIR6791					
cg08760431	MIR4999					
cg09533720	MIR4322					
cg10007372	MIR1181					
cg22683879	MIR1181					
cg17602882	MIR1181					
cg25732522	MIR1181					

cg10271981	MIR1181					
cg05370860	MIR1181					
cg25212776	MIR1181					
cg01522721	MIR1181					
cg12004065	MIR1181					
cg12220843	MIR1181					
cg00876541	MIR1181					
cg19324704	MIR1238					
cg03611912	MIR1238					
cg09067234	MIR1238					
cg06871184	MIR1238					
cg12956598	MIR1238					
cg19852264	MIR1238					
cg05648345	MIR1238					
cg02446225	MIR1238					
cg19485268	MIR638					
cg20727147	MIR638					
cg03638677	MIR638					
cg26994774	MIR638					
cg10873503	MIR638					
cg04740198	MIR638					
cg00735591	MIR638					
cg14508213	MIR638					
cg20041257	MIR638					
cg08207256	MIR638					
cg04360313	MIR638					
cg26257177	MIR638					
cg13432391	MIR638					
cg25487903	MIR638					
cg10948777	MIR638					
cg04349565	MIR638					
cg17165284	MIR638					
cg15392792	MIR638					
cg07248407	MIR638					
cg00466073	MIR4748					
cg27648270	MIR199A1					
cg18544365	MIR199A1					
cg23047544	MIR199A1					
cg02660440	MIR199A1					
cg06754197	MIR199A1					
cg02907064	MIR199A1					
cg23068797	MIR199A1					
cg03216043	MIR199A1					
cg13965612	MIR199A1					
cg14926083	MIR6886					
cg03695608	MIR7974					
cg20399810	MIR7974					
cg19357865	MIR27A	MIR24-2				
cg02990289	MIR27A	MIR24-2				
cg17550784	MIR27A	MIR24-2	MIR23A			

cg06606386	MIR27A	MIR24-2	MIR23A			
cg11429044	MIR23A	MIR27A	MIR24-2			
cg07706375	MIR23A	MIR24-2	MIR27A			
cg08528170	MIR24-2	MIR23A	MIR27A			
cg15787712	MIR24-2	MIR23A	MIR27A			
cg14145185	MIR24-2	MIR23A	MIR27A			
cg06855983	MIR181C	MIR181D				
cg08310363	MIR181C	MIR181D				
cg00716579	MIR181C	MIR181D				
cg18745782	MIR181D	MIR181C				
cg09747671	MIR181C	MIR181D				
cg10822545	MIR181D	MIR181C				
cg26514117	MIR181D	MIR181C				
cg24482288	MIR181D	MIR181C				
cg15972425	MIR1199					
cg05922058	MIR1199					
cg02149160	MIR1199					
cg18169289	MIR1199					
cg06905901	MIR639					
cg11581046	MIR639					
cg09782300	MIR639					
cg04329845	MIR639					
cg12846938	MIR639					
cg20630207	MIR639					
cg03205495	MIR639					
cg08434594	MIR639					
cg14339287	MIR639					
cg10649714	MIR639					
cg17162271	MIR639					
cg22469141	MIR639					
cg13237829	MIR639					
cg23470272	MIR639					
cg15649193	MIR639					
cg14898306	MIR639					
cg02849766	MIR1470					
cg12677723	MIR1470					
cg14892570	MIR1470					
cg21216828	MIR1470					
cg20702935	MIR1470					
cg15448064	MIR1470					
cg08269119	MIR1470					
cg27569742	MIR1470					
cg06553422	MIR1470					
cg07531072	MIR1470					
cg12224165	MIR1470					
cg21293810	MIR3188					
cg25387811	MIR640					
cg14251798	MIR640					
cg05403831	MIR640					
cg14873313	MIR6887					

cg09612163	MIR6719					
cg27628515	MIR641					
cg06055845	MIR641					
cg26620021	MIR641					
cg07815521	MIR641					
cg09380135	MIR641					
cg07204707	MIR6796					
cg17035787	MIR6796					
cg20969585	MIR4323					
cg26946818	MIR4323					
cg07404579	MIR4323					
cg14924324	MIR4323					
cg13354838	MIR8077					
cg04015004	MIR4531					
cg21109795	MIR8085					
cg10658072	MIR8085					
cg14858790	MIR8085					
cg09559882	MIR330					
cg09545123	MIR330					
cg03096975	MIR330					
cg07804711	MIR330					
cg12536502	MIR330					
cg07749923	MIR330					
cg16543391	MIR330					
cg01544351	MIR330					
cg25277950	MIR330					
cg18515250	MIR330					
cg00029256	MIR330					
cg27559408	MIR330					
cg02869289	MIR642					
cg13602484	MIR642					
cg13652372	MIR642					
cg02452310	MIR642					
cg24565698	MIR642A					
cg00283662	MIR642					
cg00433887	MIR769					
cg02624019	MIR769					
cg08426951	MIR769					
cg02072464	MIR320E					
cg03281512	MIR320E					
cg21997998	MIR6798					
cg07743843	MIR4324					
cg12660698	MIR6800					
cg07407950	MIR4751					
cg23160488	MIR99B	MIRLET7E	MIR125A			
cg27367878	MIR125A	MIR99B	MIRLET7E			
cg03010301	MIR125A	MIR99B	MIRLET7E			
cg05493001	MIR125A	MIR99B	MIRLET7E			
cg11794213	MIR125A	MIR99B	MIRLET7E			
cg00633015	MIR125A	MIRLET7E				

cg19782652	MIR125A	MIRLET7E				
cg18906608	MIR125A					
cg21954156	MIR6801					
cg25273903	MIR643					
cg08642528	MIR643					
cg16383318	MIR643					
cg18131559	MIR643					
cg07472764	MIR643					
cg10360420	MIR512-1	MIR512-2				
cg05237332	MIR512-1	MIR512-2				
cg12897503	MIR512-2	MIR512-1				
cg04274236	MIR512-1	MIR512-2				
cg08160970	MIR515-2	MIR515-1				
cg17686973	MIR515-2	MIR515-1				
cg24091975	MIR515-1	MIR519E	MIR515-2			
cg03799024	MIR515-2	MIR515-1				
cg17109533	MIR515-2	MIR515-1				
cg27547695	MIR515-2	MIR515-1				
cg12247976	MIR515-1	MIR515-2	MIR519C			
cg16266268	MIR519B	MIR526B				
cg12690224	MIR519B	MIR526B				
cg09514545	MIR525	MIR523				
cg25512249	MIR518F	MIR520B				
cg19537184	MIR520C	MIR526A1				
cg23319790	MIR519D	MIR517A				
cg24858376	MIR519D	MIR517A				
cg20101164	MIR517B	MIR520G				
cg19756622	MIR518A1	MIR518E				
cg08030633	MIR517C	MIR520H				
cg14709103	MIR519A1	MIR522				
cg02991888	MIR1283-2	MIR516A1				
cg05748492	MIR519A2	MIR516A2				
cg23073467	MIR516A2	MIR519A2				
cg17365367	MIR372	MIR371				
cg22462000	MIR372	MIR371				
cg25579656	MIR372	MIR371	MIR373			
cg04379522	MIR372	MIR371	MIR373			
cg22781400	MIR372	MIR371	MIR373			
cg11616411	MIR372	MIR371	MIR373			
cg24823137	MIR372	MIR371	MIR373			
cg05882315	MIR372	MIR371	MIR373			
cg20644425	MIR372	MIR371	MIR373			
cg08239565	MIR372	MIR373				
cg01975502	MIR372	MIR373				
cg18403970	MIR372	MIR373				
cg09484214	MIR372	MIR373				
cg10200169	MIR372	MIR373				
cg16388088	MIR371B	MIR373				
cg09422614	MIR935					
cg07785717	MIR935					

cg18055623	MIR935					
cg03593550	MIR935					
cg01666793	MIR935					
cg25235766	MIR935					
cg02694017	MIR935					
cg13814875	MIR4752					
cg17070310	MIR4752					
cg04608494	MIR6802					
cg21823267	MIR6803					
cg12791085	MIR6803					
cg02357735	MIR6807					
cg06354774	MIR6869					
cg14988425	MIR6869					
cg26011437	MIR1292					
cg26132853	MIR1292					
cg19734087	MIR1292					
cg09045249	MIR1292					
cg16160867	MIR1292					
cg13115617	MIR1292					
cg10544093	MIR1292					
cg00405774	MIR1292					
cg08563487	MIR1292					
cg14542207	MIR1292					
cg01629329	MIR1292					
cg00350371	MIR1292					
cg18146506	MIR1292					
cg19137662	MIR1292					
cg24825299	MIR103-2					
cg25085038	MIR103-2					
cg12617066	MIR103-2					
cg01983504	MIR103-2					
cg19127724	MIR103-2					
cg23650853	MIR103-2					
cg12218895	MIR103-2AS					
cg12290369	MIR3192					
cg05663594	MIR3192					
cg07388837	MIR663A	MIR663AHG				
cg12420437	MIR663A	MIR663AHG				
cg02624565	MIR3193					
cg05090896	MIR1825					
cg17724336	MIR1825					
cg06075250	MIR1825					
cg25775708	MIR4755					
cg11834907	MIR644					
cg25513321	MIR499					
cg11231913	MIR499					
cg23593986	MIR499					
cg24438334	MIR499					
cg19832184	MIR499					
cg01597727	MIR499					

cg11320187	MIR499					
cg08618909	MIR1289-1					
cg19504335	MIR1289-1					
cg17721331	MIR1289-1					
cg05969522	MIR1289-1					
cg15206656	MIR1289-1					
cg20281075	MIR1289-1					
cg03151445	MIR3646					
cg10886901	MIR3616					
cg01512638	MIR1259					
cg02903601	MIR1259					
cg12959622	MIR1259					
cg24448231	MIR1259					
cg04990372	MIR1259					
cg18598146	MIR1259					
cg16704590	MIR1259					
cg01644741	MIR1259					
cg05093113	MIR645					
cg22068580	MIR1302-5					
cg08246447	MIR1302-5					
cg21983491	MIR298	MIR296				
cg03030267	MIR298	MIR296				
cg15428904	MIR298	MIR296				
cg11828048	MIR298					
cg02049368	MIR646	MIR646HG				
cg21285193	MIR646	MIR646HG				
cg14433482	MIR646	MIR646HG				
cg05375014	MIR646	MIR646HG				
cg08025208	MIR646	MIR646HG				
cg16826172	MIR4758					
cg04763539	MIR1-1HG					
cg23280890	MIR1-1HG					
cg14893206	MIR1-1HG					
cg24219126	MIR1-1HG					
cg23103726	MIR1-1HG					
cg15010213	MIR1-1					
cg16226106	MIR1-1					
cg23792592	MIR1-1					
cg12991101	MIR1-1HG	MIR1-1				
cg22754206	MIR1-1					
cg16150381	MIR1-1					
cg27337176	MIR1-1					
cg00596687	MIR1-1					
cg11797130	MIR1-1					
cg02726814	MIR1-1					
cg07915635	MIR133A2					
cg17267710	MIR133A2					
cg15076384	MIR133A2					
cg08508227	MIR133A2					
cg19973758	MIR133A2					

cg04506202	MIR133A2					
cg07691119	MIR1-1HG					
cg17455757	MIR1-1HG					
cg22192554	MIR3196					
cg26075664	MIR3196					
cg15331656	MIR3196					
cg07850515	MIR3196					
cg00466209	MIR3196					
cg04354459	MIR4326					
cg13097741	MIR4326					
cg07835236	MIR941-1					
cg10842070	MIR941-1	MIR941-3	MIR941-2			
cg05898333	MIR941-1	MIR941-3	MIR941-2			
cg03805896	MIR941-1	MIR941-3	MIR941-2			
cg22987078	MIR941-3	MIR941-2	MIR941-1			
cg08148458	MIR941-3	MIR941-2	MIR941-1			
cg15580304	MIR941-3	MIR941-2	MIR941-1			
cg14523475	MIR941-3	MIR941-2	MIR941-1			
cg01966791	MIR1914					
cg11392297	MIR1914					
cg07135405	MIR1914					
cg01073332	MIR1914	MIR647				
cg21221377	MIR1914	MIR647				
cg07507493	MIR1914	MIR647				
cg20252837	MIR1914	MIR647				
cg14440024	MIR1914	MIR647				
cg01637548	MIR647					
cg27370812	MIR647					
cg00514186	MIR647					
cg06382345	MIR99AHG					
cg21636685	MIR99AHG					
cg20630582	MIR99AHG					
cg22789730	MIR99AHG					
cg25106676	MIR99AHG					
cg01075038	MIR99AHG					
cg07500168	MIR99AHG					
cg14847493	MIR99AHG					
cg17977969	MIR99AHG					
cg08924004	MIR99AHG					
cg11702026	MIR99AHG					
cg07535042	MIR99AHG					
cg15999949	MIR99AHG					
cg11239407	MIR99AHG					
cg23539114	MIR99AHG					
cg07413966	MIR99AHG					
cg07259358	MIR99AHG					
cg02711653	MIR99AHG					
cg16240189	MIR99AHG					
cg00407017	MIR99AHG					
cg19981515	MIR99AHG					

cg04864577	MIR99AHG					
cg13953717	MIR99AHG					
cg05713399	MIR99AHG					
cg17509180	MIR99AHG					
cg18581972	MIR99AHG					
cg16063315	MIR99AHG					
cg03762502	MIR99AHG					
cg10097871	MIR99AHG					
cg07955854	MIR99AHG					
cg15230342	MIR99AHG					
cg27084959	MIR99AHG					
cg02701388	MIR99AHG					
cg20883326	MIR99AHG					
cg07592079	MIR99AHG					
cg18447751	MIR99AHG					
cg14787959	MIR99AHG					
cg24473277	MIR99AHG					
cg18983669	MIR99AHG					
cg26753518	MIR99A	MIR99AHG				
cg25353401	MIRLET7C	MIR99A				
cg21750426	MIRLET7C	MIR99A				
cg15729697	MIRLET7C	MIR99A	MIR99AHG			
cg19173502	MIRLET7C					
cg13120955	MIRLET7C					
cg19860299	MIR99AHG					
cg14571688	MIR99AHG					
cg14458094	MIR99AHG					
cg14317467	MIR99AHG					
cg15756733	MIR99AHG					
cg21692850	MIR99AHG					
cg00650240	MIR99AHG					
cg01419181	MIR99AHG					
cg04585185	MIR125B2					
cg00543364	MIR125B2					
cg27205487	MIR125B2					
cg24053070	MIR125B2					
cg19954363	MIR125B2	MIR99AHG				
cg17847345	MIR99AHG					
cg20917552	MIR155	MIR155HG				
cg04776469	MIR155	MIR155HG				
cg14544087	MIR155	MIR155HG				
cg13309012	MIR155HG	MIR155				
cg08736070	MIR5009					
cg05791108	MIR6508					
cg17360196	MIR6508					
cg16299399	MIR6508					
cg07495738	MIR6508					
cg17943520	MIR6508					
cg04116821	MIR3197					
cg04774158	MIR3197					

cg04135270	MIR3197					
cg04804434	MIR3197					
cg27595610	MIR3197					
cg00227156	MIR648					
cg19296149	MIR648					
cg16519758	MIR648					
cg15607706	MIR648					
cg09914785	MIR4761					
cg27449804	MIR4761					
cg23095729	MIR185					
cg00390484	MIR185					
cg00504285	MIR185					
cg07021468	MIR185					
cg16472542	MIR185					
cg13811469	MIR185					
cg21285564	MIR1306					
cg20689730	MIR1306					
cg01630479	MIR1306					
cg03147761	MIR1306	MIR3618				
cg00615172	MIR1306	MIR3618				
cg15454969	MIR1306	MIR3618				
cg22808478	MIR1306					
cg08706141	MIR1306					
cg07797030	MIR1306					
cg22786486	MIR1306					
cg11242552	MIR1306					
cg06670785	MIR1306					
cg06964030	MIR1306					
cg14301580	MIR6816					
cg07181341	MIR6816					
cg11632273	MIR6816					
cg21639001	MIR6816					
cg05432102	MIR6816					
cg20801666	MIR6816					
cg20566583	MIR6816					
cg12206978	MIR6816					
cg08753951	MIR1286					
cg02296376	MIR1286					
cg08221669	MIR1286					
cg06197043	MIR1286					
cg00384653	MIR1286					
cg07159686	MIR1286					
cg25037841	MIR1286					
cg22678932	MIR130B	MIR301B				
cg24330297	MIR301B	MIR130B				
cg12155013	MIR130B	MIR301B				
cg14030055	MIR130B	MIR301B				
cg02473781	MIR130B	MIR301B				
cg11673244	MIR130B	MIR301B				
cg16244770	MIR130B	MIR301B				

cg04282607	MIR301B	MIR130B				
cg07230015	MIR6817					
cg02336095	MIR6817					
cg21289124	MIR548J					
cg04479757	MIR548J					
cg22504528	MIR548J					
cg25635000	MIR548J					
cg00502618	MIR548J					
cg26210379	MIR3199-2					
cg01388803	MIR3199-2					
cg27254118	MIR3199-2					
cg02017733	MIR7109					
cg13590605	MIR7109					
cg07710843	MIR3909					
cg12241892	MIR3909					
cg27395454	MIR3909					
cg23669063	MIR6069					
cg02452950	MIR6819					
cg18297519	MIR658					
cg01839850	MIR658					
cg13641189	MIR658					
cg07699113	MIR658					
cg19070798	MIR658					
cg08519241	MIR658					
cg10470963	MIR658					
cg01182973	MIR658					
cg09189978	MIR658					
cg05046068	MIR658					
cg01508548	MIR658					
cg24233562	MIR658					
cg24845375	MIR659					
cg24578493	MIR659					
cg11245928	MIR659					
cg07059402	MIR659					
cg00565075	MIR659					
cg04076760	MIR659					
cg05211746	MIR6820					
cg14420798	MIR4534					
cg08859349	MIR4534					
cg01481205	MIR4534					
cg09916443	MIR4766					
cg00500400	MIR1281					
cg04452260	MIR1281					
cg09331127	MIR1281					
cg02046995	MIR1281					
cg24349919	MIR1281					
cg23690344	MIR6889					
cg16495809	MIR6889					
cg04424420	MIR6889					
cg15399181	MIR6889					

cg21872942	MIR33A					
cg24260710	MIR33A					
cg26207035	MIR33A					
cg17473398	MIR33A					
cg04181153	MIR33A					
cg02233213	MIR33A					
cg12249359	MIR33A					
cg13521944	MIR1249					
cg14696444	MIR1249					
cg21407354	MIR1249					
cg08378742	MIR1249					
cg13766329	MIR1249					
cg02144647	MIR1249					
cg05345310	MIR1249					
cg21331510	MIR1249					
cg24352349	MIR1249					
cg04880355	MIR1249					
cg23408471	MIRLET7BHG					
cg14666926	MIR3619	MIRLET7BHG				
cg01272212	MIRLET7BHG					
cg26371705	MIRLET7A3					
cg08695558	MIRLET7A3					
cg15702185	MIRLET7A3					
cg24911721	MIRLET7A3	MIRLET7B				
cg13251842	MIRLET7A3	MIRLET7B				
cg03841312	MIRLET7A3	MIRLET7B				
cg04063235	MIRLET7A3	MIRLET7B				
cg22121941	MIRLET7A3	MIRLET7B				
cg16114706	MIRLET7B					
cg06720082	MIRLET7B					
cg08263387	MIRLET7B					
cg20967343	MIRLET7B					
cg09096159	MIRLET7B					
cg25559898	MIRLET7B					
cg09937725	MIR3667					
cg15944251	MIR4767					
cg15157289	MIR221	MIR222				
cg14358077	MIR221	MIR222				
cg01845432	MIR4769					
cg11590435	MIR532	MIR188				
cg20963460	MIR532	MIR188				
cg16879820	MIR188	MIR532				
cg26715986	MIR188					
cg03662899	MIR188					
cg01843978	MIR362	MIR500				
cg07452499	MIR362	MIR500				
cg27463946	MIR362	MIR500				
cg10774723	MIR362	MIR500				
cg08207582	MIR362	MIR500	MIR501			
cg08342708	MIR501					

cg14313347	MIR501					
cg08709097	MIR500B					
cg15073264	MIR660					
cg21664443	MIR502	MIR660				
cg05193141	MIR502	MIR660				
cg18123612	MIR502					
cg24877449	MIR502					
cg12625872	MIR502					
cg00582864	MIR502					
cg21206555	MIR502					
cg12198632	MIR6895					
cg01530912	MIR6895					
cg18166152	MIR98					
cg07809144	MIR98					
cg05453618	MIR98	MIRLET7F2				
cg06308344	MIR98	MIRLET7F2				
cg15648245	MIRLET7F2					
cg27313362	MIRLET7F2					
cg21030907	MIRLET7F2					
cg22414759	MIR4536-2	MIR4536-1				
cg15002839	MIR4536-1					
cg00965199	MIR4536-1					
cg20552852	MIR676					
cg01528835	MIR421					
cg07195705	MIR421					
cg01874426	MIR374B	MIR421				
cg01829822	MIR374B	MIR421				
cg15376401	MIR374B	MIR421				
cg00763315	MIR374B	MIR421				
cg01076406	MIR374B	MIR421				
cg17674548	MIR374A	MIR545				
cg20598843	MIR374A	MIR545				
cg22690030	MIR374A	MIR545				
cg26533737	MIR374A	MIR545				
cg06399342	MIR545	MIR374A				
cg15401363	MIR325HG					
cg02823603	MIR325HG					
cg00118309	MIR384	MIR325HG				
cg26740830	MIR325HG					
cg06845581	MIR325	MIR325HG				
cg26366521	MIR325	MIR325HG				
cg09372698	MIR361					
cg05806250	MIR361					
cg23510372	MIR361					
cg02921294	MIR1256					
cg13933773	MIR1256					
cg17192679	MIR1256					
cg12116566	MIR1256					
cg19434750	MIR1256					
cg23628411	MIR1256					

cg20063407	MIR1256					
cg26553525	MIR1256					
cg12647861	MIR1256					
cg14353569	MIR1256					
cg01821906	MIR1256					
cg06159667	MIR1256					
cg11188837	MIR1256					
cg13214067	MIR1256					
cg00190642	MIR1256					
cg06885779	MIR1256					
cg10871818	MIR1256					
cg22952287	MIR1256					
cg07779826	MIR1256					
cg03090449	MIR1256					
cg01404374	MIR1256					
cg24602572	MIR1256					
cg12273094	MIR1256					
cg08145178	MIR1256					
cg02746718	MIR1256					
cg10984692	MIR1256					
cg25250241	MIR1256					
cg01314576	MIR652					
cg07277490	MIR3978					
cg03700990	MIR4329					
cg24489774	MIR764					
cg08096166	MIR764					
cg05574878	MIR1912					
cg11163995	MIR1912					
cg18784552	MIR1912					
cg08981801	MIR1298					
cg27477134	MIR1298					
cg18153989	MIR1298					
cg23848666	MIR1911					
cg16255697	MIR1911					
cg10997274	MIR1911					
cg00694968	MIR1911					
cg10199730	MIR448					
cg06129596	MIR448					
cg20344820	MIR448					
cg06750118	MIR1277					
cg06534952	MIR1277					
cg21288474	MIR1277					
cg20486690	MIR766					
cg16296431	MIR766					
cg25265343	MIR766					
cg25221323	MIR363	MIR92A2				
cg08261807	MIR20B	MIR363	MIR106A	MIR18B	MIR19B2	MIR92A2
cg13272357	MIR20B	MIR363	MIR18B	MIR106A	MIR19B2	MIR92A2
cg08585558	MIR20B	MIR363	MIR18B	MIR106A	MIR19B2	MIR92A2
cg19791341	MIR450A1	MIR450B				

cg22449592	MIR450B	MIR450A1				
cg12030638	MIR450A1	MIR450B	MIR450A2			
cg02505036	MIR450A1	MIR450B	MIR542	MIR450A2		
cg24923927	MIR450A1	MIR450B	MIR542	MIR450A2		
cg20978230	MIR503					
cg01972979	MIR503					
cg07194250	MIR503					
cg22955387	MIR503					
cg07776419	MIR503					
cg23245720	MIR503					
cg18412777	MIR424	MIR503				
cg01807688	MIR424	MIR503				
cg07924363	MIR424	MIR503				
cg18719157	MIR424	MIR503				
cg08141518	MIR424	MIR503				
cg22943827	MIR503	MIR503HG	MIR424			
cg07911140	MIR934					
cg00966524	MIR934					
cg15380873	MIR934					
cg00583858	MIR934					
cg06268905	MIR934					
cg02994237	MIR504					
cg06949232	MIR504					
cg08287343	MIR504					
cg26551791	MIR888	MIR890				
cg00739582	MIR888	MIR890				
cg07029395	MIR892B	MIR892A				
cg19139671	MIR892A	MIR892B				
cg10647025	MIR892A	MIR892B				
cg03829195	MIR892A	MIR892B				
cg05091313	MIR892A	MIR892B				
cg16319308	MIR507	MIR506				
cg22976112	MIR507	MIR506				
cg27312241	MIR507	MIR506				
cg02634141	MIR507	MIR506				
cg09721969	MIR507	MIR506				
cg26404952	MIR509-2	MIR509-1	MIR509-3			
cg13890773	MIR224					
cg16618605	MIR452					
cg15341392	MIR224	MIR452				
cg00018204	MIR452					
cg14686949	MIR452					
cg14017986	MIR105-1					
cg18471285	MIR105-1					
cg18904817	MIR105-1					
cg11166197	MIR105-1					
cg12182101	MIR105-1					
cg04227631	MIR767	MIR105-1				
cg07311296	MIR767	MIR105-2				
cg11428427	MIR105-2					

cg18312428	MIR718					
cg27167979	MIR718					
cg03050491	MIR718					
cg19572242	MIR718					
cg27616996	MIR718					
cg16746961	MIR718					
cg07910434	MIR6858					
cg12159336	MIR664B					
cg13449535	MIR200B					
cg07123481	MIR200B					
cg26330479	MIR429					
cg06967105	MIR429					
cg23651812	MIR429					
cg03528302	MIR429					
cg00909706	MIR34A					
cg10640389	MIR34A					
cg09994773	MIR34A					
cg24501230	MIR34A					
cg22904086	MIR3675					
cg13252549	MIR3675					
cg03698781	MIR552					
cg18983289	MIR3659					
cg16682693	MIR101-1					
cg18222371	MIR101-1					
cg16820411	MIR101-1					
cg14871564	MIR101-1					
cg00405232	MIR760					
cg17835369	MIR760					
cg02993630	MIR760					
cg19827167	MIR760					
cg03327164	MIR760					
cg04938255	MIR760					
cg25107717	MIR760					
cg17992509	MIR760					
cg16383389	MIR760					
cg15641184	MIR137HG					
cg22090860	MIR137HG					
cg03226624	MIR137HG					
cg23243552	MIR137HG					
cg04293733	MIR137					
cg22333214	MIR137					
cg05423529	MIR137					
cg10489614	MIR137					
cg26390542	MIR137HG					
cg20713662	MIR137HG					
cg09704056	MIR137HG					
cg11515530	MIR197					
cg25105745	MIR197					
cg19321304	MIR320B1					
cg05156532	MIR320B1					

cg04390865	MIR320B1					
cg18274788	MIR320B1					
cg20632224	MIR320B1					
cg12600901	MIR190B					
cg21536086	MIR190B					
cg12640942	MIR190B					
cg24880818	MIR190B					
cg18645493	MIR190B					
cg27538026	MIR92B					
cg15434337	MIR92B					
cg12678006	MIR92B					
cg03425468	MIR92B					
cg09952954	MIR92B					
cg09417889	MIR92B					
cg06906462	MIR557					
cg11157034	MIR557					
cg04436701	MIR557					
cg05212543	MIR557					
cg06533233	MIR548F1					
cg25229253	MIR548F1					
cg23208294	MIR548F1					
cg19239783	MIR548F1					
cg19261968	MIR548F1					
cg06638723	MIR548F1					
cg08470408	MIR548F1					
cg27238955	MIR181A1HG					
cg13758814	MIR181A1HG					
cg12343554	MIR181A1HG					
cg02140051	MIR181A1HG					
cg12496806	MIR181A1HG					
cg07895006	MIR181A1HG					
cg06774144	MIR181A1HG					
cg23994787	MIR181A1HG					
cg01196224	MIR181A1HG					
cg13935040	MIR181A1HG					
cg23989838	MIR181A1HG					
cg07370431	MIR181A1HG					
cg22821358	MIR181B1					
cg01092932	MIR181B1					
cg25349643	MIR181B1					
cg08866945	MIR181A1HG					
cg22898559	MIR181A1HG					
cg15265199	MIR181A1HG					
cg03386320	MIR181A1HG					
cg24379085	MIR181A1HG					
cg11984368	MIR181A1HG					
cg09398207	MIR181A1HG					
cg18818801	MIR181A1HG					
cg23828146	MIR181A1HG					
cg14441312	MIR181A1HG					

cg04876417	MIR181A1HG					
cg05619215	MIR181A1HG					
cg25264507	MIR181A1HG					
cg25249290	MIR181A1HG					
cg26158447	MIR181A1HG					
cg21517258	MIR181A1HG					
cg09702859	MIR181A1HG					
cg18391344	MIR181A1HG					
cg02999711	MIR135B					
cg25202407	MIR135B					
cg02520707	MIR135B					
cg13061767	MIR135B					
cg22159815	MIR29C					
cg10234282	MIR29C					
cg20701901	MIR29C					
cg08855249	MIR29C					
cg13093042	MIR29C					
cg15076217	MIR29C					
cg22525895	MIR29B2					
cg03446399	MIR29B2					
cg18591304	MIR205HG					
cg22688137	MIR548F3					
cg09938677	MIR548F3					
cg08068800	MIR548F3					
cg10965259	MIR548F3					
cg15644668	MIR548F3					
cg25064770	MIR548F3					
cg17864399	MIR548F3					
cg05346981	MIR548F3					
cg04517512	MIR548F3					
cg22432250	MIR548F3					
cg25126186	MIR548F3					
cg02868743	MIR548F3					
cg10384855	MIR548F3					
cg12045277	MIR548F3					
cg03904929	MIR548F3					
cg06878166	MIR548F3					
cg14959721	MIR548F3					
cg12123924	MIR548F3					
cg13327797	MIR548F3					
cg04435511	MIR548F3					
cg16015207	MIR548F3					
cg24541641	MIR548F3					
cg14378539	MIR548F3					
cg07011633	MIR548F3					
cg01359438	MIR548F3					
cg12035766	MIR548F3					
cg22064155	MIR548F3					
cg25580383	MIR548F3					
cg25897426	MIR548F3					

cg10587334	MIR548F3					
cg10695606	MIR548F3					
cg11318947	MIR548F3					
cg00837485	MIR548F3					
cg21581501	MIR548F3					
cg24289256	MIR548F3					
cg12123269	MIR548F3					
cg15791171	MIR548F3					
cg21587439	MIR548F3					
cg19088961	MIR548F3					
cg14476293	MIR548F3					
cg09629734	MIR548F3					
cg11774116	MIR548F3					
cg00514003	MIR548F3					
cg18938479	MIR548F3					
cg13739348	MIR548F3					
cg15548099	MIR548F3					
cg18008865	MIR548F3					
cg15932059	MIR548F3					
cg25509259	MIR548F3					
cg18233317	MIR548F3					
cg22725112	MIR548F3					
cg25469009	MIR548F3					
cg20485469	MIR548F3					
cg07494702	MIR548F3					
cg18217102	MIR548F3					
cg24115397	MIR548F3					
cg24909927	MIR548F3					
cg08710520	MIR548F3					
cg02229781	MIR548F3					
cg13391521	MIR548F3					
cg20576928	MIR548F3					
cg19827372	MIR548F3					
cg20331458	MIR548F3					
cg09568717	MIR5096					
cg19770278	MIR5096					
cg06067303	MIR5096					
cg23896919	MIR5096					
cg06190257	MIR5096					
cg01297352	MIR5096					
cg13917775	MIR1273E					
cg21086541	MIR1273E					
cg17479598	MIR1273E					
cg24720069	MIR1273E					
cg02805410	MIR1273E					
cg00814997	MIR1273E					
cg16520626	MIR1273E					
cg12787622	MIR1273E					
cg23787364	MIR1273E					
cg27006666	MIR1273E					

cg21743698	MIR1273E					
cg02559101	MIR1273E					
cg13489860	MIR1273E					
cg25922934	MIR1273E					
cg06707840	MIR7515HG					
cg27234853	MIR7515HG					
cg04969551	MIR7515HG					
cg06647133	MIR7515HG					
cg22601570	MIR7515HG					
cg00164078	MIR7515HG					
cg06664913	MIR7515HG					
cg02094460	MIR4262					
cg18672759	MIR4757					
cg02511750	MIR4757					
cg13618190	MIR217					
cg14237297	MIR217HG					
cg07641563	MIR216A					
cg25249448	MIR216A					
cg24343835	MIR216A					
cg12686754	MIR216A					
cg20102086	MIR216A					
cg05466976	MIR216A					
cg01160855	MIR216B					
cg02071553	MIR216B					
cg09585004	MIR217HG					
cg25035474	MIR217HG					
cg23936106	MIR217HG					
cg09860381	MIR217HG					
cg14593650	MIR217HG					
cg16879857	MIR217HG					
cg24980686	MIR217HG					
cg06815127	MIR217HG					
cg13761242	MIR217HG					
cg03672322	MIR4433B					
cg10020120	MIR548AU					
cg05617050	MIR548AU					
cg20777186	MIR4435-2HG					
cg15327395	MIR4435-2HG					
cg14513117	MIR3679					
cg16321483	MIR3679					
cg24000528	MIR10B					
cg23460578	MIR10B					
cg19519747	MIR10B					
cg01093934	MIR1246					
cg02049472	MIR1246					
cg20841588	MIR1246					
cg13236271	MIR1246					
cg16588417	MIR1246					
cg17766381	MIR548N					
cg04027132	MIR548N					

cg17201638	MIR548N					
cg26902777	MIR548N					
cg05147708	MIR548N					
cg20587874	MIR548N					
cg19648023	MIR548N					
cg03808674	MIR548N					
cg11948456	MIR548N					
cg27078812	MIR548N					
cg20885078	MIR548N					
cg01656221	MIR548N					
cg16297938	MIR548N					
cg06151464	MIR548N					
cg07457402	MIR548N					
cg12885549	MIR548N					
cg01105521	MIR548N					
cg23238438	MIR548N					
cg00074313	MIR548N					
cg25064130	MIR548N					
cg05781812	MIR548N					
cg19916659	MIR548N					
cg17740434	MIR548N					
cg10580067	MIR1302-4					
cg00941833	MIR1302-4					
cg22306928	MIR375					
cg00215432	MIR375					
cg00218620	MIR375					
cg00705280	MIR375					
cg21615583	MIR375					
cg14358282	MIR375					
cg04348419	MIR375					
cg02257674	MIR375					
cg18094076	MIR1471					
cg01058850	MIR1471					
cg12035782	MIR1471					
cg25883149	MIR1471					
cg06046580	MIR1471					
cg07513814	MIR1471					
cg09110634	MIR1471					
cg02797108	MIR4790					
cg20512713	MIR563					
cg12698662	MIR563					
cg06368401	MIR563					
cg23023604	MIR563					
cg22821220	MIR563					
cg00222455	MIR548AC					
cg21604742	MIR548AC					
cg00400494	MIR548AC					
cg05288397	MIR548AC					
cg15168942	MIR4792					
cg13423180	MIR4792					

cg11697712	MIR4442					
cg12488577	MIR4442					
cg10708972	MIR138-1					
cg07997066	MIR138-1					
cg06209298	MIR138-1					
cg03322633	MIR138-1					
cg12428299	MIR138-1					
cg03918530	MIR138-1					
cg20687414	MIR138-1					
cg05269632	MIR135A1					
cg18675902	MIR135A1					
cg23078228	MIR548A2					
cg09603919	MIR548A2					
cg04522625	MIR548A2					
cg25366141	MIR548A2					
cg13519595	MIR548A2					
cg21324884	MIR548A2					
cg18697991	MIR548A2					
cg05952841	MIR548A2					
cg05509179	MIR548A2					
cg05971592	MIR548A2					
cg05684371	MIR1324					
cg10563834	MIR1324					
cg26374481	MIR548G					
cg01726038	MIR548G					
cg13475155	MIR548G					
cg06671868	MIR548G					
cg12256747	MIR548G					
cg07866371	MIR548G					
cg21341558	MIR548G					
cg00213334	MIR548G					
cg24757159	MIR548G					
cg02145866	MIR548G					
cg24426290	MIR548G					
cg05026437	MIR548G					
cg03851861	MIR548G					
cg21938179	MIR548A3					
cg04885775	MIR568					
cg08723913	MIR568					
cg16358924	MIR568					
cg02771886	MIR568					
cg17164827	MIR568					
cg24727662	MIR548H2					
cg10231332	MIR548H2					
cg03040807	MIR548H2					
cg13846359	MIR548H2					
cg09306252	MIR548H2					
cg18682381	MIR548H2					
cg27114354	MIR548H2					
cg14083040	MIR548H2					

cg11037184	MIR548H2					
cg00258015	MIR548H2					
cg09825167	MIR548H2					
cg21090893	MIR548H2					
cg24502614	MIR1263					
cg19110521	MIR1263					
cg19550890	MIR7977					
cg06277638	MIR570					
cg16575892	MIR378D1					
cg21836699	MIR548I2					
cg27663716	MIR548I2					
cg08174890	MIR548I2					
cg25206536	MIR572					
cg04203702	MIR572					
cg06606539	MIR572					
cg25960403	MIR572					
cg14880874	MIR548AJ2					
cg04185799	MIR548AJ2					
cg13716848	MIR548AJ2					
cg07162914	MIR548AJ2					
cg25734769	MIR548AJ2					
cg27343278	MIR548AJ2					
cg12112338	MIR548AJ2					
cg02798211	MIR548AJ2					
cg08450323	MIR548AJ2					
cg17949025	MIR548AJ2					
cg18363417	MIR573					
cg08515869	MIR573					
cg10807961	MIR1273H					
cg10870267	MIR1273H					
cg14201734	MIR1273H					
cg06923767	MIR1273H					
cg24778568	MIR1273H					
cg15350946	MIR1273H					
cg00948209	MIR1273H					
cg12386065	MIR1273H					
cg18348975	MIR1273H					
cg09454295	MIR1273H					
cg03843023	MIR1273H					
cg21179425	MIR1273H					
cg05878519	MIR1273H					
cg01902584	MIR1273H					
cg10783197	MIR1273H					
cg20548068	MIR1273H					
cg27064284	MIR1273H					
cg03585049	MIR1273H					
cg08396445	MIR7641-2					
cg16473384	MIR7641-2					
cg14131313	MIR1269A					
cg25521375	MIR1269A					

cg04100549	MIR1269A					
cg16326188	MIR4452					
cg09442106	MIR5705					
cg23666362	MIR1973					
cg23740016	MIR1973					
cg11976164	MIR2054					
cg13332552	MIR2054					
cg22576711	MIR2054					
cg03976364	MIR5684					
cg03949274	MIR5684					
cg19277411	MIR5684					
cg12318602	MIR5684					
cg21687237	MIR5684					
cg14294892	MIR5684					
cg03529040	MIR5684					
cg08570631	MIR5684					
cg10123619	MIR6082					
cg23107222	MIR4276					
cg11677212	MIR1305					
cg13300024	MIR1305					
cg12655094	MIR1305					
cg02223783	MIR3945					
cg08827075	MIR3945					
cg04251661	MIR4280					
cg11833735	MIR4280					
cg05367489	MIR3660					
cg22581536	MIR548AO					
cg09753632	MIR583					
cg04481923	MIR886					
cg18678645	MIR886					
cg06536614	MIR886					
cg25340688	MIR886					
cg26896946	MIR886					
cg00124993	MIR886					
cg08745965	MIR886					
cg18797653	MIR886					
cg20921808	MIR5197					
cg23915659	MIR1303					
cg06201287	MIR1303					
cg11127866	MIR1303					
cg20179863	MIR1303					
cg01305537	MIR1303					
cg16901161	MIR3142					
cg17120588	MIR146A					
cg08350739	MIR146A					
cg03306240	MIR146A					
cg23066234	MIR4634					
cg22827729	MIR7641-2					
cg22134024	MIR7641-2					
cg26601590	MIR548A1					

cg03915569	MIR548A1					
cg03000711	MIR548A1					
cg07725389	MIR219-1					
cg01910419	MIR219-1					
cg18007341	MIR1275					
cg03903398	MIR1275					
cg01956472	MIR1275					
cg04259565	MIR1275					
cg12974668	MIR1275					
cg25360535	MIR1275					
cg02074259	MIR3925					
cg03820148	MIR4462					
cg11746148	MIR4462					
cg05491093	MIR4462					
cg14701682	MIR206					
cg03899824	MIR206					
cg13693143	MIR206					
cg22887663	MIR206					
cg15671688	MIR206					
cg22720139	MIR206					
cg26962533	MIR206					
cg25284213	MIR206					
cg06903579	MIR133B					
cg14487577	MIR133B					
cg03824238	MIR133B					
cg10488199	MIR133B					
cg14351526	MIR30C2					
cg25566787	MIR30C2					
cg25306883	MIR30C2					
cg15319027	MIR30C2					
cg19098437	MIR30A					
cg20815778	MIR30A					
cg26053571	MIR30A					
cg01434892	MIR30A					
cg21303169	MIR30A					
cg16506910	MIR30A					
cg16152480	MIR4643					
cg09111718	MIR548H3					
cg01823120	MIR548H3					
cg27442580	MIR548H3					
cg19521384	MIR548H3					
cg25199591	MIR2113					
cg06138966	MIR2113					
cg09836205	MIR2113					
cg06081793	MIR5695					
cg01979460	MIR588					
cg19582000	MIR588					
cg25760888	MIR548H5					
cg15974831	MIR4465					
cg04675191	MIR4465					

cg15808266	MIR1273C					
cg16076191	MIR1202					
cg10494330	MIR1202					
cg07184013	MIR148A					
cg25771013	MIR148A					
cg03853208	MIR148A					
cg16291657	MIR148A					
cg09596116	MIR148A					
cg12693702	MIR148A					
cg00399596	MIR148A					
cg16447012	MIR196B					
cg18311537	MIR196B					
cg15912800	MIR196B					
cg26259537	MIR196B					
cg05250768	MIR196B					
cg01024168	MIR196B					
cg05027336	MIR196B					
cg08964780	MIR196B					
cg25644556	MIR196B					
cg24884519	MIR196B					
cg08362210	MIR196B					
cg10265254	MIR550A2					
cg04840800	MIR129-1					
cg24044186	MIR129-1					
cg09344485	MIR129-1					
cg11364290	MIR129-1					
cg04524088	MIR129-1					
cg20734982	MIR129-1					
cg24423782	MIR182					
cg17677032	MIR182					
cg04579608	MIR182					
cg13713066	MIR182					
cg08755972	MIR182					
cg09787315	MIR182					
cg00477302	MIR182					
cg09537448	MIR96					
cg13374528	MIR96					
cg09388414	MIR96					
cg24616828	MIR96					
cg03446876	MIR96					
cg08100535	MIR96					
cg11370011	MIR29A					
cg10758022	MIR29A					
cg07558442	MIR29A					
cg02000032	MIR29B1					
cg23380552	MIR5707					
cg01912428	MIR5707					
cg08380316	MIR5707					
cg26430142	MIR3674					
cg22461390	MIR596					

cg06742077	MIR596					
cg17937101	MIR596					
cg05650680	MIR596					
cg25673948	MIR596					
cg11998141	MIR596					
cg09177567	MIR596					
cg09899173	MIR596					
cg12142501	MIR596					
cg15821939	MIR596					
cg16481961	MIR596					
cg25295395	MIR4660					
cg20239358	MIR3148					
cg05216534	MIR548O2					
cg13550493	MIR548O2					
cg16657582	MIR548O2					
cg13435938	MIR548O2					
cg08677259	MIR548O2					
cg22666787	MIR548O2					
cg19496363	MIR1268A					
cg19578234	MIR1268A					
cg14684255	MIR1268A					
cg04424780	MIR1268A					
cg12650829	MIR1268A					
cg18616114	MIR1268A					
cg14420620	MIR1268A					
cg02356258	MIR1268A					
cg03287314	MIR1268A					
cg16047262	MIR1268A					
cg21978026	MIR1268A					
cg10280702	MIR1268A					
cg01272629	MIR1268A					
cg16922635	MIR1268A					
cg03340334	MIR1268A					
cg02249596	MIR1268A					
cg00791136	MIR1268A					
cg05564644	MIR1268A					
cg14739038	MIR1268A					
cg11334241	MIR1268A					
cg18560789	MIR1268A					
cg09661621	MIR1268A					
cg15083435	MIR1268A					
cg17528453	MIR1268A					
cg06162386	MIR1268A					
cg26413319	MIR1268A					
cg18699598	MIR1268A					
cg04460239	MIR1268A					
cg25239439	MIR1268A					
cg12424249	MIR1268A					
cg16008467	MIR1268A					
cg06967452	MIR1268A					

cg13352986	MIR1268A					
cg10698928	MIR124-2					
cg07792478	MIR124-2					
cg14590098	MIR124-2					
cg02226645	MIR124-2					
cg04104463	MIR124-2					
cg25900085	MIR124-2					
cg05474726	MIR124-2					
cg27313642	MIR124-2					
cg16189671	MIR124-2					
cg20653075	MIR124-2					
cg05455720	MIR124-2					
cg04559779	MIR124-2					
cg06610119	MIR2052HG					
cg26665229	MIR2052HG					
cg17580326	MIR2052HG					
cg16955780	MIR2052HG					
cg07709181	MIR2052HG					
cg13025694	MIR2052HG					
cg08157118	MIR2052HG					
cg01405527	MIR2052HG					
cg06276439	MIR2052HG					
cg07035704	MIR2052					
cg26754262	MIR2052					
cg21856508	MIR2052HG					
cg06014707	MIR2052HG					
cg11288819	MIR5708					
cg12286210	MIR7641-2					
cg04845520	MIR7641-2					
cg19776344	MIR3150B					
cg20238164	MIR3150B					
cg18549594	MIR4471					
cg12834926	MIR4662B					
cg24032265	MIR1204					
cg13698548	MIR1204					
cg22384423	MIR1204					
cg11792826	MIR1208					
cg24465482	MIR1208					
cg00444740	MIR1208					
cg07018107	MIR1208					
cg05425602	MIR30B					
cg20831094	MIR30B					
cg01417339	MIR30B					
cg25964744	MIR30B					
cg03331035	MIR30B					
cg16657434	MIR30D					
cg01609214	MIR30D					
cg18975690	MIR30D					
cg07773515	MIR31HG					
cg21445603	MIR31HG					

cg16512376	MIR31HG					
cg23976185	MIR31HG					
cg20963107	MIR31HG					
cg07955015	MIR31HG					
cg01599028	MIR31HG					
cg00632587	MIR31HG					
cg09008353	MIR4475					
cg05099385	MIR6130					
cg19344277	MIR6130					
cg24178514	MIR6130					
cg06932691	MIR6130					
cg11931203	MIR6130					
cg14282114	MIR6130					
cg08956415	MIR6130					
cg17768313	MIR6130					
cg03870472	MIR6130					
cg21021332	MIR6130					
cg00932528	MIR548H3					
cg02398371	MIR548H3					
cg15788451	MIR548H3					
cg11998579	MIR548H3					
cg14449524	MIR548H3					
cg16055294	MIR548H3					
cg02582781	MIR548H3					
cg03121659	MIR548H3					
cg02558684	MIRLET7A1					
cg09902908	MIRLET7F1					
cg03275648	MIRLET7D					
cg03016486	MIR8081					
cg13781167	MIR8081					
cg05205299	MIR548Q					
cg14588738	MIR548Q					
cg08995067	MIR7702					
cg08881769	MIR3134					
cg07738306	MIR3134					
cg12776892	MIR3134					
cg14478242	MIR3134					
cg12935627	MIR3134					
cg11255687	MIR3134					
cg21025168	MIR3134					
cg07815919	MIR3134					
cg24795721	MIR3134					
cg06624369	MIR3134					
cg13644295	MIR3134					
cg04291471	MIR3134					
cg16843250	MIR3134					
cg03757043	MIR3134					
cg20155849	MIR3134					
cg12834462	MIR3134					
cg02151068	MIR3134					

cg22576265	MIR147					
cg09399225	MIR147					
cg01731518	MIR600HG					
cg13304325	MIR600HG					
cg14525247	MIR219-2					
cg10404717	MIR219-2					
cg20413392	MIR219-2					
cg14467654	MIR219-2					
cg00654637	MIR219-2					
cg13298616	MIR1268A					
cg20650753	MIR1268A					
cg19082696	MIR1268A					
cg06521718	MIR1268A					
cg00230342	MIR1268A					
cg11361720	MIR1268A					
cg24070801	MIR1268A					
cg10682141	MIR1268A					
cg06311238	MIR1268A					
cg01381219	MIR1268A					
cg07364026	MIR1268A					
cg11446824	MIR1268A					
cg14626525	MIR602					
cg00265277	MIR602					
cg21512799	MIR602					
cg11198094	MIR602					
cg06282561	MIR602					
cg00415455	MIR6072					
cg27438067	MIR6072					
cg14216723	MIR4293					
cg13959943	MIR1265					
cg22745143	MIR1265					
cg18372847	MIR1265					
cg08469305	MIR1265					
cg07191657	MIR1265					
cg21767615	MIR1265					
cg11651443	MIR8086					
cg17442723	MIR5100					
cg21452633	MIR4679-1					
cg11781069	MIR4679-2					
cg13860000	MIR607					
cg06527815	MIR607					
cg00695064	MIR607					
cg22883879	MIR607					
cg00991520	MIR607					
cg20517640	MIR607					
cg09701700	MIR146B					
cg25851152	MIR146B					
cg08437570	MIR146B					
cg05251190	MIR146B					
cg05858126	MIR146B					

cg15857661	MIR146B					
cg13442016	MIR146B					
cg23172333	MIR5694					
cg12661000	MIR5694					
cg20540672	MIR5694					
cg12454619	MIR5694					
cg23446857	MIR5694					
cg08789645	MIR5694					
cg08482145	MIR5694					
cg21759440	MIR5694					
cg07378239	MIR5694					
cg08512188	MIR5694					
cg10389108	MIR5694					
cg12761059	MIR5694					
cg11697653	MIR5694					
cg17378265	MIR5694					
cg02085369	MIR5694					
cg00130307	MIR5694					
cg06774207	MIR5694					
cg15675054	MIR5694					
cg23219905	MIR5694					
cg22532652	MIR5694					
cg00287908	MIR5694					
cg20361828	MIR5694					
cg11476463	MIR5694					
cg09683457	MIR5694					
cg23400579	MIR202					
cg11164162	MIR202					
cg19348206	MIR202					
cg05468584	MIR202					
cg09763180	MIR202					
cg14674124	MIR202					
cg13169484	MIR202					
cg14524334	MIR202					
cg07944707	MIR202					
cg16593552	MIR210HG					
cg03880841	MIR210					
cg15482500	MIR210					
cg27193691	MIR210					
cg01277369	MIR210					
cg07410811	MIR210					
cg05858042	MIR210					
cg08200293	MIR210					
cg02471760	MIR210					
cg25619191	MIR302E					
cg23225255	MIR4299					
cg26733645	MIR8070					
cg26039305	MIR670					
cg02791753	MIR670					
cg07552322	MIR670					

cg26024682	MIR670					
cg14294096	MIR670					
cg07616094	MIR670					
cg04458645	MIR670HG					
cg18514644	MIR670HG					
cg11074814	MIR129-2					
cg06372015	MIR129-2					
cg14901205	MIR129-2					
cg01514668	MIR129-2					
cg11638181	MIR129-2					
cg04355791	MIR129-2					
cg15556502	MIR129-2					
cg14416371	MIR129-2					
cg14944647	MIR129-2					
cg01939477	MIR129-2					
cg16407471	MIR129-2					
cg05376374	MIR129-2					
cg06114465	MIR4487					
cg20610740	MIR4487					
cg01681881	MIR130A					
cg00135888	MIR130A					
cg05082527	MIR130A					
cg23371208	MIR130A					
cg16520038	MIR130A					
cg10512089	MIR130A					
cg02258444	MIR192					
cg27083891	MIR192					
cg09349409	MIR192					
cg18262830	MIR192					
cg23266943	MIR194-2					
cg04238983	MIR612					
cg05363534	MIR612					
cg21144158	MIR612					
cg13167340	MIR612					
cg26122422	MIR4491					
cg15803103	MIR4491					
cg27291055	MIR100HG					
cg17381365	MIR100HG					
cg03732541	MIR100HG					
cg11191385	MIR100HG					
cg25169483	MIR100HG					
cg00655788	MIR100HG					
cg22870970	MIR100HG					
cg12256875	MIR100HG					
cg20804799	MIR100HG					
cg24493634	MIR100HG					
cg00049528	MIR100HG					
cg25444249	MIR100HG					
cg24749015	MIR100HG					
cg02093449	MIR100HG					

cg01577707	MIR100HG					
cg14584292	MIR100HG					
cg15871951	MIR100HG					
cg17681256	MIR100HG					
cg23684399	MIR100HG					
cg19049785	MIR100HG					
cg04803402	MIR4697HG					
cg16642299	MIR200C					
cg15426815	MIR200C					
cg23067082	MIR141					
cg19794481	MIR141					
cg18185189	MIR141					
cg12161331	MIR141					
cg02624246	MIR141					
cg20206204	MIR614					
cg05346831	MIR614					
cg24587625	MIR614					
cg05305603	MIR3974					
cg10662314	MIR196A2					
cg05412137	MIR196A2					
cg15215690	MIR196A2					
cg19607429	MIR196A2					
cg08053137	MIR196A2					
cg18771937	MIR196A2					
cg14679255	MIR196A2					
cg16400495	MIR196A2					
cg09796911	MIR1279					
cg20481419	MIR5692B					
cg15090644	MIR5700					
cg10549753	MIR492					
cg11212178	MIR492					
cg08922308	MIR492					
cg03407651	MIR492					
cg15862706	MIR492					
cg10525574	MIR492					
cg20220255	MIR492					
cg16667463	MIR331					
cg11690824	MIR331					
cg09995736	MIR331					
cg15028128	MIR135A2					
cg27280022	MIR135A2					
cg11030620	MIR135A2					
cg23912509	MIR135A2					
cg07287698	MIR135A2					
cg12638357	MIR1827					
cg13173541	MIR1827					
cg24232662	MIR1827					
cg05101019	MIR4496					
cg07883407	MIR4496					
cg17588091	MIR4496					

cg16911487	MIR1302-1					
cg00846136	MIR1302-1					
cg20470083	MIR1302-1					
cg08824012	MIR8072					
cg08761167	MIR548F5					
cg09505337	MIR548F5					
cg16041090	MIR548F5					
cg06531158	MIR548F5					
cg09063173	MIR548F5					
cg00805473	MIR548F5					
cg08871224	MIR548F5					
cg02390858	MIR548F5					
cg00339726	MIR548F5					
cg02636162	MIR548F5					
cg08613350	MIR548F5					
cg20371437	MIR548F5					
cg24757752	MIR548F5					
cg23483562	MIR320D1					
cg21794665	MIR320D1					
cg16430726	MIR320D1					
cg08715009	MIR320D1					
cg04565201	MIR759					
cg17843124	MIR759					
cg13767001	MIR759					
cg22750273	MIR759					
cg06529264	MIR1297					
cg11725835	MIR4500HG					
cg22296874	MIR4500HG					
cg15558727	MIR4500HG					
cg11440148	MIR4500HG					
cg01608962	MIR4500HG					
cg18554216	MIR622					
cg26399947	MIR548AN					
cg07832271	MIR548AN					
cg16428374	MIR548AN					
cg11432250	MIR548AS					
cg26889764	MIR548AI					
cg21903890	MIR548AI					
cg05693395	MIR548AI					
cg00687805	MIR548AI					
cg04427087	MIR548AI					
cg20017856	MIR548AI					
cg00873878	MIR548AI					
cg19491776	MIR6076					
cg13301249	MIR548AZ					
cg07939497	MIR548AZ					
cg06337700	MIR548AZ					
cg23671795	MIR4708					
cg01632188	MIR1260					
cg02577745	MIR493					

cg10940210	MIR493					
cg04714497	MIR493					
cg12785573	MIR493					
cg11346901	MIR493					
cg04802696	MIR493					
cg16762843	MIR493					
cg27039821	MIR665					
cg18804680	MIR665					
cg11887124	MIR665					
cg19945937	MIR665					
cg26255560	MIR431					
cg01971130	MIR431					
cg09873328	MIR370					
cg01339630	MIR370					
cg13760742	MIR370					
cg23388037	MIR370					
cg14072069	MIR370					
cg14866339	MIR379					
cg14440205	MIR379					
cg20562547	MIR379					
cg02691745	MIR411					
cg24768094	MIR380					
cg11780042	MIR329-2					
cg06961429	MIR329-2					
cg01131100	MIR494					
cg07429629	MIR494					
cg10400174	MIR494					
cg14148088	MIR494					
cg01244514	MIR494					
cg15862128	MIR494					
cg17341844	MIR494					
cg12823329	MIR494					
cg21548109	MIR1193					
cg04272133	MIR543					
cg01232479	MIR543					
cg18875631	MIR543					
cg05865548	MIR543					
cg12242174	MIR543					
cg18805031	MIR543					
cg10270238	MIR543					
cg21146428	MIR543					
cg10300864	MIR495					
cg00856952	MIR495					
cg26931307	MIR495					
cg04826652	MIR495					
cg02662576	MIR495					
cg21809913	MIR495					
cg27074221	MIR495					
cg14910227	MIR495					
cg02174748	MIR300					

cg10137253	MIR300					
cg14427563	MIR1185-1					
cg06392565	MIR1185-1					
cg10079327	MIR1185-1					
cg05216211	MIR1185-1					
cg02975060	MIR1185-2					
cg13192714	MIR1185-2					
cg13546609	MIR1185-2					
cg13677859	MIR381					
cg03290602	MIR889					
cg11005998	MIR655					
cg00647917	MIR655					
cg22971028	MIR655					
cg03862290	MIR655					
cg04223832	MIR655					
cg11116300	MIR655					
cg16438722	MIR487A					
cg01586072	MIR487A					
cg03019112	MIR487A					
cg00395657	MIR487A					
cg23399577	MIR487A					
cg03059138	MIR487A					
cg06746453	MIR487A					
cg07521789	MIR382					
cg03153765	MIR453					
cg00594228	MIR453					
cg23029159	MIR154					
cg19477205	MIR154					
cg14355941	MIR496					
cg02883666	MIR496					
cg19631264	MIR496					
cg26116969	MIR496					
cg15171962	MIR496					
cg21176597	MIR496					
cg11869269	MIR377					
cg20474788	MIR377					
cg03663955	MIR377					
cg05138957	MIR377					
cg11721554	MIR377					
cg22542859	MIR377					
cg14906110	MIR377					
cg18163364	MIR377					
cg22823821	MIR377					
cg11134801	MIR377					
cg10006887	MIR377					
cg10529599	MIR656					
cg04578631	MIR656					
cg23456330	MIR656					
cg16695570	MIR656					
cg04563422	MIR656					

cg06889454	MIR656					
cg16796899	MIR656					
cg09982291	MIR203A					
cg27314336	MIR203					
cg13673833	MIR203					
cg23960324	MIR203					
cg03401997	MIR203					
cg10059536	MIR203					
cg01520867	MIR203					
cg24454784	MIR203A					
cg06218726	MIR203					
cg21091128	MIR203					
cg25588576	MIR7641-2					
cg19481727	MIR7641-2					
cg14743553	MIR1268A					
cg16271911	MIR4510					
cg26431773	MIR4712					
cg22117079	MIR8067					
cg02587412	MIR422A					
cg19700150	MIR422A					
cg01542115	MIR422A					
cg12131601	MIR422A					
cg08116550	MIR422A					
cg01130777	MIR422A					
cg10302164	MIR548H4					
cg01280098	MIR548H4					
cg06049171	MIR548H4					
cg06022664	MIR548H4					
cg20238128	MIR548H4					
cg15773080	MIR548H4					
cg01154656	MIR548H4					
cg24305693	MIR548H4					
cg05079405	MIR548H4					
cg21938506	MIR548H4					
cg13330363	MIR548H4					
cg11155172	MIR548H4					
cg13323256	MIR548H4					
cg00187099	MIR548H4					
cg22540575	MIR548H4					
cg17429682	MIR548H4					
cg20347647	MIR548H4					
cg05093741	MIR548H4					
cg04198144	MIR548H4					
cg21260288	MIR548H4					
cg21155118	MIR548H4					
cg21274136	MIR548H4					
cg07987890	MIR548H4					
cg21963854	MIR548H4					
cg09727046	MIR548H4					
cg11926525	MIR548H4					

cg19539048	MIR548H4					
cg08413060	MIR548H4					
cg04028604	MIR548H4					
cg04673937	MIR548H4					
cg16774511	MIR548H4					
cg15311954	MIR548H4					
cg24212738	MIR548H4					
cg08039084	MIR548H4					
cg06506623	MIR548H4					
cg05451359	MIR548H4					
cg11098984	MIR548H4					
cg09638686	MIR548H4					
cg13348907	MIR548H4					
cg06916725	MIR548H4					
cg14753493	MIR548H4					
cg14470223	MIR548H4					
cg21635706	MIR548H4					
cg25987514	MIR548H4					
cg24248007	MIR548H4					
cg19856383	MIR548H4					
cg26553741	MIR548H4					
cg10191501	MIR548H4					
cg07286682	MIR548H4					
cg00362690	MIR548H4					
cg17480705	MIR548H4					
cg11644401	MIR548H4					
cg27417749	MIR548H4					
cg13227699	MIR630					
cg12877524	MIR4313					
cg15441999	MIR4313					
cg10815745	MIR5003					
cg10745413	MIR5003					
cg01207916	MIR184					
cg23121785	MIR184					
cg00722320	MIR184					
cg04947764	MIR184					
cg01397141	MIR184					
cg07873150	MIR4514					
cg21998967	MIR4514					
cg07741205	MIR1179					
cg08867933	MIR1179					
cg22144942	MIR1179					
cg02054724	MIR7-2					
cg18824446	MIR7-2					
cg09160955	MIR7-2					
cg23176340	MIR7-2					
cg01925965	MIR7-2					
cg09852187	MIR7-2					
cg22456251	MIR7-2					
cg00576773	MIR9-3					

cg02278768	MIR9-3					
cg01882870	MIR9-3					
cg25950235	MIR9-3					
cg21529323	MIR9-3					
cg12530503	MIR9-3					
cg03082580	MIR9-3					
cg13888600	MIR9-3					
cg03433313	MIR662					
cg22185428	MIR662					
cg09066676	MIR662					
cg16202509	MIR3677					
cg02528154	MIR940					
cg09195657	MIR940					
cg08318085	MIR940					
cg09452257	MIR940					
cg16869547	MIR940					
cg13882748	MIR8065					
cg00138641	MIR193B					
cg09918657	MIR193B					
cg10420310	MIR193B					
cg04018325	MIR193B					
cg06273075	MIR193B					
cg07665535	MIR193B					
cg21396064	MIR193B					
cg24229568	MIR365A					
cg09607047	MIR365-1					
cg22550658	MIR365A					
cg06916001	MIR365-1					
cg00928596	MIR365-1					
cg02533339	MIR365-1					
cg22388260	MIR365-1					
cg02886263	MIR365-1					
cg00677407	MIR1826					
cg05992347	MIR1826					
cg06617961	MIR1826					
cg11032038	MIR1826					
cg26563248	MIR1826					
cg08521332	MIR1826					
cg07685228	MIR5095					
cg13627776	MIR5095					
cg04973399	MIR5095					
cg12954379	MIR5095					
cg14691307	MIR5095					
cg12081759	MIR5095					
cg16824290	MIR5095					
cg24714883	MIR5095					
cg23491344	MIR5095					
cg19981865	MIR5095					
cg09839654	MIR5095					
cg17205193	MIR5095					

cg05815034	MIR5095					
cg12387740	MIR5095					
cg21590180	MIR5095					
cg10969470	MIR5095					
cg07917516	MIR5095					
cg07822388	MIR5095					
cg11318772	MIR5095					
cg17863326	MIR5095					
cg16912030	MIR5095					
cg09788217	MIR5095					
cg25084279	MIR5095					
cg05603440	MIR5095					
cg02714692	MIR5095					
cg19654612	MIR5095					
cg03815480	MIR5095					
cg01464247	MIR5095					
cg20361881	MIR5095					
cg02714331	MIR5095					
cg17273098	MIR5095					
cg21555172	MIR5095					
cg11525063	MIR5095					
cg16964357	MIR5095					
cg21006034	MIR5095					
cg09557888	MIR5095					
cg09758361	MIR5095					
cg02806824	MIR5095					
cg25562878	MIR5095					
cg24818690	MIR5095					
cg25024312	MIR5095					
cg08557347	MIR5095					
cg15556732	MIR5095					
cg05150619	MIR5095					
cg06448699	MIR5095					
cg06205938	MIR5095					
cg04885455	MIR5095					
cg15687179	MIR5095					
cg19147649	MIR5095					
cg26475094	MIR138-2					
cg02961808	MIR138-2					
cg19836088	MIR138-2					
cg07737292	MIR138-2					
cg18615472	MIR4720					
cg25694498	MIR4720					
cg02034330	MIR4720					
cg07476328	MIR5093					
cg05744073	MIR132					
cg12255698	MIR132					
cg19405854	MIR132					
cg25440818	MIR132					
cg05945782	MIR212					

cg00572843	MIR212					
cg27639133	MIR1253					
cg14311597	MIR1253					
cg07169873	MIR1253					
cg18614984	MIR1253					
cg09652807	MIR1253					
cg11235787	MIR195					
cg20446176	MIR497HG					
cg21283720	MIR497HG					
cg27648405	MIR497HG					
cg16944574	MIR497HG					
cg02968629	MIR497HG					
cg09805692	MIR451					
cg22700328	MIR193A					
cg21915970	MIR193A					
cg23892547	MIR193A					
cg20286236	MIR193A					
cg25571269	MIR193A					
cg22894896	MIR193A					
cg22533683	MIR193A					
cg24984384	MIR365-2					
cg07953150	MIR365-2					
cg01376318	MIR365-2					
cg26083576	MIR365-2					
cg24361571	MIR365-2					
cg06237697	MIR365-2					
cg21869027	MIR365-2					
cg21843277	MIR4734					
cg06769231	MIR2117					
cg02389634	MIR2117					
cg01165909	MIR2117					
cg03184776	MIR2117					
cg25687874	MIR2117					
cg07930121	MIR5089					
cg26072749	MIR10A					
cg26916621	MIR10A					
cg07631144	MIR10A					
cg15649236	MIR10A					
cg01572694	MIR10A					
cg14884929	MIR10A					
cg07625849	MIR10A					
cg13652985	MIR10A					
cg10432569	MIR196A1					
cg02329038	MIR196A1					
cg23941495	MIR196A1					
cg21958069	MIR196A1					
cg26608174	MIR196A1					
cg04735310	MIR196A1					
cg01452847	MIR196A1					
cg15035143	MIR142					

cg21232937	MIR142					
cg00057966	MIR142					
cg00176888	MIR142					
cg26112797	MIR142					
cg10530767	MIR142					
cg01951274	MIR142					
cg19466818	MIR142					
cg06471491	MIR21					
cg27023597	MIR21					
cg04276626	MIR21					
cg02515217	MIR21					
cg15759721	MIR21					
cg07181702	MIR21					
cg26974217	MIR548W					
cg00715363	MIR548W					
cg08124860	MIR548W					
cg09881857	MIR548W					
cg18630030	MIR548W					
cg00018216	MIR548W					
cg21717601	MIR548W					
cg14362952	MIR548W					
cg23247281	MIR548W					
cg26908257	MIR548W					
cg10914143	MIR548W					
cg12044703	MIR548W					
cg05738235	MIR548W					
cg09785144	MIR548W					
cg06592908	MIR548W					
cg11708912	MIR548W					
cg23018548	MIR548W					
cg00013702	MIR548W					
cg21573696	MIR548W					
cg04302567	MIR548W					
cg01465350	MIR548W					
cg05705892	MIR548W					
cg20751048	MIR548W					
cg17755509	MIR3976HG					
cg05112903	MIR3976HG					
cg18822271	MIR3976HG					
cg11678964	MIR3976					
cg18322658	MIR7153					
cg05827233	MIR187					
cg18981979	MIR187					
cg18720617	MIR187					
cg24369310	MIR187					
cg03599197	MIR187					
cg16673522	MIR187					
cg18268164	MIR187					
cg19312305	MIR122					
cg12907477	MIR122					

cg05249155	MIR122					
cg00481280	MIR122					
cg00287319	MIR3591					
cg15446050	MIR1268A					
cg00749454	MIR1268A					
cg18222066	MIR1268A					
cg21191470	MIR1268A					
cg14570348	MIR1268A					
cg03687936	MIR1268A					
cg23512763	MIR1268A					
cg15477257	MIR1268A					
cg09859911	MIR1268A					
cg23755509	MIR1268A					
cg11811349	MIR1268A					
cg15457725	MIR1268A					
cg00329803	MIR7-3HG					
cg05028773	MIR24-2					
cg08198483	MIR24-2					
cg16572540	MIR24-2					
cg25147193	MIR181C					
cg21153040	MIR181D					
cg18586440	MIR769					
cg16039972	MIR769					
cg01737592	MIR769					
cg21210537	MIR769					
cg03727337	MIR769					
cg18374625	MIR150					
cg15617950	MIR150					
cg27388703	MIR150					
cg06105296	MIR150					
cg05880105	MIR150					
cg03737367	MIR150					
cg27417567	MIR8074					
cg20643675	MIR1323					
cg17392201	MIR1323					
cg24128045	MIR1323					
cg26110907	MIR1323					
cg06625244	MIR1323					
cg01411759	MIR498					
cg22844368	MIR498					
cg10362294	MIR498					
cg22166633	MIR520E					
cg27352015	MIR520E					
cg15783452	MIR520E					
cg11179120	MIR519E					
cg23173647	MIR520F					
cg14113046	MIR520F					
cg10382967	MIR520F					
cg11835050	MIR519C					
cg01864361	MIR519C					

cg15930678	MIR519C					
cg09796029	MIR519C					
cg09318158	MIR1283-1					
cg18710710	MIR520A					
cg10753636	MIR520A					
cg23097843	MIR526B					
cg00047185	MIR526B					
cg16790055	MIR519B					
cg22537343	MIR525					
cg26325497	MIR525					
cg21592803	MIR525					
cg00054741	MIR523					
cg12395420	MIR518F					
cg17857791	MIR520B					
cg11251554	MIR518B					
cg15993786	MIR518B					
cg06445981	MIR518B					
cg09404289	MIR526A1					
cg14119827	MIR526A1					
cg18877961	MIR526A1					
cg13122532	MIR520C					
cg17670263	MIR520C					
cg24014849	MIR520C					
cg22725901	MIR518C					
cg21744136	MIR518C					
cg15616006	MIR518C					
cg16466613	MIR524					
cg23908943	MIR524					
cg27664418	MIR517A					
cg04573316	MIR519D					
cg24480735	MIR521-2					
cg08188609	MIR521-2					
cg00101104	MIR521-2					
cg19095219	MIR521-2					
cg25843705	MIR520D					
cg22921228	MIR520D					
cg03807917	MIR520G					
cg21456301	MIR520G					
cg18712351	MIR520G					
cg11461263	MIR520G					
cg13924954	MIR516B2					
cg04370182	MIR516B2					
cg24699132	MIR526A2					
cg18748580	MIR526A2					
cg15937784	MIR526A2					
cg24787924	MIR518E					
cg00109776	MIR518E					
cg03652429	MIR518D					
cg20467339	MIR518D					
cg10583119	MIR518D					

cg17129943	MIR516B1					
cg14209677	MIR516B1					
cg09430586	MIR518A2					
cg23061412	MIR518A2					
cg13600227	MIR521-1					
cg01574903	MIR522					
cg26757562	MIR519A1					
cg25349981	MIR519A1					
cg05382137	MIR527					
cg02700479	MIR527					
cg15604044	MIR516A1					
cg09852439	MIR516A1					
cg26242772	MIR1283-2					
cg16908824	MIR1283-2					
cg00182416	MIR516A2					
cg15471981	MIR373					
cg23072571	MIR373					
cg18291941	MIR663					
cg27138951	MIR663					
cg06007966	MIR663					
cg01521987	MIR663					
cg08304190	MIR663					
cg14345012	MIR663					
cg04150495	MIR663					
cg20395967	MIR663					
cg10715092	MIR663					
cg09687946	MIR7641-2					
cg08802738	MIR7641-2					
cg08095361	MIR7641-2					
cg01472578	MIR7641-2					
cg04542869	MIR7641-2					
cg21315017	MIR645					
cg09082617	MIR645					
cg09369136	MIR645					
cg06795741	MIR645					
cg13869899	MIR296					
cg14977365	MIR296					
cg25188071	MIR296					
cg05045228	MIR646HG					
cg18988219	MIR646HG					
cg10295671	MIR646HG					
cg18100581	MIR646HG					
cg05975176	MIR646HG					
cg03344985	MIR646HG					
cg10673290	MIR646HG					
cg10052396	MIR646HG					
cg17070748	MIR646HG					
cg08508541	MIR646HG					
cg05324597	MIR646HG					
cg11177296	MIR646HG					

cg27419751	MIR646HG					
cg27585830	MIR646HG					
cg17431446	MIR646HG					
cg09575157	MIR646HG					
cg07935832	MIR646HG					
cg07360763	MIR646HG					
cg23326334	MIR646HG					
cg00836031	MIR646HG					
cg16416232	MIR646HG					
cg05882929	MIR646HG					
cg08684257	MIR646HG					
cg02196420	MIR646HG					
cg01236281	MIR646HG					
cg13314718	MIR646HG					
cg19864886	MIR646HG					
cg12267218	MIR646HG					
cg02603007	MIR646HG					
cg17033471	MIR1257					
cg06048153	MIR1257					
cg03145994	MIR1257					
cg13991324	MIR1257					
cg19986126	MIR1257					
cg01273384	MIR1257					
cg08737296	MIR124-3					
cg02650317	MIR124-3					
cg02065637	MIR124-3					
cg04927004	MIR124-3					
cg15699267	MIR124-3					
cg20277905	MIR124-3					
cg19267861	MIR124-3					
cg03387135	MIR124-3					
cg01052879	MIR124-3					
cg18627360	MIR124-3					
cg15028514	MIR124-3					
cg06660530	MIR124-3					
cg18772588	MIR124-3					
cg25970929	MIR155HG					
cg00565412	MIR155HG					
cg24855498	MIR155HG					
cg00607521	MIR155HG					
cg19769982	MIR155HG					
cg07007506	MIR155HG					
cg17297071	MIR155HG					
cg23433889	MIR155HG					
cg12749863	MIR155HG					
cg14315558	MIR155HG					
cg03872783	MIR155HG					
cg07143733	MIR155HG					
cg10137287	MIR5009					
cg08634018	MIR5009					

cg17652718	MIR5009					
cg17938553	MIR5009					
cg10859636	MIR5009					
cg14197404	MIR5009					
cg23422270	MIR5009					
cg18986745	MIR5009					
cg08248790	MIR5009					
cg12787185	MIR5009					
cg12975010	MIR5009					
cg15200151	MIR5009					
cg14202820	MIR5009					
cg19115260	MIR5009					
cg00402042	MIR5009					
cg15886943	MIR5009					
cg11562659	MIR5009					
cg25446727	MIR5009					
cg03673191	MIR5009					
cg11714647	MIR5009					
cg18250881	MIR5009					
cg05452868	MIR5009					
cg03799971	MIR5009					
cg17888482	MIR5009					
cg02580944	MIR5009					
cg20186982	MIR5009					
cg03794154	MIR5009					
cg25118545	MIR5009					
cg12544812	MIR5009					
cg02868440	MIR5009					
cg05017902	MIR5009					
cg15424115	MIR5009					
cg08237735	MIR5009					
cg00224335	MIR4327					
cg09978395	MIR802					
cg01027226	MIR802					
cg12430252	MIR802					
cg18862975	MIR802					
cg22414262	MIR802					
cg00871610	MIR802					
cg21776091	MIR649					
cg16806458	MIR649					
cg04378107	MIR301B					
cg16974014	MIR130B					
cg03328201	MIR130B					
cg22797479	MIR650					
cg04765078	MIR650					
cg00379323	MIR650					
cg07604927	MIR5571					
cg10726463	MIR659					
cg18702576	MIR659					
cg08131204	MIR1281					

cg08353446	MIR378I					
cg12141088	MIR378I					
cg16269274	MIR3201					
cg17007012	MIR4535					
cg18703109	MIR651					
cg06453471	MIR651					
cg05940049	MIR7641-2					
cg25675072	MIR222					
cg22672977	MIR222					
cg12879539	MIR222					
cg00868980	MIR1468					
cg10794473	MIR1468					
cg08264124	MIR1468					
cg08464860	MIR1468					
cg06701191	MIR223					
cg19127840	MIR223					
cg13716034	MIR223					
cg25047306	MIR384					
cg23061351	MIR1256					
cg02216057	MIR1256					
cg04476849	MIR1256					
cg19628038	MIR1256					
cg05599348	MIR1256					
cg21562836	MIR1256					
cg08340885	MIR1256					
cg24965800	MIR6087					
cg08634657	MIR220A					
cg07653550	MIR220A					
cg05878968	MIR220A					
cg20454429	MIR363					
cg20788415	MIR106A					
cg24667168	MIR450B					
cg08303228	MIR542					
cg10796603	MIR542					
cg04630982	MIR505					
cg26326746	MIR505					
cg15056572	MIR505					
cg16719099	MIR505					
cg23192918	MIR505					
cg17488785	MIR320D2					
cg01963147	MIR320D2					
cg02060185	MIR892C					
cg01340286	MIR892C					
cg08291487	MIR890					
cg05776098	MIR890					
cg01651728	MIR890					
cg16380536	MIR888					
cg05175213	MIR888					
cg24853313	MIR892A					
cg27069588	MIR892A					

cg19878017	MIR892B					
cg15918587	MIR891B					
cg07927718	MIR891B					
cg27617163	MIR891B					
cg25696807	MIR891A					
cg08967287	MIR891A					
cg08085487	MIR891A					
cg13921680	MIR513C					
cg15186531	MIR513C					
cg02562693	MIR513C					
cg20343467	MIR513A1					
cg27167381	MIR506					
cg26218823	MIR508					
cg22262544	MIR508					
cg01374885	MIR508					
cg13154234	MIR508					
cg04037410	MIR514B					
cg08111264	MIR514B					
cg14583361	MIR514B					
cg05883757	MIR510					
cg23318169	MIR2114					
cg15033973	MIR2114					
cg21870760	MIR2114					
cg23570034	MIR2114					
cg23698976	MIR2114					
cg08316488	MIR2114					

· in very young
, Elisa Alonso3,

As regulated
NA is

MIR18A

