# Supplementary material for: Methylation deregulation of miRNA promoters identifies miR124-2 as a survival biomarker in Breast Cancer in very young women
Source: Sci Rep. 2018 Sep 26;8:14373. doi: 10.1038/s41598-018-32393-3 (PMC6158237; doi:10.1038/s41598-018-32393-3)
Supplement: Supplementary file 4 — Supplementary Table 4 [file 41598_2018_32393_MOESM4_ESM.pdf]

Methylation deregulation of miRNA promoters identifies miR124-2 as a survival biomarker in Breast Cancer in very young women. Sara S. Oltra1, Maria Peña-Chilet1, Victoria Vidal-Tomas 1, Kirsty Flower2, María Teresa Martinez1, Elisa Alonso3, Octavio Burgues3, Ana Lluch1¥, James M. Flanagan2, and Gloria Ribas1¥*

**Supplementary Table 4:** Table includes information about miRNAs and probes regulating them.

| miRNA | Probe number | Regulatory CpG Probes |
|---|---|---|
| MIR1268A | 293 | cg20734982, cg24423782, cg17677032, cg04579608, cg13713066, cg08755972, cg09787315, cg00477302, cg09537448, cg13374528, cg09388414, cg24616828, cg03446876, cg08100535, cg01877565, cg06242719, cg03104765, cg14794735, cg22047901, cg04819959, cg24037795, cg08761490, cg11370011, cg10758022, cg07558442, cg21434355, cg03856510, cg03776809, cg02000032, cg13396334, cg26312916, cg10242760, cg10979181, cg26188365, cg10493317, cg21200229, cg16486721, cg00710715, cg20052308, cg13192883, cg11769486, cg19139509, cg14355023, cg25429902, cg09335647, cg01615815, cg26636917, cg06880494, cg14627974, cg03436967, cg06927522, cg02515354, cg16341266, cg26548251, cg007911361, cg055646441, cg147390381, cg113342411, cg185607891, cg096616211, cg150834351, cg175284531, cg061623861, cg264133191, cg186995981, cg044602391, cg252394391, cg124242491, cg160084671, cg069674521, cg133529861, cg132986161, cg206507531, cg190826961, cg065217181, cg002303421, cg113617201, cg240708011, cg106821411, cg063112381, cg013812191, cg073640261, cg114468241, cg147435531, cg154460501, cg007494541, cg182220661, cg211914701, cg06855983, cg08310363, cg00716579, cg05324597, cg12241892, cg27395454, cg23669063, cg02452950, cg18297519, cg01839850, cg13641189, cg07699113, cg19070798, cg08519241, cg10470963, cg01182973, cg003149041, cg181612851, cg163212661, cg194963631, cg195782341, cg146842551, cg044247801, cg126508291, cg186161141, cg144206201, cg023562581, cg032873141, cg160472621, cg219780261, cg102807021, cg012726291, cg169226351, cg033403341, cg022495961, cg16494576, cg21376204, cg16543247, cg18222066, cg21191470, cg14570348, cg08000406, cg10825521, cg09556749, cg04119107, cg05034718, cg26837739, cg09505423, cg27053789, cg05916938, cg13795156, cg05048676, cg03656862, cg27328231, cg09002055, cg25472617, cg00611436, cg22302608, cg20693146, cg19072837, cg03687936, cg13811969, cg02990289, cg17550784, cg06606386, cg11429044, cg07706375, cg08528170, cg15787712, cg14145185, cg25147193, cg18986330, cg11840833, cg08694786, cg00527484, cg27569588, cg06910115, cg00912722, cg10527285, cg26157803, cg02540736, cg10597322, cg06846458, cg18186343, cg03039990, cg01022345, cg19509303, cg02577745, cg10940210, cg04714497, cg12785573, cg11346901, cg04802696, cg16762843, cg24044238, cg02022380, cg06165706, cg18340059, cg23546343, cg15516314, cg07087646, cg01316116, cg05295233, cg09319210, cg04936377, cg10502071, cg16164739, cg01846028, cg00407815, cg08253253, cg19225438, cg27386631, cg11658051, cg09077612, cg27513510, cg03357215, cg14325848, cg05623247, cg09666525, cg16192458, cg23671795, cg16424038, cg09934721, cg25845814, cg14257776, cg23674379, cg18421195, cg10993988, cg01632188, cg24006305, cg06883544, cg25952096, cg10134156, cg21095982, cg07843309, cg19929625, cg12264462, cg13622660, cg17191445, cg26671937, cg15358487, cg09552714, cg17002091, cg17198937, cg22507969, cg16975584, cg03184350, cg05013730, cg08374341, cg19796617, cg01722003, cg09981964, cg23998942, cg22556505, cg19686543, cg22735402, cg06755671, cg04720886, cg17868330, cg05640346, cg02756921, cg00860590, cg12616484, cg02572956, cg15529111, cg06083642, cg04382077, cg18479961, cg10832012, cg18476993, cg17548326, cg16477744, cg10310310, cg03361379, cg12779445, cg17527969, cg07968094, cg11763745, cg12414009, cg01123783, cg24990327, cg10688790, cg02373104, cg16595458, cg09362918, cg22807241, cg00105060, cg15578140, cg04486528, cg14739284, cg09597759, cg18970103, cg00826997, cg01067813, cg23548616, cg17096328, cg08260861, cg145703481, cg036879361, cg235127631, cg154772571, cg098599111, cg237555091, cg118113491, cg154577251 |
| MIR548H4 | 186 | cg22806002, cg18515591, cg22879515, cg21881253, cg13767940, cg26561785, cg01192900, cg24041078, cg08827001, cg05281603, cg09148270, cg12108074, cg09505361, cg18217031, cg20342998, cg02208900, cg27124567, cg12547459, cg01768115, cg27523854, cg07762927, cg11282724, cg03558921, cg27291055, cg17381365, cg16865908, cg26916936, cg02101355, cg03891346, cg24213115, cg07281370, cg24150623, cg06749053, cg20475322, cg24603444, cg01837269, cg07685357, cg03732541, cg11191385, cg25169483, cg00655788, cg22870970, cg11795976, cg19888881, cg12256875, cg20804799, cg24493634, cg00049528, cg03649143, cg06811361, cg18590130, cg19116912, cg17726105, cg24655701, cg25444249, cg24749015, cg02093449, cg01577707, cg14584292, cg15871951, cg17681256, cg23684399, cg19049785, cg11613644, cg10789610, cg17425265, cg20296844, cg23827862, cg04803402, cg17793749, cg16642299, cg15426815, cg23792592, cg12991101, cg22754206, cg16150381, cg27337176, cg00596687, cg11797130, cg02726814, cg07915635, cg17267710, cg15076384, cg08508227, cg19973758, cg04506202, cg07691119, cg17455757, cg08737296, cg02650317, cg02065637, cg04927004, cg15699267, cg20277905, cg19267861, cg03387135, cg01052879, cg103021643, cg012800983, cg060491713, cg060226643, cg202381283, cg157730803, cg011546561, cg243056931, cg050794051, cg219385062, cg133303632, cg24702147, cg20979075, cg00366413, cg27534624, cg18959988, cg23067082, cg19794481, cg18185189, cg16416232, cg05882929, cg08684257, cg02196420, cg01236281, cg13314718, cg19864886, cg02049368, cg21285193, cg14433482, cg05375014, cg08025208, cg12267218, cg02603007, cg17033471, cg06048153, cg03145994, cg13991324, cg19986126, cg01273384, cg16826172, cg04763539, cg23280890, cg14893206, cg24219126, cg23103726, cg15010213, cg111551721, cg133232561, cg001870991, cg225405751, cg174296821, cg203476471, cg050937411, cg041981441, cg212602881, cg211551181, cg212741361, cg079878901, cg219638542, cg097270461, cg119265252, cg195390482, cg084130602, cg040286042, cg046739371, cg167745111, cg153119541, cg242127381, cg080390841, cg065066231, cg054513591, cg110989841, cg096386861, cg133489071, cg069167251, cg147534931, cg144702231, cg216357062, cg259875142, cg242480072, cg198563831, cg265537411, cg101915011, cg072866821, cg003626901, cg174807051, cg116444011, cg274177491cg16226106, |
| MIR548N | 155 | cg21580460, cg09856671, cg27656579, cg17086320, cg07608537, cg20585792, cg26189762, cg07240301, cg00617212, cg13999701, cg15404715, cg15931313, cg23632539, cg13504602, cg18341081, cg21769534, cg02023150, cg21895505, cg10866988, cg23896919, cg06190257, cg01297352, cg16393730, cg27304428, cg03760658, cg01011934, cg16962115, cg03678274, cg00045689, cg04144714, cg13917775, cg07821529, cg25223332, cg16129451, cg22602349, cg00354771, cg17112200, cg15236354, cg13598366, cg00209105, cg12670110, cg04732087, cg07241406, cg01545863, cg14294034, cg04737542, cg22006089, cg21086541, cg17479598, cg24720069, cg02805410, cg00814997, cg16520626, cg12787622, cg23787364, cg27006666, cg21743698, cg02559101, cg13489860, cg25922934, cg14038077, cg13494769, cg12966865, cg16864003, cg03828080, cg18755413, cg15972734, cg06707840, cg27234853, cg04969551, cg06647133, cg22601570, cg01160855, cg17019616, cg17826531, cg12964647, cg26072749, cg26916621, cg07631144, cg15649236, cg01572694, cg14884929, cg07625849, cg13652985, cg10432569, cg02329038, cg23941495, cg21958069, cg26608174, cg04735310, cg01452847, cg21164660, cg01638497, cg16280986, cg13849515, cg06625332, cg177663811, cg040271321, cg172016381, cg269027771, cg051477081, cg205878741, cg196480231, cg038086741, cg119484561, cg270788121, cg208850781, cg00164078, cg06664913, cg13670878, cg00178749, cg02094460, cg15960578, cg18672759, cg02511750, cg05652528, cg16892661, cg05516842, cg26803803, cg10525105, cg02746110, cg02118630, cg02606469, cg16769912, cg09247556, cg21058639, cg09874326, cg00956987, cg24443673, cg13823166, cg15792957, cg10285618, cg01866861, cg09373730, cg20668834, cg13618190, cg14237297, cg07641563, cg25249448, cg24343835, cg12686754, cg20102086, cg05466976, cg016562211, cg162979381, cg061514641, cg074574022, cg128855492, cg011055212, cg232384382, cg000743131, cg250641301, cg057818121, cg199166591, cg177404341 |
| MIR548G | 143 | cg24560920, cg05098551, cg11251378, cg06341370, cg04053862, cg23412136, cg17458653, cg06287708, cg26149223, cg12089249, cg03991736, cg18193109, cg11539052, cg01659489, cg00074313, cg25064130, cg05781812, cg13962724, cg02342910, cg04888852, cg06807315, cg19916659, cg17740434, cg04321497, cg15609237, cg19906284, cg08691235, cg08736813, cg18977423, cg16214269, cg02659030, cg10038720, cg13313833, cg10087519, cg09915519, cg17933893, cg16910097, cg06867904, cg27495572, cg10939966, cg03814399, cg14792798, cg09777256, cg14869618, cg01690287, cg17396522, cg18183294, cg16234718, cg22950670, cg24600426, cg27352348, cg05273635, cg15569739, cg16826256, cg01103718, cg06316114, cg14195166, cg16516429, cg19982471, cg16705245, cg13285968, cg01452873, cg10866755, cg22657780, cg24575676, cg11113760, cg09279240, cg06695611, cg16400999, cg14670435, cg00651020, cg05687686, cg11854219, cg26753290, cg11354643, cg04103432, cg08237722, cg15476410, cg21558423, cg25080630, cg22124629, cg26006165, cg17827949, cg18929085, cg06161762, cg15993931, cg07566172, cg05337739, cg10580067, cg00941833, cg07865161, cg20953052, cg00332776, cg14777783, cg27291385, cg15616915, cg19223411, cg08460635, cg14045814, cg23131909, cg22306928, cg00215432, cg00218620, cg00705280, cg21615583, cg14358282, cg05787409, cg13723257, cg04348419, cg02257674, cg18854412, cg26394220, cg12981188, cg23869335, cg24411913, cg04671367, cg15511049, cg02219451, cg21012050, cg18528621, cg02611257, cg22414362, cg04855975, cg23417140, cg26974217, cg00715363, cg08124860, cg09881857, cg18630030, cg018221241, cg263744811, cg017260381, cg134751551, cg066718681, cg122567471, cg078663711, cg213415581, cg002133341, cg247571591, cg021458661, cg244262901, cg050264371, cg038518611 |
| MIR5095 | 141 | cg19685229, cg12704462, cg18805612, cg21913981, cg00009001, cg19844829, cg18144866, cg08824012, cg17120358, cg24826413, cg20337110, cg14026942, cg23491508, cg26749351, cg00379002, cg08837158, cg18479875, cg10298988, cg04781016, cg03159456, cg17963042, cg27084959, cg02701388, cg20883326, cg07592079, cg18447751, cg14787959, cg24473277, cg18983669, cg26753518, cg25353401, cg21750426, cg15729697, cg19173502, cg13120955, cg19860299, cg14571688, cg14458094, cg14317467, cg15756733, cg21692850, cg00650240, cg01419181, cg04585185, cg00543364, cg27205487, cg24053070, cg19954363, cg17847345, cg25970929, cg00565412, cg24855498, cg00607521, cg19769982, cg07007506, cg17297071, cg23433889, cg12749863, cg14315558, cg03872783, cg07143733, cg20917552, cg04776469, cg14544087, cg13309012, cg10137287, cg08634018, cg17652718, cg17938553, cg10859636, cg110256411, cg020834331, cg229633171, cg097917851, cg107210661, cg051010191, cg078834071, cg175880911, cg134493941, cg072254751, cg248712261, cg257208031, cg006083341, cg190611421, cg169114871, cg008461361, cg204700831, cg181760571, cg125738431, cg180878031, cg092306791, cg130176341, cg076852282, cg136277762, cg049733992, cg129543792, cg146913073, cg120817591, cg168242901, cg247148831, cg234913441, cg199818651, cg098396541, cg172051931, cg058150342, cg123877402, cg215901802, cg109694702, cg079175162, cg078223882, cg113187722, cg178633262, cg169120302, cg097882172, cg250842792, cg056034402, cg027146923, cg196546121, cg038154801, cg014642472, cg203618811, cg027143311, cg172730981, cg215551721, cg115250631, cg169643571, cg210060341, cg095578882, cg097583612, cg028068241, cg255628781, cg248186901, cg250243121, cg085573471, cg155567321, cg051506191, cg064486991, cg062059381, cg048854551, cg156871791, cg191476491 |
| MIR548F3 | 138 | cg19510057, cg18804562, cg21938179, cg03902122, cg07042371, cg05288356, cg02711460, cg15433309, cg14753740, cg25626508, cg05005023, cg07451859, cg09652807, cg12537546, cg11235787, cg06679494, cg02329670, cg21040575, cg24257550, cg11263011, cg04430582, cg20446176, cg21283720, cg27648405, cg16944574, cg02968629, cg14634563, cg12579196, cg06674503, cg27617207, cg01234911, cg01678692, cg26397352, cg18156845, cg26779305, cg06775068, cg26804183, cg16848280, cg25591377, cg00442174, cg20544516, cg24161106, cg11393407, cg09494646, cg13245539, cg07415388, cg20443227, cg03164243, cg04805065, cg09186408, cg19619576, cg00017887, cg24357003, cg05896714, cg19157647, cg04864152, cg20272287, cg07733567, cg24553547, cg24547137, cg02796621, cg02206323, cg26619894, cg09805692, cg19196414, cg16670446, cg12682573, cg09030392, cg17723549, cg05054006, cg01863613, cg26813908, cg27066254, cg21930443, cg03837627, cg226881372, cg099386771, cg080688001, cg109652591, cg156446681, cg250647701, cg178643991, cg053469811, cg045175121, cg224322501, cg251261861, cg028687431, cg103848551, cg120452771, cg039049291, cg068781661, cg149597211, cg121239241, cg133277971, cg044355111, cg160152071, cg245416411, cg143785391, cg070116331, cg013594381, cg120357661, cg220641551, cg255803831, cg258974261, cg105873341, cg106956061, cg113189471, cg008374851, cg215815011, cg242892561, cg121232691, cg157911711, cg215874391, cg190889611, cg144762931, cg096297341, cg117741161, cg005140031, cg189384791, cg137393481, cg155480991, cg180088651, cg159320591, cg255092591, cg182333171, |

| | | |
|---|---|---|
| MIR548F5 | 122 | cg11925789, cg04054359, cg27428462, cg08474649, cg20155849, cg12834462, cg02151068, cg14040871, cg14004457, cg23936341, cg18717689, cg10504741, cg22576265, cg09399225, cg01731518, cg14766769, cg13930811, cg14431734, cg13563462, cg13304325, cg00424588, cg14166365, cg01567328, cg00978117, cg12954102, cg06321925, cg12286351, cg05106358, cg04042162, cg09595054, cg17713013, cg27342371, cg19122747, cg01246632, cg07909384, cg16849649, cg17639867, cg09105685, cg17615262, cg08999352, cg27070458, cg22783100, cg17017819, cg07292215, cg27343747, cg14723090, cg03959541, cg06953499, cg13718827, cg17153540, cg16481138, cg08445157, cg24225917, cg01916700, cg11418490, cg21824858, cg11100685, cg15010497, cg15068681, cg26802390, cg04710273, cg26179116, cg22873268, cg15167810, cg10028175, cg04353365, cg18745642, cg14525247, cg10404717, cg20413392, cg20451272, cg14467654, cg04235540, cg00654637, cg13298616, cg20650753, cg19082696, cg06521718, cg00230342, cg11361720, cg24070801, cg10494844, cg17016635, cg02748477, cg11441832, cg11338041, cg06710212, cg20487632, cg12765908, cg18870599, cg07363202, cg10232005, cg10461164, cg13157805, cg23593672, cg12168488, cg20101164, cg03807917, cg21456301, cg18712351, cg11461263, cg13924954, cg04370182, cg24699132, cg18748580, cg15937784, cg24787924, cg00109776, cg19756622, cg087611671, cg095053371, cg160410902, cg065311582, cg090631732, cg008054732, cg088712241, cg023908581, cg003397262, cg026361622, cg086133502, cg203714373, cg247577522 |
| MIR548AZ | 121 | cg26213561, cg04153571, cg00282704, cg24823751, cg05341330, cg01815730, cg07158816, cg01283398, cg11651443, cg19478638, cg05559808, cg12329853, cg09916797, cg19767857, cg00064589, cg00622375, cg15856028, cg24439831, cg25060796, cg02887712, cg15437223, cg16324767, cg00022938, cg21524784, cg02790122, cg16276613, cg02194384, cg07538027, cg13992202, cg15279236, cg06348245, cg02501544, cg27242911, cg16617349, cg02632490, cg25972714, cg16692757, cg16570507, cg23322812, cg12757181, cg01856509, cg26271970, cg17210014, cg24497344, cg23477281, cg21562001, cg21452633, cg11781069, cg17343879, cg03948048, cg24445034, cg17856907, cg13860000, cg06527815, cg00695064, cg22883879, cg00991520, cg20517640, cg10581650, cg01375259, cg23751416, cg26882429, cg12469964, cg09443153, cg26241514, cg02393496, cg25979474, cg09701700, cg25851152, cg08437570, cg05251190, cg05858126, cg15857661, cg13442016, cg10949319, cg12798274, cg06102342, cg20502234, cg25574153, cg25717590, cg21688999, cg17384806, cg04205369, cg23130731, cg03775802, cg25414746, cg08540829, cg06773798, cg25588914, cg16889524, cg06031160, cg24201716, cg15372479, cg09632185, cg23206103, cg18864164, cg00568128, cg25451306, cg17741865, cg01390168, cg24012440, cg26301661, cg25135143, cg13565543, cg23426816, cg03719236, cg03115097, cg13555855, cg27102904, cg26986226, cg06722483, cg26042267, cg20893537, cg22781400, cg11616411, cg24823137, cg174427231, cg114784951, cg133012491, cg079394971, cg063377001 |
| MIR1273H | 106 | cg04792715, cg00490249, cg25312696, cg14069251, cg26874558, cg20610269, cg21038223, cg14148944, cg21019820, cg23479905, cg07725313, cg24584723, cg17988347, cg12741606, cg06362328, cg07969202, cg22991739, cg14035970, cg03345933, cg17259862, cg07610339, cg23581009, cg17790804, cg08673882, cg09066298, cg19019198, cg25583774, cg20891622, cg00906833, cg02017047, cg05269632, cg18675902, cg01600732, cg03690528, cg23078228, cg09603919, cg02953897, cg04522625, cg25366141, cg13519595, cg21324884, cg19376734, cg00832222, cg05952841, cg03981818, cg05509179, cg10926971, cg05971592, cg06615524, cg27308177, cg26526531, cg01970886, cg23015354, cg20382675, cg18558767, cg15226786, cg21585477, cg05684371, cg10563834, cg18951035, cg26374481, cg01726038, cg13475155, cg06671868, cg12256747, cg07866371, cg21341558, cg00213334, cg24757159, cg02007225, cg15724116, cg25134567, cg04153722, cg20152382, cg09259308, cg07043025, cg08963258, cg04798303, cg12896445, cg11375638, cg19942007, cg03633686, cg09408064, cg01814935, cg16361383, cg22731393, cg04782064, cg186979911, cg108079611, cg108702671, cg142017341, cg069237671, cg247785681, cg153509461, cg009482091, cg123860651, cg183489751, cg094542951, cg038430231, cg211794251, cg058785191, cg019025841, cg107831971, cg205480681, cg270642841, cg035850491 |
| MIR548W | 102 | cg12382480, cg23363242, cg23821020, cg04257196, cg18480071, cg16864411, cg05394484, cg17574593, cg18714170, cg24856024, cg17518643, cg04036282, cg06338239, cg21317441, cg10145411, cg06199909, cg15353530, cg13885053, cg02013168, cg17148213, cg09561830, cg19635644, cg02702515, cg18560638, cg16193278, cg24330404, cg07424038, cg03156020, cg12413643, cg16261025, cg19890584, cg23872155, cg14015828, cg23109066, cg17549097, cg04721126, cg26399947, cg07832271, cg16428374, cg14765087, cg11432250, cg25549968, cg07532986, cg03542468, cg13130979, cg19233792, cg18729186, cg10336220, cg07589927, cg19576080, cg09335599, cg18020268, cg15094224, cg20801666, cg20566583, cg12206978, cg08753951, cg02296376, cg08221669, cg06197043, cg00384653, cg07159686, cg25037841, cg21776091, cg16806458, cg04378107, cg22678932, cg24330297, cg12155013, cg14030055, cg02473781, cg11673244, cg16244770, cg04282607, cg16974014, cg03328201, cg114772731, cg265325271, cg227199981, cg269742171, cg007153631, cg081248601, cg098818571, cg186300301, cg000182161, cg217176011, cg143629521, cg232472811, cg269082571, cg109141431, cg120447031, cg057382351, cg097851441, cg065929081, cg117089121, cg230185481, cg000137021, cg215736961, cg043025671, cg014653501, cg057058921, cg207510481 |
| MIR3134 | 99 | cg18311537, cg15912800, cg26259537, cg05250768, cg01024168, cg05027336, cg08964780, cg25644556, cg24884519, cg08362210, cg15293367, cg18134292, cg11642336, cg10265254, cg12570246, cg22492271, cg11845241, cg00629788, cg15444722, cg21840031, cg24709463, cg12487742, cg20912014, cg21414933, cg16954452, cg16315945, cg04102916, cg05354946, cg26840824, cg07733057, cg01459030, cg18773807, cg12348198, cg16120102, cg26749833, cg03816029, cg21317815, cg04650977, cg22669561, cg04138378, cg09021393, cg18964251, cg19624555, cg26977749, cg09184764, cg09555403, cg01290725, cg24854681, cg22420044, cg09522706, cg22638766, cg00170236, cg08971171, cg06218186, cg17022727, cg03265037, cg02924836, cg09238235, cg00287016, cg09538129, cg09186183, cg00094751, cg10094907, cg18745690, cg14701566, cg04360313, cg26257177, cg13432391, cg25487903, cg10948777, cg04349565, cg17165284, cg15392792, cg07248407, cg00466073, cg27648270, cg18544365, cg23047544, cg02660440, cg06754197, cg02907064, cg23068797, cg088817691, cg077383061, cg127768921, cg144782421, cg129356271, cg112556871, cg210251681, cg078159191, cg247957211, cg066243691, cg136442951, cg042914711, cg168432501, cg037570431, cg201558491, cg128344621, cg021510681 |
| MIR548F1 | 97 | cg09952954, cg09417889, cg09292077, cg24836396, cg12446939, cg16998122, cg07234865, cg20582655, cg26371731, cg15270145, cg13149127, cg10778599, cg06609427, cg09793584, cg22630361, cg22629266, cg06266663, cg12604331, cg08480098, cg05345888, cg26543539, cg25935178, cg21348515, cg10855283, cg00683996, cg25682974, cg22967866, cg06783901, cg22341854, cg26766880, cg16746864, cg17940268, cg24428232, cg06928021, cg24554839, cg17323982, cg10332369, cg23910043, cg12530592, cg06906462, cg11157034, cg04436701, cg05212543, cg12176191, cg16716044, cg20972167, cg20269458, cg25214966, cg01006503, cg19219378, cg24002149, cg21450888, cg09281118, cg17178220, cg03322057, cg09043115, cg16084482, cg12033297, cg09024791, cg20323571, cg09005612, cg10987850, cg07796261, cg11333566, cg01188867, cg21099163, cg02176951, cg11344217, cg22439209, cg04384018, cg13393373, cg26258950, cg13578899, cg04616652, cg11029198, cg16201684, cg03221073, cg16436686, cg09411231, cg06533233, cg25229253, cg23208294, cg19239783, cg03009864, cg09213330, cg18615472, cg25694498, cg02034330, cg24834981, cg12481789, cg065332331, cg252292531, cg232082941, cg192397831, cg192619681, cg066387231, cg084704081 |
| MIR548AN | 71 | cg05733895, cg19775372, cg23815565, cg23642110, cg19061781, cg06518008, cg05483851, cg26580968, cg22545525, cg24346519, cg25333889, cg01850295, cg03678049, cg02903877, cg17879823, cg21829017, cg01381219, cg07364026, cg04370758, cg16013896, cg01242189, cg01662894, cg22912535, cg07140093, cg01784331, cg11065747, cg12970946, cg11851976, cg05581661, cg05072637, cg01309597, cg23590542, cg17388916, cg16756504, cg18360270, cg01206174, cg24188030, cg07396237, cg15821414, cg04772569, cg02408313, cg23847029, cg03635774, cg02566189, cg11210410, cg12306793, cg01194567, cg02273999, cg03196845, cg11446824, cg03269638, cg05233902, cg27495603, cg20528695, cg06701473, cg02740487, cg09526255, cg09227887, cg01894436, cg21507303, cg13304430, cg11626619, cg04271687, cg12328140, cg00501622, |
| MIR5694 | 69 | cg10945180, cg22180675, cg04319844, cg00328284, cg05066959, cg19214487, cg05535168, cg07817409, cg05722052, cg05250467, cg04249036, cg18309927, cg06080856, cg25499471, cg07618155, cg25678108, cg10698928, cg07792478, cg14590098, cg02226645, cg04104463, cg15448064, cg08269119, cg27569742, cg06553422, cg07531072, cg12224165, cg21293810, cg25387811, cg14251798, cg05403831, cg14873313, cg09612163, cg27628515, cg06055845, cg26620021, cg07815521, cg09380135, cg07204707, cg17035787, cg20969585, cg26946818, cg07404579, cg14924324, cg13354838, cg231723331, cg126610001, cg205406721, cg124546191, cg234468571, cg087896451, cg084821451, cg217594401, cg073782391, cg085121881, cg103891081, cg127610591, cg116976531, cg173782651, cg020853691, cg001303071, cg067742072, cg156750542, |
| MIR5009 | 67 | cg04673937, cg27477134, cg18153989, cg23848666, cg16255697, cg10997274, cg00694968, cg10199730, cg06129596, cg20344820, cg06750118, cg06534952, cg21288474, cg20486690, cg16296431, cg25265343, cg08634657, cg07653550, cg05878968, cg20454429, cg25221323, cg08261807, cg13272357, cg08585558, cg20788415, cg24667168, cg19791341, cg22449592, cg12030638, cg02505036, cg24923927, cg08303228, cg10796603, cg20978230, cg101372871, cg086340181, cg176527181, cg179385531, cg108596361, cg141974041, cg234222701, cg189867451, cg082487901, cg127871851, cg129750101, cg152001511, cg142028201, cg191152601, cg004020421, cg158869431, cg115626591, cg254467271, cg036731911, cg117146471, cg182508811, cg054528681, cg037999711, cg178884821, cg025809441, cg201869821, cg037941541, cg251185451, cg125448121, cg028684401, cg050179021, cg154241151, cg082377351 |
| MIR5096 | 64 | cg10804699, cg00909706, cg10640389, cg09994773, cg24501230, cg17776832, cg24540396, cg22487972, cg18833720, cg12002159, cg05427056, cg00061368, cg03785447, cg13393986, cg20786733, cg25214531, cg05709437, cg12723191, cg11873772, cg07472880, cg22351390, cg06638723, cg08470408, cg04875097, cg03237401, cg26481500, cg08969297, cg07435647, cg27238955, cg13758814, cg12343554, cg02140051, cg12496806, cg07895006, cg06774144, cg23994787, cg01196224, cg13935040, cg23989838, cg07370431, cg22821358, cg01092932, cg25349643, cg26160460, cg16010809, cg00111333, cg06105747, cg03426600, cg17969792, cg08866945, cg22898559, cg15265199, cg13770529, cg25346576, cg07855056, cg14888846, cg24102938, cg24675879, cg095687171, cg197702781, cg060673031, cg238969191, cg061902571, cg012973521 |
| MIR181A1HG | 63 | cg14571071, cg16854097, cg05712513, cg07476328, cg07829693, cg25575961, cg26317934, cg01664843, cg01770799, cg27467238, cg00052925, cg08941871, cg26697882, cg06927305, cg22516975, cg19015264, cg17801058, cg02529627, cg03775941, cg19896046, cg12696026, cg03244874, cg13191319, cg05744073, cg12255698, cg19405854, cg25440818, cg14610962, cg16048942, cg21810173, cg000662701, cg047884421, cg245056192, cg272389551, cg137588141, cg123435541, cg021400511, cg124968061, cg078950061, cg067741441, cg239947871, cg011962241, cg139350401, cg239898381, cg073704311, cg088669451, cg228985591, cg152651991, cg033863201, cg243790851, cg119843681, cg093982071, cg188188011, cg238281461, cg144413121, cg048764171, cg056192151, cg252645071, cg252492901, cg261584471, cg215172581, cg097028591, cg183913441 |
| MIR646HG | 63 | cg01076406, cg17674548, cg20598843, cg22690030, cg26533737, cg06399342, cg15401363, cg02823603, cg25047306, cg00118309, cg26740830, cg06845581, cg26366521, cg09372698, cg05806250, cg23510372, cg23061351, cg02216057, cg04476849, cg19628038, cg05599348, cg21562836, cg02921294, cg13933773, cg17192679, cg12116566, cg19434750, cg23628411, cg20063407, cg031448141, cg067062041, cg206151411, cg131742291, cg075676791, cg050452281, cg189882191,cg102956711, cg181005811, cg059751761, cg033449851, cg106732901, cg100523961, cg170707481, cg085085411, cg053245971, cg111772961, cg274197511, cg275858301, cg174314461, cg095751571, cg079358321, cg073607631, cg233263341, cg008360311, cg164162321, cg058829291, cg086842571, cg021964201, cg012362811, cg133147181, cg198648861, cg122672181, cg026030071 |
| MIR7641-2 | 58 | cg00104570, cg01854420, cg06062590, cg05674309, cg22567852, cg15029882, cg03955542, cg15534778, cg08537847, cg25768776, cg27200158, cg09970855, cg06032483, cg00571033, cg01275681, cg14278808, cg15248835, cg04946456, cg10829374, cg04158241, cg14820613, cg20555778, cg26683906, cg06513099, cg05878876, cg10467906, cg14907703, cg13666122, cg24579698, cg00505001, cg26199443, cg09964116, cg26998842, cg17517390, cg25219651, cg05716290, cg17070748, cg08508541, cg08264124, cg08464860, cg06701191, cg19127840, cg13716034, cg01963147, cg083964451, cg164733841, cg228277291, cg221340241, cg122862101, cg048455201, cg255885761, cg194817271, cg096879461, cg088027381, cg080953611, cg014725781, cg045428691, cg059400491 |
| MIR548H3 | 54 | cg13370209, cg27033302, cg21836699, cg27663716, cg08174890, cg25206536, cg04203702, cg06606539, cg25960403, cg00352325, cg19628270, cg16683394, cg21129041, cg06316739, cg00749969, cg10603296, cg07711036, cg22508905, cg14880874, cg04185799, cg13716848, cg07162914, cg25734769, cg27343278, cg12112338, cg02798211, cg08450323, cg17949025, cg18363417, cg08515869, cg18253971, cg24399872, cg18288303, cg01962062, cg09067234, cg06871184, cg12956598, cg19852264, cg05648345, cg02446225, cg19485268, cg20727147, cg091117182, cg018231201, cg274425801, cg195213841, cg009325281, cg023983711, cg157884511, cg119985792, cg144495241, cg160552941, cg025827811, cg031216591 |

| | | |
|---|---|---|
| MIR99AHG | 51 | cg27236331, cg14554415, cg09886641, cg07775813, cg13757194, cg26820811, cg09476148, cg25991730, cg15985873, cg03162973, cg08500698, cg04880611, cg14251844, cg22169791, cg15477165, cg05387996, cg09253111, cg20823973, cg12734206, cg13848961, cg07446205, cg18819275, cg15197583, cg25345561, cg15040157, cg08148660, cg20532999, cg17073392, cg09453870, cg27118929, cg02670903, cg19461383, cg22952917, cg06680906, cg12704993, cg11400650, cg25173269, cg04525074, cg20577165, cg11155172, cg13323256, cg00187099, cg22540575, cg17429682, cg20347647, cg05093741, cg04198144, cg09727046, cg238184011, cg219638541, cg181497942 |
| MIR1256 | 48 | cg12846127, cg12462099, cg08386913, cg07262266, cg18132919, cg05216534, cg13550493, cg16804902, cg01866220, cg19641184, cg16797148, cg26837844, cg23054308, cg16935914, cg18129863, cg10854110, cg27048624, cg04571421, cg22264517, cg02396086, cg06036686, cg03372467, cg02558026, cg09041483, cg00960050, cg07378245, cg08297053, cg06665774, cg10515900, cg21469373, cg24169735, cg13914857, cg06625777, cg01064941, cg07029395, cg19139671, cg10647025, cg03829195, cg05091313, cg19878017, cg15918587, cg230613511, cg022160571, cg044768491, cg196280381, cg055993481, cg215628361, cg083408851 |
| MIR7853 | 47 | cg13327797, cg04435511, cg16015207, cg24541641, cg14378539, cg07011633, cg01359438, cg12035766, cg22064155, cg25580383, cg25897426, cg10587334, cg10695606, cg11318947, cg00837485, cg21581501, cg24289256, cg12123269, cg15791171, cg21587439, cg19088961, cg14476293, cg09629734, cg11774116, cg00514003, cg18938479, cg13739348, cg15548099, cg18008865, cg15932059, cg25509259, cg18233317, cg22725112, cg25469009, cg20485469, cg07494702, cg18217102, cg24115397, cg24909927, cg08710520, cg02229781, cg13391521, cg20576928, cg19827372, cg20331458, cg03332116, cg16400754 |
| MIR1273E | 44 | cg18818801, cg23828146, cg14441312, cg04876417, cg05619215, cg25264507, cg25249290, cg26158447, cg21517258, cg09702859, cg18391344, cg17360849, cg11661940, cg23236366, cg10539122, cg10987004, cg17369088, cg05141870, cg11257193, cg18949641, cg01373258, cg22700328, cg21915970, cg23892547, cg20286236, cg25571269, cg22894896, cg22533683, cg24984384, cg07953150, cg139177751, cg210865411, cg174795981, cg247200691, cg028054101, cg008149971, cg165206261, cg127876221, cg237873641, cg270066661, cg217436981, cg025591011, cg134898601, cg259229341 |
| MIR100HG | 43 | cg16055294, cg02582781, cg04274236, cg20643675, cg17392201, cg24128045, cg26110907, cg06625244, cg01411759, cg22844368, cg10362294, cg22166633, cg27352015, cg15783452, cg08160970, cg17686973, cg24091975, cg11179120, cg23173647, cg14113046, cg10382967, cg03799024, cg119985791, cg272910551, cg173813651, cg037325411, cg111913851, cg251694831, cg006557881, cg228709701, cg122568751, cg208047991, cg244936341, cg000495281, cg254442491, cg247490151, cg020934491, cg015777071, cg145842921, cg158719511, cg176812561, cg236843991, cg190497851 |
| MIR548H2 | 42 | cg13414834, cg01712737, cg11574092, cg13232118, cg17367832, cg06591185, cg24133207, cg02797108, cg08495468, cg10093972, cg20745248, cg24605341, cg23230910, cg05365685, cg15254736, cg07314821, cg12115190, cg06444180, cg12044703, cg05738235, cg09785144, cg06592908, cg11708912, cg23018548, cg00013702, cg13960857, cg21781319, cg26640037, cg21573696, cg04302567, cg247276621, cg102313321, cg030408071, cg138463591, cg093062521, cg186823811, cg271143541, cg140830401, |
| MIR548O2 | 38 | cg09865068, cg10230957, cg19318253, cg23915659, cg06201287, cg11127866, cg20179863, cg01305537, cg11216595, cg16901161, cg17120588, cg08350739, cg03306240, cg03407996, cg27137124, cg25938287, cg12812838, cg05664895, cg27271216, cg26502194, cg09887953, cg24141911, cg00534479, cg17324804, cg24185852, cg25592746, cg02721852, cg19554207, cg25916625, cg10915225, cg15830378, cg13020663, cg052165341, cg135504931, cg166575821, cg134359381, cg086772591, cg226667871 |
| MIR548Q | 37 | cg13727277, cg17581870, cg09174653, cg02022181, cg06242730, cg21308528, cg12338935, cg23184739, cg13063348, cg11807318, cg09196059, cg16011164, cg01942816, cg22108567, cg25343388, cg09286367, cg17419731, cg01024247, cg00966405, cg04155485, cg03040622, cg02355304, cg20511815, cg17149568, cg15802249, cg26792730, cg00240971, cg17777998, cg07184013, cg25771013, cg03853208, cg16291657, cg09596116, cg14508213, cg20041257, cg052052991, cg145887381 |
| MIR548AE2 | 33 | cg27280022, cg11030620, cg23912509, cg07287698, cg12638357, cg13173541, cg24232662, cg10243781, cg26395896, cg01019512, cg17203920, cg11025641, cg02083433, cg22963317, cg09791785, cg10721066, cg05101019, cg07883407, cg17588091, cg13449394, cg07225475, cg24871226, cg25720803, cg00608334, cg19061142, cg16911487, cg00846136, cg20470083, cg18176057, cg12573843, cg18087803, cg09230679, cg13017634 |
| MIR548A2 | 31 | cg25572910, cg03956640, cg15658577, cg21749200, cg16419334, cg14284588, cg22050893, cg03941238, cg16298238, cg08735147, cg24230840, cg06634637, cg24816622, cg05976412, cg23371940, cg14487926, cg23726145, cg23013151, cg07101541, cg27047829, cg07871369, cg230782281, cg096039191, cg045226251, cg253661411, |
| MIR6130 | 31 | cg01979460, cg19582000, cg25760888, cg05835950, cg15974831, cg04675191, cg19698987, cg00637980, cg19016265, cg12965535, cg13237947, cg25732522, cg10271981, cg05370860, cg25212776, cg01522721, cg12004065, cg12220843, cg00876541, cg19324704, cg03611912, cg050993851, cg193442771, cg241785141, cg069326911, |
| MIR656 | 29 | cg22068580, cg08246447, cg13869899, cg14977365, cg25188071, cg21983491, cg03030267, cg228319781, cg022584441, cg270838911, cg093494091, cg040248222, cg227884852, cg257707022, cg127785803, cg026752603, cg130370813, cg271630613, cg206999603, cg207983944, cg008613164, cg027057524, cg105295991, cg045786311, |
| MIR155HG | 28 | cg04028604, cg25250241, cg08340885, cg24965800, cg01314576, cg07277490, cg03700990, cg24489774, cg08096166, cg05574878, cg11163995, cg18784552, cg08981801, cg119265251, cg195390481, cg084130601, cg259709291, cg005654121, cg248554981, cg006075211, cg197699821, cg070075061, cg172970711, cg234338891, cg127498631, |
| MIR548AC | 27 | cg01243312, cg27339255, cg00213281, cg05826944, cg07459181, cg18900544, cg22084004, cg17307471, cg08631141, cg06064461, cg19447671, cg02622866, cg26965059, cg00829688, cg01943478, cg19758054, cg08643007, cg14931631, cg17504336, cg04276626, cg02515217, cg15759721, cg07181702, cg002224551, cg216047421, |
| MIR548T | 27 | cg21604660, cg26487966, cg18654971, cg15201847, cg14079131, cg07707227, cg10928257, cg21905516, cg21240555, cg03638907, cg09942712, cg04251661, cg11833735, cg23035142, cg20857794, cg10576384, cg13237382, cg05367489, cg15411403, cg17588371, cg06616408, cg05246994, cg04877466, cg23551527, cg17170590, cg22581536, |
| MIR124-3 | 26 | cg13214067, cg00190642, cg06885779, cg10871818, cg22952287, cg07779826, cg03090449, cg01404374, cg24602572, cg12273094, cg08145178, cg02746718, cg10984692, cg087372961, cg026503171, cg020656371, cg049270041, cg156992671, cg202779051, cg192678611, cg033871351, cg010528791, cg186273601, cg150285141, cg066605301, |
| MIR548AI | 25 | cg18372847, cg08469305, cg07191657, cg21767615, cg21348752, cg27244585, cg03611732, cg25726414, cg05088677, cg06760536, cg12362662, cg16908824, cg00182416, cg05748492, cg23073467, cg17365367, cg22462000, cg25579656, cg268897641, cg219038901, cg056933951, cg006878051, cg044270871, cg200178561, cg008738781 |
| MIR124-2 | 24 | cg22782812, cg21690947, cg14128911, cg01074840, cg19433767, cg23512763, cg15477257, cg09859911, cg23755509, cg11811349, cg15457725, cg26090256, cg106989281, cg077924781, cg145900981, cg022266451, cg041044631, cg259000852, cg054747262, cg273136422, cg161896711, cg206530752, cg054557202, cg045597791 |
| MIR129-2 | 24 | cg03727337, cg02072464, cg03281512, cg21997998, cg07743843, cg18374625, cg15617950, cg27388703, cg06105296, cg05880105, cg03737367, cg12660698, cg110748145, cg063720155, cg149012055, cg015146685, cg116381815, cg043557915, cg155565025, cg144163714, cg149446474, cg019394773, cg164074712, cg053763742 |
| MIR196B | 24 | cg20009044, cg17446457, cg19312305, cg12907477, cg05249155, cg00481280, cg00287319, cg03217254, cg20442503, cg09703947, cg03413810, cg00969570, cg24774002, cg164470121, cg183115371, cg159128001, cg262595371, cg052507681, cg010241681, cg050273361, cg089647801, cg256445561, cg248845191, cg083622101 |
| MIR377 | 23 | cg12959622, cg24448231, cg04990372, cg18598146, cg16704590, cg01644741, cg05093113, cg21315017, cg09082617, cg09369136, cg06795741, cg097823441, cg118692692, cg204747882, cg036639552, cg051389572, cg117215542, cg225428592, cg149061102, cg181633642, cg228238212, cg111348012, cg100068872 |
| MIR2052HG | 22 | cg24283288, cg27028514, cg06494105, cg23953773, cg09402652, cg22771960, cg18355533, cg09590897, cg18331165, cg14520588, cg06455385, cg066101191, cg266652291, cg175803261, cg169557801, cg077091811, cg130256941, cg081571181, cg014055271, cg062764391, cg218565081, cg060147071 |
| MIR425 | 22 | cg13348640, cg11948456, cg27078812, cg20885078, cg01656221, cg16297938, cg06151464, cg12885549, cg01105521, cg13667965, cg04697953, cg27480241, cg00460704, cg15580874, cg14386946, cg074574021, cg142537641, cg232384381, cg175636221, cg005803541, cg131513611, cg190602271 |
| MIR596 | 22 | cg00749454, cg14885826, cg12688336, cg24695115, cg05421282, cg02710210, cg09896650, cg02591892, cg09739816, cg05562282, cg16673791, cg224613901, cg067420771, cg179371015, cg056506801, cg256739481, cg119981411, cg091775671, cg098991731, cg121425011, cg158219391, cg164819611 |
| MIR1908 | 21 | cg08633665, cg23063070, cg01084257, cg01621145, cg04945860, cg16934485, cg05076730, cg26180383, cg02243522, cg01940297, cg07084941, cg11792826, cg24465482, cg00444740, cg07018107, cg18290852, cg13719966, cg05425602, cg20831094, cg01417339, cg25964744 |
| MIR202 | 21 | cg25900085, cg05474726, cg27313642, cg04015004, cg21109795, cg10658072, cg14858790, cg09559882, cg09545123, cg03096975, cg07804711, cg12536502, cg234005792, cg111641622, cg193482062, cg054685842, cg097631802, cg146741242, cg131694842, cg145243341, cg079447071 |
| MIR217HG | 21 | cg21184495, cg14511007, cg03576592, cg08943214, cg03810621, cg26883949, cg06769231, cg02389634, cg01165909, cg03184776, cg226881371, cg142372971, cg095850041, cg250354741, cg239361061, cg098603811, cg145936501, cg168798571, cg249806861, cg068151271, cg137612421 |
| MIR3163 | 21 | cg03499052, cg02628902, cg11532267, cg05616278, cg08055924, cg00818473, cg10387769, cg25943096, cg03084103, cg02699698, cg16246356, cg10807961, cg10870267, cg14201734, cg06923767, cg24778568, cg15350946, cg00948209, cg12386065, cg08453238, cg02151939 |
| MIR375 | 21 | cg13363808, cg12232996, cg26576274, cg23289226, cg16557125, cg21232937, cg00057966, cg00176888, cg26112797, cg10530767, cg01951274, cg19466818, cg14890660, cg223069281, cg002154321, cg002186201, cg007052801, cg216155831, cg143582821, cg043484191, cg022576741 |
| MIR410 | 20 | cg02705752, cg25770702, cg22831978, cg02258444, cg27083891, cg09349409, cg119855621, cg084607101, cg053220901, cg207983941, cg008613161, cg127785801, cg026752601, cg130370811, cg271630611, cg206999601, cg040248221, cg227884851, cg082244772, cg132821804 |
| MIR496 | 20 | cg07152460, cg13475388, cg13100764, cg24636220, cg05623655, cg11229063, cg09782344, cg15206656, cg20281075, cg03151445, cg10886901, cg01512638, cg02903601, cg039215991, cg143559412, cg028836663, cg196312642, cg261169692, cg151719622, cg211765972 |
| MIR548AJ2 | 20 | cg15252599, cg11242972, cg15095482, cg07374160, cg17864737, cg07398555, cg24791843, cg20848842, cg05351998, cg00169586, cg148808741, cg041857991, cg137168481, cg071629141, cg257347691, cg273432781, cg121123381, cg027982111, cg084503231, cg179490251 |
| MIR636 | 20 | cg05693395, cg00687805, cg04427087, cg20017856, cg00873878, cg17651653, cg25408758, cg21449177, cg22451117, cg27503740, cg11597887, cg05709325, cg12070337, cg10103520, cg08134221, cg13676849, cg08274139, cg10195619, cg19500057, cg06490299 |
| MIR96 | 20 | cg12804227, cg16907400, cg16964413, cg14933412, cg25109528, cg26764880, cg088270751, cg157005201, cg142097641, cg049597161, cg264231851, cg048282671, cg182695951, cg083226971, cg095374481, cg133745281, cg093884141, cg246168281, cg034468761, cg081005351 |
| MIR31HG | 19 | cg13873343, cg06299872, cg04866810, cg12088501, cg05215474, cg08760431, cg09533720, cg10007372, cg22683879, cg195624531, cg091117181, cg077735151, cg214456031, cg165123761, cg239761851, cg209631071, cg079550151, cg015990281, cg006325871 |
| MIR369 | 19 | cg12778580, cg02675260, cg13037081, cg27163061, cg20699960, cg04024822, cg22788485, cg257707021, cg271733222, cg202950713, cg239924493, cg119855623, cg132821803, cg084607103, cg053220903, cg082244773, cg207983943, cg008613163, cg027057523 |
| MIR638 | 19 | cg18384960, cg21876806, cg01132653, cg02135464, cg19603100, cg25069907, cg23492249, cg10969550, cg10036091, cg18484488, cg03556771, cg10082525, cg00175487, cg24768094, cg25562958, cg09243104, cg23310549, cg12820006, cg26015115 |
| MIR760 | 19 | cg21603975, cg06205938, cg04885455, cg15687179, cg19147649, cg02839651, cg19767580, cg06149446, cg26475094, cg02961808, cg004052321, cg178353691, cg029936301, cg198271671, cg033271641, cg049382551, cg251077171, cg179925091, cg163833891 |
| MIR210 | 18 | cg05455720, cg16543391, cg01544351, cg25277950, cg18515250, cg00029256, cg27559408, cg02869289, cg13602484, cg206530751, cg038808411, cg154825001, cg271936912, cg012773691, cg074108112, cg058580422, cg082002932, cg024717602 |
| MIR663 | 18 | cg06308344, cg15648245, cg27313362, cg21030907, cg22414759, cg15002839, cg00965199, cg00868980, cg10794473, cg182919411, cg271389511, cg060079661, cg015219871, cg083041901, cg143450121, cg041504951, cg203959671, cg107150921 |
| MIR132 | 17 | cg01397463, cg12329484, cg19115260, cg00402042, cg15886943, cg11562659, cg022014911, cg061182061, cg121405301, cg160410901, cg065311581, cg090631731, cg008054731, cg057440731, cg122556981, cg194058541, cg254408181 |
| MIR137HG | 17 | cg19836088, cg07737292, cg22328598, cg16902958, cg00054301, cg19966955, cg06912425, cg056242341, cg011182952, cg254341162, cg156411841, cg220908601, cg032266241, cg232435521, cg263905421, cg207136621, cg097040561 |
| MIR191 | 17 | cg07457402, cg14253764, cg23238438, cg17563622, cg00580354, cg13151361, cg19060227, cg04432606, cg11770407, cg128855491, cg011055211, cg136679651, cg046979531, cg274802411, cg004607041, cg155808741, cg143869461 |
| MIR192 | 17 | cg26373812, cg00611614, cg09235308, cg04018533, cg02140559, cg24137216, cg19782652, cg18906608, cg21954156, cg25273903, cg164349401, cg191194641, cg095531601, cg022584442, cg270838912, cg093494092, cg182628301 |
| MIR373 | 17 | cg07809144, cg05453618, cg205471311, cg232709241, cg142851421, cg182642981, cg028876231, cg127822011, cg071263992, cg030500962, cg109480702, cg212300212, cg046721822, cg021807372, cg079184532, cg154719811, cg230725711 |

| | | |
|---|---|---|
| MIR485 | 17 | cg12517394, cg09677638, cg15598662, cg00786201, cg226624821, cg038056841, cg254012841, cg147256411, cg037350131, cg253268961, cg248707741, cg169165222, cg187992662, cg117301003, cg031142443, cg075836513, cg088733533 |
| MIR675 | 17 | cg26665229, cg17580326, cg16955780, cg07709181, cg13025694, cg08157118, cg01405527, cg06276439, cg07035704, cg26754262, cg21856508, cg06014707, cg11288819, cg12286210, cg04845520, cg19119970, cg22413205 |
| MIR7515HG | 17 | cg02999711, cg20254084, cg25202407, cg01376318, cg26083576, cg24361571, cg06237697, cg21869027, cg15005345, cg09903166, cg067078401, cg272348531, cg049695511, cg066471331, cg226015701, cg001640781, cg066649131 |
| MIR10A | 16 | cg19296149, cg16519758, cg15607706, cg09914785, cg27449804, cg23095729, cg00390484, cg00504285, cg260727491, cg269166211, cg076311441, cg156492361, cg015726941, cg148849291, cg076258491, cg136529851 |
| MIR10B | 16 | cg05445932, cg26799245, cg00242242, cg02491119, cg20461262, cg18564052, cg13712950, cg02776913, cg20650194, cg06673490, cg06598332, cg09111258, cg02742085, |
| MIR142 | 16 | cg00615172, cg15454969, cg22808478, cg08706141, cg07797030, cg22786486, cg11242552, cg06670785, cg150351431, cg212329371, cg000579661, cg001768881, cg261127971, cg105307671, cg019512741, cg194668181 |
| MIR17HG | 16 | cg11050908, cg21792562, cg24529616, cg20562143, cg02027349, cg02067741, cg14953687, cg23741702, cg09661924, cg05965535, cg05594766, cg073812311, cg081933761, cg267537811, cg267894121, cg249420391 |
| MIR1915 | 16 | cg17371911, cg02167149, cg20332679, cg13457217, cg04194933, cg02310165, cg18339098, cg01706263, cg14201324, cg04963089, cg25010142, cg19004110, cg03469437, |
| MIR196A2 | 16 | cg16266268, cg12690224, cg16790055, cg22537343, cg26325497, cg21592803, cg09514545, cg00054741, cg106623141, cg054121371, cg152156901, cg196074291, cg080531371, cg187719371, cg146792551, cg164004951 |
| MIR203 | 16 | cg11828048, cg05045228, cg18988219, cg10295671, cg18100581, cg05975176, cg10673290, cg10052396, cg273143361, cg136738331, cg239603241, cg034019971, cg100595361, cg015208671, cg062187262, cg210911281 |
| MIR206 | 16 | cg18822271, cg11678964, cg02942546, cg05747251, cg18322658, cg10248218, cg09895876, cg12273502, cg147016821, cg038998241, cg136931431, cg228876631, cg156716881, cg227201391, cg269625331, cg252842131 |
| MIR2110 | 16 | cg14739038, cg11334241, cg18560789, cg09661621, cg15083435, cg17528453, cg06162386, cg26413319, cg18699598, cg04460239, cg25239439, cg12424249, cg16008467, |
| MIR412 | 16 | cg20295071, cg23992449, cg27173322, cg11985562, cg08460710, cg05322090, cg08224477, cg132821801, cg207983942, cg008613162, cg027057522, cg127785802, cg026752602, cg130370812, cg271630612, cg206999602 |
| MIR423 | 16 | cg08715009, cg05361272, cg00363066, cg24331563, cg14791048, cg20988440, cg18526167, cg21683783, cg04565201, cg17843124, cg13767001, cg22750273, cg06529264, |
| MIR449C | 16 | cg15055095, cg22481643, cg26234786, cg06758801, cg03656137, cg07600990, cg09573197, cg23221889, cg00015159, cg13612275, cg08350549, cg00210994, cg15963552, |
| MIR494 | 16 | cg19734087, cg09045249, cg16160867, cg13115617, cg10544093, cg00405774, cg08563487, cg14542207, cg011311003, cg074296293, cg104001743, cg141480883, cg012445143, cg158621283, cg173418442, cg128233292 |
| MIR495 | 16 | cg23650853, cg12218895, cg12290369, cg05663594, cg18291941, cg27138951, cg06007966, cg01521987, cg103008641, cg008569521, cg269313071, cg048266521, cg026625761, cg218099131, cg270742211, cg149102271 |
| MIR543 | 16 | cg00350371, cg18146506, cg19137662, cg24825299, cg25085038, cg12617066, cg01983504, cg19127724, cg042721332, cg012324792, cg188756312, cg058655482, cg122421742, cg188050311, cg102702381, cg211464281 |
| MIR5684 | 16 | cg01055610, cg19125323, cg22450146, cg12503190, cg13324603, cg21028463, cg01574134, cg05482832, cg039763641, cg039492741, cg192774111, cg123186021, cg216872371, cg142948921, cg035290401, cg085706311 |
| MIR574 | 16 | cg02629170, cg04257209, cg07109801, cg27634187, cg04878973, cg06365567, cg25691553, cg01142001, cg17217654, cg15463769, cg17092349, cg19526908, cg19767734, |
| MIR611 | 16 | cg18588204, cg12834926, cg23283234, cg03457528, cg08334153, cg13784855, cg24032265, cg25247520, cg11201447, cg13698548, cg22384423, cg27190014, cg03611307, |
| MIR639 | 16 | cg00856952, cg26931307, cg04826652, cg02662576, cg21809913, cg27074221, cg14910227, cg02047319, cg01200264, cg10268144, cg20084852, cg08095475, cg14037354, |
| MIR886 | 16 | cg18609037, cg19835688, cg02317955, cg26766064, cg18637486, cg06807993, cg06869212, cg23295826, cg044819231, cg186786451, cg065366141, cg253406881, |
| MIR9-3 | 16 | cg215293231, cg125305031, cg030825801, cg138886001 |
| MIR933 | 16 | cg22318562, cg25850845, cg21440584, cg26032238, cg03318314, cg15987431, cg01534125, cg01569295, cg20821842, cg17441181, cg08473764, cg26614229, cg02464665, |
| MIR141 | 15 | cg02558684, cg10386111, cg09902908, cg21799523, cg11835050, cg01864361, cg15930678, cg09796029, cg09318158, cg208159111, cg230670821, cg197944811, |
| MIR24-2 | 15 | cg14148088, cg01244514, cg15862128, cg05046068, cg01508548, cg24233562, cg120448621, cg117800421, cg069614291, cg011311001, cg104001741, cg074296292, |
| MIR299 | 15 | cg22424892, cg05448231, cg07342901, cg11753499, cg13210239, cg17985533, cg15922305, cg09701145, cg25437674, cg06029905, cg02808220, cg24183187, cg22932993, |
| MIR329-1 | 15 | cg09196809, cg13559712, cg061499711, cg178771101, cg260393051, cg027917531, cg075523221, cg228245522, cg256191912, cg232252552, cg267336452, cg213622332, |
| MIR655 | 15 | cg01472578, cg04542869, cg05090896, cg17724336, cg06075250, cg25775708, cg161507982, cg020053362, cg227047752, cg110059981, cg006479171, cg229710281, |
| MIR668 | 15 | cg22662482, cg180097981, cg117301001, cg031142441, cg169165221, cg088733531, cg187992661, cg075836512, cg038056842, cg254012842, cg147256412, cg037350132, |
| MIR758 | 15 | cg26253759, cg15489575, cg22824552, cg25619191, cg23225255, cg26733645, cg06149971, cg21362233, cg16732457, cg12517288, cg19536085, cg091968092, |
| MIR762HG | 15 | cg09876651, cg00256294, cg15090644, cg03444423, cg03407651, cg17967602, cg27533635, cg00030523, cg19702383, cg25492056, cg04663692, cg15028128, cg105497531, |
| MIR92B | 15 | cg10489614, cg23207534, cg26390542, cg03417340, cg00699693, cg07281938, cg03848856, cg01735503, cg03549146, cg275380261, cg154343371, cg126780061, |
| MIR1247 | 14 | cg18262830, cg24803202, cg08432452, cg00589493, cg13092487, cg24154336, cg00400165, cg00376448, cg02494703, cg06104877, cg23266943, cg18715874, cg21463067, |
| MIR1258 | 14 | cg02071553, cg12668144, cg09585004, cg25035474, cg23936106, cg09860381, cg14593650, cg16879857, cg24980686, cg06815127, cg13761242, cg03672322, cg27438307, |
| MIR127 | 14 | cg05468584, cg09763180, cg14674124, cg13182610, cg15686115, cg16892611, cg14524334, cg07944707, cg01825707, cg16593552, cg03880841, cg15482500, cg131694841, |
| MIR1292 | 14 | cg06889454, cg16796899, cg03721993, cg00328051, cg22325330, cg10590512, cg01693026, cg18301891, cg15238382, cg02287710, cg22688428, cg01664864, cg02840823, |
| MIR138-1 | 14 | cg14733743, cg04115908, cg16094326, cg05099035, cg01593570, cg20194910, cg13095998, cg107089721, cg079970661, cg062092981, cg033226331, cg124282991, |
| MIR146B | 14 | cg15649193, cg14898306, cg02849766, cg12677723, cg14892570, cg21216828, cg20702935, cg097017001, cg258511521, cg084375701, cg052511901, cg058581261, |
| MIR1471 | 14 | cg26111107, cg19343352, cg01964121, cg10325038, cg10822495, cg02656609, cg02611741, cg180940761, cg010588501, cg120357821, cg258831491, cg060465801, |
| MIR148A | 14 | cg18394275, cg21736038, cg13361168, cg06655225, cg17675546, cg09885380, cg08879181, cg071840131, cg257710131, cg038532081, cg162916571, cg095961161, |
| MIR182 | 14 | cg26480358, cg18654464, cg14066773, cg24866700, cg01627405, cg03694555, cg20930863, cg244237821, cg176770321, cg045796081, cg137130661, cg087559721, |
| MIR187 | 14 | cg02336095, cg21289124, cg04479757, cg22504528, cg25635000, cg00502618, cg26210379, cg058272331, cg189819791, cg187206171, cg243693101, cg035991971, |
| MIR193A | 14 | cg05017902, cg15424115, cg08237735, cg00224335, cg09978395, cg01027226, cg12430252, cg227003281, cg219159701, cg238925471, cg202862361, cg255712691, |
| MIR193B | 14 | cg11702026, cg07535042, cg15999949, cg11239407, cg23539114, cg07413966, cg07259358, cg001386411, cg099186571, cg104203101, cg040183252, cg062730752, |
| MIR196A1 | 14 | cg07021468, cg16472542, cg13811469, cg21285564, cg20689730, cg01630479, cg03147761, cg104325691, cg023290381, cg239414951, cg219580691, cg266081741, |
| MIR25 | 14 | cg18948646, cg09031823, cg08030987, cg09363604, cg03650550, cg19057326, cg16326188, cg09442106, cg10066125, cg02147683, cg10021238, cg02875879, cg06540697, |
| MIR29C | 14 | cg04312413, cg03978498, cg22208012, cg05945782, cg00572843, cg03592438, cg27639133, cg103878071, cg221598151, cg102342821, cg207019011, cg088552491, |
| MIR329-2 | 14 | cg17877110, cg26039305, cg02791753, cg07552322, cg26011437, cg26132853, cg213622331, cg125172881, cg195360851, cg091968091, cg135597121, cg167324572, |
| MIR365-2 | 14 | cg18862975, cg22414262, cg00871610, cg05791108, cg17360196, cg16299399, cg07495738, cg249843841, cg079531501, cg013763181, cg260835761, cg243615711, |
| MIR372 | 14 | cg11134801, cg10006887, cg07126399, cg03050096, cg10948070, cg21230021, cg04672182, cg02180737, cg07918453, cg20547131, cg23270924, cg14285142, cg18264298, |
| MIR376B | 14 | cg04355791, cg15556502, cg14416371, cg01939477, cg142940961, cg076160941, cg044586451, cg185146441, cg110748141, cg063720151, cg149012051, cg015146681, |
| MIR380 | 14 | cg21728792, cg14890224, cg12158055, cg14988425, cg159223051, cg097011451, cg254376741, cg060299051, cg028082201, cg241831871, cg229329931, cg016682791, |
| MIR453 | 14 | cg03805684, cg25401284, cg14725641, cg03735013, cg25326896, cg24870774, cg08618909, cg19504335, cg125173941, cg096776381, cg155986621, cg007862011, |
| MIR487A | 14 | cg11834907, cg25513321, cg11231913, cg23593986, cg24438334, cg19832184, cg01597727, cg164387221, cg015860721, cg030191121, cg003956571, cg233995771, |
| MIR492 | 14 | cg11251554, cg15993786, cg06445981, cg09404289, cg14119827, cg18877961, cg19537184, cg105497532, cg112121782, cg089223082, cg034076511, cg158627061, |
| MIR493 | 14 | cg08239565, cg01975502, cg18403970, cg09484214, cg10200169, cg15471981, cg16388088, cg025777451, cg109402101, cg047144971, cg127855731, cg113469011, |
| MIR539 | 14 | cg23371208, cg20176573, cg206107401, cg016818811, cg001358881, cg050825271, cg165200381, cg176806411, cg050460261, cg256282571, cg260610011, cg125808202, |
| MIR654 | 14 | cg26024682, cg14294096, cg07616094, cg04458645, cg18514644, cg11074814, cg06372015, cg14901205, cg01514668, cg11638181, cg14944647, cg144163711, |
| MIR7-2 | 14 | cg22987078, cg08148458, cg15580304, cg14523475, cg01966791, cg11392297, cg07135405, cg020547241, cg188244461, cg091609551, cg231763401, cg019259651, |
| MIR1306 | 13 | cg06916725, cg14753493, cg14470223, cg21635706, cg25987514, cg24248007, cg19856383, cg26553741, cg10191501, cg07286682, cg00362690, cg17480705, cg11644401 |
| MIR136 | 13 | cg02471760, cg14063402, cg26663525, cg15269875, cg14937069, cg19943238, cg03175030, cg18511798, cg148589911, cg199165451, cg074108111, cg058580421, |
| MIR1909 | 13 | cg03859739, cg15746029, cg08332815, cg26535023, cg17224504, cg17112491, cg25966852, cg13301249, cg07939497, cg06337700, cg06239513, cg14016128, cg17775912 |
| MIR21 | 13 | cg20405620, cg06964030, cg14301580, cg07181341, cg11632273, cg21639001, cg05432102, cg064714911, cg270235971, cg042766261, cg025152171, cg157597211, |
| MIR212 | 13 | cg02201491, cg06118206, cg12140530, cg16041090, cg06531158, cg09063173, cg00805473, cg25446727, cg03673191, cg013974631, cg123294841, cg059457821, |
| MIR345 | 13 | cg18607338, cg13596132, cg13207036, cg12382424, cg21623395, cg13151449, cg03062642, cg20246901, cg21759440, cg07378239, cg08512188, cg10389108, cg12761059 |
| MIR432 | 13 | cg27193691, cg01277369, cg14858991, cg19916545, cg07410811, cg05858042, cg08200293, cg024717601, cg140634021, cg266635251, cg152698751, cg149370691, |
| MIR548D2 | 13 | cg20407154, cg08669738, cg15280864, cg10637930, cg19716542, cg05876692, cg21108691, cg05909164, cg02813845, cg24233224, cg13320585, cg20481419, cg24437010 |
| MIR769 | 13 | cg06746453, cg07521789, cg10734581, cg24845375, cg24578493, cg11245928, cg07059402, cg00565075, cg185864401, cg160399721, cg017375921, cg212105371, |
| MIR93 | 13 | cg080309871, cg093636041, cg036505501, cg190573261, cg147191291, cg040868711, cg163261882, cg094421062, cg100661252, cg021476832, cg100212382, cg028758792, |
| MIR1182 | 12 | cg08970825, cg13899576, cg07897389, cg05193015, cg03434095, cg08582384, cg07840186, cg12917056, cg05535321, cg15809837, cg18670723, cg14745151 |
| MIR1204 | 12 | cg06165213, cg18051316, cg09583599, cg08810053, cg01869058, cg10223234, cg19646028, cg01400401, cg04640886, cg240322651, cg136985481, cg223844231 |
| MIR1257 | 12 | cg26553525, cg12647861, cg14353569, cg01821906, cg06159667, cg11188837, cg170334711, cg060481531, cg031459941, cg139913241, cg199861261, cg012733841 |
| MIR125B1 | 12 | cg02294239, cg06398756, cg06112642, cg02121060, cg11384475, cg03308555, cg05206385, cg00932528, cg02398371, cg15788451, cg11998579, cg14449524 |
| MIR1265 | 12 | cg02149160, cg18169289, cg06905901, cg11581046, cg09782300, cg04329845, cg139599431, cg227451431, cg183728471, cg084693051, cg071916571, cg217676151 |
| MIR1275 | 12 | cg12714522, cg07238273, cg11321186, cg21974464, cg23216916, cg08693080, cg180073411, cg039033981, cg019564721, cg042595651, cg129746681, cg253605351 |
| MIR129-1 | 12 | cg02467518, cg01386868, cg02368428, cg04207179, cg14763933, cg23837289, cg048408001, cg240441862, cg093444852, cg113642902, cg045240881, cg207349821 |
| MIR130A | 12 | cg23160488, cg27367878, cg03010301, cg05493001, cg11794213, cg00633015, cg016818812, cg001358882, cg050825272, cg233712083, cg165200382, cg105120893 |
| MIR130B | 12 | cg22192614, cg20948366, cg05185738, cg17972789, cg13912196, cg02852421, cg22943827, cg07911140, cg134603191, cg161686831, cg169740141, cg033282011 |
| MIR150 | 12 | cg04076760, cg05211746, cg14420798, cg08859349, cg01481205, cg09916443, cg183746251, cg156179501, cg273887031, cg061052961, cg058801051, cg037373671 |
| MIR1826 | 12 | cg18581972, cg16063315, cg03762502, cg10097871, cg07955854, cg15230342, cg006774071, cg059923471, cg066179611, cg110320381, cg265632481, cg085213321 |
| MIR2114 | 12 | cg03050491, cg19572242, cg27616996, cg16746961, cg07910434, cg12159336, cg233181691, cg150339731, cg218707601, cg235700341, cg236989761, cg083164881 |
| MIR216A | 12 | cg16007628, cg08209724, cg20017464, cg21843277, cg21680502, cg19862334, cg076415631, cg252494481, cg243438351, cg126867541, cg201020861, cg054669761 |
| MIR219-1 | 12 | cg18834352, cg09559242, cg19975560, cg04548705, cg10303145, cg18304251, cg25291692, cg23952550, cg15394672, cg08782169, cg077253891, cg019104191 |
| MIR30A | 12 | cg18554492, cg06865629, cg11475886, cg17106157, cg15236080, cg22204047, cg190984371, cg208157781, cg260535711, cg014348921, cg213031691, cg165069101 |
| MIR330 | 12 | cg08509270, cg09337069, cg03290602, cg11005998, cg21124040, cg21346855, cg00707427, cg00647917, cg22971028, cg03862290, cg04223832, cg11116300 |
| MIR34C | 12 | cg08471950, cg11347475, cg00122951, cg26054672, cg03475101, cg05099385, cg19344277, cg088822641, cg235322701, cg062273691, cg090083531, cg045223391 |
| MIR365-1 | 12 | cg16240189, cg19981515, cg04864577, cg13953717, cg05713399, cg17509180, cg096070472, cg069160011, cg009285961, cg025333391, cg223882601, cg028862632 |
| MIR422A | 12 | cg17431446, cg09575157, cg07935832, cg07360763, cg23326334, cg00836031, cg025874121, cg197001501, cg015421151, cg121316011, cg081165501, cg011307771 |
| MIR429 | 12 | cg13440132, cg04862658, cg23820714, cg27309907, cg023316731, cg112039901, cg133625461, cg218107931, cg263304791, cg069671051, cg236518121, cg035283021 |
| MIR487B | 12 | cg13202936, cg13098091, cg12580820, cg06114465, cg20610740, cg01681881, cg00135888, cg05082527, cg16520038, cg038320781, cg233712081, cg105120891 |
| MIR497HG | 12 | cg02390858, cg20371437, cg20186982, cg03794154, cg25118545, cg12544812, cg02868440, cg204461761, cg212837201, cg276484051, cg169445741, cg029686291 |
| MIR503 | 12 | cg00816290, cg07828800, cg05147158, cg16862518, cg00071872, cg18763079, cg14691307, cg176851311, cg076852281, cg136277761, cg049733991, cg129543791 |
| MIR548AP | 12 | cg18642361, cg00944580, cg06339716, cg16682693, cg18222371, cg25665165, cg16820411, cg22731975, cg14871564, cg02629942, cg27455537, cg25945989 |
| MIR548I4 | 12 | cg26171815, cg23019039, cg26019654, cg01578939, cg13650580, cg26894722, cg19296299, cg00978822, cg01988325, cg25728115, cg113678531, cg031359821 |
| MIR607 | 12 | cg14339287, cg10649714, cg17162271, cg22469141, cg13237829, cg23470272, cg138600001, cg065278151, cg006950641, cg228838791, cg009915201, cg205176401 |
| MIR658 | 12 | cg00722320, cg04947764, cg01397141, cg13096580, cg14239640, cg06612459, cg08976738, cg16530970, cg22929280, cg03589751, cg05208249, cg05721790 |
| MIR665 | 12 | cg06774207, cg23219905, cg22532652, cg23072571, cg09422614, cg07785717, cg18055623, cg156750541, cg270398211, cg188046801, cg118871241, cg199459371 |

| | | |
|---|---|---|
| MIR670 | 12 | cg00283662, cg00433887, cg02624019, cg08426951, cg18586440, cg16039972, cg260393052, cg027917532, cg075523222, cg260246823, cg142940964, cg076160944 |
| MIR802 | 12 | cg07194250, cg22955387, cg07776419, cg23245720, cg18412777, cg01807688, cg099783951, cg010272261, cg124302521, cg188629751, cg224142621, cg008716101 |
| MIRLET7I | 12 | cg25844969, cg03706840, cg08446512, cg13595195, cg04885140, cg10016185, cg05821976, cg22500349, cg13234893, cg13576904, cg00020229, cg12479035 |
| MIR376A1 | 12 | cg155565021, cg044586452, cg185146442, cg110748142, cg063720152, cg149012052, cg149446472, cg019394772, cg144163713, cg015146684, cg116381814, cg043557914 |
| MIR376A2 | 12 | cg043557911, cg260246822, cg015146682, cg116381812, cg142940963, cg076160943, cg044586453, cg185146443, cg110748143, cg063720153, cg149012053, cg155565024 |
| MIR1181 | 11 | cg26254637, cg18947995, cg02344891, cg02524808, cg04699566, cg09873328, cg01339630, cg13760742, cg23388037, cg14072069, cg14866339 |
| MIR1224 | 11 | cg00841784, cg24956366, cg25459931, cg12309867, cg14485651, cg00222455, cg21604742, cg00400494, cg05288397, cg15168942, cg13423180 |
| MIR1234 | 11 | cg03189678, cg05893251, cg09705232, cg13957432, cg15778867, cg15968925, cg19535507, cg23689404, cg12172080, cg26759943, cg13330760 |
| MIR1237 | 11 | cg18004197, cg16627915, cg08936078, cg17082719, cg02850815, cg13509702, cg15650298, cg19670588, cg00128812, cg10451724, cg24120597 |
| MIR137 | 11 | cg05624234, cg16421621, cg03125605, cg04650403, cg07173484, cg011182951, cg254341161, cg042937331, cg223332141, cg054235291, cg104896141 |
| MIR1469 | 11 | cg11122256, cg11313099, cg07617764, cg01190168, cg15851964, cg17169243, cg27606822, cg09263904, cg02115865, cg23911696, cg01149144 |
| MIR1470 | 11 | cg12881150, cg14144314, cg17869516, cg04585937, cg21882238, cg05348084, cg02174748, cg10137253, cg14427563, cg06392565, cg10079327 |
| MIR154 | 11 | cg03921599, cg17721331, cg05969522, cg071524601, cg134753881, cg131007641, cg246362201, cg056236551, cg112290631, cg230291592, cg194772052 |
| MIR181A2HG | 11 | cg18547866, cg09946681, cg14344583, cg13673375, cg13075119, cg08111064, cg18053783, cg10506249, cg14158769, cg118573101, cg203329071 |
| MIR184 | 11 | cg21663427, cg26075664, cg15331656, cg07850515, cg00466209, cg04354459, cg012079161, cg231217851, cg007223201, cg049477641, cg013971411 |
| MIR194-2 | 11 | cg16434940, cg19119464, cg09553160, cg08642528, cg263738121, cg006116141, cg092353081, cg040185331, cg021405591, cg241372161, cg232669431 |
| MIR2277 | 11 | cg05396688, cg24828422, cg04371579, cg17983017, cg11268077, cg05759046, cg04606556, cg07552538, cg17241937, cg04114269, cg13219301 |
| MIR300 | 11 | cg08304190, cg14345012, cg149446471, cg019394771, cg155565022, cg144163712, cg015146683, cg116381813, cg043557913, cg021747481, cg101372531 |
| MIR33B | 11 | cg12942606, cg21092373, cg12206359, cg08766508, cg10623685, cg13669476, cg04415610, cg17674402, cg16725262, cg19353642, cg194092541 |
| MIR409 | 11 | cg13282180, cg20798394, cg00861316, cg271733221, cg082244771, cg027057521, cg202950712, cg239924492, cg119855622, cg084607102, cg053220902 |
| MIR411 | 11 | cg21167159, cg16153294, cg15317267, cg06354774, cg224248921, cg054482311, cg073429011, cg117534991, cg132102391, cg179855331, cg026917451 |
| MIR431 | 11 | cg00287908, cg20361828, cg11476463, cg09683457, cg23400579, cg11164162, cg19348206, cg03593550, cg01666793, cg262555601, cg019711301 |
| MIR433 | 11 | cg13169484, cg002879081, cg203618281, cg114764631, cg096834571, cg234005791, cg111641621, cg193482061, cg054685841, cg097631801, cg146741241 |
| MIR4435- | 11 | cg04517512, cg22432250, cg25126186, cg02868743, cg10384855, cg12045277, cg03904929, cg21384971, cg24389730, cg207771861, cg153273951 |
| MIR4500HG | 11 | cg01574903, cg14709103, cg26757562, cg25349981, cg05382137, cg104814701, cg117258351, cg222968741, cg155587271, cg114401481, cg016089621 |
| MIR568 | 11 | cg06324993, cg21717601, cg14362952, cg23247281, cg26908257, cg10914143, cg048857751, cg087239131, cg163589241, cg027718861, cg171648271 |
| MIR589 | 11 | cg09538777, cg05670935, cg03843023, cg21179425, cg05878519, cg01902584, cg10783197, cg20548068, cg15071233, cg09503809, cg06321636 |
| MIR612 | 11 | cg09045465, cg15604056, cg13131168, cg16383318, cg18131559, cg07472764, cg10360420, cg042389831, cg053635341, cg211441581, cg131673401 |
| MIR657 | 11 | cg23254057, cg00356134, cg27116069, cg11701550, cg05365942, cg10921624, cg10460086, cg14133042, cg02523350, cg05580240, cg241181551 |
| MIR661 | 11 | cg13061103, cg14701682, cg03899824, cg13693143, cg22887663, cg15671688, cg22720139, cg26962533, cg25284213, cg06903579, cg14487577 |
| MIR6717 | 11 | cg07459895, cg18576861, cg13224090, cg06292259, cg10281361, cg14626525, cg00265277, cg21512799, cg11198094, cg06282561, cg12326440 |
| MIR7-3 | 11 | cg08244382, cg13662173, cg08175935, cg02135795, cg24721750, cg13555689, cg26976046, cg05899507, cg21103400, cg21217911, cg10726559 |
| MIRLET7B | 11 | cg23763043, cg09500815, cg05127369, cg15070718, cg15533524, cg03433313, cg043303711, cg230427061, cg022784991, cg186147341, cg015012081 |
| MIR1-1 | 10 | cg01362581, cg03297845, cg11440679, cg22117079, cg09392381, cg10545768, cg11560666, cg02219476, cg03944501, cg173656181 |
| MIR101-1 | 10 | cg08557347, cg15556732, cg05150619, cg06448699, cg197842441, cg137497771, cg166826931, cg182223711, cg168204111, cg148715641 |
| MIR106B | 10 | cg04086871, cg05868449, cg163261881, cg094421061, cg100661251, cg021476831, cg100212381, cg028758791, cg065406971, cg147191292 |
| MIR1180 | 10 | cg01719718, cg03565833, cg01929699, cg16621560, cg25874148, cg00269670, cg01409434, cg14247325, cg21504345, cg05757376 |
| MIR1185-1 | 10 | cg05376374, cg07388837, cg12420437, cg04150495, cg20395967, cg164074711, cg144275631, cg063925651, cg100793271, cg052162111 |
| MIR1228 | 10 | cg19444866, cg25355826, cg21281732, cg03016486, cg13781167, cg17201343, cg14048837, cg10068516, cg16246882, cg14057303 |
| MIR1246 | 10 | cg14949813, cg04458627, cg18075185, cg01521220, cg11210138, cg010939341, cg020494721, cg208415881, cg132362711, cg165884171 |
| MIR1249 | 10 | cg02278768, cg01882870, cg25950235, cg21529323, cg12530503, cg03082580, cg13888600, cg03922814, cg14266604, cg07276140 |
| MIR1253 | 10 | cg11714647, cg18250881, cg05452868, cg03799971, cg17888482, cg276391331, cg143115971, cg071698731, cg186149841, cg096528071 |
| MIR1276 | 10 | cg19607429, cg08053137, cg18771937, cg14679255, cg16400495, cg10561239, cg09137135, cg13827287, cg00240007, cg16784745 |
| MIR1303 | 10 | cg23176214, cg26068527, cg24085713, cg13466409, cg11138142, cg239156591, cg062012871, cg111278661, cg201798631, cg013055371 |
| MIR1323 | 10 | cg00500400, cg04452260, cg09331127, cg02046995, cg24349919, cg206436751, cg173922011, cg241280451, cg261109071, cg066252441 |
| MIR135A2 | 10 | cg22725901, cg21744136, cg15616006, cg16466613, cg23908943, cg150281281, cg272800221, cg110306201, cg239125091, cg072876981 |
| MIR135B | 10 | cg02989808, cg02544160, cg00940313, cg02342533, cg18758230, cg04631281, cg029997112, cg252024072, cg025207071, cg130617671 |
| MIR152 | 10 | cg15179595, cg07418289, cg22409939, cg16346169, cg24521382, cg02140096, cg16154454, cg21043695, cg08172426, cg06164122 |
| MIR1538 | 10 | cg16877681, cg12178237, cg15259593, cg15561647, cg05093686, cg21884062, cg01952313, cg12573705, cg03014934, cg22855860 |
| MIR181B1 | 10 | cg15854570, cg13274183, cg10801670, cg24505619, cg02972607, cg12765886, cg06053959, cg228213581, cg010929321, cg253496431 |
| MIR181C | 10 | cg17341844, cg12823329, cg21548109, cg01232479, cg10726463, cg042721331, cg188756311, cg058655481, cg122421741, cg251471931 |
| MIR181D | 10 | cg04272133, cg18875631, cg05865548, cg12242174, cg18702576, cg173418441, cg128233291, cg215481091, cg012324791, cg211530401 |
| MIR190B | 10 | cg16996262, cg17985281, cg07307803, cg01814186, cg08804013, cg126009011, cg215360861, cg126409421, cg248808181, cg186454931 |
| MIR2117 | 10 | cg04116821, cg04774158, cg04135270, cg04804434, cg27595610, cg067692311, cg023896341, cg011659091, cg031847761, cg256878741 |
| MIR219-2 | 10 | cg20399810, cg05028773, cg08198483, cg16572540, cg19357865, cg145252471, cg104047171, cg204133921, cg144676541, cg006546371 |
| MIR301B | 10 | cg13460319, cg16168683, cg08141518, cg221926141, cg209483661, cg051857381, cg179727891, cg139121961, cg028524211, cg043781071 |
| MIR30B | 10 | cg27547281, cg02351925, cg20375099, cg16804044, cg25458523, cg054256021, cg208310941, cg014173391, cg259647441, cg033310351 |
| MIR320B1 | 10 | cg21898052, cg01301935, cg26670552, cg26967433, cg08665271, cg193213041, cg051565321, cg043908651, cg182747881, cg206322241 |
| MIR370 | 10 | cg25235766, cg02694017, cg13814875, cg17070310, cg04608494, cg098733281, cg013396301, cg137607421, cg233880371, cg140720691 |
| MIR505 | 10 | cg27167381, cg16319308, cg22976112, cg27312241, cg02634141, cg046309821, cg263267461, cg150565721, cg167190991, cg231929181 |
| MIR548AO | 10 | cg16808912, cg04411201, cg26273312, cg05878887, cg06077670, cg06533586, cg00991994, cg15518046, cg06414998, cg225815361 |
| MIR563 | 10 | cg13132363, cg19273756, cg14032089, cg06471491, cg27023597, cg205127132, cg126986622, cg063684012, cg230236042, cg228212202 |
| MIR564 | 10 | cg14399060, cg00767581, cg04248916, cg01923073, cg23157415, cg24386890, cg01093934, cg02049472, cg20841588, cg13236271 |
| MIR602 | 10 | cg18745782, cg09747671, cg10822545, cg26514117, cg24482288, cg146265251, cg002652771, cg215127991, cg111980941, cg062825611 |
| MIR632 | 10 | cg22296874, cg15558727, cg11440148, cg08728669, cg01608962, cg18554216, cg25139619, cg09732987, cg25308542, cg01068014 |
| MIR637 | 10 | cg09565745, cg18485627, cg00594101, cg27039821, cg18804680, cg11887124, cg19945937, cg26255560, cg01971130, cg20526085 |
| MIR659 | 10 | cg07873150, cg21998967, cg15421821, cg06773519, cg18488733, cg01766718, cg02994237, cg06949232, cg107264631, cg187025761 |
| MIR662 | 10 | cg19835331, cg09612552, cg06162516, cg23174344, cg06382345, cg21636685, cg20630582, cg034333131, cg221854281, cg090666761 |
| MIR671 | 10 | cg05001715, cg25771615, cg21830711, cg09848695, cg19875969, cg22703659, cg17533458, cg07803236, cg05880353, cg06401019 |
| MIR922 | 10 | cg10866847, cg15555217, cg08922540, cg26949465, cg10708972, cg07997066, cg06209298, cg03322633, cg12428299, cg03918530 |
| MIR940 | 10 | cg25106676, cg01075038, cg07500168, cg14847493, cg17977969, cg025281541, cg091956572, cg083180852, cg094522571, cg168695471 |
| MIR1197 | 9 | cg01668279, cg121580551, cg262537591, cg154895751, cg228245521, cg256191911, cg232252551, cg267336451, cg061499713 |
| MIR1225 | 9 | cg21544377, cg00874357, cg05102308, cg14108394, cg25084332, cg17099076, cg21883042, cg19484886, cg23118562 |
| MIR1250 | 9 | cg08718220, cg16348475, cg04893124, cg11730290, cg04590632, cg22475368, cg16083780, cg22708188, cg17649996 |
| MIR134 | 9 | cg10248302, cg18009798, cg11730100, cg03114244, cg07583651, cg16916522, cg08873353, cg18799266, cg226624822 |
| MIR140 | 9 | cg12029639, cg13446906, cg25618572, cg24769969, cg07554357, cg14408605, cg02558362, cg20704442, cg04916802 |
| MIR143HG | 9 | cg19505196, cg16821175, cg14208839, cg17068417, cg24727662, cg10231332, cg03040807, cg13846359, cg09306252 |
| MIR190 | 9 | cg02397217, cg07107321, cg08337765, cg16111645, cg11050977, cg17650615, cg00530918, cg01580228, cg21011320 |
| MIR199A1 | 9 | cg21501241, cg16575409, cg20796695, cg11035122, cg09250367, cg13637893, cg23935255, cg21456450, cg08969114 |
| MIR199A2 | 9 | cg12600901, cg21536086, cg12640942, cg24880818, cg18645493, cg20448212, cg17257175, cg06420088, cg03055449 |
| MIR200C | 9 | cg20815911, cg27547695, cg12247976, cg025586841, cg103861111, cg099029081, cg217995231, cg166422991, cg154268151 |
| MIR27A | 9 | cg12044862, cg11780042, cg06961429, cg01131100, cg074296291, cg104001742, cg141480882, cg012445142, cg158621282 |
| MIR296 | 9 | cg01829822, cg15376401, cg00763315, cg083906221, cg259986781, cg012676481, cg138698991, cg149773651, cg251880711 |
| MIR320A | 9 | cg20090508, cg27167181, cg09655487, cg17416790, cg03543120, cg07125256, cg04317047, cg16684117, cg21242144 |
| MIR338 | 9 | cg12367585, cg271160691, cg117015501, cg053659421, cg109216241, cg104600861, cg141330421, cg025233501, cg055802401 |
| MIR339 | 9 | cg24962826, cg06678432, cg07017842, cg02499945, cg05731995, cg11589462, cg01622001, cg13144371, cg01366462 |
| MIR346 | 9 | cg16657582, cg02235235, cg17404487, cg17836499, cg08382292, cg09801901, cg17367733, cg18672880, cg27369048 |
| MIR379 | 9 | cg21823267, cg12791085, cg02357735, cg211671591, cg161532941, cg153172671, cg148663391, cg144402051, cg205625471 |
| MIR382 | 9 | cg11320187, cg102483021, cg075836511, cg180097982, cg117301002, cg031142442, cg088733532, cg169165223, cg075217891 |
| MIR4658 | 9 | cg12093220, cg15939539, cg11175091, cg03204787, cg14640220, cg19426761, cg23666362, cg23740016, cg11976164 |
| MIR484 | 9 | cg19629843, cg09796911, cg09177306, cg19808663, cg14166992, cg18262805, cg02778009, cg16544548, cg22268341 |
| MIR519C | 9 | cg12249359, cg08353446, cg12141088, cg13521944, cg028836662, cg118350501, cg018643611, cg159306781, cg097960291 |
| MIR520G | 9 | cg14358077, cg25675072, cg22672977, cg12879539, cg036639551, cg038079171, cg214563011, cg187123511, cg114612631 |
| MIR645 | 9 | cg02655980, cg20552852, cg01528835, cg07195705, cg01874426, cg213150171, cg090826171, cg093691361, cg067957411 |
| MIR6737 | 9 | cg16383389, cg15641184, cg22090860, cg03226624, cg23243552, cg04293733, cg22333214, cg05423529, cg13417096 |
| MIR885 | 9 | cg25930602, cg02440797, cg21472830, cg11479653, cg18584042, cg22097777, cg08471449, cg25409638, cg14513117 |
| MIR892A | 9 | cg10969470, cg07917516, cg07822388, cg11318772, cg05883757, cg23318169, cg215901801, cg248533131, cg270695881 |
| MIR9-1 | 9 | cg19709754, cg20263826, cg00700455, cg18375960, cg19154516, cg02490260, cg10824792, cg11515530, cg25105745 |
| MIR323 | 9 | cg167324571, cg016682792, cg262537592, cg154895752, cg228245523, cg256191913, cg232252553, cg267336453, cg213622333 |
| MIR371 | 9 | cg111348011, cg100068871, cg071263991, cg030500961, cg109480701, cg212300211, cg046721821, cg021807371, cg079184531 |
| MIR1-1HG | 8 | cg09672452, cg20033079, cg20070464, cg18806815, cg10193091, cg17365618, cg01542115, cg12131601 |
| MIR1185-2 | 8 | cg16407471, cg10715092, cg02624565, cg09687946, cg053763741, cg029750601, cg131927141, cg135466091 |
| MIR1208 | 8 | cg02479575, cg26005082, cg07642566, cg21927946, cg117928261, cg244654821, cg004447401, cg070181071 |
| MIR122 | 8 | cg01388803, cg27254118, cg02017733, cg13590605, cg193123051, cg129074771, cg052491551, cg004812801 |
| MIR1226 | 8 | cg16588417, cg09633152, cg16427534, cg00789792, cg04486436, cg24192368, cg21182526, cg18251859 |
| MIR1229 | 8 | cg16179239, cg01895189, cg22008020, cg09704444, cg05171064, cg19550890, cg14553506, cg23059797 |
| MIR1238 | 8 | cg14440205, cg20562547, cg14410227, cg18440069, cg13278105, cg02691745, cg20067612, cg00989505 |
| MIR124-1 | 8 | cg06536614, cg25340688, cg26896946, cg00124993, cg08745965, cg18797653, cg19032799, cg04184179 |
| MIR1259 | 8 | cg04521583, cg10579797, cg14661236, cg20636382, cg23505252, cg15372290, cg08436772, cg08824909 |
| MIR125A | 8 | cg13753460, cg04761768, cg21103170, cg06934265, cg23488816, cg14431528, cg01119169, cg114398692 |
| MIR128-2 | 8 | cg18637574, cg17668949, cg12480658, cg15573536, cg01737698, cg19045894, cg24000528, cg23460578 |
| MIR133B | 8 | cg02292342, cg05766064, cg25890168, cg02645550, cg069035791, cg144875771, cg038242381, cg104881991 |
| MIR138-2 | 8 | cg14197404, cg23422270, cg18986745, cg08248790, cg264750941, cg029618081, cg198360881, cg077372921 |

| | | |
|---|---|---|
| MIR145 | 8 | cg09825167, cg21090893, cg22484330, cg04394119, cg13090484, cg16794867, cg01965463, cg26827139 |
| MIR1468 | 8 | cg05776098, cg01651728, cg26551791, cg00739582, cg008689801, cg107944731, cg082641241, cg084648601 |
| MIR153-2 | 8 | cg16695999, cg00964137, cg03964851, cg22733357, cg09753632, cg23504723, cg10511110, cg07988843 |
| MIR183 | 8 | cg08827075, cg15700520, cg14209764, cg04959716, cg26423185, cg04828267, cg18269595, cg08322697 |
| MIR1914 | 8 | cg01154656, cg24305693, cg05079405, cg21938506, cg13330363, cg12945769, cg03066823, cg27357049 |
| MIR195 | 8 | cg24757752, cg26995690, cg02580944, cg003397261, cg026361621, cg086133501, cg203714371, cg112357871 |
| MIR200A | 8 | cg02331673, cg11203990, cg13362546, cg21810793, cg134495351, cg071234811, cg015374941, cg028253441 |
| MIR200B | 8 | cg13449535, cg07123481, cg01537494, cg02825344, cg01723420, cg00204984, cg134495352, cg071234812 |
| MIR222 | 8 | cg02060185, cg01340286, cg08291487, cg065020711, cg161088351, cg256750721, cg226729771, cg128795391 |
| MIR2276 | 8 | cg11512305, cg07089575, cg23404491, cg26087577, cg25884222, cg07426997, cg05226457, cg19950186 |
| MIR24-1 | 8 | cg02588532, cg23804785, cg09089341, cg09406630, cg17871838, cg05005235, cg146496651, cg187624221 |
| MIR29A | 8 | cg08893293, cg15190406, cg10094037, cg073647601, cg060174051, cg113700111, cg107580221, cg075584421 |
| MIR30C2 | 8 | cg15671915, cg21024311, cg16915294, cg07202461, cg143515261, cg255667871, cg253068831, cg153190271 |
| MIR320D1 | 8 | cg03652429, cg20467339, cg10583119, cg17129943, cg234835622, cg217946652, cg164307262, cg087150091 |
| MIR34A | 8 | cg25283230, cg00327399, cg23810424, cg03013259, cg009097061, cg106403891, cg099947731, cg245012301 |
| MIR34B | 8 | cg08882264, cg23532270, cg06227369, cg09008353, cg04522339, cg084719501, cg113474751, cg001229511 |
| MIR508 | 8 | cg18904817, cg11166197, cg12182101, cg04227631, cg262188231, cg222625441, cg013748851, cg131542341 |
| MIR5188 | 8 | cg22189216, cg09449167, cg11499685, cg16423770, cg05273161, cg11000817, cg00291004, cg03014004 |
| MIR521-2 | 8 | cg09937725, cg15944251, cg18703109, cg06453471, cg244807351, cg081886091, cg001011041, cg190952191 |
| MIR557 | 8 | cg00158530, cg08209934, cg04703221, cg03885048, cg069064621, cg111570341, cg044367011, cg052125431 |
| MIR572 | 8 | cg18982976, cg15369381, cg15163417, cg21952149, cg252065361, cg042037021, cg066065391, cg259604031 |
| MIR575 | 8 | cg27434411, cg08078966, cg03277051, cg20948024, cg18056266, cg09782454, cg19968403, cg02441647 |
| MIR647 | 8 | cg17286244, cg23698666, cg24750854, cg219385061, cg133303631, cg129457691, cg030668231, cg273570491 |
| MIR6516 | 8 | cg19491776, cg20126937, cg00561206, cg17424516, cg19081329, cg21790677, cg01230180, cg02114711 |
| MIR6816 | 8 | cg27417749, cg23365173, cg00309133, cg19993316, cg22381808, cg20363989, cg07057074, cg13086606 |
| MIR6850 | 8 | cg17774634, cg23858074, cg00472393, cg11182931, cg22682162, cg22566643, cg10518340, cg21877580 |
| MIR759 | 8 | cg14209677, cg09430586, cg23061412, cg08030633, cg045652011, cg178431241, cg137670011, cg227502731 |
| MIR877 | 8 | cg05789925, cg20674424, cg24082174, cg05199755, cg27374881, cg00670721, cg01712079, cg23010205 |
| MIR889 | 8 | cg10512089, cg17680641, cg05046026, cg25628257, cg26061001, cg08095361, cg201765731, cg032906021 |
| MIR890 | 8 | cg13154234, cg04037410, cg08111264, cg058150341, cg123877401, cg082914871, cg057760981, cg016517281 |
| MIR937 | 8 | cg12974668, cg25360535, cg21789597, cg02074259, cg03820148, cg11746148, cg05491093, cg22216174 |
| MIR941-1 | 8 | cg15533448, cg08397218, cg10302164, cg01280098, cg060491712, cg060226642, cg202381282, cg157730802 |
| MIR943 | 8 | cg24674304, cg25189074, cg13740840, cg23569156, cg10911865, cg07542475, cg02160333, cg17410922 |
| MIRLET7A3 | 8 | cg15832108, cg26584653, cg21318213, cg04330371, cg23042706, cg02278499, cg18614734, cg01501208 |
| MIR376C | 8 | cg260246821, cg142940962, cg076160942, cg044586454, cg185146444, cg110748144, cg063720154, cg149012054 |
| MIR381HG | 8 | cg161507981, cg020053361, cg227047751, cg038320782, cg233712082, cg201765732, cg125808203, cg061144653 |
| MIR1207 | 7 | cg27331144, cg02214637, cg20255906, cg00468163, cg05525200, cg18007341, cg03903398 |
| MIR1236 | 7 | cg15435900, cg10579986, cg06336230, cg17304531, cg19463199, cg12023911, cg27013765 |
| MIR124-2HG | 7 | cg06189266, cg09133836, cg03369432, cg23035172, cg12697637, cg04851992, cg00004915 |
| MIR1260 | 7 | cg23172333, cg12661000, cg20540672, cg12454619, cg23446857, cg20644425, cg016321881 |
| MIR1281 | 7 | cg26291655, cg12058064, cg06527213, cg07741205, cg08867933, cg08287343, cg081312041 |
| MIR1286 | 7 | cg17641440, cg26929513, cg21605389, cg13343238, cg10098175, cg25584422, cg07148591 |
| MIR1301 | 7 | cg18987126, cg22159815, cg10234282, cg20701901, cg08855249, cg13093042, cg15076217 |
| MIR149 | 7 | cg14592981, cg16498879, cg15928974, cg26837747, cg16208848, cg07510373, cg04840148 |
| MIR1539 | 7 | cg06154883, cg07355632, cg21434113, cg20793562, cg00453774, cg14980467, cg10799802 |
| MIR1913 | 7 | cg11732729, cg08162457, cg10503919, cg06833610, cg07848274, cg13018338, cg10380484 |
| MIR1976 | 7 | cg21986272, cg24343445, cg21784980, cg07298692, cg06786434, cg06271655, cg00209355 |
| MIR22 | 7 | cg04476865, cg13027104, cg15421870, cg06641651, cg07617482, cg08761167, cg105646261 |
| MIR22HG | 7 | cg11657317, cg10564626, cg09505337, cg21100351, cg08981212, cg13953455, cg15535534 |
| MIR23A | 7 | cg07429629, cg10400174, cg141480881, cg012445141, cg158621281, cg069614292, cg011311002 |
| MIR26A1 | 7 | cg19519747, cg07011913, cg12127282, cg08717880, cg27160395, cg17104824, cg00014998 |
| MIR2861 | 7 | cg00002531, cg26768703, cg00872676, cg04840800, cg159089751, cg116945131, cg033263991 |
| MIR326 | 7 | cg12389845, cg12094723, cg07103258, cg02133084, cg12538810, cg12495376, cg07773515 |
| MIR33A | 7 | cg23176340, cg01925965, cg09852187, cg22456251, cg03682656, cg01407044, cg00576773 |
| MIR3656 | 7 | cg08956415, cg17768313, cg03870472, cg21021332, cg10456132, cg02254327, cg20292224 |
| MIR378D2 | 7 | cg08486060, cg24291455, cg06831953, cg22827729, cg22134024, cg26601590, cg03915569 |
| MIR381 | 7 | cg03832078, cg08802738, cg132029361, cg130980911, cg125808201, cg061144651, cg136778591 |
| MIR421 | 7 | cg02533339, cg22388260, cg028862631, cg272887411, cg011092431, cg004850471, cg221780851 |
| MIR4496 | 7 | cg22415746, cg04573316, cg24480735, cg08188609, cg051010192, cg078834072, cg175880912 |
| MIR450B | 7 | cg01782749, cg15186531, cg239231791, cg176730591, cg252223571, cg222242571, cg246671681 |
| MIR483 | 7 | cg12957092, cg10801015, cg06204973, cg27238381, cg03463578, cg19776344, cg20238164 |
| MIR486 | 7 | cg24404973, cg05063169, cg17872779, cg23066234, cg18319687, cg19726536, cg16578938 |
| MIR499 | 7 | cg24705841, cg09976282, cg01684389, cg06314969, cg13905606, cg20260028, cg08096702 |
| MIR502 | 7 | cg09195657, cg08318085, cg09452257, cg16869547, cg08683389, cg07224637, cg13882748 |
| MIR506 | 7 | cg18471285, cg178633261, cg169120301, cg097882171, cg250842791, cg056034401, cg271673811 |
| MIR515-1 | 7 | cg06753050, cg02883666, cg214921371, cg052492711, cg155082801, cg092131651, cg143559411 |
| MIR515-2 | 7 | cg21492137, cg05249271, cg15508280, cg09213165, cg14355941, cg028836661, cg067530502 |
| MIR520C | 7 | cg23468699, cg04063235, cg22121941, cg16114706, cg131225321, cg176702631, cg240148491 |
| MIR525 | 7 | cg15171962, cg21331510, cg24352349, cg04880355, cg225373431, cg263254971, cg215928031 |
| MIR526A1 | 7 | cg24911721, cg13251842, cg03841312, cg234686991, cg094042891, cg141198271, cg188779611 |
| MIR618 | 7 | cg07704578, cg15133882, cg00476022, cg03835940, cg23563692, cg25774694, cg08000025 |
| MIR6790 | 7 | cg25063505, cg18862502, cg11205335, cg14937446, cg06792448, cg02675985, cg19603885 |
| MIR6848 | 7 | cg25566787, cg25306883, cg15319027, cg19098437, cg20815778, cg26053571, cg01434892 |
| MIR892B | 7 | cg21590180, cg15033973, cg109694701, cg079175161, cg078223881, cg113187721, cg198780171 |
| MIR921 | 7 | cg18616702, cg16414821, cg18135087, cg02890365, cg10688896, cg27157872, cg10687301 |
| MIR935 | 7 | cg05537796, cg14007694, cg22977139, cg25122402, cg19944840, cg21878393, cg18269894 |
| MIR941-3 | 7 | cg06049171, cg06022664, cg20238128, cg15773080, cg083972181, cg103021641, cg012800981 |
| MIR941-2 | 7 | cg060491711, cg060226641, cg202381281, cg157730801, cg083972182, cg103021642, cg012800982 |
| MIRLET7E | 7 | cg114398691, cg234888161, cg144315281, cg137534602, cg047617682, cg211031702, cg069342652 |
| MIR103-2 | 6 | cg18804615, cg00741900, cg09982291, cg27314336, cg13673833, cg23960324 |
| MIR105-1 | 6 | cg17273098, cg21555172, cg11525063, cg16964357, cg21006034, cg095578881 |
| MIR1179 | 6 | cg10842070, cg05898333, cg03805896, cg077412051, cg088679331, cg221449421 |
| MIR1205 | 6 | cg25511278, cg07725389, cg01910419, cg19356389, cg26106778, cg11011640 |
| MIR1248 | 6 | cg22978384, cg02179478, cg09736928, cg08490094, cg24102420, cg21384588 |
| MIR1251 | 6 | cg06845425, cg10183426, cg22459858, cg15841006, cg12326635, cg22166550 |
| MIR1269A | 6 | cg09025253, cg12404798, cg23473419, cg141313131, cg255213751, cg041005491 |
| MIR1280 | 6 | cg14084144, cg23743058, cg04813781, cg05037455, cg12285570, cg24119006 |
| MIR1282 | 6 | cg24286190, cg22862808, cg07099752, cg26703857, cg12293583, cg16249681 |
| MIR1287 | 6 | cg13413777, cg01344051, cg21968223, cg06264430, cg03248910, cg13435938 |
| MIR1289-1 | 6 | cg16271911, cg16930925, cg22894805, cg22233082, cg14472937, cg15563355 |
| MIR1302-1 | 6 | cg00101104, cg19095219, cg25843705, cg169114872, cg008461362, cg204700832 |
| MIR1305 | 6 | cg05394852, cg23471991, cg21586296, cg116772121, cg133000241, cg126550941 |
| MIR1322 | 6 | cg03894789, cg22030962, cg18251187, cg04986004, cg23186640, cg25520280 |
| MIR133A2 | 6 | cg04182114, cg23486561, cg09610163, cg18065177, cg02587412, cg19700150 |
| MIR135A1 | 6 | cg18351741, cg26222940, cg04959250, cg13547672, cg052696321, cg186759021 |
| MIR146A | 6 | cg24688837, cg23535449, cg11236755, cg171205881, cg083507391, cg033062401 |
| MIR147B | 6 | cg24213572, cg06075793, cg26859772, cg23904570, cg13965908, cg16020638 |
| MIR16-2 | 6 | cg23023604, cg22821220, cg24987741, cg205127131, cg126986621, cg063684011 |
| MIR181A1 | 6 | cg00066270, cg04788442, cg158545701, cg132741831, cg108016701, cg245056191 |
| MIR1827 | 6 | cg27664418, cg23319790, cg24858376, cg126383571, cg131735411, cg242326621 |
| MIR185 | 6 | cg08039084, cg06506623, cg05451359, cg11098984, cg09638686, cg13348907 |
| MIR205 | 6 | cg20041381, cg24577137, cg26803837, cg13365658, cg27342087, cg24356321 |
| MIR2054 | 6 | cg25333216, cg00819114, cg03570858, cg119761641, cg133325521, cg225767111 |
| MIR211 | 6 | cg08793239, cg21144158, cg15595954, cg10090749, cg13167340, cg22040449 |
| MIR2113 | 6 | cg05827233, cg18981979, cg18720617, cg251995911, cg061389661, cg098362051 |
| MIR218-1 | 6 | cg21933155, cg20436810, cg19219068, cg09150212, cg11565377, cg01112720 |
| MIR218-2 | 6 | cg15847886, cg26663696, cg13783238, cg12785694, cg17554896, cg04001997 |
| MIR220A | 6 | cg27617163, cg25696807, cg08967287, cg086346571, cg076535501, cg058789681 |
| MIR223 | 6 | cg16380536, cg05175213, cg24853313, cg067011911, cg191278401, cg137160341 |
| MIR26A2 | 6 | cg10096536, cg14130004, cg01951972, cg20134331, cg13496359, cg03602781 |
| MIR26B | 6 | cg01035219, cg26266985, cg18480318, cg25317338, cg26833654, cg14506197 |
| MIR29B2 | 6 | cg10387807, cg14311597, cg07169873, cg043124131, cg225258951, cg034463991 |
| MIR301A | 6 | cg01772760, cg12585342, cg08218990, cg21049840, cg11982292, cg23333994 |

| | | |
|---|---|---|
| MIR302A | 6 | cg17207485, cg211756851, cg210107152, cg016281812, cg151657352, cg167592183 |
| MIR302D | 6 | cg21175685, cg15165735, cg172074851, cg167592181, cg210107151, cg016281811 |
| MIR30C1 | 6 | cg23955417, cg11377047, cg24993174, cg12515083, cg12999797, cg03698781 |
| MIR30D | 6 | cg14684642, cg04942111, cg00083848, cg166574341, cg016092141, cg189756901 |
| MIR30E | 6 | cg15707579, cg24440131, cg00359365, cg24468934, cg16395432, cg09641213 |
| MIR325HG | 6 | cg12706810, cg16786260, cg13273037, cg042718701, cg153072301, cg229160421 |
| MIR331 | 6 | cg13122532, cg17670263, cg24014849, cg166674631, cg116908241, cg099957361 |
| MIR363 | 6 | cg07994786, cg08085487, cg059247411, cg129660051, cg085586521, cg204544291 |
| MIR367 | 6 | cg26105803, cg16759218, cg21010715, cg01628181, cg172074852, cg211756854 |
| MIR3976HG | 6 | cg22797479, cg04765078, cg00379323, cg177555091, cg051129031, cg188222711 |
| MIR424 | 6 | cg17685131, cg07685228, cg13627776, cg04973399, cg12954379, cg146913072 |
| MIR4462 | 6 | cg11124635, cg17755509, cg05112903, cg038201481, cg117461481, cg054910931 |
| MIR449A | 6 | cg09271158, cg17047890, cg20009349, cg05906075, cg006952711, cg068884631 |
| MIR451 | 6 | cg09532726, cg23483562, cg21794665, cg16430726, cg08736070, cg098056921 |
| MIR4517 | 6 | cg02387491, cg02554051, cg22986737, cg08784247, cg00323458, cg02585417 |
| MIR4640 | 6 | cg19400113, cg09085792, cg15245749, cg06277638, cg27227930, cg04605696 |
| MIR4649 | 6 | cg27025274, cg20185964, cg15335915, cg27064284, cg03585049, cg17431830 |
| MIR4716 | 6 | cg10548355, cg02797411, cg10051714, cg00804634, cg22123156, cg19245310 |
| MIR4720 | 6 | cg12787185, cg12975010, cg15200151, cg186154721, cg256944981, cg020343301 |
| MIR497 | 6 | cg00339726, cg02636162, cg08613350, cg247577521, cg269956901, cg203714372 |
| MIR498 | 6 | cg23690344, cg16495809, cg04424420, cg014117591, cg228443681, cg103622941 |
| MIR513C | 6 | cg15341392, cg00018204, cg14686949, cg139216801, cg151865311, cg025626931 |
| MIR514B | 6 | cg07311296, cg11428427, cg18312428, cg040374101, cg081112641, cg145833611 |
| MIR518B | 6 | cg26371705, cg08695558, cg15702185, cg112515541, cg159937861, cg064459811 |
| MIR518C | 6 | cg06720082, cg08263387, cg20967343, cg227259011, cg217441361, cg156160061 |
| MIR518D | 6 | cg07452499, cg27463946, cg10774723, cg036524291, cg204673391, cg105831191 |
| MIR520E | 6 | cg15399181, cg21872942, cg24260710, cg221666331, cg273520151, cg157834521 |
| MIR520F | 6 | cg17473398, cg04181153, cg02233213, cg231736471, cg141130461, cg103829671 |
| MIR526A2 | 6 | cg20963460, cg16879820, cg26715986, cg246991321, cg187485801, cg159377841 |
| MIR526B | 6 | cg13766329, cg02144647, cg196312641, cg261169691, cg230978431, cg000471851 |
| MIR542 | 6 | cg02562693, cg20343467, cg252223572, cg222242572, cg083032281, cg107966031 |
| MIR548A1 | 6 | cg08106445, cg10914865, cg14140599, cg266015901, cg039155691, cg030007111 |
| MIR548AU | 6 | cg09571887, cg04706583, cg06264199, cg07930121, cg100201201, cg056170501 |
| MIR548AW | 6 | cg10893656, cg12877853, cg15187151, cg26308704, cg06747087, cg06179179 |
| MIR548I2 | 6 | cg06360318, cg18122056, cg04093078, cg218366991, cg276637161, cg081748901 |
| MIR551A | 6 | cg16561256, cg26330479, cg06967105, cg23651812, cg03528302, cg16712243 |
| MIR551B | 6 | cg01047111, cg20505332, cg20015729, cg12416878, cg16119643, cg11782594 |
| MIR558 | 6 | cg07664909, cg00823526, cg22525895, cg03446399, cg18591304, cg27261597 |
| MIR5707 | 6 | cg02124727, cg05079117, cg22091742, cg233805521, cg019124281, cg083803161 |
| MIR597 | 6 | cg10205925, cg08360457, cg03307581, cg14205800, cg04481923, cg18678645 |
| MIR6125 | 6 | cg02408243, cg01418386, cg18508449, cg22785801, cg13973641, cg23330818 |
| MIR614 | 6 | cg18710710, cg10753636, cg23097843, cg202062041, cg053468311, cg245876251 |
| MIR629 | 6 | cg12161331, cg02624246, cg02753020, cg13786932, cg08411094, cg11297610 |
| MIR631 | 6 | cg05346831, cg24587625, cg10564060, cg05305603, cg06771291, cg13277047 |
| MIR650 | 6 | cg00966524, cg15380873, cg00583858, cg227974791, cg047650781, cg003793231 |
| MIR6733 | 6 | cg13110636, cg18983289, cg03909081, cg26820000, cg07387734, cg14092259 |
| MIR6825 | 6 | cg01058850, cg12035782, cg25883149, cg06046580, cg07513814, cg09110634 |
| MIR718 | 6 | cg13615360, cg04868709, cg14929554, cg10849147, cg11063021, cg17567941 |
| MIR744 | 6 | cg08031206, cg19552794, cg21880213, cg09355500, cg05107190, cg07249860 |
| MIR762 | 6 | cg15862706, cg10525574, cg20220255, cg16667463, cg11690824, cg09995736 |
| MIR765 | 6 | cg19321304, cg05156532, cg04390865, cg18274788, cg20632224, cg09099500 |
| MIR874 | 6 | cg08840687, cg22894604, cg00445142, cg23405198, cg17473262, cg01184905 |
| MIR887 | 6 | cg07805332, cg17388683, cg08539350, cg13477253, cg03275067, cg15077343 |
| MIR888 | 6 | cg05815034, cg12387740, cg14583361, cg26404952, cg163805361, cg051752131 |
| MIR891A | 6 | cg08316488, cg13890773, cg16618605, cg256968071, cg089672871, cg080854871 |
| MIR891B | 6 | cg21870760, cg23570034, cg23698976, cg159185871, cg079277181, cg276171631 |
| MIR17 | 6 | cg020677411, cg149536871, cg073812312, cg081933762, cg267537813, cg267894124 |
| MIR19B1 | 6 | cg059655351, cg237417022, cg249420392, cg096619243, cg267537814, cg267894125 |
| MIR541 | 6 | cg202950711, cg239924491, cg132821802, cg271733223, cg119855624, cg084607104 |
| MIR18A | 6 | cg149536872, cg073812313, cg081933763, cg237417024, cg267537815, 1 |
| MIR106A | 5 | cg13921680, cg059247412, cg129660053, cg085586523, cg207884151 |
| MIR1178 | 5 | cg14862827, cg21046659, cg24239321, cg22887281, cg04291471 |
| MIR1203 | 5 | cg09379151, cg04430472, cg19567023, cg06215240, cg11857748 |
| MIR1227 | 5 | cg26748435, cg14111726, cg27267322, cg02284419, cg09297676 |
| MIR1231 | 5 | cg12626411, cg02758410, cg11245017, cg13801259, cg11227300 |
| MIR125B2 | 5 | cg21260288, cg21155118, cg21274136, cg07987890, cg21963854 |
| MIR126 | 5 | cg09899173, cg12142501, cg15821939, cg16481961, cg08865574 |
| MIR1272 | 5 | cg27164575, cg21173689, cg05401712, cg11617306, cg26122422 |
| MIR1278 | 5 | cg19261968, cg09910993, cg13459734, cg24264066, cg14451430 |
| MIR1283-2 | 5 | cg14906110, cg12198632, cg01530912, cg262427721, cg169088241 |
| MIR1290 | 5 | cg13560937, cg20524150, cg20207416, cg17344234, cg10053134 |
| MIR1296 | 5 | cg00471826, cg06820725, cg05527782, cg20196141, cg26454401 |
| MIR133A1 | 5 | cg21615907, cg06653974, cg21647636, cg005797341, cg048239581 |
| MIR143 | 5 | cg18682381, cg27114354, cg14083040, cg11037184, cg00258015 |
| MIR15B | 5 | cg20512713, cg12698662, cg06368401, cg230236041, cg228212201 |
| MIR188 | 5 | cg08983215, cg10268345, cg05598886, cg267752891, cg161590901 |
| MIR194-1 | 5 | cg08910524, cg07667243, cg217517331, cg105240701, cg130659381 |
| MIR19A | 5 | cg07381231, cg08193376, cg26753781, cg26789412, cg237417025 |
| MIR203A | 5 | cg06103064, cg15428904, cg03344985, cg099822911, cg244547842 |
| MIR208B | 5 | cg00415455, cg27438067, cg04390575, cg27116149, cg10537327 |
| MIR2116 | 5 | cg08960830, cg05473648, cg25837979, cg17233829, cg04726985 |
| MIR215 | 5 | cg21751733, cg10524070, cg13065938, cg089105241, cg076672431 |
| MIR216B | 5 | cg22688137, cg26403285, cg02249795, cg011608551, cg020715531 |
| MIR23B | 5 | cg23676215, cg00884195, cg16970310, cg146496652, cg187624222 |
| MIR29B1 | 5 | cg07364760, cg06017405, cg21477068, cg00968931, cg020000321 |
| MIR3196 | 5 | cg08116550, cg01130777, cg18901478, cg12002820, cg10537328 |
| MIR3197 | 5 | cg00687674, cg18263455, cg23036340, cg18445764, cg14851122 |
| MIR328 | 5 | cg00861695, cg15042891, cg07152812, cg22412649, cg10587183 |
| MIR340 | 5 | cg10187475, cg00299558, cg17009978, cg12821315, cg03981074 |
| MIR342 | 5 | cg08482145, cg15809032, cg17625407, cg07991335, cg015029791 |
| MIR362 | 5 | cg07036112, cg18447131, cg01965047, cg13949829, cg02879554 |
| MIR3621 | 5 | cg00678005, cg25708403, cg13048261, cg06142662, cg25304543 |
| MIR3652 | 5 | cg07494164, cg15067802, cg10974064, cg11929218, cg08115465 |
| MIR3663HG | 5 | cg03311185, cg10794439, cg058366591, cg085046011, cg136039141 |
| MIR374A | 5 | cg07370023, cg09730820, cg20342118, cg09662369, cg177370881 |
| MIR374B | 5 | cg02886263, cg27288741, cg01109243, cg00485047, cg22178085 |
| MIR378A | 5 | cg01030411, cg27641106, cg24720939, cg00972731, cg16245944 |
| MIR383 | 5 | cg13688408, cg01726320, cg26585864, cg00113247, cg16109075 |
| MIR3912 | 5 | cg16062053, cg15164276, cg17528257, cg21658839, cg09652501 |
| MIR3913-1 | 5 | cg08527343, cg08995067, cg08881769, cg07738306, cg12776892 |
| MIR3917 | 5 | cg22904086, cg13252549, cg02650522, cg25174210, cg26824639 |
| MIR4458HG | 5 | cg00351980, cg14460963, cg15164782, cg25162651, cg24123724 |
| MIR449B | 5 | cg00695271, cg06888463, cg170478901, cg200093491, cg059060751 |
| MIR450A1 | 5 | cg23923179, cg17673059, cg25222357, cg22224257, cg017827491 |
| MIR454 | 5 | cg12507840, cg09114848, cg16178786, cg04627415, cg00033818 |
| MIR455 | 5 | cg19680554, cg20422157, cg22266405, cg14224139, cg22018180 |
| MIR489 | 5 | cg13083186, cg06265999, cg15272005, cg12874961, cg14301438 |
| MIR507 | 5 | cg17863326, cg16912030, cg09788217, cg25084279, cg05603440 |
| MIR516A1 | 5 | cg00582864, cg21206555, cg149061101, cg156040441, cg098524391 |
| MIR5187 | 5 | cg25940485, cg19582647, cg01918824, cg00324023, cg24771345 |
| MIR518E | 5 | cg03662899, cg01843978, cg051389571, cg247879241, cg001097761 |
| MIR519A1 | 5 | cg22542859, cg05193141, cg18123612, cg267575621, cg253499811 |
| MIR545 | 5 | cg17737088, cg073700231, cg097308201, cg203421181, cg096623691 |

| | | |
|---|---|---|
| MIR548AY | 5 | cg15688583, cg08004406, cg26022159, cg04591012, cg23480273 |
| MIR548C | 5 | cg01590557, cg10545141, cg09779010, cg05205299, cg14588738 |
| MIR548D1 | 5 | cg11722227, cg13575606, cg11929960, cg10806543, cg12338747 |
| MIR548J | 5 | cg22363909, cg18503694, cg18146964, cg19563248, cg00260818 |
| MIR549 | 5 | cg09300189, cg20984590, cg18838148, cg02911529, cg10662314 |
| MIR585 | 5 | cg09100593, cg24502614, cg19110521, cg21490751, cg07747690 |
| MIR590 | 5 | cg15466196, cg16437766, cg13671094, cg21321611, cg23551256 |
| MIR593 | 5 | cg23107222, cg11677212, cg13300024, cg12655094, cg02223783 |
| MIR595 | 5 | cg19152802, cg02562921, cg03214468, cg15254366, cg20943266 |
| MIR598 | 5 | cg24920847, cg17915286, cg14759064, cg02726137, cg02370376 |
| MIR609 | 5 | cg21978026, cg10280702, cg01272629, cg16922635, cg03340334 |
| MIR623 | 5 | cg03131516, cg11295002, cg12207024, cg00425865, cg12146158 |
| MIR628 | 5 | cg18821418, cg22969079, cg17074821, cg03932760, cg16137891 |
| MIR635 | 5 | cg27126814, cg04573702, cg21828716, cg05241732, cg25428233 |
| MIR641 | 5 | cg13677859, cg00844429, cg26233951, cg10177827, cg25296532 |
| MIR642 | 5 | cg16438722, cg01586072, cg03019112, cg00395657, cg03059138 |
| MIR643 | 5 | cg21071540, cg23940023, cg09291131, cg03153765, cg00594228 |
| MIR646 | 5 | cg03144814, cg06706204, cg20615141, cg13174229, cg07567679 |
| MIR6508 | 5 | cg16774511, cg15311954, cg02002788, cg15098410, cg24399529 |
| MIR6787 | 5 | cg04933432, cg04615725, cg09663027, cg16057182, cg15773296 |
| MIR6843 | 5 | cg24549289, cg17856005, cg27083040, cg23917868, cg11671363 |
| MIR6853 | 5 | cg09836205, cg19366816, cg03697143, cg27364880, cg12396622 |
| MIR6854 | 5 | cg07883606, cg27205928, cg16492851, cg04682977, cg10089657 |
| MIR6892 | 5 | cg14042821, cg08478427, cg21470876, cg05239577, cg25013978 |
| MIR92A1 | 5 | cg24942039, cg096619241, cg267894122, cg059655352, cg237417023 |
| MIR934 | 5 | cg12081759, cg04433936, cg16824290, cg24714883, cg23491344 |
| MIR936 | 5 | cg18616114, cg14420620, cg02356258, cg03287314, cg16047262 |
| MIR939 | 5 | cg21303169, cg16506910, cg01211496, cg16152480, cg25275283 |
| MIR942 | 5 | cg12746041, cg05851510, cg09254646, cg08696889, cg09556871 |
| MIR99B | 5 | cg11439869, cg137534601, cg047617681, cg211031701, cg069342651 |
| MIRLET7C | 5 | cg07344019, cg23966476, cg18149794, cg20189274, cg17635774 |
| MIRLET7F2 | 5 | cg07665535, cg21396064, cg24229568, cg040183251, cg062730751 |
| MIR500 | 5 | cg070361121, cg184471311, cg019650471, cg139498291, cg028795541 |
| MIR302C | 5 | cg167592182, cg211756853, cg210107153, cg016281813, cg151657353 |
| MIR107 | 4 | cg01128614, cg15295468, cg11625387, cg23100309 |
| MIR1199 | 4 | cg18805031, cg10270238, cg21146428, cg10300864 |
| MIR1202 | 4 | cg16126877, cg01878705, cg160761911, cg104943301 |
| MIR1252 | 4 | cg14478242, cg12935627, cg11255687, cg21025168 |
| MIR1263 | 4 | cg01465350, cg05705892, cg245026141, cg191105211 |
| MIR1266 | 4 | cg07588932, cg08377894, cg08536044, cg25899829 |
| MIR1267 | 4 | cg23302214, cg16903347, cg14435720, cg17485016 |
| MIR1284 | 4 | cg06125055, cg11969913, cg10949583, cg03283842 |
| MIR1288 | 4 | cg15207708, cg21629505, cg19350059, cg23814365 |
| MIR1289-2 | 4 | cg08723913, cg18627042, cg16358924, cg02771886 |
| MIR1302-4 | 4 | cg09342858, cg15035143, cg105800671, cg009418331 |
| MIR1307 | 4 | cg19578234, cg14684255, cg04424780, cg12650829 |
| MIR1324 | 4 | cg20836224, cg27097489, cg056843711, cg105638341 |
| MIR133A1HG | 4 | cg00579734, cg04823958, cg24025859, cg05185154 |
| MIR1343 | 4 | cg18549594, cg02742934, cg14732116, cg00088264 |
| MIR139 | 4 | cg09056223, cg02672776, cg02224202, cg14719465 |
| MIR147 | 4 | cg03216043, cg13965612, cg225762651, cg093992251 |
| MIR148B | 4 | cg05022212, cg06812263, cg06889514, cg04451615 |
| MIR151B | 4 | cg01502979, cg25194512, cg176254071, cg079913351 |
| MIR153-1 | 4 | cg16450290, cg20769477, cg03085443, cg07330251 |
| MIR1537 | 4 | cg03386320, cg24379085, cg11984368, cg09398207 |
| MIR155 | 4 | cg11926525, cg19539048, cg08413060, cg040286041 |
| MIR186 | 4 | cg22525715, cg00763223, cg13691257, cg14777634 |
| MIR1910 | 4 | cg25192925, cg08520986, cg18830373, cg26700590 |
| MIR1911 | 4 | cg08378275, cg11564551, cg26351511, cg16956574 |
| MIR197 | 4 | cg06386401, cg25534638, cg115155301, cg251057451 |
| MIR1973 | 4 | cg16046954, cg23263051, cg236663621, cg237400161 |
| MIR1974 | 4 | cg04569855, cg04521697, cg00573148, cg09957895 |
| MIR1975 | 4 | cg07975356, cg26417468, cg09229423, cg02268620 |
| MIR1978 | 4 | cg26645003, cg23617067, cg24658737, cg19530142 |
| MIR198 | 4 | cg05729783, cg27002958, cg18906489, cg06832651 |
| MIR199B | 4 | cg09344485, cg11364290, cg04524088, cg240441861 |
| MIR2052 | 4 | cg21887303, cg24943002, cg070357041, cg267542621 |
| MIR2053 | 4 | cg24636809, cg25613385, cg23222808, cg18577693 |
| MIR210HG | 4 | cg20653075, cg07749923, cg054557201, cg165935521 |
| MIR2278 | 4 | cg10494330, cg08759764, cg23570321, cg13057938 |
| MIR27B | 4 | cg14649665, cg18762422, cg008841951, cg169703101 |
| MIR298 | 4 | cg08390622, cg25998678, cg01267648, cg26431773 |
| MIR3150B | 4 | cg24763265, cg00329803, cg197763441, cg202381641 |
| MIR3175 | 4 | cg03668470, cg13038544, cg07124859, cg21702971 |
| MIR320C2 | 4 | cg16964791, cg19324608, cg26779688, cg02027685 |
| MIR320D2 | 4 | cg09721969, cg26218823, cg174887851, cg019631471 |
| MIR337 | 4 | cg15675054, cg067742071, cg232199051, cg225326521 |
| MIR3607 | 4 | cg22092811, cg18337222, cg03671265, cg13790797 |
| MIR3609 | 4 | cg25521375, cg04100549, cg05672616, cg04237666 |
| MIR365A | 4 | cg02711653, cg00407017, cg242295681, cg225506581 |
| MIR3663 | 4 | cg05836659, cg08504601, cg13603914, cg033111851 |
| MIR3665 | 4 | cg23262272, cg13311392, cg16702144, cg01447389 |
| MIR3675 | 4 | cg17345633, cg25562878, cg229040861, cg132525491 |
| MIR3679 | 4 | cg10382221, cg05096161, cg145131171, cg163214831 |
| MIR378I | 4 | cg04630982, cg26326746, cg083534461, cg121410881 |
| MIR3939 | 4 | cg13280484, cg10605649, cg00058903, cg12004639 |
| MIR3945 | 4 | cg26980256, cg15818827, cg022237831, cg088270752 |
| MIR3960 | 4 | cg15908975, cg11694513, cg03326399, cg000025311 |
| MIR4280 | 4 | cg14158468, cg25171729, cg042516611, cg118337351 |
| MIR4313 | 4 | cg15028514, cg06660530, cg128775241, cg154419991 |
| MIR4323 | 4 | cg02882979, cg19560831, cg18863119, cg14667980 |
| MIR4442 | 4 | cg04660733, cg16241842, cg116977121, cg124885771 |
| MIR4465 | 4 | cg27422060, cg22576612, cg159748311, cg046751911 |
| MIR4487 | 4 | cg07407950, cg27417567, cg061144654, cg206107402 |
| MIR4491 | 4 | cg05237332, cg12897503, cg261224221, cg158031031 |
| MIR4514 | 4 | cg13097741, cg07835236, cg078731501, cg219989671 |
| MIR4519 | 4 | cg10549753, cg11212178, cg08922308, cg034444231 |
| MIR452 | 4 | cg03815480, cg20361881, cg02714331, cg014642471 |
| MIR4526 | 4 | cg10181798, cg00884188, cg25144602, cg10363208 |
| MIR4669 | 4 | cg08380316, cg26430142, cg22461390, cg06742077 |
| MIR4691 | 4 | cg07471156, cg27617225, cg03515927, cg00503998 |
| MIR4757 | 4 | cg19617599, cg25191231, cg186727591, cg025117501 |
| MIR4781 | 4 | cg19872746, cg07494239, cg21063313, cg09620514 |
| MIR4792 | 4 | cg08342762, cg01033787, cg151689421, cg134231801 |
| MIR486-2 | 4 | cg07569575, cg11855741, cg23573591, cg04354589 |
| MIR488 | 4 | cg27538026, cg15434337, cg12678006, cg03425468 |
| MIR490 | 4 | cg26566472, cg23863230, cg10269619, cg18190776 |
| MIR5003 | 4 | cg18772588, cg22192554, cg108157451, cg107454131 |
| MIR512-1 | 4 | cg23029159, cg19477205, cg06265809, cg110690711 |
| MIR512-2 | 4 | cg11069071, cg230291591, cg194772051, cg062658091 |
| MIR516A2 | 4 | cg22823821, cg18166152, cg181633641, cg001824161 |
| MIR516B1 | 4 | cg08207582, cg08342708, cg171299431, cg142096771 |
| MIR516B2 | 4 | cg01845432, cg11590435, cg139249541, cg043701821 |

| | | |
|---|---|---|
| MIR517A | 4 | cg16269274, cg118692691, cg204747881, cg276644181 |
| MIR518A2 | 4 | cg14313347, cg08709097, cg094305861, cg230614121 |
| MIR519B | 4 | cg19631264, cg26116969, cg05345310, cg167900551 |
| MIR519D | 4 | cg11869269, cg20474788, cg17007012, cg045733161 |
| MIR520A | 4 | cg21407354, cg08378742, cg187107101, cg107536361 |
| MIR520D | 4 | cg05940049, cg15157289, cg258437051, cg229212281 |
| MIR524 | 4 | cg09096159, cg25559898, cg164666131, cg239089431 |
| MIR527 | 4 | cg24877449, cg12625872, cg053821371, cg027004791 |
| MIR548L | 4 | cg06921261, cg08207531, cg17340314, cg06257733 |
| MIR548O | 4 | cg03949274, cg19277411, cg12318602, cg21687237 |
| MIR555 | 4 | cg20713662, cg09704056, cg14007733, cg17308926 |
| MIR5572 | 4 | cg06677980, cg13488877, cg23018181, cg02864240 |
| MIR567 | 4 | cg10225197, cg02571840, cg01336796, cg12337227 |
| MIR5680 | 4 | cg23104816, cg20635067, cg17327665, cg04292889 |
| MIR5703 | 4 | cg17592802, cg17762933, cg02405538, cg02851043 |
| MIR573 | 4 | cg18876162, cg06921368, cg183634171, cg085158691 |
| MIR588 | 4 | cg03599197, cg16673522, cg019794601, cg195820001 |
| MIR591 | 4 | cg12533689, cg08396445, cg16473384, cg14131313 |
| MIR592 | 4 | cg09538819, cg26518884, cg06549863, cg10123619 |
| MIR599 | 4 | cg03000711, cg09433733, cg05142576, cg01704860 |
| MIR600 | 4 | cg23391045, cg08314089, cg15355620, cg24142470 |
| MIR600HG | 4 | cg14926083, cg03695608, cg017315181, cg133043251 |
| MIR601 | 4 | cg01674169, cg07133355, cg27008901, cg24190430 |
| MIR604 | 4 | cg08316073, cg06333435, cg11699593, cg10206887 |
| MIR6072 | 4 | cg21153040, cg15972425, cg004154551, cg274380671 |
| MIR613 | 4 | cg02277272, cg04582108, cg06265451, cg04169578 |
| MIR617 | 4 | cg07815919, cg24795721, cg06624369, cg13644295 |
| MIR619 | 4 | cg06033953, cg07313836, cg05046382, cg05392988 |
| MIR620 | 4 | cg03415664, cg15325530, cg27072201, cg14728609 |
| MIR634 | 4 | cg04594986, cg10325896, cg02839951, cg17050183 |
| MIR648 | 4 | cg08041140, cg26208764, cg09662531, cg06222917 |
| MIR649 | 4 | cg07924363, cg18719157, cg217760911, cg168064581 |
| MIR6504 | 4 | cg16510860, cg22182936, cg13003178, cg19947060 |
| MIR651 | 4 | cg23192918, cg17488785, cg187031091, cg064534711 |
| MIR670HG | 4 | cg01737592, cg21210537, cg044586455, cg185146445 |
| MIR6831 | 4 | cg01598284, cg12809539, cg04323831, cg10782455 |
| MIR6840 | 4 | cg13332552, cg22576711, cg01003872, cg11214083 |
| MIR6872 | 4 | cg03724445, cg25102782, cg06333167, cg09890200 |
| MIR6880 | 4 | cg03757043, cg11297378, cg15806381, cg12303909 |
| MIR6889 | 4 | cg22144942, cg02054724, cg18824446, cg09160955 |
| MIR708 | 4 | cg21445603, cg05687603, cg04901018, cg16512376 |
| MIR711 | 4 | cg04027132, cg17201638, cg26902777, cg05147708 |
| MIR7152 | 4 | cg20239358, cg21565218, cg09761153, cg01491603 |
| MIR770 | 4 | cg11697653, cg17378265, cg02085369, cg00130307 |
| MIR7704 | 4 | cg19152529, cg20386024, cg27665913, cg14788420 |
| MIR7845 | 4 | cg02874133, cg22686872, cg24783876, cg10621390 |
| MIR8081 | 4 | cg04740198, cg00735591, cg030164861, cg137811671 |
| MIR892C | 4 | cg22262544, cg01374885, cg020601851, cg013402861 |
| MIR9-2 | 4 | cg14394740, cg10561392, cg21419939, cg14196304 |
| MIR938 | 4 | cg25284208, cg08264885, cg14294245, cg22217144 |
| MIR944 | 4 | cg23220346, cg26160492, cg04787317, cg08171483 |
| MIR95 | 4 | cg22067839, cg14777519, cg18775606, cg15085006 |
| MIR98 | 4 | cg09918657, cg10420310, cg04018325, cg06273075 |
| MIR99A | 4 | cg23818401, cg073440191, cg239664761, cg181497941 |
| MIRLET7A1 | 4 | cg06036509, cg03638677, cg235457741, cg025586842 |
| MIRLET7F1 | 4 | cg23545774, cg26994774, cg060365091, cg099029082 |
| MIRLET7G | 4 | cg00352349, cg21446511, cg20370152, cg05106421 |
| MIR144 | 4 | cg095327261, cg234835621, cg217946651, cg164307261 |
| MIR20A | 4 | cg237417021, cg267537812, cg096619242, cg267894123 |
| MIR302B | 4 | cg151657351, cg211756852, cg210107154, cg016281814 |
| MIR548AA2 | 4 | cg242332241, cg133205851, cg204814191, cg244370101 |
| MIR92A2 | 4 | cg079947861, cg059247415, cg129660055, cg085586525 |
| MIR1-2 | 3 | cg12411817, cg16185223, cg240258591 |
| MIR100 | 3 | cg11728484, cg19177732, cg04819135 |
| MIR103-1 | 3 | cg16226436, cg00060683, cg06386451 |
| MIR1183 | 3 | cg20818807, cg26953669, cg02877191 |
| MIR1200 | 3 | cg17844867, cg18839354, cg24735829 |
| MIR1206 | 3 | cg18486707, cg03341723, cg25026287 |
| MIR1245A | 3 | cg11503425, cg10020120, cg05617050 |
| MIR1262 | 3 | cg16698293, cg13669156, cg02058215 |
| MIR1273A | 3 | cg12065914, cg01627201, cg10030624 |
| MIR1277 | 3 | cg04127262, cg21489445, cg27415713 |
| MIR1279 | 3 | cg15139258, cg12395420, cg097969111 |
| MIR1298 | 3 | cg04779243, cg24975947, cg00843452 |
| MIR1304 | 3 | cg05775586, cg21576021, cg07725673 |
| MIR15A | 3 | cg04580583, cg155090001, cg238067741 |
| MIR1825 | 3 | cg21091128, cg14521367, cg25588576 |
| MIR1912 | 3 | cg08521332, cg13031987, cg24246947 |
| MIR1972-1 | 3 | cg02229135, cg05446322, cg22037492 |
| MIR1979 | 3 | cg17918158, cg25199192, cg01443549 |
| MIR208A | 3 | cg19760211, cg15905865, cg02458318 |
| MIR20B | 3 | cg05924741, cg12966005, cg08558652 |
| MIR2355 | 3 | cg03065507, cg03726156, cg17190157 |
| MIR3153 | 3 | cg12482648, cg07426000, cg22574343 |
| MIR3154 | 3 | cg24044186, cg093444851, cg113642901 |
| MIR3155A | 3 | cg06292304, cg24185864, cg18246262 |
| MIR3166 | 3 | cg20963107, cg07955015, cg01599028 |
| MIR3199-2 | 3 | cg02012771, cg03387238, cg02426940 |
| MIR32 | 3 | cg12693702, cg00399596, cg16447012 |
| MIR320C1 | 3 | cg07923716, cg14230816, cg15745972 |
| MIR335 | 3 | cg07072293, cg11941971, cg19089725 |
| MIR361 | 3 | cg20671702, cg06682219, cg11725326 |
| MIR3614 | 3 | cg27111395, cg25536196, cg01855585 |
| MIR3655 | 3 | cg18321315, cg27292264, cg05801066 |
| MIR384 | 3 | cg04271870, cg27069588, cg250473061 |
| MIR3909 | 3 | cg10815745, cg10745413, cg01207916 |
| MIR4254 | 3 | cg25059646, cg17342214, cg16183402 |
| MIR4434 | 3 | cg09938677, cg08068800, cg10965259 |
| MIR4453 | 3 | cg25091526, cg22952011, cg19229209 |
| MIR4470 | 3 | cg16370061, cg03834394, cg03341985 |
| MIR448 | 3 | cg15859072, cg09945985, cg15832560 |
| MIR4524A | 3 | cg21242212, cg03918703, cg137857791 |
| MIR4534 | 3 | cg11479035, cg22243298, cg13892257 |
| MIR4536-1 | 3 | cg22550658, cg06916001, cg096070471 |
| MIR4655 | 3 | cg10713842, cg04933317, cg12289524 |
| MIR4667 | 3 | cg23766333, cg23841711, cg19521384 |
| MIR4674 | 3 | cg25673948, cg11998141, cg09177567 |
| MIR4678 | 3 | cg10471143, cg14140920, cg11611793 |
| MIR4688 | 3 | cg00293796, cg03317309, cg15943335 |
| MIR4692 | 3 | cg07625237, cg19069367, cg12903452 |
| MIR4697HG | 3 | cg15651103, cg17109533, cg048034021 |
| MIR4738 | 3 | cg08588953, cg24939330, cg26889764 |
| MIR5001 | 3 | cg23062120, cg15427166, cg18471021 |

| | | |
|---|---|---|
| MIR501 | 3 | cg19949931, cg16187303, cg028795542 |
| MIR504 | 3 | cg19981865, cg09839654, cg17205193 |
| MIR5090 | 3 | cg14294892, cg03529040, cg08570631 |
| MIR5091 | 3 | cg11521721, cg05411186, cg01090930 |
| MIR518F | 3 | cg21176597, cg14666926, cg123954201 |
| MIR519E | 3 | cg26207035, cg067530501, cg111791201 |
| MIR520B | 3 | cg01272212, cg211765971, cg178577911 |
| MIR522 | 3 | cg21664443, cg225428591, cg015749031 |
| MIR523 | 3 | cg23408471, cg151719621, cg000547411 |
| MIR532 | 3 | cg26775289, cg16159090, cg089832151 |
| MIR544A | 3 | cg16150798, cg02005336, cg22704775 |
| MIR548B | 3 | cg04252242, cg16919074, cg24518794 |
| MIR548K | 3 | cg20555305, cg03636657, cg17741937 |
| MIR548P | 3 | cg04016922, cg00814786, cg16318181 |
| MIR553 | 3 | cg10774435, cg02216570, cg23820245 |
| MIR554 | 3 | cg04938255, cg25107717, cg17992509 |
| MIR556 | 3 | cg00385751, cg09680704, cg05986111 |
| MIR559 | 3 | cg01334432, cg17860090, cg17848546 |
| MIR566 | 3 | cg20310435, cg17833257, cg11625255 |
| MIR569 | 3 | cg15928247, cg01966636, cg20806436 |
| MIR570 | 3 | cg26054057, cg01334081, cg062776381 |
| MIR571 | 3 | cg20687414, cg16615211, cg08430604 |
| MIR577 | 3 | cg09765831, cg09648366, cg21900069 |
| MIR578 | 3 | cg20530613, cg13409084, cg11019088 |
| MIR5787 | 3 | cg13411604, cg14807549, cg23927983 |
| MIR579 | 3 | cg11168235, cg05642802, cg03059470 |
| MIR580 | 3 | cg08331946, cg08905723, cg25826640 |
| MIR581 | 3 | cg00648285, cg03021388, cg21589858 |
| MIR587 | 3 | cg21766808, cg17223176, cg27389764 |
| MIR603 | 3 | cg03368399, cg21925296, cg06112727 |
| MIR608 | 3 | cg08677259, cg22666787, cg19496363 |
| MIR624 | 3 | cg05387457, cg04686790, cg11165626 |
| MIR633 | 3 | cg11477273, cg26532527, cg22719998 |
| MIR640 | 3 | cg13098800, cg01709493, cg02975060 |
| MIR653 | 3 | cg04787349, cg130831861, cg062659991 |
| MIR660 | 3 | cg02528154, cg091956571, cg083180851 |
| MIR663B | 3 | cg06878166, cg14959721, cg12123924 |
| MIR6740 | 3 | cg11460208, cg14156134, cg27097224 |
| MIR6742 | 3 | cg24379599, cg09390927, cg07387629 |
| MIR6752 | 3 | cg05012516, cg08172304, cg05972303 |
| MIR6773 | 3 | cg21392700, cg03356172, cg13238479 |
| MIR6847 | 3 | cg10488199, cg26014966, cg14351526 |
| MIR6866 | 3 | cg19629686, cg07235355, cg17799287 |
| MIR6875 | 3 | cg14738611, cg03562412, cg03976364 |
| MIR761 | 3 | cg15984861, cg01959873, cg13716165 |
| MIR766 | 3 | cg01314715, cg10667857, cg21046624 |
| MIR7846 | 3 | cg01432902, cg12427039, cg10506390 |
| MIR8085 | 3 | cg18604419, cg08051194, cg18096939 |
| MIR8485 | 3 | cg03794445, cg20918933, cg11377865 |
| MIRLET7A2 | 3 | cg03121659, cg08942734, cg04299170 |
| MIRLET7BHG | 3 | cg19652186, cg00267482, cg207055181 |
| MIRLET7D | 3 | cg00752601, cg10873503, cg032756484 |
| MIRLET7DHG | 3 | cg15808266, cg16076191, cg007526011 |
| MIR1972-2 | 3 | cg022291351, cg054463221, cg220374921 |
| MIR202HG | 3 | cg259000851, cg054747261, cg273136421 |
| MIR3618 | 3 | cg216357061, cg259875141, cg242480071 |
| MIR548AA1 | 3 | cg117222271, cg135756061, cg119299601 |
| MIR7515 | 3 | cg029997111, cg202540841, cg252024071 |
| MIR875 | 3 | cg094337331, cg051425761, cg017048601 |
| MIR18B | 3 | cg129660052, cg085586522, cg059247413 |
| MIR450A2 | 3 | cg176730592, cg252223573, cg222242573 |
| MIR19B2 | 3 | cg059247414, cg129660054, cg085586524 |
| MIR101-2 | 2 | cg07439409, cg03108701 |
| MIR105-2 | 2 | cg02806824, cg097583611 |
| MIR1193 | 2 | cg01629329, cg215481092 |
| MIR1243 | 2 | cg08243711, cg18344769 |
| MIR1244-4 | 2 | cg03275648, cg05555502 |
| MIR1255A | 2 | cg05283542, cg24198004 |
| MIR1255B2 | 2 | cg10239863, cg02223139 |
| MIR1261 | 2 | cg00632587, cg07241951 |
| MIR1273C | 2 | cg11070777, cg158082661 |
| MIR1273F | 2 | cg11135080, cg22674756 |
| MIR1283-1 | 2 | cg14696444, cg093181581 |
| MIR1291 | 2 | cg20355301, cg00532885 |
| MIR1295 | 2 | cg08446735, cg11095157 |
| MIR1297 | 2 | cg13600227, cg065292641 |
| MIR1302-5 | 2 | cg00360534, cg08860879 |
| MIR1302-6 | 2 | cg02446672, cg14278514 |
| MIR1302-8 | 2 | cg13528713, cg12934576 |
| MIR16-1 | 2 | cg15509000, cg23806774 |
| MIR181A2 | 2 | cg11857310, cg12402823 |
| MIR181B2 | 2 | cg20332907, cg124028231 |
| MIR204 | 2 | cg06445043, cg06081793 |
| MIR205HG | 2 | cg18614984, cg185913041 |
| MIR214 | 2 | cg25824899, cg14805136 |
| MIR217 | 2 | cg04865412, cg136181901 |
| MIR219A2 | 2 | cg18161285, cg16321266 |
| MIR221 | 2 | cg06502071, cg16108835 |
| MIR224 | 2 | cg19654612, cg01464247 |
| MIR2682 | 2 | cg01118295, cg25434116 |
| MIR302E | 2 | cg13652372, cg256191914 |
| MIR3065 | 2 | cg04631545, cg24118155 |
| MIR31 | 2 | cg19562453, cg09111718 |
| MIR3115 | 2 | cg16129065, cg05617775 |
| MIR3124 | 2 | cg26045471, cg26455554 |
| MIR3142 | 2 | cg02004044, cg169011611 |
| MIR3143 | 2 | cg11660531, cg09438228 |
| MIR3148 | 2 | cg23908593, cg202393581 |
| MIR3171 | 2 | cg13959943, cg22745143 |
| MIR3181 | 2 | cg05047276, cg07160044 |
| MIR3192 | 2 | cg10059536, cg01520867 |
| MIR3201 | 2 | cg15056572, cg162692741 |
| MIR320B2 | 2 | cg19452298, cg25811451 |
| MIR320E | 2 | cg14942536, cg01533177 |
| MIR324 | 2 | cg17205233, cg04541021 |
| MIR325 | 2 | cg15307230, cg22916042 |
| MIR3591 | 2 | cg07710843, cg002873191 |
| MIR3610 | 2 | cg12316667, cg25771580 |
| MIR3615 | 2 | cg14030388, cg02658046 |
| MIR3650 | 2 | cg03617850, cg01227064 |
| MIR3651 | 2 | cg20999110, cg18605905 |
| MIR3659 | 2 | cg25024312, cg189832891 |
| MIR3660 | 2 | cg15398430, cg053674891 |

| | | |
|---|---|---|
| MIR3671 | 2 | cg19784244, cg13749777 |
| MIR3674 | 2 | cg15446050, cg264301421 |
| MIR3677 | 2 | cg22789730, cg162025091 |
| MIR3680-2 | 2 | cg08668140, cg05348982 |
| MIR378D1 | 2 | cg10046367, cg165758921 |
| MIR3911 | 2 | cg02090654, cg17540499 |
| MIR3925 | 2 | cg06099315, cg020742591 |
| MIR3974 | 2 | cg00047185, cg053056031 |
| MIR3976 | 2 | cg07604927, cg116789641 |
| MIR4257 | 2 | cg19827167, cg03327164 |
| MIR4262 | 2 | cg03529568, cg020944601 |
| MIR4276 | 2 | cg22059098, cg231072221 |
| MIR4290 | 2 | cg20889285, cg18051234 |
| MIR4293 | 2 | cg05922058, cg142167231 |
| MIR4299 | 2 | cg02452310, cg232252554 |
| MIR4311 | 2 | cg15803103, cg03242880 |
| MIR4317 | 2 | cg20894023, cg02322229 |
| MIR4319 | 2 | cg20488765, cg12798649 |
| MIR4326 | 2 | cg11935618, cg00089864 |
| MIR4327 | 2 | cg01972979, cg002243351 |
| MIR4429 | 2 | cg02520707, cg19841284 |
| MIR4433B | 2 | cg25687874, cg036723221 |
| MIR4439 | 2 | cg22687497, cg21503989 |
| MIR4452 | 2 | cg26815021, cg163261883 |
| MIR4471 | 2 | cg21300318, cg185495941 |
| MIR4475 | 2 | cg17602882, cg090083532 |
| MIR4479 | 2 | cg06460618, cg25295395 |
| MIR4488 | 2 | cg13589481, cg27593320 |
| MIR4492 | 2 | cg11931203, cg14282114 |
| MIR4497 | 2 | cg11199714, cg09890892 |
| MIR4499 | 2 | cg05410331, cg15167811 |
| MIR4510 | 2 | cg11177296, cg162719111 |
| MIR4513 | 2 | cg08698035, cg05737842 |
| MIR4515 | 2 | cg05412137, cg15215690 |
| MIR4535 | 2 | cg16719099, cg170070121 |
| MIR4633 | 2 | cg10620048, cg05966699 |
| MIR4634 | 2 | cg20273576, cg230662341 |
| MIR4636 | 2 | cg24426290, cg05026437 |
| MIR4643 | 2 | cg22806068, cg161524801 |
| MIR4648 | 2 | cg17874329, cg22311686 |
| MIR4651 | 2 | cg26777800, cg12532854 |
| MIR4659B | 2 | cg01023783, cg01516119 |
| MIR4660 | 2 | cg00433107, cg252953951 |
| MIR4662B | 2 | cg20893022, cg128349261 |
| MIR4677 | 2 | cg12636482, cg19093225 |
| MIR4679-1 | 2 | cg03205495, cg214526331 |
| MIR4679-2 | 2 | cg08434594, cg117810691 |
| MIR4681 | 2 | cg17886457, cg10715527 |
| MIR4686 | 2 | cg19712386, cg10500570 |
| MIR4687 | 2 | cg24919348, cg10659042 |
| MIR4689 | 2 | cg06883956, cg15903571 |
| MIR4707 | 2 | cg21105876, cg08653328 |
| MIR4708 | 2 | cg05882315, cg236717951 |
| MIR4712 | 2 | cg27419751, cg264317731 |
| MIR4721 | 2 | cg03742132, cg08595690 |
| MIR4734 | 2 | cg17943520, cg218432771 |
| MIR4740 | 2 | cg06593686, cg15422361 |
| MIR4741 | 2 | cg07591882, cg14417226 |
| MIR4752 | 2 | cg02582514, cg05297328 |
| MIR4761 | 2 | cg15241106, cg24212738 |
| MIR4774 | 2 | cg17166334, cg27592871 |
| MIR4785 | 2 | cg12740087, cg14502628 |
| MIR4790 | 2 | cg07505964, cg027971081 |
| MIR486-1 | 2 | cg08981957, cg13786191 |
| MIR5004 | 2 | cg21238254, cg04288299 |
| MIR5089 | 2 | cg00227156, cg079301211 |
| MIR5093 | 2 | cg14202820, cg074763281 |
| MIR5094 | 2 | cg16521032, cg05776138 |
| MIR510 | 2 | cg27167979, cg058837571 |
| MIR5100 | 2 | cg20630207, cg174427232 |
| MIR513A1 | 2 | cg14017986, cg203434671 |
| MIR5197 | 2 | cg11600078, cg209218081 |
| MIR519A2 | 2 | cg18163364, cg228238211 |
| MIR521-1 | 2 | cg15073264, cg136002271 |
| MIR548A3 | 2 | cg00018216, cg219381791 |
| MIR548AS | 2 | cg26242772, cg114322501 |
| MIR548F2 | 2 | cg25486386, cg14691596 |
| MIR548F4 | 2 | cg11367853, cg03135982 |
| MIR548H1 | 2 | cg17442723, cg11478495 |
| MIR548H5 | 2 | cg18268164, cg257608881 |
| MIR550A2 | 2 | cg27091642, cg102652541 |
| MIR552 | 2 | cg24818690, cg036987811 |
| MIR5571 | 2 | cg06268905, cg076049271 |
| MIR561 | 2 | cg11941066, cg20428383 |
| MIR5692B | 2 | cg25512249, cg204814192 |
| MIR5695 | 2 | cg24369310, cg060817931 |
| MIR5696 | 2 | cg25064770, cg17864399 |
| MIR5700 | 2 | cg17857791, cg150906441 |
| MIR5705 | 2 | cg17205803, cg094421063 |
| MIR5708 | 2 | cg05444640, cg112888191 |
| MIR583 | 2 | cg20292484, cg097536321 |
| MIR605 | 2 | cg10734892, cg12489601 |
| MIR6076 | 2 | cg04379522, cg194917761 |
| MIR6082 | 2 | cg25538649, cg101236191 |
| MIR6087 | 2 | cg07927718, cg249658001 |
| MIR6124 | 2 | cg19681251, cg24315859 |
| MIR6132 | 2 | cg06169041, cg10857203 |
| MIR6165 | 2 | cg12538845, cg11292807 |
| MIR621 | 2 | cg10682141, cg06311238 |
| MIR622 | 2 | cg02700479, cg185542161 |
| MIR625 | 2 | cg14141340, cg05678252 |
| MIR626 | 2 | cg14906130, cg12556644 |
| MIR630 | 2 | cg18627360, cg132276991 |
| MIR6509 | 2 | cg09149005, cg19713787 |
| MIR663A | 2 | cg24454784, cg06218726 |
| MIR6716 | 2 | cg24178514, cg06932691 |
| MIR6729 | 2 | cg07720540, cg07830126 |
| MIR6746 | 2 | cg03331035, cg16657434 |
| MIR6748 | 2 | cg01609214, cg18975690 |
| MIR6751 | 2 | cg22358108, cg17395859 |
| MIR6753 | 2 | cg02369245, cg11861389 |
| MIR6766 | 2 | cg24219985, cg10989175 |
| MIR6775 | 2 | cg05623392, cg12062668 |

| | | |
|---|---|---|
| MIR6778 | 2 | cg24558576, cg12646800 |
| MIR6781 | 2 | cg19723657, cg04908961 |
| MIR6786 | 2 | cg09523808, cg09948795 |
| MIR6789 | 2 | cg08635913, cg24438311 |
| MIR6791 | 2 | cg05371620, cg18141891 |
| MIR6796 | 2 | cg17613498, cg01078903 |
| MIR6803 | 2 | cg10529599, cg04578631 |
| MIR6817 | 2 | cg13227699, cg06586700 |
| MIR6822 | 2 | cg25942990, cg01152019 |
| MIR6824 | 2 | cg20587874, cg19648023 |
| MIR6830 | 2 | cg13717684, cg01397968 |
| MIR6849 | 2 | cg23300129, cg22395021 |
| MIR6851 | 2 | cg14756724, cg27442580 |
| MIR6852 | 2 | cg25199591, cg06138966 |
| MIR6869 | 2 | cg16695570, cg04563422 |
| MIR6877 | 2 | cg23380552, cg01912428 |
| MIR6878 | 2 | cg17835369, cg02993630 |
| MIR6890 | 2 | cg03012876, cg00534253 |
| MIR6895 | 2 | cg11631342, cg00138641 |
| MIR7-1 | 2 | cg02471248, cg17198132 |
| MIR7-3HG | 2 | cg09189978, cg003298031 |
| MIR7106 | 2 | cg11740035, cg00410048 |
| MIR7109 | 2 | cg12877524, cg15441999 |
| MIR7153 | 2 | cg07230015, cg183226581 |
| MIR7156 | 2 | cg19238448, cg01067603 |
| MIR764 | 2 | cg11032038, cg26563248 |
| MIR767 | 2 | cg09557888, cg09758361 |
| MIR7702 | 2 | cg08207256, cg089950671 |
| MIR7703 | 2 | cg11629527, cg14216723 |
| MIR7847 | 2 | cg04559779, cg06610119 |
| MIR7974 | 2 | cg00752143, cg13435266 |
| MIR7977 | 2 | cg20751048, cg195508901 |
| MIR8065 | 2 | cg08924004, cg138827481 |
| MIR8067 | 2 | cg27585830, cg221170791 |
| MIR8070 | 2 | cg24565698, cg267336454 |
| MIR8072 | 2 | cg22921228, cg088240121 |
| MIR8074 | 2 | cg08131204, cg274175671 |
| MIR8086 | 2 | cg12846938, cg116514431 |
| MIR8089 | 2 | cg09618015, cg01026256 |
| MIR920 | 2 | cg08849095, cg00351472 |
| MIR1244-2 | 2 | cg032756481, cg055555021 |
| MIR3680-1 | 2 | cg086681401, cg053489821 |
| MIR663AHG | 2 | cg244547841, cg062187261 |
| MIR1244-3 | 2 | cg032756482, cg055555022 |
| MIR323A | 2 | cg121580552, cg061499712 |
| MIR1244-1 | 2 | cg032756483, cg055555023 |
| MIR103-2AS | 1 | cg03401997 |
| MIR103A1 | 1 | cg15334941 |
| MIR1201 | 1 | cg17292885 |
| MIR1245B | 1 | cg10410507 |
| MIR1269B | 1 | cg02298831 |
| MIR1271 | 1 | cg01543197 |
| MIR1273G | 1 | cg24700993 |
| MIR128-1 | 1 | cg26289892 |
| MIR1293 | 1 | cg02324722 |
| MIR1302-2 | 1 | cg15543621 |
| MIR219B | 1 | cg00314904 |
| MIR220B | 1 | cg27267198 |
| MIR2681 | 1 | cg09260946 |
| MIR28 | 1 | cg13383435 |
| MIR3074 | 1 | cg21020852 |
| MIR3122 | 1 | cg03992926 |
| MIR3125 | 1 | cg13061767 |
| MIR3129 | 1 | cg20777186 |
| MIR3130-1 | 1 | cg15327395 |
| MIR3131 | 1 | cg11390344 |
| MIR3132 | 1 | cg14511781 |
| MIR3145 | 1 | cg27212978 |
| MIR3151 | 1 | cg17711252 |
| MIR3155B | 1 | cg02690648 |
| MIR3160-2 | 1 | cg22679949 |
| MIR3167 | 1 | cg01024878 |
| MIR3173 | 1 | cg08789645 |
| MIR3174 | 1 | cg21913159 |
| MIR3176 | 1 | cg24789224 |
| MIR3177 | 1 | cg12023318 |
| MIR3183 | 1 | cg13652338 |
| MIR3186 | 1 | cg27170797 |
| MIR3188 | 1 | cg05216211 |
| MIR3193 | 1 | cg17188759 |
| MIR3611 | 1 | cg21111824 |
| MIR3613 | 1 | cg11465913 |
| MIR3616 | 1 | cg21190038 |
| MIR3619 | 1 | cg20705518 |
| MIR3646 | 1 | cg14864357 |
| MIR3654 | 1 | cg10252212 |
| MIR3661 | 1 | cg17164827 |
| MIR3664 | 1 | cg22384877 |
| MIR3666 | 1 | cg06772049 |
| MIR3667 | 1 | cg22185428 |
| MIR3678 | 1 | cg20871143 |
| MIR3686 | 1 | cg01956472 |
| MIR3689B | 1 | cg17937101 |
| MIR3692 | 1 | cg09454295 |
| MIR3714 | 1 | cg16321483 |
| MIR371B | 1 | cg12782201 |
| MIR378B | 1 | cg14471282 |
| MIR378H | 1 | cg12509383 |
| MIR3907 | 1 | cg03220633 |
| MIR3913-2 | 1 | cg01920129 |
| MIR3921 | 1 | cg01822124 |
| MIR3926-2 | 1 | cg20921808 |
| MIR3935 | 1 | cg22982536 |
| MIR3936 | 1 | cg04885775 |
| MIR3938 | 1 | cg21868410 |
| MIR3972 | 1 | cg08540942 |
| MIR3973 | 1 | cg25847416 |
| MIR3978 | 1 | cg05992347 |
| MIR4258 | 1 | cg16789076 |
| MIR4259 | 1 | cg19811108 |
| MIR4260 | 1 | cg27294431 |
| MIR4265 | 1 | cg05346981 |
| MIR4269 | 1 | cg26471191 |

| | | |
|---|---|---|
| MIR4271 | 1 | cg06226973 |
| MIR4274 | 1 | cg00979307 |
| MIR4279 | 1 | cg12196473 |
| MIR4281 | 1 | cg18042072 |
| MIR4282 | 1 | cg25926549 |
| MIR4287 | 1 | cg22941668 |
| MIR4292 | 1 | cg22246148 |
| MIR4304 | 1 | cg16843250 |
| MIR4314 | 1 | cg01640572 |
| MIR4315-2 | 1 | cg21994794 |
| MIR4321 | 1 | cg09241708 |
| MIR4322 | 1 | cg23682215 |
| MIR4324 | 1 | cg01648556 |
| MIR4329 | 1 | cg06617961 |
| MIR4423 | 1 | cg10164186 |
| MIR4437 | 1 | cg00931692 |
| MIR4441 | 1 | cg04221848 |
| MIR4444-1 | 1 | cg27628857 |
| MIR4466 | 1 | cg18348975 |
| MIR4469 | 1 | cg05851148 |
| MIR4482 | 1 | cg02249596 |
| MIR4500 | 1 | cg10481470 |
| MIR4503 | 1 | cg26945813 |
| MIR4505 | 1 | cg04681122 |
| MIR4516 | 1 | cg16098340 |
| MIR4524B | 1 | cg13785779 |
| MIR4525 | 1 | cg01257685 |
| MIR4529 | 1 | cg03916245 |
| MIR4531 | 1 | cg02044725 |
| MIR4536-2 | 1 | cg09607047 |
| MIR4538 | 1 | cg21903890 |
| MIR4540 | 1 | cg08687105 |
| MIR4635 | 1 | cg22366214 |
| MIR4637 | 1 | cg03851861 |
| MIR4638 | 1 | cg21545762 |
| MIR4647 | 1 | cg16575892 |
| MIR4656 | 1 | cg12344891 |
| MIR4657 | 1 | cg08589987 |
| MIR4665 | 1 | cg18863314 |
| MIR4673 | 1 | cg05650680 |
| MIR4683 | 1 | cg21867571 |
| MIR4696 | 1 | cg25526243 |
| MIR4706 | 1 | cg17636008 |
| MIR4715 | 1 | cg05363534 |
| MIR4718 | 1 | cg26305717 |
| MIR4722 | 1 | cg03471320 |
| MIR4724 | 1 | cg11725835 |
| MIR4727 | 1 | cg17926458 |
| MIR4730 | 1 | cg21677210 |
| MIR4733 | 1 | cg22476237 |
| MIR4743 | 1 | cg18730515 |
| MIR4748 | 1 | cg06549228 |
| MIR4751 | 1 | cg26238975 |
| MIR4755 | 1 | cg19481727 |
| MIR4758 | 1 | cg12392104 |
| MIR4766 | 1 | cg08616234 |
| MIR4767 | 1 | cg09066676 |
| MIR4769 | 1 | cg26945867 |
| MIR4777 | 1 | cg07469205 |
| MIR4787 | 1 | cg24808667 |
| MIR4793 | 1 | cg03808674 |
| MIR4794 | 1 | cg06360841 |
| MIR4795 | 1 | cg11548914 |
| MIR4797 | 1 | cg07816047 |
| MIR4799 | 1 | cg18114034 |
| MIR4802 | 1 | cg02145866 |
| MIR4999 | 1 | cg23377000 |
| MIR5006 | 1 | cg09986564 |
| MIR500B | 1 | cg16202509 |
| MIR5087 | 1 | cg00405232 |
| MIR509-2 | 1 | cg02714692 |
| MIR517B | 1 | cg03663955 |
| MIR517C | 1 | cg11721554 |
| MIR518A1 | 1 | cg05138957 |
| MIR5191 | 1 | cg24669741 |
| MIR548AD | 1 | cg05922993 |
| MIR548AL | 1 | cg09767648 |
| MIR548AQ | 1 | cg12488577 |
| MIR548AR | 1 | cg22624321 |
| MIR548AT | 1 | cg02297838 |
| MIR550B1 | 1 | cg16167943 |
| MIR5583-1 | 1 | cg09764150 |
| MIR5584 | 1 | cg06379876 |
| MIR5588 | 1 | cg11697712 |
| MIR5591 | 1 | cg18697991 |
| MIR5685 | 1 | cg23398781 |
| MIR5690 | 1 | cg24951514 |
| MIR5699 | 1 | cg12616174 |
| MIR586 | 1 | cg04889413 |
| MIR6069 | 1 | cg23121785 |
| MIR6071 | 1 | cg15644668 |
| MIR6075 | 1 | cg14976283 |
| MIR6080 | 1 | cg15703728 |
| MIR610 | 1 | cg20544279 |
| MIR6129 | 1 | cg15562377 |
| MIR615 | 1 | cg12017968 |
| MIR627 | 1 | cg24823998 |
| MIR642A | 1 | cg23399577 |
| MIR644 | 1 | cg14743553 |
| MIR6499 | 1 | cg04596122 |
| MIR6510 | 1 | cg07641807 |
| MIR6511B2 | 1 | cg12332613 |
| MIR6512 | 1 | cg09184742 |
| MIR6513 | 1 | cg23682214 |
| MIR652 | 1 | cg00677407 |
| MIR664B | 1 | cg09730359 |
| MIR6715A | 1 | cg00791136 |
| MIR6715B | 1 | cg05564644 |
| MIR6718 | 1 | cg06284898 |
| MIR6719 | 1 | cg13546609 |
| MIR6720 | 1 | cg09616647 |
| MIR6727 | 1 | cg25101291 |
| MIR6732 | 1 | cg01168343 |

| | | |
|---|---|---|
| MIR6735 | 1 | cg02482035 |
| MIR6736 | 1 | cg09160344 |
| MIR6741 | 1 | cg14676242 |
| MIR6743 | 1 | cg16189671 |
| MIR6749 | 1 | cg19997557 |
| MIR6755 | 1 | cg23976185 |
| MIR6756 | 1 | cg12452440 |
| MIR6757 | 1 | cg09951047 |
| MIR6758 | 1 | cg08838825 |
| MIR676 | 1 | cg00928596 |
| MIR6764 | 1 | cg14634569 |
| MIR6765 | 1 | cg04238983 |
| MIR6768 | 1 | cg11782684 |
| MIR6769A | 1 | cg20351875 |
| MIR6769B | 1 | ch.1.3642016F |
| MIR6772 | 1 | cg09180566 |
| MIR6774 | 1 | cg03640487 |
| MIR6776 | 1 | cg08871224 |
| MIR6777 | 1 | cg19409254 |
| MIR6779 | 1 | cg08604905 |
| MIR6780B | 1 | cg07550016 |
| MIR6784 | 1 | cg10740663 |
| MIR6798 | 1 | cg12573499 |
| MIR6800 | 1 | cg22423977 |
| MIR6801 | 1 | cg18444589 |
| MIR6802 | 1 | cg21513316 |
| MIR6807 | 1 | cg23456330 |
| MIR6809 | 1 | cg13379731 |
| MIR6811 | 1 | cg22247160 |
| MIR6819 | 1 | cg16318949 |
| MIR6820 | 1 | cg17457701 |
| MIR6823 | 1 | cg17766381 |
| MIR6834 | 1 | cg20680819 |
| MIR6836 | 1 | cg21387653 |
| MIR6837 | 1 | cg08729793 |
| MIR6838 | 1 | cg18124756 |
| MIR6845 | 1 | cg11304866 |
| MIR6846 | 1 | cg03824238 |
| MIR6855 | 1 | cg08791395 |
| MIR6856 | 1 | cg01088352 |
| MIR6858 | 1 | cg00766382 |
| MIR6860 | 1 | cg21254731 |
| MIR6863 | 1 | cg00967013 |
| MIR6876 | 1 | cg12675571 |
| MIR6879 | 1 | cg00694874 |
| MIR6882 | 1 | cg20206204 |
| MIR6886 | 1 | cg01587049 |
| MIR6887 | 1 | cg13192714 |
| MIR6893 | 1 | cg16511841 |
| MIR7108 | 1 | cg09099968 |
| MIR7110 | 1 | cg18094076 |
| MIR7113 | 1 | cg22638461 |
| MIR7155 | 1 | cg08573869 |
| MIR7160 | 1 | cg25966737 |
| MIR7844 | 1 | cg01630798 |
| MIR7848 | 1 | cg04259565 |
| MIR7850 | 1 | cg23375912 |
| MIR7851 | 1 | cg24104437 |
| MIR7852 | 1 | cg07034096 |
| MIR7854 | 1 | cg21364568 |
| MIR7973-2 | 1 | cg06466538 |
| MIR8077 | 1 | cg14361526 |
| MIR8078 | 1 | cg25763735 |
| MIR873 | 1 | cg15134168 |
| MIR876 | 1 | cg01823120 |
| MIR103-1AS | 1 | cg162264361 |
| MIR1302-10 | 1 | cg155436211 |
| MIR203B | 1 | cg061030641 |
| MIR3689D2 | 1 | cg179371011 |
| MIR4315-1 | 1 | cg219947941 |
| MIR4444-2 | 1 | cg276288571 |
| MIR4458 | 1 | cg241237241 |
| MIR4697 | 1 | cg156511031 |
| MIR503HG | 1 | cg146913071 |
| MIR509-1 | 1 | cg027146921 |
| MIR520H | 1 | cg117215541 |
| MIR6511B1 | 1 | cg123326131 |
| MIR1302-9 | 1 | cg155436212 |
| MIR3689E | 1 | cg179371012 |
| MIR509-3 | 1 | cg027146922 |
| MIR1302-11 | 1 | cg155436213 |
| MIR3689A | 1 | cg179371013 |
| MIR3689F | 1 | cg179371014 |
